# Temporal trends in associations between severe mental illness and risk of cardiovascular disease: A systematic review and meta-analysis

**DOI:** 10.1371/journal.pmed.1003960

**Published:** 2022-04-19

**Authors:** Amanda M Lambert, Helen M Parretti, Emma Pearce, Malcolm J Price, Mark Riley, Ronan Ryan, Natalie Tyldesley-Marshall, Tuba Saygın Avşar, Gemma Matthewman, Alexandra Lee, Khaled Ahmed, Maria Lisa Odland, Christoph U. Correll, Marco Solmi, Tom Marshall

**Affiliations:** 1 Institute of Applied Health Research, University of Birmingham, Birmingham, United Kingdom; 2 Norwich Medical School, University of East Anglia, Norwich, United Kingdom; 3 NIHR Birmingham Biomedical Research Centre, University Hospitals Birmingham NHS Foundation Trust and University of Birmingham, Birmingham, United Kingdom; 4 Department of Applied Health Research, University College London, London, United Kingdom; 5 Department of Obstetrics and Gynecology, St Olavs Hospital, Trondheim University Hospital, Trondheim, Norway; 6 Malawi-Liverpool-Wellcome Trust Research Institute, Blantyre, Malawi; 7 Institute of Life Course and Medical Sciences, University of Liverpool, United Kingdom; 8 The Zucker Hillside Hospital, Department of Psychiatry, Northwell Health, Glen Oaks, New York, United States of America; 9 Donald and Barbara Zucker School of Medicine at Hofstra/Northwell, Department of Psychiatry and Molecular Medicine, Hempstead, New York, United States of America; 10 Charité Universitätsmedizin Berlin, Department of Child and Adolescent Psychiatry, Berlin, Germany; 11 Department of Psychiatry, University of Ottawa, Ontario, Canada; 12 Department of Mental Health, The Ottawa Hospital, Ontario, Canada; 13 Ottawa Hospital Research Institute (OHRI), Clinical Epidemiology Program, University of Ottawa, Ottawa, Ontario, Canada; King’s College London, UNITED KINGDOM

## Abstract

**Background:**

Severe mental illness (SMI; schizophrenia, bipolar disorders (BDs), and other nonorganic psychoses) is associated with increased risk of cardiovascular disease (CVD) and CVD-related mortality. To date, no systematic review has investigated changes in population level CVD-related mortality over calendar time. It is unclear if this relationship has changed over time in higher-income countries with changing treatments.

**Methods and findings:**

To address this gap, a systematic review was conducted, to assess the association between SMI and CVD including temporal change. Seven databases were searched (last: November 30, 2021) for cohort or case**–**control studies lasting ≥1 year, comparing frequency of CVD mortality or incidence in high-income countries between people with versus without SMI. No language restrictions were applied. Random effects meta-analyses were conducted to compute pooled hazard ratios (HRs) and rate ratios, pooled standardised mortality ratios (SMRs), pooled odds ratios (ORs), and pooled risk ratios (RRs) of CVD in those with versus without SMI. Temporal trends were explored by decade. Subgroup analyses by age, sex, setting, world region, and study quality (Newcastle–Ottawa scale (NOS) score) were conducted. The narrative synthesis included 108 studies, and the quantitative synthesis 59 mortality studies (with (≥1,841,356 cases and 29,321,409 controls) and 28 incidence studies (≥401,909 cases and 14,372,146 controls). The risk of CVD-related mortality for people with SMI was higher than controls across most comparisons, except for total CVD-related mortality for BD and cerebrovascular accident (CVA) for mixed SMI. Estimated risks were larger for schizophrenia than BD. Pooled results ranged from SMR = 1.55 (95% confidence interval (CI): 1.33 to 1.81, *p* < 0.001), for CVA in people with BD to HR/rate ratio = 2.40 (95% CI: 2.25 to 2.55, *p* < 0.001) for CVA in schizophrenia. For schizophrenia and BD, SMRs and pooled HRs/rate ratios for CHD and CVD mortality were larger in studies with outcomes occurring during the 1990s and 2000s than earlier decades (1980s: SMR = 1.14, 95% CI: 0.57 to 2.30, *p* = 0.71; 2000s: SMR = 2.59, 95% CI: 1.93 to 3.47, *p* < 0.001 for schizophrenia and CHD) and in studies including people with younger age. The incidence of CVA, CVD events, and heart failure in SMI was higher than controls. Estimated risks for schizophrenia ranged from HR/rate ratio 1.25 (95% CI: 1.04 to 1.51, *p* = 0.016) for total CVD events to rate ratio 3.82 (95% CI: 3.1 to 4.71, *p* < 0.001) for heart failure. Incidence of CHD was higher in BD versus controls. However, for schizophrenia, CHD was elevated in higher-quality studies only. The HR/rate ratios for CVA and CHD were larger in studies with outcomes occurring after the 1990s. Study limitations include the high risk of bias of some studies as they drew a comparison cohort from general population rates and the fact that it was difficult to exclude studies that had overlapping populations, although attempts were made to minimise this.

**Conclusions:**

In this study, we found that SMI was associated with an approximate doubling in the rate ratio of CVD-related mortality, particularly since the 1990s, and in younger groups. SMI was also associated with increased incidence of CVA and CHD relative to control participants since the 1990s. More research is needed to clarify the association between SMI and CHD and ways to mitigate this risk.

## Abstract 2

Amanda Lambert and co-workers study associations between severe mental illness and cardiovascular disease outcomes over time.

## Author summary

**Why was this study done?:**

- Previous research has identified higher incidence and mortality from cardiovascular disease (CVD) in people with severe mental illness (SMI) than those without SMI.
- It is not known whether the gap has changed over time.

**What did the researchers do and find?:**

- We conducted a comprehensive systematic review and meta-analysis of 108 cohort and case–control studies, including over 30 million participants.
- Compared to the general population, people with SMI, particularly schizophrenia, generally had higher levels of cardiovascular-related mortality and a higher incidence of CVD.
- For schizophrenia and bipolar disorder (BD), the rate ratios for coronary heart disease (CHD) mortality and cardiovascular mortality were larger in the 1990s and 2000s than in earlier studies, and the rate ratios were larger in younger people.

**What do these findings mean?:**

- We confirmed a strong association between SMI and incidence of and mortality from CVD.
- This association became stronger in the 1990s and 2000s.

## Introduction

Mortality in people with mental disorders is more than 2.2 times that of the general population and more than 2.5 times in those with psychoses, according to a systematic review and meta-analysis of 148 studies [1]. A recent estimate suggested that mental health disorders could be associated with 14.3% of all deaths across the globe, corresponding to around 8 million deaths each year [1].

The increased premature mortality seems to be the highest in schizophrenia spectrum disorders (schizophrenia), who die around 14.5 years earlier than people without schizophrenia, according to data from 11 studies and 247,603 patients [2]. In a large national cohort study, the mortality rate ratio was 3.2 in 104,000 people with schizophrenia [3].

In bipolar disorder (BD), figures are also concerning, with life expectancy decreased by 9 to 12 years in males and 8 to 10.5 years in females [4]. In a large cohort study, the mortality rate ratio was around 2.4 in 58,000 participants with BD compared with the general population [3].

More than two-thirds of the increased mortality in people with mental health disorders has been estimated to be due to natural causes [1]. When looking at cause-specific mortality in those with mental health disorders, cardiovascular disease (CVD) plays a major role [5]. According to a recent large national cohort study in Taiwan, the standardised mortality ratio (SMR) for CVD-related mortality was 3.02 (95% confidence interval (CI): 2.80 to 3.24) in those with schizophrenia and 1.87 (95% CI: 1.70 to 2.05) for BD [3].

While excess mortality from CVD for people with severe mental illness (SMI) is established, the relationship with CVD incidence is less clear. CVD is associated with modifiable risk factors such as smoking, hypertension, obesity, dyslipidemia, diabetes, alcohol consumption, and physical inactivity. People with SMI are known to have higher prevalence of risk factors that are associated with CVD [6] and face barriers to healthcare that reduce their ability to benefit from effective treatment [7–11]. Despite high CVD-related mortality rates, CVD may remain undiagnosed in people with schizophrenia and BD. A recent study found that people with schizophrenia were 66% less likely to be diagnosed with CVD prior to CVD-related death than individuals without SMI [12]. Women with BD were 38% less likely than control participants to be diagnosed.

The disease burden of both SMI and CVD is greater among higher-income countries compared with middle- or lower-income countries. Mortality from CVD is particularly high among high-income countries (294 deaths per 100,000 compared with 239.9 globally) [13]. There is also variation in the prevalence of schizophrenia, with a higher prevalence in developed countries compared to less developed countries (median 3.3 versus 2.6 per 1,000) [14]. The course, severity, and outcome of schizophrenia are generally worse in high-income compared with low-income countries. For example, in a World Health Organization (WHO) 10-country study, remission of their condition was experienced by 62.7% of patients with schizophrenia in developing countries, compared with only 36.8% in developed countries [15]. The rates of disability-adjusted life years (DALYs) from schizophrenia and BD are higher among World Bank high-income countries than other countries. In 2019, the rate of DALYs in high-income countries was 244.18 per 100,000 for schizophrenia and 174.69 for BD. This compares with 195.25 and 100.84 globally [16].

There have been modest, but steady reductions in CVD incidence in the general populations of high-income countries in the last 3 decades [17]. According to the Global Burden of Disease study, incidence of coronary heart disease (CHD) and stroke decreased by 13% and 7%, respectively, in World Bank high-income countries [18] between 1990 and 2019. This may be due to overall improvements in levels of some risk factors, in particular smoking and blood pressure levels [19]. Improved treatments for CVD have also reduced mortality [20].

However, it remains unclear whether the risk of CVD diagnosis and mortality for SMI patients has changed over time.

According to one meta-analysis, the gap in premature mortality between people with SMI and the general population does not seem to have changed over time [2]. It remains unknown whether the gap has remained unchanged also for CVD-related mortality. A recent large cohort study did not describe substantial changes from 2005 to 2010 regarding CVD-related increased mortality in schizophrenia compared with the general population (SMR 3.16 to 3.02), although there was a 10% reduction in SMR for BD (2.08 to 1.87) [3]. However, another cohort study found a widening gap in CVD-related mortality between SMI and the general population [21].

Yet, to the best of our knowledge, a comprehensive systematic review and meta-analysis on the trend over time of increased CVD-related mortality and incidence in schizophrenia and BD is currently missing. Indeed, previous reviews of the association between SMI and CVD have mainly focused on either a single SMI diagnosis (schizophrenia and BD) or a single cardiovascular outcome (myocardial infarction and stroke) or considered CVD mortality separately from incidence, and some excluded unpublished or non-English language studies [5,22–29]. These previous reviews have shown inconsistencies in included studies and differences in the scale and direction of associations. There are also recent large studies not included in previous reviews [30].

Most importantly, none of the previous reviews specifically investigated time trend of CVD-related mortality risk in those with schizophrenia and BD.

An up-to-date, comprehensive, systematic review of the association between SMI and CVD incidence and mortality is therefore indicated. The aim of this systematic review and meta-analysis was to specifically investigate changes over time of CVD-related mortality and incidence, in addition to providing updated pooled estimates of increased CVD mortality and incidence in those with schizophrenia and BD compared with the general population in high-income countries.

## Methods

The present systematic review follows a prepublished publicly available protocol registered with PROSPERO (CRD42017058068) and adheres to Preferred Reporting Items for Systematic Review and Meta-Analyses (PRISMA) guidelines [31] and to Meta-analysis Of Observational Studies in Epidemiology (MOOSE) [32]. PRISMA 2020 checklist and abstract checklist are available in S1 and S2 Files, respectively. Minor deviations from the protocol are listed in S3 File.

### Inclusion criteria

#### Population

Eligible studies included participants aged 16 to 65 years at onset of psychotic disorder. The upper age limit was chosen because the onset of psychotic disorders is uncommon at older ages, but transient psychotic conditions are associated with dementia, making it difficult to distinguish between this and SMI [33,34]. Studies were included if at least 90% of participants were within the age range. Only studies in high-income countries (defined as International Monetary Fund “Advanced Economies” [35]: Western Europe, Canada, United States of America, Australia, New Zealand, Taiwan, South Korea, and Japan) were included due to differences between high- and low- or middle-income countries in the diagnosis, prevalence, treatment, and prognosis of SMI [7,8].

#### Exposure

The exposure of interest was a diagnosis of SMI (schizophrenia, other psychoses, and BD). SMI was defined by the Diagnostic and Statistical Manual of Mental Disorders (DSM), International Classification of Diseases (ICD), and other clinical coding systems (e.g., OPCRIT, OXMIS, and Read codes), operational systems, or clinical diagnosis recorded in patient notes [36–39]. Dissociative, multiple personality disorders, depression (in the absence of psychosis or BD), dementia, learning disabilities, and obsessive-compulsive disorder were excluded.

#### Comparator

Eligible studies compared people with SMI to control participants without SMI or to the general population. Participants could be in an inpatient, outpatient, or community setting.

#### Outcome

The primary outcomes were mortality from, or incidence of, CVD (i.e., CHD, cerebrovascular accident (CVA), or heart failure) subsequent to SMI diagnosis and its time trend. Studies that reported composite outcomes including both fatal and nonfatal CVD events were analysed together with incidence studies as the majority of CVD events in these studies were not fatal. Studies were eligible if they described methods to exclude CVD at baseline. However, some studies reporting CVD-related mortality did not exclude individuals with prevalent CVD. These studies were included only if a prevalence of CVD below 10% at baseline was reported. Studies reporting all circulatory disease mortality were included [40] because two-thirds of deaths from all circulatory disease (ICD-10 Chapter I00-I99) are from CHD or stroke. However, those reporting all incident circulatory disease were excluded as incident circulatory disease diagnoses are mainly CVD risk factors [41]. Studies reporting only CVD risk factors were excluded. Studies were eligible if the mean follow-up period was at least 1 year.

#### Study design

Designs could include cohort studies or case–control studies but not cross-sectional, case series, case reports, or uncontrolled studies. Case–control studies compared the odds of SMI compared with no SMI in people who died from, or were newly diagnosed with, CVD compared with matched control participants. See S4 File for full inclusion criteria.

### Search strategy

The following bibliographic databases were searched up to November 30, 2021: MEDLINE, EMBASE, and PsycINFO via Ovid and CINAHL via EBSCO. The British Library’s ZETOC and Web of Science Conference Proceedings Citation Index were searched to the same date for conference abstracts and the Cochrane Library CENTRAL database searched up to December 1, 2021 for relevant trials. Initial searches were performed in 2017 and updated in 2019 and again in 2021 using the same search methods but narrowing the searches to 2017 and 2019 onwards, respectively. No language restrictions were applied. Citations of relevant reviews and included studies were searched as was grey literature, including OpenGrey. Detailed search strategies are included in S5 File.

### Screening and data extraction

Pairs of reviewers, working independently, firstly screened titles and abstracts and secondly screened full texts. Disagreements were resolved by discussion and if necessary, by reference to a third independent reviewer. Data extraction was conducted by a first reviewer (AML) and checked by a second (EP) using a form adapted from a previous review [5] (S6 File). If necessary, study authors were contacted for more information.

Hazard ratios (HRs), relative risks, rate ratios, risk ratios (RR), odds ratios (ORs), SMRs, or standardised incidence ratios (SIRs) measuring the association between SMI and CVD were extracted or sufficient information to calculate them. Risk measures adjusted for factors related to cardiovascular risk (e.g., socioeconomic factors, comorbidities, smoking, and alcohol use) were extracted where available; otherwise, measures adjusted for age and sex only were extracted. Risk measures were extracted from graphs if not reported in the text. CIs for effect estimates were extracted or calculated from available information.

For studies reporting results for the same cohort that were stratified over calendar time periods, estimates for the whole period were selected if available; otherwise, the earliest time period was selected to avoid a “healthy survivor” effect [30,42–44]. However, for studies that reported SMRs with multiple periods of follow-up [45], actual and expected deaths were summed and the ratio calculated to provide an estimated overall SMR. SMRs for males and females were combined to give a figure for persons if that was unreported. CIs were calculated using the method described by Kirkwood and Sterne [46].

### Risk of bias assessment

Risk of bias was assessed using a version of the Newcastle–Ottawa scale (NOS) [47] adapted to include extra criteria on accuracy of outcome measurement and identification of confounders from another checklist for cohort studies [48] (S7 File). Risk of bias was assessed with the NOS independently by 2 researchers. Any discrepancies were discussed, and consensus reached with the aid of a third reviewer when required.

### Data synthesis

Narrative and quantitative synthesis of evidence were conducted, and study characteristics recorded in tables for all included studies. Where appropriate, meta-analysis was conducted using DerSimonian and Laird random effects models due to expected population, clinical, and methodological heterogeneity between studies [49]. Heterogeneity was measured using I^2^ and chi-squared statistics and 95% prediction intervals [50]. Associations between different SMIs, CVD outcomes, and type of effect estimate were presented in separate subgroups in forest plots and meta-analyses. Rate ratios were combined with HRs since they have the same interpretation.

Studies that may have included the same patients in overlapping periods of recruitment were not included in the same meta-analyses to avoid double counting outcomes. Rules were devised to determine which studies should be included if the period of overlap was greater than 10% of the total follow-up time (S8 File). Those with fewer exposed cases, or shorter follow-up durations, were excluded to minimise the risk of double counting events.

Where calculable, results for both sexes were included in the meta-analysis; otherwise, separate results for males and females were used. For each meta-analysis with more than 10 studies, potential publication bias or small study effects were assessed visually using funnel plots and formally tested using Egger test [51]. If evidence of significant bias was found, a random effects trim-and-fill procedure [52] was applied to estimate the effect of potential unpublished studies.

To assess temporal trends, studies were allocated to decades according to the median year of follow-up. Further analysis increased the granularity by allocating to 5-year periods according to the median year of follow-up.

All analyses were performed in STATA MP version 14.2 (StataCorp. 2015. Stata Statistical Software: Release 14. College Station, TX: StataCorp LP).

### Subgroup analyses

Analysis of prespecified subgroups was conducted. If there were at least 10 study results, random effects meta-regression was performed to explore potential sources of heterogeneity that were measured at study group level [53,54]. Temporal trends, (decade of study conduct), setting (community; community and inpatient; and inpatient), and world region (North America [USA and Canada]); United Kingdom; Scandinavia [Denmark, Finland and Sweden]; Other Western Europe and Israel; Oceania [Australia and New Zealand]; and Far East) were included as covariates in univariate meta-regressions for each subgroup combination of SMI diagnosis (schizophrenia and related conditions; BD), and each mortality outcome (all circulatory disease; CHD; and CVA). Heterogeneity was assessed for each meta-regression by comparing the I^2^ from the random effects meta-analysis and the residual I^2^ after fitting covariates. The amount of between-study variation accounted for by covariates was measured by calculating adjusted R^2^. *p*-Values for model fit were used to assess the association between covariates and SMI and outcomes [55,56]. Subgroup analyses of cohort studies were conducted by sex and by age group at baseline and assessed by forest plots. Studies that reported results for ages ≤60 years were plotted separately from results for older groups or all ages.

### Sensitivity analyses

Sensitivity analysis was performed to assess the effect of making different decisions on inclusion of studies in the meta-analyses, where studies were subject to overlapping periods of recruitment and thus may have included the same patients, as described above. The effect of inclusion of results for different age groups, medications, or follow-up periods was also assessed in sensitivity analyses.

Sensitivity analysis was conducted to test the effect of replacing included studies with studies that had been excluded due to overlapping recruitment periods. As there was only one case–control study, this was combined with cohort studies.

## Results

**Fig 1:**
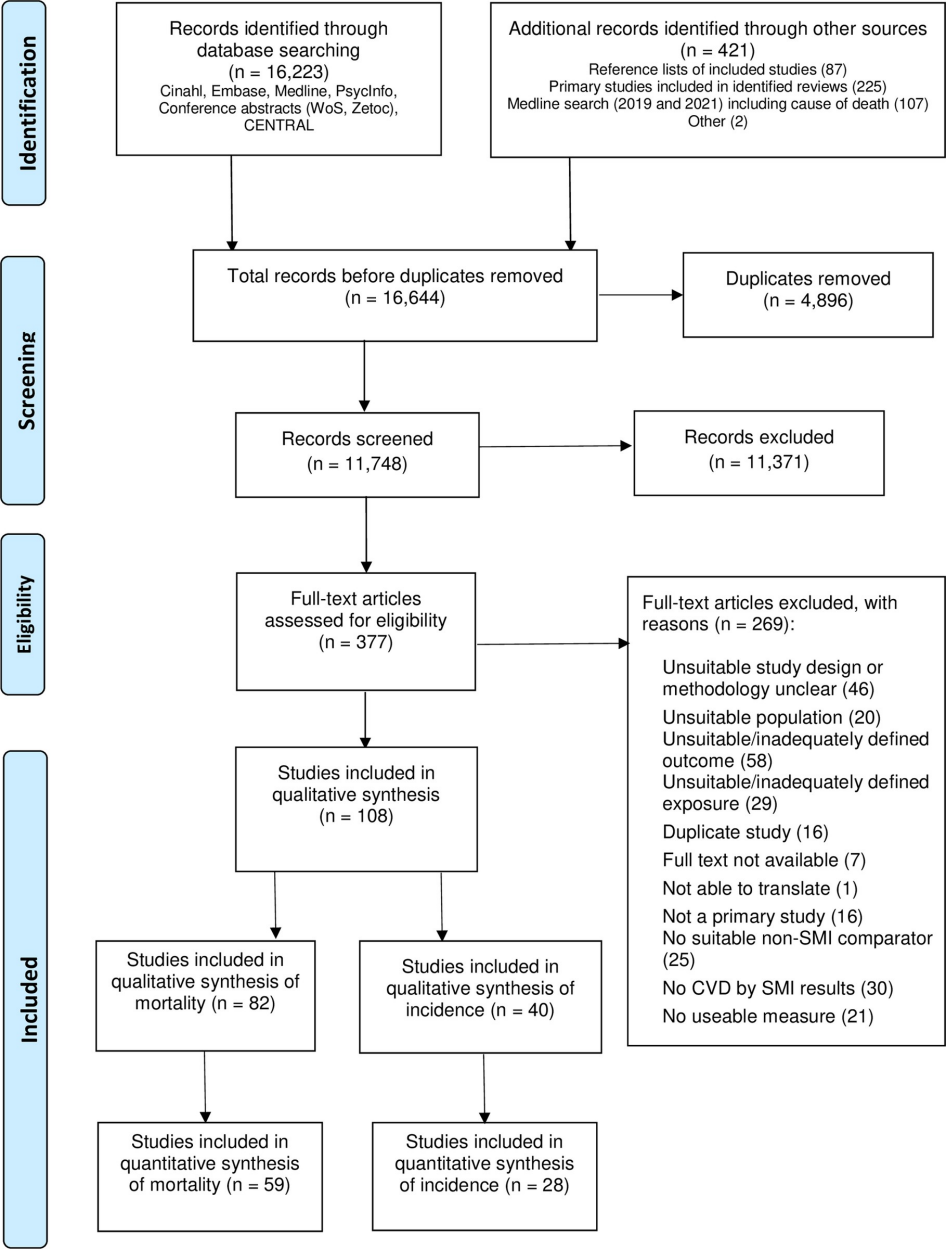
PRISMA flowchart. CVD, cardiovascular disease; SMI, severe mental illness.

Main searches identified 16,223 records (both CV incidence and mortality). After removal of duplicates, 11,748 titles and abstracts were screened, and 11,371 were excluded. Of 377 full-text articles assessed, 108 were included in the narrative review. A further 25 studies with overlapping periods of recruitment and 6 with insufficient information were excluded from the quantitative analysis, leaving 77 included in quantitative analysis of either CVD or CVD mortality. Of these, 59 reported CV mortality data, and 28 reported CV incidence (Fig 1). Mortality studies included in the narrative review are listed in S9 File and incidence studies in S10 File. Studies excluded from the narrative review are listed, with reasons, in S11 File.

### Characteristics of included studies

#### Mortality

All 82 studies included in the review reporting mortality outcomes were cohort studies (S12 File). Of these, 66 included patients with schizophrenia or related conditions, 30 included patients with BD, and 6 articles reported results for mixed SMI. For CVD mortality outcomes, 43 reported CHD, 31 reported CVA, 1 reported heart failure, and 54 reported all circulatory disease mortality. SMRs were the most common measure of relative risk (48 papers). The others reported rate ratios or HRs apart from a single study that reported ORs. Combined results for both sexes were obtained from most articles (k = 67). A total of 15 articles contributed outcomes from the 1970s or earlier, 18 from the 1980s, 20 from the 1990s, 29 from the 2000s, and 7 since 2010. Papers may have contributed results to more than 1 decade. Moreover, 38 of the 82 mortality studies were from Scandinavia, 13 from North America, 12 from Western Europe and Israel (excluding UK and Scandinavia), 11 from the UK, 5 from the Far East, and 3 from Oceania. A total of 41 studies were inpatient only; 17 were community only; and 24 were conducted in both inpatient and community settings. Furthermore, 5 studies that did not include CIs or standard errors were not included in the meta-analysis.

#### Incidence

CVD incidence was reported by 40 studies (S13 File): 1 case–control study and 39 cohort studies. Altogether, 29 articles included patients diagnosed with schizophrenia or related conditions and 21 included patients with BD. No studies reported results for mixed SMI. For cardiovascular outcomes, CHD was reported by 28 articles, CVA by 18, heart failure by 5, and incidence of total CVD events, defined mainly as combined heart disease and stroke, by 9. Rate ratios were reported by 13, HRs by 14, and ORs by 6; relative risks were obtained from 6 studies, and 1 reported SIRs. Results for all persons were reported by 33 articles, 6 reported males and females separately and 1 reported results for males only. No studies reported outcomes with a median decade of the 1970s or earlier; 6 reported results from the 1980s, 11 from the 1990s, 23 from the 2000s, and 1 since 2010. Moreover, 18 of the incidence studies were from Scandinavia; 10 from North America; and 7 were from the Far East. The others were from the UK, Australia, other Western Europe (excluding Scandinavia and UK), and Israel. A total of 12 studies were inpatients only, 19 were both inpatients and community, and 9 were set in the community only. One study [57] did not report CIs or standard errors, so we were unable to include those results in the meta-analysis.

Full forest plots for all studies included in the review are in S14 and S15 Files.

### Risk of bias of included studies

#### Mortality

Most studies that reported mortality outcomes were rated to have high risk of bias mainly due to lack of adjustment for confounders, lack of clarity about presence of CVD at baseline, and control participants being drawn from a different population to the SMI cohort (S16 File). Many studies assessed as being of higher risk of bias used general population mortality rates as the comparison. Only half the studies included persons with SMI in the community; the other half included only inpatients. Ascertainment of SMI was good as most studies used medical records. However, there was potential for bias due to a lack of clarity regarding ascertainment of all cardiovascular outcomes. Adequacy of reporting and duration of follow-up were rated as good for around half of the studies.

#### Incidence

There was also high risk of bias among many of the included studies that reported CVD incidence (S17 File); ascertainment of CVD was good in only 7 cohort studies, and there was a lack of clarity regarding presence of CVD at baseline for 32 studies. However, 26 incidence studies adjusted for at least 1 confounder, and adequacy of reporting and duration of follow-up were rated as good for over half of the studies. Overall, 11 incidence studies [58–68] were assessed to have low risk of bias.

### Meta-analysis

#### Mortality

Altogether, 59 studies contributed HRs, rate ratios, ORs, or SMRs of cardiovascular mortality in people with SMI for meta-analysis. Some studies contributed more than 1 result, e.g., different SMIs, multiple outcomes, or separate male and female results. The number of included participants was not reported for some studies; however, the total among those that did report this information was 1,841,356 cases and 29,321,409 controls. The risk of CVD mortality for people with SMI was higher than for control participants, for each type of SMI, each CVD outcome, and each type of effect, except pooled HRs/rate ratios for all circulatory disease mortality in BD and SMRs for CVA in mixed SMI. Pooled estimated risks appeared larger for schizophrenia than BD. Pooled results for SMI/CVD combinations with more than 1 study ranged from SMR = 1.55 (95% CI: 1.33 to 1.81, *p* < 0.001) for CVA in people with BD to HR/rate ratio = 2.40 (95% CI: 2.25 to 2.55, *p* < 0.001) for CVA in schizophrenia (Table 1).

**Table 1:** Pooled relative risks of cardiovascular mortality by SMI and cardiovascular outcome.

SMI	Outcome	Measure	Studies/Results	Pooled effect (95% CI)	*p*-value	Prediction interval	I^2^	X^2^	df	*p*-value
SCZ	CVA	HR/rate ratio	3/3	**2.4** (2.25–2.55)	<0.001	1.6–3.6	0.0	0.5	2	0.785
SCZ	CVA	SMR	14/16	**1.93** (1.63–2.28)	<0.001	1.02–3.67	91.5	**176.3**	15	<0.001
SCZ	CHD	HR/rate ratio	7/10	**1.8** (1.44–2.24)	<0.001	0.79–4.09	98.9	**827.1**	9	<0.001
SCZ	CHD	SMR	14/16	**1.94** (1.58–2.37)	<0.001	0.81–4.62	98.0	**733.8**	15	<0.001
SCZ	CVD	HR/rate ratio	10/12	**1.91** (1.52–2.41)	<0.001	0.79–4.66	98.9	**979.0**	11	<0.001
SCZ	CVD	OR	1/2	**2.55** (1.74–3.74)	<0.001	.	91.8	**12.1**	1	<0.001
SCZ	CVD	SMR	18/20	**1.96** (1.61–2.39)	<0.001	0.77–4.97	99.2	**2414.0**	19	<0.001
SCZ	HF	HR/rate ratio	1/1	**3.25** (2.94–3.6)	<0.001	.	.	0.0	0	.
BD	CVA	HR/rate ratio	2/2	**2.01** (1.81–2.22)	<0.001	.	0.0	0.5	1	0.498
BD	CVA	SMR	5/7	**1.55** (1.33–1.81)	<0.001	0.99–2.44	74.0	**23.1**	6	0.001
BD	CHD	HR/rate ratio	5/7	**1.61** (1.34–1.94)	<0.001	0.93–2.8	90.2	**61.2**	6	<0.001
BD	CHD	SMR	5/7	**1.67** (1.54–1.82)	<0.001	1.33–2.1	57.7	**14.2**	6	0.028
BD	CVD	HR/rate ratio	2/2	1.52 (0.84–2.75)	0.162	.	94.6	**18.5**	1	<0.001
BD	CVD	OR	1/2	**1.83** (1.57–2.14)	<0.001	.	65.2	2.9	1	0.090
BD	CVD	SMR	12/14	**1.65** (1.53–1.77)	<0.001	1.29–2.1	80.4	**66.4**	13	<0.001
SMI	CVA	SMR	1/1	0.8 (0.16–4.08)	0.788	.	.	0.0	0	.
SMI	CHD	SMR	1/1	**3.7** (2.47–5.55)	<0.001	.	.	0.0	0	.
SMI	CVD	HR/rate ratio	1/1	**5.41** (2.4–12.2)	<0.001	.	.	0.0	0	.
SMI	CVD	SMR	4/5	**2.3** (1.63–3.25)	<0.001	0.63–8.4	92.9	**56.3**	4	<0.001

“.”*—*not calculable (insufficient observations).

Results where 95% CIs exclude the null highlighted in bold.

BD, bipolar disorder; CHD, coronary heart disease; CI, confidence interval; CVA, cerebrovascular accident; CVD, all circulatory disease; HF, heart failure; HR, hazard ratio; OR, odds ratio; SCZ, schizophrenia; SMI, mixed schizophrenia and bipolar disorder; SMR, standardised mortality ratio.

**Fig 2:**
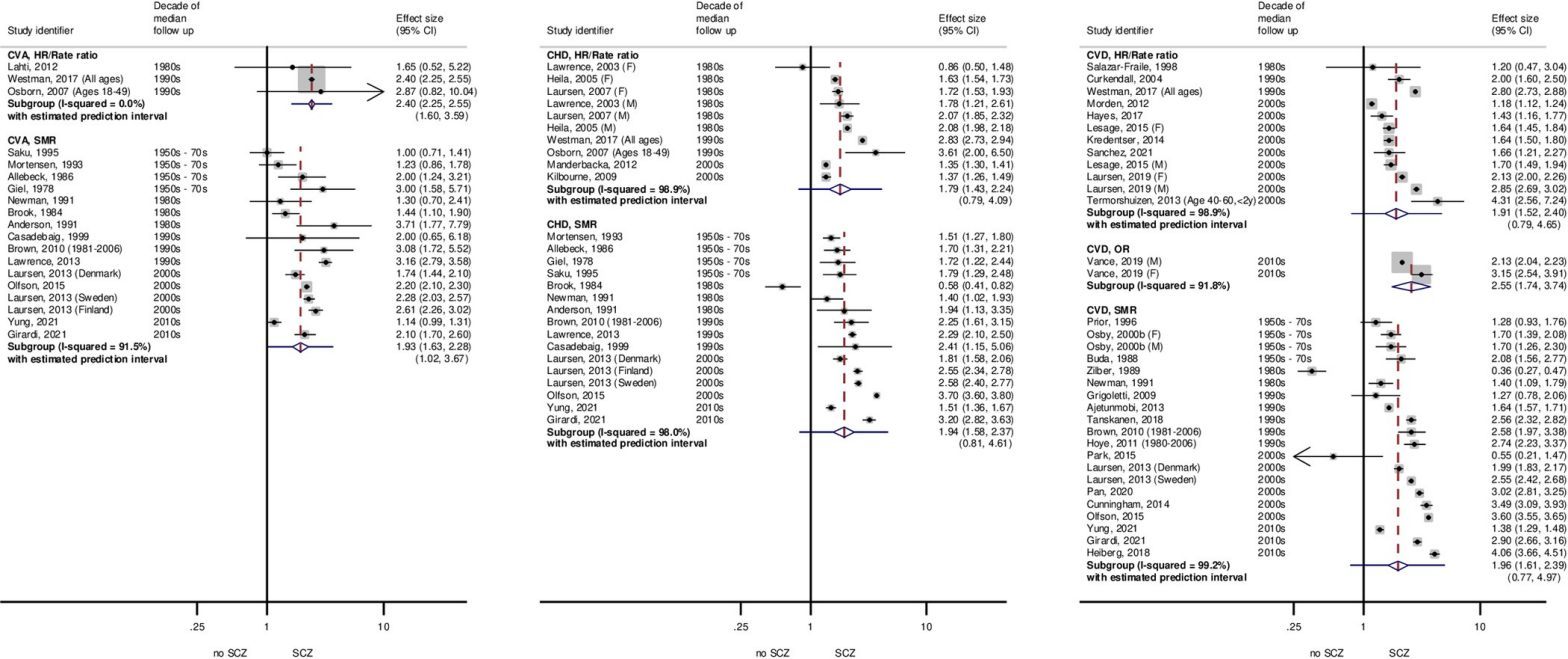
Forest plots showing relative risk of CVD mortality in those with versus without schizophrenia, studies included in meta-analysis. Weights are from random effects model. Dashed lines show pooled estimated risks within subgroups. Studies were allocated to decades according to the median year of follow-up. CHD, coronary heart disease; CVA, cerebrovascular accident; CVD, all circulatory disease; F, female; HR, hazard ratio; M, male; OR, odds ratio; SCZ, schizophrenia; SMR, standardised mortality ratio.

**Fig 3:**
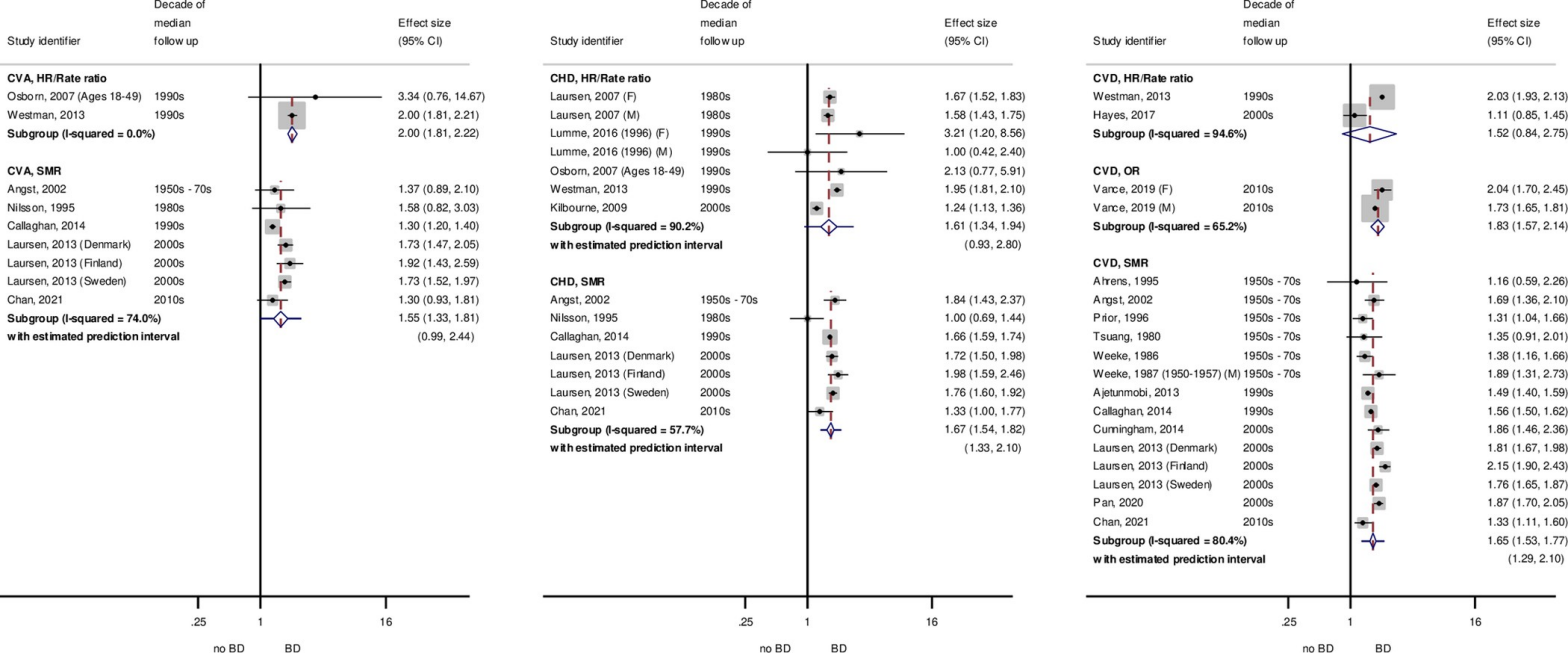
Forest plots showing relative risk of CVD mortality in those with versus without BD, studies included in meta-analysis. Weights are from random effects model. Dashed lines show pooled estimated risks within subgroups. Studies were allocated to decades according to the median year of follow-up. BD, bipolar disorder; CHD, coronary heart disease; CVA, cerebrovascular accident; CVD, all circulatory disease; F, female; HR, hazard ratio; M, male; OR, odds ratio; SMR, standardised mortality ratio.

We found significant heterogeneity affecting all subgroups (*p* < 0.05 and I^2^ > 50%), except for CVA pooled HRs/rate ratios in both schizophrenia and BD and ORs for CVD in BD (the latter based on a single study). Prediction intervals may be informative in describing the likely range of future studies [54]. For schizophrenia, 95% prediction intervals did not overlap unity for CVA, and for BD, they did not overlap unity for CHD and all circulatory disease SMRs. Thus, for these combinations of SMI and CVD mortality, we would expect a future study to report elevated mortality ratios. Figs 2 and 3 show forest plots of studies reporting CVD mortality outcomes for schizophrenia and BD that were included in meta-analyses.

#### Incidence

Results from 28 studies that contributed data on risk of cardiovascular incidence in people with SMI compared with control participants were used in meta-analysis. Some studies contributed more than 1 result. The number of included participants for studies that did report this information was 401,090 cases and 14,372,146 controls. Pooled results for schizophrenia and BD, for incidence of CVA, CHD, major CVD events, and heart failure, are shown in Table 2. As for mortality, most pooled results involving more than 1 study indicated an elevated risk of CVD incidence for participants with SMI compared with control participants, with the notable exception of CHD for people with schizophrenia. Pooled ORs indicated a reduced risk of CHD incidence (OR: 0.76, 95% CI: 0.61 to 0.96, *p* = 0.077), while RRs and pooled HRs/rate ratios included the null. Risks of CVA, total cardiovascular events, and heart failure for both schizophrenia and BD and risk of CHD for BD were in a positive direction for all except 2 studies, which were not significantly different from unity [69,70]. In subgroups containing more than 1 study, there was significant heterogeneity in most groups (*p* < 0.1) and I^2^ > 50%, except for ORs and RRs for CVA and ORs for CHD and HF in schizophrenia, CVA RRs, and CVD HR/rate ratios in BD. Prediction intervals for all subgroups included unity except for HRs/rate ratios for CVA in BD. Studies reporting incidence outcomes are shown in Figs A and B in S18 File.

**Table 2:** Pooled relative risks of cardiovascular incidence by SMI and cardiovascular outcome.

SMI	Outcome	Measure	Studies/Results	Pooled effect (95% CI)	*p*-value	Prediction interval	I^2^	X^2^	df	*p*-value
SCZ	CVA	HR/rate ratio	4/5	**1.66** (1.36–2.02)	<0.001	0.82–3.34	94.6	**74.2**	4	<0.001
SCZ	CVA	OR	2/2	**1.88** (1.31–2.7)	0.001	.	0.0	0.3	1	0.586
SCZ	CVA	RR	3/3	**1.4** (1.31–1.49)	<0.001	0.94–2.09	0.0	0.3	2	0.861
SCZ	CHD	HR/rate ratio	8/10	1.15 (0.99–1.35)	0.077	0.65–2.04	97.8	**415**	9	<0.001
SCZ	CHD	OR	2/2	**0.76** (0.61–0.96)	0.022	.	0.0	0.4	1	0.515
SCZ	CHD	RR	3/3	0.91 (0.72–1.14)	0.403	0.07–11.47	73.7	**7.6**	2	0.022
SCZ	CHD	SIR	1/2	**1.48** (1.3–1.69)	<0.001	.	85.0	**6.7**	1	0.01
SCZ	CVD	HR/rate ratio	3/3	**1.25** (1.04–1.51)	0.016	0.14–11.5	83.3	**12**	2	0.003
SCZ	CVD	OR	1/2	**1.8** (1.24–2.62)	0.002	.	95.3	**21.4**	1	<0.001
SCZ	HF	HR/rate ratio	1/1	**3.82** (3.1–4.71)	<0.001	.	.	0.0	0	.
SCZ	HF	OR	2/2	**1.79** (1.04–3.07)	0.034	.	57.3	2.3	1	0.126
SCZ	HF	RR	2/2	**1.72** (1.08–2.75)	0.023	.	75.1	**4.0**	1	0.045
BD	CVA	HR/rate ratio	4/5	**1.6** (1.41–1.81)	<0.001	1.07–2.4	70.6	**13.6**	4	0.009
BD	CVA	OR	1/1	**3.39** (1.91–6.01)	<0.001	.	0.0	0.0	0	.
BD	CVA	RR	2/2	**1.31** (1.18–1.45)	<0.001	.	0.0	0.0	1	0.882
BD	CHD	HR/rate ratio	5/6	**1.47** (1.16–1.87)	0.002	0.63–3.43	94.6	**92.2**	5	<0.001
BD	CHD	OR	1/1	**2.68** (1.51–4.76)	0.001	.	.	0.0	0	.
BD	CHD	RR	1/1	0.97 (0.5–1.88)	0.928	.	.	0.0	0	.
BD	CVD	HR/rate ratio	2/2	**1.47** (1.32–1.63)	<0.001	.	0.0	0.1	1	0.807
BD	CVD	OR	2/3	**1.88** (1.34–2.66)	<0.001	0.03–134.7	95.4	**43.7**	2	<0.001
BD	HF	HR/rate ratio	1/1	**3.3** (2.27–4.79)	<0.001	.	.	0.0	0	.
BD	HF	RR	1/1	1.73 (0.88–3.4)	0.111	.	.	0.0	0	.

“.”—not calculable (insufficient observations).

Results where 95% CIs exclude the null highlighted in bold.

BD, bipolar disorder; CHD, coronary heart disease; CI, confidence interval; CVA, cerebrovascular accident; CVD, major cardiovascular events; HF, heart failure; HR, hazard ratio; OR, odds ratio; RR, risk ratio; SCZ, schizophrenia; SIR, standardised incidence ratio.

### Temporal trends

#### Mortality

Results from meta-regression (Table 3) showed that for schizophrenia, SMRs for cerebrovascular mortality in the 1990s were 2-fold higher than in the 1970s and earlier (SMR: 1.96, 95% CI: 1.02 to 3.78, *p* = 0.044). The earliest decade for which results were available was chosen as the reference group. Pooled HR/rate ratios for CHD were 60% higher in the 1990s compared with the 1980s (HR/rate ratio: 1.61 [95% CI: 1.14 to 2.28, *p* = 0.014]). However, the estimated risks for the strength of association between SMI and CVD was lower in the 2000s compared with the 1980s (HR/rate ratio: 0.75 [95% CI: 0.56 to 1.00, *p* = 0.052]). BD studies in the 2000s reported SMRs for all circulatory disease higher than those in the 1950s to 1970s (SMR: 1.27, 95% CI: 1.09 to 1.47, *p* = 0.006).

Heterogeneity between studies was reduced by the inclusion of decade in the case of schizophrenia and CVA (I^2^ = 91.5%, residual I^2^ = 79.0%), with decade accounting for a substantial amount of the between study variation (adjusted R^2^ = 37.5%). Similarly, for BD and all CVD mortality, decade reduced heterogeneity and accounted for considerable between-study variation (I^2^ = 80.4%, residual I^2^ = 34.3%, adjusted R^2^ = 88.9%). For schizophrenia and CHD, there was still considerable heterogeneity after the inclusion of decade (residual I^2^ = 96.6%, adjusted R^2^ = 85.7%), but the model *p*-value of 0.007 indicates some evidence for a relationship between decade and size of association between schizophrenia and CVD mortality.

**Fig 4:**
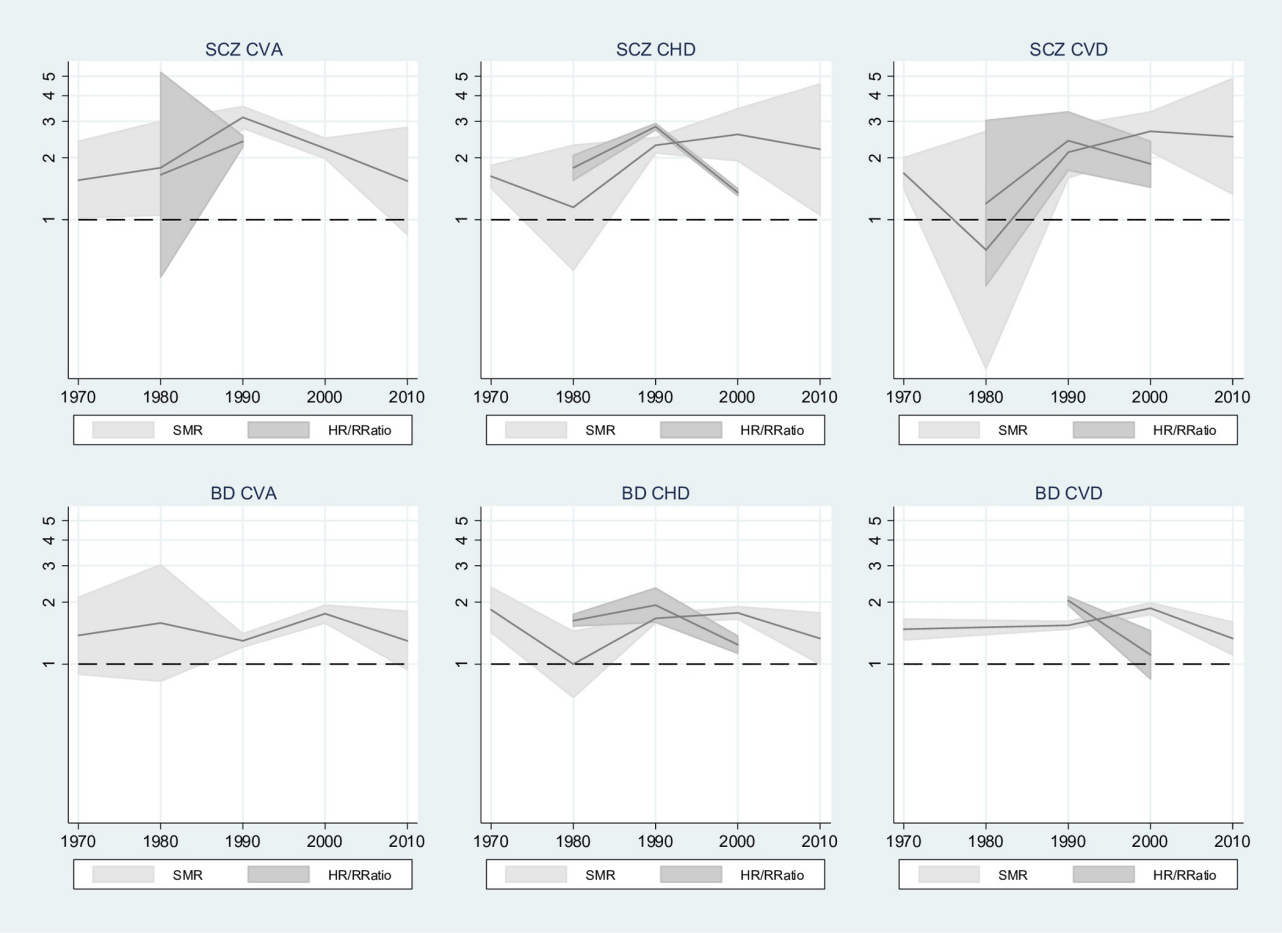
Trend in risk of CVD mortality for SMI compared with control participants, by median decade. BD, bipolar disorder; CHD, coronary heart disease; CI, confidence interval; CVA, cerebrovascular accident; CVD, all circulatory disease; HR, hazard ratio; RRatio, rate ratio; SMI, severe mental illness; SMR, standardised mortality ratio; SCZ, schizophrenia. The year 1970 includes studies with median between 1950 and 1970. y-Axes show estimated risks on log scale, and shaded areas show 95% CIs for risk estimates.

Fig 4 shows the trends in CVD mortality by SMI across decades. For schizophrenia and BD, there appeared to be stronger associations between SMI and both CVD and CHD mortality in studies reporting SMRs occurring during the 1990s and 2000s, compared with earlier decades, suggesting that, on this measure, the gap between people with SMI and the general population may be widening. In the 2010s, SMRs were reduced slightly, but CIs were wider. Pooled HRs and rate ratios appeared to peak in the 1990s. CVA SMRs peaked in the 1990s for schizophrenia and in the 2000s for BD. A summary forest plot of meta-analysis results by SMI, outcome, and decade with the inclusion of prediction intervals is shown in Fig A in S19 File. Most prediction intervals were wide and included the null, indicating that any future study could potentially show a wide range of potential effects including decreased risk of CVD mortality for SMI compared with control participants (see also Table A in S19 File).

For most SMI/CVD combinations, increasing the granularity by splitting time into 5-year periods for meta-regression did not add any further information as there were too few studies in each group. For those groups that did have sufficient observations, CIs were wide, and most results indicated no increased risk for any 5-year period compared with the reference period. Only for CVA in schizophrenia did the 5-year analysis show higher SMRs for 1995 to 1999 compared with 1950 to 1969 (see Table C in S19 File). Graphs showing the trends in CVD-related mortality for SMI compared with control participants, by 5-year periods, are presented in Fig C in S19 File.

#### Incidence

**Fig 5:**
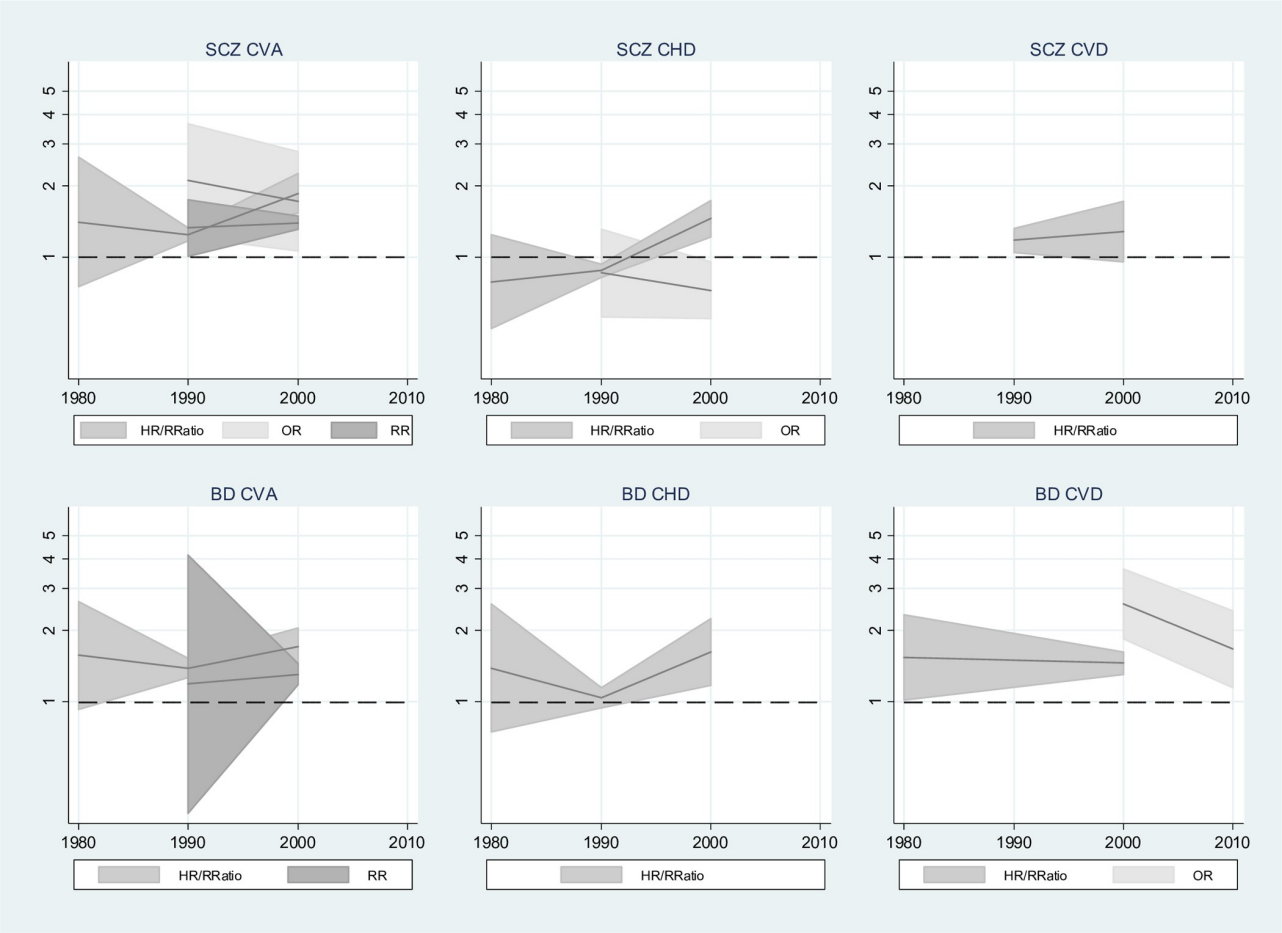
Trend in risk of CVD incidence for SMI compared with control participants, by median decade. BD, bipolar disorder; CHD, coronary heart disease; CI, confidence interval; CVA, cerebrovascular accident; CVD, major cardiovascular events; HR, hazard ratio; OR, odds ratio; RR, risk ratio; RRatio, rate ratio; SCZ, schizophrenia; SMI, severe mental illness. y-Axes show estimated risks on log scale, and shaded areas show 95% CIs for risk estimates.

Meta-regression was not performed on cardiovascular incidence data as there were insufficient results in each subgroup. Fig 5 shows the trends in CVD incidence by SMI across decades. For schizophrenia and BD, RRs and pooled HRs and rate ratios increased during the 2000s. No studies reported results on these measures beyond this decade. These measures appear to show a widening gap between people with SMI and the general population. ORs, however, showed decreased effects between the 1990s and later years.

A summary forest plot of meta-analysis results by SMI, CVD incidence outcome, and decade with prediction intervals is shown in Fig B in S19 File. All prediction intervals that were able to be calculated included the null, indicating that any future study could potentially show a decreased risk of CVD incidence for SMI compared with the general population. Tabulated results and trends by 5-year intervals are in Table B and Fig D in S19 File.

**Table 3:** Results of meta-regressions: estimates of increased risk relative to the reference category for study-level explanatory variables; exponentiated regression coefficients (95% CIs), *p*-values.

Explanatory variable (if no. of studies/results ≥10)			Schizophrenia			BD
			CVA	CHD	CVD	CVD
Decade	SMR	1950s-70s (ref) 1980s 1990s 2000s 2010s	1 1.14 (0.59–2.20), 0.673 **1.96** (1.02–3.78), 0.044 1.45 (0.85–2.46), 0.153 1.01 (0.54–1.89), 0.978	1 0.67 (0.36–1.22), 0.169 1.38 (0.75–2.52), 0.267 1.55 (0.92–2.59), 0.089 1.31 (0.70–2.45), 0.358	1 0.43 (0.17–1.06), 0.063 1.26 (0.63–2.53), 0.489 1.51 (0.77–2.96), 0.214 1.52 (0.70–3.29), 0.271	1 – 1.04 (0.89–1.21), 0.579 **1.27** (1.09–1.47), 0.006 0.91 (0.69–1.18), 0.425
	*Model p-value*, *residual I*^*2*^, *adjusted R*^*2*^		*0*.*193*, *79*.*0%*, *37*.*5%*	*0*.*073*, *96*.*6%*, *38*.*3%*	*0*.*059*, *98*.*3%*, *39*.*5%*	*0*.*003*, *34*.*3%*, *88*.*9%*
	HR/rate ratio	1950s–70s 1980s (ref) 1990s 2000s 2010s	Insufficient observations	- 1 **1.61** (1.14–2.28), 0.014 0.75 (0.56–1.00), 0.052 -	- 1 1.99 (0.46–8.60), 0.314 1.54 (0.39–6.14), 0.500 -	Insufficient observations
	*Model p-value*, *residual I*^*2*^, *adjusted R*^*2*^			*0*.*007*, *87*.*0%*, *85*.*7%*	*0*.*487*, *98*.*3%*, *2*.*1%*	
Setting	SMR	Community (ref) Community and inpatient Inpatient	1 0.62 (0.21–1.81), 0.355 0.61 (0.21–1.74), 0.325	1 1.02 (0.37–2.80), 0.969 0.76 (0.28–2.03), 0.552	1 1.19 (0.43–3.29), 0.728 0.93 (0.33–2.58), 0.887	1 0.96 (0.77–1.19), 0.672 -
	*Model p-value*, *residual I*^*2*^, *adjusted R*^*2*^		*0*.*603*, *92*.*5%*, *-8*.*8%*	*0*.*412*, *97*.*4%*, *-0*.*9%*	*0*.*699*, *98*.*6%*, *-5*.*7%*	*0*.*672*, *81*.*5%*, *-11*.*1%*
	HR/rate ratio	Community (ref) Community and inpatient Inpatient	Insufficient observations	1 0.67 (0.26–1.76), 0.359 0.98 (0.47–2.05), 0.954	1 1.25 (0.79–1.98), 0.292 1.71 (0.79–3.68), 0.150	Insufficient observations
	*Model p-value*, *residual I*^*2*^, *adjusted R*^*2*^			*0*.*524*, *99*.*1%*, *-18*.*7%*	*0*.*279*, *98*.*2%*, *6*.*1%*	
Region	SMR	UK (ref) Far East North America Oceania Other EU/Israel Scandinavia	1 **0.32** (0.15–0.68), 0.007 0.58 (0.27–1.24), 0.140 0.95 (0.42–2.13), 0.888 0.58 (0.28–1.19), 0.122 0.60 (0.30–1.17), 0.118	1 0.78 (0.25–2.38), 0.626 1.11 (0.36–3.39), 0.836 1.09 (0.29–4.11), 0.887 0.78 (0.29–2.13), 0.600 0.95 (0.37–2.43), 0.897	1 0.88 (0.31–2.46), 0.787 1.26 (0.46–3.42), 0.634 1.98 (0.49–8.07), 0.313 0.63 (0.23–1.75), 0.351 1.35 (0.58–3.15), 0.457	1 1.13 (0.79–1.64), 0.451 1.06 (0.72–1.56), 0.740 1.31 (0.79–2.16), 0.255 1.11 (0.70–1.75), 0.620 1.26 (0.92–1.71), 0.131
	*Model p-value*, *residual I*^*2*^, *adjusted R*^*2*^		*0*.*034*, *68*.*5%*, *59*.*9%*	*0*.*942*, *94*.*4%*, *-37*.*6%*	*0*.*417*, *97*.*9%*, *0*.*04%*	*0*.*586*, *73*.*2%*, *-17*.*4%*
	HR/rate ratio	UK (ref) Far East North America Oceania Other EU/Israel Scandinavia	Insufficient observations	1 - 0.38 (0.11–1.30), 0.103 0.36 (0.11–1.21), 0.085 - 0.52 (0.18–1.51), 0.187	1 - 1.11 (0.56–2.21), 0.729 - 1.51 (0.67–3.42), 0.273 1.80 (0.88–3.67), 0.094	Insufficient observations
	*Model p-value*, *residual I*^*2*^, *adjusted R*^*2*^			*0*.*247*, *99*.*2%*, *16*.*6%*	*0*.*109*, *94*.*8%*, *53*.*8%*	
*Random effects meta-analysis I* ^*2*^		*SMR*	*91*.*5%*	*98*.*0%*	*99*.*2%*	*80*.*4%*
		*HR/rate ratio*		*98*.*9%*	*98*.*9%*	

BD, bipolar disorder; CI, confidence interval; CHD, coronary heart disease; CVA, cerebrovascular accident; CVD, all circulatory disease; HR, hazard ratio; North America, USA and Canada; Other EU, Western Europe excluding UK and Scandinavia; ref, reference group; SMR, standardised mortality ratio.

Results where 95% CIs exclude the null highlighted in bold. Models adjusted for age and sex and minimum number of additional factors.

### Subgroup analysis

#### Mortality

**Age group**. Estimated risks for younger age groups were larger than for older ages in the same study or in the equivalent SMI/outcome/effect type subgroup (Fig A in S20 File).

**Sex**. CIs of pooled estimates for males and females overlapped for all combinations of SMI and CVD mortality (Figs B, C and D in S20 File), except for schizophrenia ORs for CVD mortality and pooled HRs/rate ratios for heart failure mortality. Single studies contributed results to these 2 outcomes. There was a higher reported estimated risk for mortality from heart failure for males compared with females (males: rate ratio: 4.30, 95% CI: 3.71 to 4.98; females: rate ratio: 2.66, 95% CI: 2.32 to 3.06). However, the OR for circulatory mortality was higher for females than males (males: OR 2.13, 95% CI: 2.04 to 2.23; females: OR 3.15, 95% CI: 2.54 to 3.91).

**Location and setting**. Results of meta-regression showed no differences in CVD-related mortality for any region compared with the UK, except for studies reporting SMRs for the Far East, which had a smaller association between schizophrenia and CVD mortality: SMR: 0.32, 95% CI: 0.15 to 0.68, *p* = 0.007 (Table 3). However, there was still considerable residual heterogeneity between studies (residual I^2^ > 68.5%). The inclusion of region reduced I^2^ from 91.5% to 68.5%.

The association between SMI and CVD did not vary for different settings (inpatient, outpatient, and community).

**Other factors**. No further subgroup analyses by study quality, length of follow-up, or confounding factors were completed owing to lack of applicable studies. Only 3 mortality studies were judged to be of low risk of bias and studies either lacked reported rates for confounding factors in the SMI and control populations or varied in the confounders included.

#### Incidence

**Risk of bias.** Meta-analysis was conducted for the studies assessed to be at low risk of bias. Pooled relative risks for the low risk of bias studies presented a similar picture to results from studies included in the meta-analysis for most SMI/CVD subgroups (see Fig E in S20 File). One notable exception was incidence of CHD for schizophrenia. The pooled HR/rate ratio for the higher-quality schizophrenia studies was raised (HR/rate ratio: 1.35, 95% CI: 1.03 to 1.79, *p* = 0.032), whereas the pooled result for all included schizophrenia studies did not exclude the null (HR/rate ratio: 1.15, 95% CI: 0.9 to 1.35, *p* = 0.077).

### Sensitivity analysis

#### Excluded studies

A total of 18 mortality studies and 11 incidence studies were excluded from the meta-analysis because overlapping periods of exposure with another included study may have meant that the same participants were double counted if the studies were included. Sensitivity analyses explored the effect of replacing included studies with excluded ones. This made little difference to the findings for CVD mortality; however, some pooled estimated risks were increased (Table A in S21 File). Only one inclusion affected results, increasing the rate ratio of circulatory mortality for BD from HR/rate ratio: 1.52 (95% CI: 0.84 to 2.75) to 1.78 (95% CI: 1.28 to 2.47) when the included study [71] was replaced by an alternative that used the same exposed population [60]. Sensitivity analysis made little difference to the findings for BD and CVD incidence (Table B in S21 File). However, for schizophrenia, there were inconsistent results when substituting some of the included studies, particularly for CHD. The pooled HR/rate ratio for CHD was borderline for the studies included in the meta-analysis (HR/rate ratio: 1.15 (95% CI: 0.99 to 1.35). Sensitivity analysis widened the CIs around the null for some studies but increased the estimated risk away from the null for others.

There were 2 excluded studies of schizophrenia that we were not able to obtain full texts for, so could not confirm the methodology, but they showed similar results for incidence of CHD [72] and heart failure [73] to those presented in Table 1. Another study excluded from the review, because middle-income as well as high-income countries were included [74] reported similar results for incidence of stroke to those in Table 2.

#### Confounding factors (incidence)

Results adjusted for age and sex were extracted from incidence studies. A total of eight studies reported results adjusted by age and sex only. Moreover, 20 studies reported models adjusted for up to 18 additional factors, median 5 factors. Of these, 5 did not report results adjusted for age and sex alone. Meta-analysis using models adjusted for factors in addition to age and sex made little difference to the direction of results, although estimated risks were reduced for some subgroups (Table C in S21 File).

Seven incidence studies adjusted for smoking. Seven studies reported rates of antipsychotic drug use. Subgroup analysis by confounding factors was not possible, because there was too much variability in the confounders adjusted for.

#### Publication bias and small study effects

Small study effects were assessed visually by funnel plots (S22 and S23 Files). Mortality results for SMRs, ORs, HRs, and rate ratios were combined for visual assessment. There was some asymmetry in the funnel plots, particularly for CHD and CVD mortality in schizophrenia. Contour funnel plots for these 2 subgroups showed that some studies with nonsignificant results may be missing (white areas in the plots). Egger test was performed for subgroups with more than 10 studies (Table A in S22 File). There was evidence of significant biases for CHD mortality and CVD mortality in schizophrenia studies reporting SMRs. Applying random effects trim-and-fill procedures [53] added 5 unpublished studies reporting CHD mortality and 2 reporting all circulatory disease mortality for schizophrenia (Figs G and H in S22 File). The resulting pooled random effects estimate changed from SMR = 1.94 (95% CI: 1.58 to 2.37, *p* < 0.001) to SMR = 1.61 (95% CI: 1.11 to 2.33, *p* = 0.012) for CHD and from SMR = 1.96 (95% CI: 1.61 to 2.38, *p* < 0.001) to SMR = 1.77 (95% CI: 1.27 to 2.47, *p* = 0.001) for all circulatory disease.

Incidence results for HRs, rate ratios, ORs, RRs, and SIRs were combined due to small numbers of studies in each group. Egger test was performed for incidence of CVA and CHD in schizophrenia as only those subgroups had more than 10 studies (Table A in S23 File). Significant small study bias was not found when different SMIs and different outcomes were considered separately, although when results from all studies were considered together, publication bias was indicated (Egger test for bias: 1.94, 95% CI: 0.16 to 3.72, *p* = 0.034). However, applying trim and fill methodology did not add any additional unpublished studies.

## Discussion

This large-scale systematic review has been conducted in a methodologically robust way and has yielded up-to-date comprehensive evidence on the association between SMI- and CVD-related mortality and incidence. It has found that the CVD mortality rate for people with SMI is around twice that of the general population. People with schizophrenia are at greater risk than those with BD; however, the disparity exists across all types of SMI and both cerebrovascular and cardiac mortality. The review also found a consistent relationship between SMI and increased incidence of CVA, CVD events, and heart failure. The relationship between SMI and CHD was less clear; a positive association only became apparent for high-quality studies with low risk of bias.

Some evidence was found of changes in the risk of CVD over time, and results from some studies suggest that the gap between SMI and the general population appears to be widening. The size of the association between SMI and both CHD and all circulatory mortality increased until the 2000s, following this it appeared to decline. Estimated risks of the associations between schizophrenia and CVA mortality were also highest in the 1990s, whereas for BD, they were highest for CVA mortality in the 2000s.

Studies reporting incidence of CVD since the 1990s had larger associations on most measures between SMI and diagnosis of CVA and CHD compared with earlier studies. ORs appeared to show the reverse trend; however, only 5 studies contributed results that could be used in this trend analysis.

The apparent increases in CVD risk for people with SMI relative to control participants that we have found in recent decades are in marked contrast to the reducing rates of CVD experienced by the general population. Since 1990, there has been a steady decline in the rates of CVD mortality and incidence in the populations of high-income countries [17]. Most European countries have also seen a halving in overall population CHD mortality since the 1980s [75].

Increasing trends in CVD mortality for people with SMI relative to control participants have been noted by other authors [30,44,76,77]. An increase in CHD hospital admissions for people with schizophrenia compared with the general population since the 1990s has also been reported [44].

The increased relative risk of CVD diagnosis in more recent decades may be a result of disparity in smoking prevalence between people with SMI and the general population [78,79] or increased use of antipsychotics. The changes since the 1990s approximately coincide with the release of newer, second-generation antipsychotics (SGAs), which are known to have worse metabolic effects [80–82]. The use of these drugs has increased over the last 20 years for treatment of both schizophrenia and BD [83–86].

There was significant heterogeneity between studies, which was partly explained by decade of results and world region, the latter due to differences in CVA incidence between the Far East and the UK. Although some evidence of differences between decades and between regions were found, the large number of CIs calculated from multiple meta-regressions mean that findings should be interpreted cautiously. Some evidence of small study effects was found by the review, affecting the association between schizophrenia and CVD mortality. However, publication bias is unlikely to account for the increased risk of CVD mortality found in the review as the pooled effect remained elevated, even after application of the trim and fill method. Small study bias could only be assessed for incidence studies when both types of SMI exposure and all CVD outcomes were combined. Although significant bias was found, analysis found no additional missing studies. This analysis may be flawed as it was not possible to exclude double counting of populations at risk or of outcomes. Thus, results may not have been independent, and the random effects assumption may have been violated. However, the analysis is of value in demonstrating that the scale of any potential publication bias is likely to be small.

### Comparison with previous literature

#### Mortality

*Schizophrenia*

Cerebrovascular accident. In contrast to all circulatory disease and CHD, the pooled CVA mortality ratio for schizophrenia, SMR: 1.93 (95% CI: 1.63 to 2.28, *p* < 0.001), is in the opposite direction to that found by other authors [24,28]. It is unclear which studies were included in these reviews, but the reviews were relatively small: One review only included 3 studies (median SMR: 0.69, IQR: 0.61 to 1.30) [24], and the other reported an SMR based on only 490 deaths (SMR: 81, 95% CI: 73 to 87) [28].

The current review, however, included 14 studies in the pooled SMR; it was based on 2,800 deaths, and all studies showed a higher risk of CVA mortality for people with schizophrenia compared to the general population. Pooled HRs and rate ratios were also elevated and were not reduced by the substitution of excluded studies in sensitivity analyses.

Coronary heart disease. The pooled result for the 14 studies reporting CHD SMRs for schizophrenia, SMR: 1.94 (95% CI: 1.58–2.37, *p* < 0.001), was higher, but in the same direction as other smaller reviews [24,28]. Further, sensitivity analysis showed that substituting some of the excluded studies could change the pooled SMR and HR/rate ratio. Small study effects or publication bias could account for some of this discrepancy; an analysis accounting for small study bias reduced the estimated risk to SMR: 1.61 (95% CI: 1.11 to 2.33, *p* = 0.012).

All circulatory disease. The pooled results for circulatory mortality in schizophrenia, from 28 studies (HR/rate ratio: 1.91, 95% CI: 1.52 to 2.41, *p* < 0.001; SMR: 1.96, 95% CI: 1.61 to 2.39, *p* < 0.001), were slightly lower than, but in broad agreement with, those of a recent review based on 9 studies [5]. Sensitivity analysis found that rate ratios were increased by the inclusion of one study that had high HRs [87] that was also included in the previous review [5].

*Bipolar disorder*

All circulatory disease. The pooled SMR of 1.65, 95% CI: 1.53 to 1.77, *p* < 0.001 and combined HR and rate ratio of 1.52, 95% CI: 0.84 to 2.75, *p* = 0.162 for all circulatory mortality in BD are similar to ratios reported by other authors [5,27].

#### Incidence

*Schizophrenia*

Cerebrovascular accident. Previous reviews have also reported increased risks of stroke with schizophrenia and with bipolar disease [5,22,23,25].

Coronary heart disease. The current review found that people with schizophrenia are less likely to be diagnosed with CHD than the general population, in contrast to previous reviews [5,25]. The disparity may be explained by variation in included primary studies.

Heterogeneity in incidence of CHD could reflect differences in ascertainment. Five schizophrenia studies only included inpatient diagnosis of CHD [65,88–91], which could have missed both milder cases treated in the community and deaths from undiagnosed CHD outside of hospital.

*Bipolar disorder*

Coronary heart disease. The nonsignificant positive associations between CHD and BD were similar to those reported elsewhere [5,22].

*Schizophrenia and bipolar disorder*

All circulatory disease. The increased likelihood of total CVD events and SMI (both schizophrenia and BD) correspond with the findings of previous reviews [5,25].

#### Change over time

We have found evidence that the gap in CVD mortality between people with SMI and the general population appears to be widening. This finding supports the observations in some of the included studies that CVD mortality has increased in recent years for people with SMI relative to the general population [21,44,76,77]. This is likely to be due to a number of factors. In developed countries, adult smoking rates have fallen for the general population in recent decades: from average rates of 33.1% in 1980 to 23.5% in 2012 [92]. However, people with long-term mental health problems have not seen a similar decline [78,79]. SGA medication has been in use since the 1990s [81,82]. However, there is a possible trade-off in their use between the benefits of treating psychotic symptoms against the metabolic problems that can result which increase the risk of CVD. Use of some SGAs, especially clozapine and olanzapine, is likely to result in weight gain, particularly with long-term use [93]. Certain SGAs (particularly clozapine, olanzapine, and quetiapine) are associated with a higher risk of diabetes than first-generation antipsychotics (FGAs) [94]. There is also some evidence of elevated lipid levels with use of clozapine, olanzapine, and risperidone compared with FGAs [95]. Differential access to physical health treatments for people with SMI is also an issue. Rates of cardiac revascularisation procedures have increased over the last 20 years [96]. However, people with schizophrenia who have acute coronary syndrome are much less likely than others to receive this type of treatment [8].

### Strengths and limitations

#### Strengths

The strengths of this review are its size and scope. Both schizophrenia and BD and different cardiovascular diagnoses were included. A wide search strategy was used, not limited by language or date of publication. Studies reporting different measures of relative risk were included, and changes over time were investigated. Robust methodology including duplicate screening, and data extraction was used. Possible sources of heterogeneity between studies were investigated. Most studies had an average follow-up duration of at least 5 years, some considerably longer, and long enough for CVD outcomes to occur. The adequacy of reporting follow-up was generally good as many of the studies were carried out in Scandinavia, which has near complete recording of population health records.

#### Limitations

There are also several limitations. It was difficult to exclude studies that had overlapping populations. Although attempts were made to minimise this, it is possible that there may be some double counting of patient populations. This means that the CIs may be slightly narrower than they would have been if all duplicated patients had been excluded. Many of the studies were at high risk of bias as they drew a comparison cohort from general population rates. The majority of studies did not adjust for cardiovascular risk factors. Furthermore, it was not possible to use reported results that were adjusted for important confounders as there was inconsistency in the confounders reported.

Despite including conference abstracts, there was some evidence of small study bias among studies reporting CHD and circulatory mortality for schizophrenia. These were studies with a higher risk of bias that used general population mortality rates to report SMRs. However, even after adjustment for possible unpublished studies, the risk of excess mortality remained elevated.

Although the literature searches found many papers measuring the association between SMI and the risk of cardiovascular mortality, searching one database for the additional search term “cause of death” revealed some additional studies. Thus, a small number of relevant studies may have been missed by failing to include this term in all database searches. However, any effect on the results is likely to be small due to the large number of included studies.

SMRs were calculated from available information if not reported directly (S24 File). If only whole numbers of expected deaths were available, this may have led to rounding errors. This is unlikely to have had much effect on the results.

#### Change over time

Temporal changes were assessed using decade of median year of follow-up. By comparing estimates from different studies from different time periods, we are performing an ecological analysis. Studies could have had outcomes spanning multiple decades but would only have been counted against one. This methodology may have reduced the apparent difference in relative risk between decades highlighted in the review. More insight on temporal changes may be gained by considering large, long-term cohort studies, and the use of individual patient data.

#### SMI definitions

There was some inconsistency in definitions of SMI diagnosis, which could have accounted for some of the heterogeneity between studies. Most studies used DSM or ICD coding to define SMI, but some defined SMI less robustly as confirmed by psychiatrist or otherwise recorded in notes [77].

Around half the included studies reporting CVD outcomes for schizophrenia grouped schizoaffective disorder together with schizophrenia. Another study grouped schizoaffective disorder with BD [97]. Schizophrenia causes more significant disability than BD [98]; schizoaffective disorder has symptoms of both conditions, so its inclusion could have reduced the average severity of schizophrenia exposure or increased it for BD [99].

Some definitions of schizophrenia included schizotypal disorder [89,100–106] (a type of nonpsychotic personality disorder below the clinical threshold for schizophrenia) [36]. Others did not specify either the diagnostic system or the codes used [45,107–113], so severity was not able to be determined. The severity of schizophrenia cases could also have been reduced by the exclusion of schizophrenia with substance use disorder [101].

Although studies that included psychotic depression were eligible for inclusion, other forms of major depressive disorder were not, due to the difficulty in distinguishing major depression from other types and severity of depression.

Studies where all participants were treated with lithium included those diagnosed with BD and schizoaffective disorder. However, unipolar depression and other SMI diagnoses that did not meet inclusion criteria were also present [97,114,115]. It was not possible to exclude these people, so some misclassified patients were included, although numbers were small. Categorisation of SMI in other studies may also be subject to misclassification due to variation in psychiatric diagnostic labelling [116].

#### CVD ascertainment

Most studies did not demonstrate adequately that CVD was not present at the start of the study. Although studies were selected such that SMI was diagnosed before cardiovascular death, presence of diagnosed or undiagnosed CVD prior to death was unknown for most studies. In incidence studies, although it is likely that SMI diagnosis was made at a younger age than incidence of CVD, it was not possible to be confident that CVD did occur after the start of SMI diagnosis.

#### Age

Studies were included regardless of the participants’ age at cardiovascular outcome, which was not known for most studies. However, larger associations between SMI and CVD were noted among mortality studies that did report relative risks for younger age groups. This could be due to a healthy survivor effect: Those with less SMI are more likely to survive to older ages.

#### Confounding factors

Not many mortality studies adjusted for confounding factors, other than age and sex, so exploration of the effects of important covariates, such as smoking, ethnicity, deprivation, and other cardiovascular risk factors was not possible. However, some studies noted that antipsychotics were associated with higher risks of cardiovascular mortality [87,117,118]. More research is needed to assess the effects of confounders on CVD mortality.

Most of the incidence studies did not adjust for important confounders with inconsistency in the confounders reported for different studies. Using results that were adjusted for confounders made little difference to the results, but it is likely that adjusting for factors such as smoking, comorbidities, obesity, or antipsychotic use may explain the increased risks.

#### Setting

Included studies were generally representative of persons with SMI. Those that included only inpatients were likely to have missed less severe cases, potentially overestimating risks, although no significant heterogeneity due to setting was detected. Community settings were underrepresented in the review: Many of the recruited exposed populations were inpatients. Consequently, the review includes an overrepresentation of participants with more severe symptoms of exposure and outcome. A lack of patients with relatively mild symptoms from primary care settings may explain some of the apparent underdiagnosis of CHD seen in the review.

#### Risk of bias

There was a risk of selection or information bias affecting many of the included studies. The risk of CVA diagnosis and total CVD events found for all studies remained elevated among the high-quality studies. However, risk of CHD diagnosis was elevated only for the high-quality studies.

#### Reporting by sex

Results for all persons were included where possible, but for some studies, data were only available separately for males and females. In these cases, results from each sex were included separately in the meta-analyses. However, results for males and females within the same study are not independent, so the random effects assumption of the meta-analysis could be violated. In practice, this is unlikely to have much effect on the conclusions.

## Conclusions

This systematic review and meta-analysis found that people with SMI are at increased risk of mortality from CVD compared with the general population and that the risk may be increasing over time. It also found that the risk of diagnosis with stroke, heart failure, and major cardiovascular events was elevated for people with SMI compared with control participants.

It was not possible to explore the effects of confounders, such as smoking and obesity, so at least some of the excess risk may be attributable to these residual factors. There was considerable heterogeneity between studies, partly explained by decade of results, world region, and age group. Prediction intervals included unity for some combinations of SMI and CVD mortality; thus, a future study may not find an increased risk of CVD mortality for people with SMI. However, this review included results from 82 studies, only 7 of which reported any estimated risks smaller than one. Any future study is unlikely to materially change the review findings.

Deaths from CVD make a large contribution to the inequalities in life expectancy experienced by those with SMI [6]. This result highlights the need for targeted interventions to improve the CVD health of people with SMI. A better mechanistic understanding of the reasons for the higher mortality rates would enable identification of where inequalities could be reduced. More research is needed to explore the role of smoking, obesity, antipsychotic medication, and access as well as adherence to CVD treatments for the excess cardiovascular mortality.

More research is needed to understand the reasons for the higher morbidity risk and to assess why it may have been worsening in recent decades. Such research should include the investigation of the relationship to cardiovascular risk factors, such as obesity, diabetes, blood pressure, smoking, and antipsychotics, and the role of access and adherence to treatments for these risk factors. Cohort studies accounting for these, and other important confounders, could help further investigate these factors.

### Author contributions

AML conducted the literature searches, carried out the analysis, and drafted the review. TM, HMP, and RR critically appraised and provided guidance during the development of the protocol and the review process. TM and HMP edited and redrafted the manuscript. AML, EP, HMP, TM, MO, MR, NT, TSA, GM, AL, and KA screened titles and abstracts or reviewed full texts. AML and EP conducted data extraction and risk of bias assessment. MJP provided advice on statistical analysis. CUC and MS contributed data from a previous review and the data extraction form. All authors read, edited, and approved the final version of the final manuscript.

## Supporting information

S1 File PRISMA Checklist. PRISMA, Preferred Reporting Items for Systematic Review and Meta-Analyses.
(DOCX)

S2 File PRISMA Abstract Checklist. PRISMA, Preferred Reporting Items for Systematic Review and Meta-Analyses.
(DOCX)

S3 File Deviations from protocol. (DOCX)

S4 File Inclusion and exclusion criteria. (DOCX)

S5 File Search strategies by database. (DOCX)

S6 File Data extraction form. (DOCX)

S7 File Risk of bias forms for mortality and incidence studies. Table A: Risk of bias form for cohort studies. Table B: Risk of bias form for case–control studies.
(DOCX)

S8 File Decision rules for overlapping studies. (DOCX)

S9 File List of included mortality studies. (DOCX)

S10 File List of included incidence studies. (DOCX)

S11 File List of excluded studies. (DOCX)

S12 File Characteristics of included mortality studies. (DOCX)

S13 File Characteristics of included incidence studies. (DOCX)

S14 File Forest plots reporting cardiovascular mortality outcomes, across decades. Fig A: Schizophrenia, mortality from CVA, SMRs. Fig B: Schizophrenia, mortality from CHD, SMRs. Fig C: Schizophrenia, mortality from all circulatory disease, SMRs. Fig D: BD, mortality from CVA, SMRs. Fig E: BD, mortality from CHD, SMRs. Fig F: BD, mortality from all circulatory disease, SMRs. Fig G: Schizophrenia, mortality from CVA, HRs, rate ratios and ORs. Fig H: Schizophrenia, mortality from CHD, HRs, rate ratios and ORs. Fig I: Schizophrenia, mortality from all circulatory disease, HRs, rate ratios and ORs. Fig J: Schizophrenia, mortality from heart failure, HRs, rate ratios and ORs. Fig K: BD, mortality from CVA, HRs, rate ratios and ORs. Fig L: BD, mortality from CHD, HRs, rate ratios and ORs. Fig M: BD, mortality from all circulatory disease, HRs, rate ratios and ORs. Fig N: Mixed SMI, mortality from CVDs, HRs, rate ratios, ORs, SMRs. BD, bipolar disorder; CHD, coronary heart disease; CVA, cerebrovascular accident; HR, hazard ratio; OR, odds ratio; SMR, standardised mortality ratio.
(PDF)

S15 File Forest plots reporting cardiovascular incidence outcomes, across decades. Fig A: Schizophrenia, incidence of CVA, HRs, rate ratios and ORs. Fig B: Schizophrenia, incidence of CHD, HRs, rate ratios and ORs. Fig C: Schizophrenia, incidence of major cardiovascular events, HRs, rate ratios and ORs. Fig D: Schizophrenia, incidence of heart failure, HRs, rate ratios and odds ratios. Fig E: BD, incidence of CVA, HRs, rate ratios, and ORs. Fig F: BD, incidence of CHD, HRs, rate ratios and ORs. Fig G: BD, incidence of major cardiovascular events, HRs, rate ratios and ORs. Fig H: BD, incidence of heart failure, HRs, rate ratios and ORs. BD, bipolar disorder; CHD, coronary heart disease; CVA, cerebrovascular accident; HR, hazard ratio; OR, odds ratio.
(PDF)

S16 File Risk of bias assessment for included mortality studies. Table A: Risk of bias assessment of studies reporting cardiovascular mortality outcomes, cohort studies.
(DOCX)

S17 File Risk of bias assessment for included incidence studies. Table A: Risk of bias assessment of studies reporting cardiovascular incidence outcomes, cohort studies. Table B: Risk of bias assessment of studies reporting cardiovascular incidence outcomes, case**–**control studies.
(DOCX)

S18 File Meta-analysis of CVD incidence outcomes. Fig A: Forest plots showing relative risk of CVD incidence in those with versus without schizophrenia, studies included in meta-analysis. Fig B: Forest plots showing relative risk of CVD incidence in those with versus without BD, studies included in meta-analysis. BD, bipolar disorder; CVD, cardiovascular disease.
(PDF)

S19 File Analysis of temporal trends. Fig A: Forest plot showing pooled estimates of cardiovascular mortality ratios by SMI, outcome and decade. Fig B: Forest plot showing pooled estimates of cardiovascular incidence ratios by SMI, outcome and decade. Table A: Meta-analysis: pooled results by SMI and cardiovascular mortality. Table B: Meta-analysis: pooled results by SMI and cardiovascular incidence. Table C: Results of meta-regressions: estimates of increased effect size relative to the reference category by median 5-year calendar period of outcome, exponentiated regression coefficients (95% CIs,) *p*-values. Fig C: Trend in risk of CVD mortality for SMI compared with controls, by median 5-year calendar period of outcome. Fig D: Trend in risk of CVD incidence for SMI compared with controls, by median 5-year calendar period of outcome. CI, confidence interval; CVD, cardiovascular disease; SMI, severe mental illness.
(PDF)

S20 File Subgroup analysis. Fig A: Forest plots showing studies reporting CVD mortality risk for SMI versus controls for (a) age groups ≤60; (b) older age groups or all ages. Fig B: Forest plots showing relative risk of CVD mortality in those with versus without schizophrenia, studies included in meta-analysis for (a) males; (b) females. Fig C: Forest plots showing relative risk of CVD mortality in those with versus without BD, studies included in meta-analysis for (a) males; (b) females. Fig D: Forest plots showing relative risk of CVD mortality in those with versus without mixed SMI, studies included in meta-analysis for (a) males; (b) females. Fig E: Forest plots of CVD incidence for (a) studies with low risk of bias, (b) all studies included in meta-analysis, schizophrenia and BD. BD, bipolar disorder; CVD, cardiovascular disease; SMI, severe mental illness.
(PDF)

S21 File Sensitivity analysis. Table A: Sensitivity analyses showing effect of replacing included with excluded results, studies reporting mortality outcomes. Table B: Sensitivity analyses showing effect of replacing included with excluded results, studies reporting incidence outcomes. Table C: Pooled relative risks of cardiovascular incidence by SMI and CVD outcome, comparison of minimally adjusted, and fully adjusted models reported by included studies. CVD, cardiovascular disease; SMI, severe mental illness.
(DOCX)

S22 File Assessment of publication bias: mortality studies. Fig A: Funnel plots for visual assessment of publication bias for studies reporting relative risk of CVD mortality for schizophrenia compared with controls, CVA. Fig B: Funnel plots for visual assessment of publication bias for studies reporting relative risk of CVD mortality for schizophrenia compared with controls, CHD. Fig C: Funnel plots for visual assessment of publication bias for studies reporting relative risk of CVD mortality for schizophrenia compared with controls, all circulatory disease. Fig D: Funnel plots for visual assessment of publication bias for studies reporting relative risk of CVD mortality for BD compared with controls, CVA. Fig E: Funnel plots for visual assessment of publication bias for studies reporting relative risk of CVD mortality for BD compared with controls, CHD. Fig F: Funnel plots for visual assessment of publication bias for studies reporting relative risk of CVD mortality for BD compared with controls, all circulatory disease. Table A: Results of Egger tests for publication bias, mortality outcomes. Fig G: Funnel plot of schizophrenia and CHD mortality including 5 unpublished studies estimated from trim and fill. Fig H: Funnel plot of schizophrenia and all circulatory disease mortality including 2 unpublished studies estimated from trim and fill. BD, bipolar disorder; CHD, coronary heart disease; CVA, cerebrovascular accident; CVD, cardiovascular disease.
(PDF)

S23 File Assessment of publication bias: incidence studies. Fig A: Funnel plots for visual assessment of publication bias for studies reporting relative risk of CVD incidence for schizophrenia compared with controls, CVA. Fig B: Funnel plots for visual assessment of publication bias for studies reporting relative risk of CVD incidence for schizophrenia compared with controls, CHD. Fig C: Funnel plots for visual assessment of publication bias for studies reporting relative risk of CVD incidence for schizophrenia compared with controls, major cardiovascular events. Fig D: Funnel plots for visual assessment of publication bias for studies reporting relative risk of CVD incidence for schizophrenia compared with controls, heart failure. Fig E: Funnel plots for visual assessment of publication bias for studies reporting relative risk of CVD incidence for BD compared with controls, CVA. Fig F: Funnel plots for visual assessment of publication bias for studies reporting relative risk of CVD incidence for BD compared with controls, CHD. Fig G: Funnel plots for visual assessment of publication bias for studies reporting relative risk of CVD incidence for BD compared with controls, major cardiovascular events. Fig H: Funnel plots for visual assessment of publication bias for studies reporting relative risk of CVD incidence for BD compared with controls, heart failure. Fig I: Funnel plots for visual assessment of publication bias for studies reporting relative risk of CVD incidence for schizophrenia compared with controls, all CVD. Fig J: Funnel plots for visual assessment of publication bias for studies reporting relative risk of CVD incidence for BD compared with controls, all CVD. Fig K: Funnel plots for visual assessment of publication bias for studies reporting relative risk of CVD incidence for schizophrenia and BD compared with controls, CVA. Fig L: Funnel plots for visual assessment of publication bias for studies reporting relative risk of CVD incidence for schizophrenia and BD compared with controls, CHD. Fig M: Funnel plots for visual assessment of publication bias for studies reporting relative risk of CVD incidence for schizophrenia and BD compared with controls, major cardiovascular events. Fig N: Funnel plots for visual assessment of publication bias for studies reporting relative risk of CVD incidence for schizophrenia and BD compared with controls, heart failure. Fig O: Funnel plots for visual assessment of publication bias for studies reporting relative risk of CVD incidence for schizophrenia and BD compared with controls, all CVD. Table A: Results of Egger tests for publication bias, incidence outcomes. BD, bipolar disorder; CHD, coronary heart disease; CVA, cerebrovascular accident; CVD, cardiovascular disease.
(PDF)

S24 File Example calculations for estimating unreported cases. (DOCX)

## Abbreviations

BD: bipolar disorder
CHD: coronary heart disease
CI: confidence interval
CVA: cerebrovascular accident
CVD: cardiovascular disease
DALY: disability-adjusted life year
DSM: Diagnostic and Statistical Manual of Mental Disorders
FGA: first-generation antipsychotic
HR: hazard ratio
ICD: International Classification of Diseases
MOOSE: Meta-analysis Of Observational Studies in Epidemiology
NOS: Newcastle–Ottawa scale
OR: odds ratio
PRISMA: Preferred Reporting Items for Systematic Review and Meta-Analyses
RR: risk ratio
SGA: second-generation antipsychotic
SIR: standardised incidence ratio
SMI: severe mental illness
SMR: standardised mortality ratio
WHO: World Health Organization
