# Supplementary material for: Temporal trends in associations between severe mental illness and risk of cardiovascular disease: A systematic review and meta-analysis
Source: PLoS Med. 2022 Apr 19;19(4):e1003960. doi: 10.1371/journal.pmed.1003960 (PMC9017899; doi:10.1371/journal.pmed.1003960)
Supplement: S3 File — (DOCX) [file pmed.1003960.s003.docx]

# S3 File. Deviations from protocol

| **Protocol** | **Deviation** | **Rationale** |
| --- | --- | --- |
| Ages 16+ | Ages 16-65 | The upper age limit was chosen because onset of psychotic disorder is uncommon at older ages, but transient psychotic conditions are associated with dementia making it difficult to distinguish between this and SMI |
| Include RCTs | Exclude RCTs | Not appropriate for research question |
| Subgroup analysis by length of follow- up | Subgroup analysis by length of follow-up not carried out | Most studies did not report the mean duration of follow- up. Also, it may not be a reliable source of heterogeneity. For prevalent cases it may be biased because people included are those who haven’t already died. For incident cases then outcomes may not occur in a short follow-up. Will also depend on age group. |
