# Supplementary material for: Temporal trends in associations between severe mental illness and risk of cardiovascular disease: A systematic review and meta-analysis
Source: PLoS Med. 2022 Apr 19;19(4):e1003960. doi: 10.1371/journal.pmed.1003960 (PMC9017899; doi:10.1371/journal.pmed.1003960)
Supplement: S4 File — (DOCX) [file pmed.1003960.s004.docx]

# S4 File. Inclusion and exclusion criteria

| **PICOS** | **Include** | **Exclude** |
| --- | --- | --- |
| Population | SMI in adults aged 16-65 (if a study includes people over 65, include as long as the majority are aged 16-65)  Schizophrenia  Bipolar disorder  Other psychotic condition | Dis-associative, multiple personality disorders  Depression in the absence of psychosis or bipolar  Dementia  Learning disabilities  OCD  Puerperal psychoses |
| Comparator | General population or people without SMI | Other chronic condition as comparator *eg* diabetes |
| Outcome | CVD mortality or morbidity  CHD event or diagnosis  Acute coronary syndrome, Myocardial Infarction, coronary thrombosis, angina  Coronary revascularisation  Cerebrovascular accident (CVA)  Stroke/TIA  Heart failure | Exclude CVD outcomes prior to SMI diagnosis  CVD risk factors, *ie* Cardio-metabolic disease (diabetes, hypertension, hyperlipidaemia)  Cardiovascular risk (*eg* Framingham 10 year risk score)  Cardiac complications of medication or surgery |
| Study design | RCTs  Non-randomised controlled trials  Cohort studies  Case-control studies | Uncontrolled studies  Cross-sectional studies  Case series  Single case reports  Narrative reviews |
| Setting | Developed countries:  Australia, Austria, Belgium, Canada, Cyprus, Czech Republic, Denmark, Estonia, Finland, France, Germany, Greece, Hong Kong, Iceland, Ireland, Israel, Italy, Japan, Latvia, Lithuania, Luxembourg, Macao, Malta, Netherlands, New Zealand, Norway, Portugal, Puerto Rico, San Marino, Singapore, Slovak Republic, Slovenia, South Korea, Spain, Sweden, Switzerland, Taiwan, UK, USA | Other countries  Emerging and developing Europe (*eg* Croatia, Poland, Romania) |
| Follow-up | 1 year or more | Follow-up less than 1 year |

SMI – severe mental illness, CVD – cardiovascular disease, OCD – obsessive compulsive disorder,

CHD – coronary heart disease, TIA – transient ischaemic attack, RCTs – randomised controlled trials
