# Supplementary material for: Temporal trends in associations between severe mental illness and risk of cardiovascular disease: A systematic review and meta-analysis
Source: PLoS Med. 2022 Apr 19;19(4):e1003960. doi: 10.1371/journal.pmed.1003960 (PMC9017899; doi:10.1371/journal.pmed.1003960)
Supplement: S5 File — (DOCX) [file pmed.1003960.s005.docx]

# S5 File. Search strategies by database

**MEDLINE, via Ovid platform, 1946 to November Week 3 2021**

Search dates: 19 September 2017 (including MEDLINE in-Process), updated 8 March 2019 (including MEDLINE in-Process), and 30 November 2021

1 ((serious or severe or chronic or persistent) adj ("mental illness*" or "mental disorder$1" or "mental disease$1")).ti,ab.

2 exp Schizophrenia/ or Schizophrenia.mp.

3 exp Schizotypal Personality Disorder/ or Schizotypal Personality Disorder$1.mp.

4 ("dementia pr?ecox" or "schizo affective" or schizoaffective or schizophreniform or hebephrenia or paraphrenia or oneirophrenia).mp.

5 affective disorders, psychotic/ or capgras syndrome/ or delusional parasitosis/ or morgellons disease/ or paranoid disorders/ or psychotic disorders/

6 ("psychotic affective disorder$1" or capgras or "delusional misidentification syndrome" or morgellons or "cotard$2 syndrome" or "delusional parasitosis" or "delusional disorder$1" or "psychotic disorder$1" or psychos#s).mp.

7 Paranoid Disorders/ or ("paranoid disorder$1" or "paranoid ideation" or paranoia).mp.

8 ("sander$2 disease" or "folie a deux" or "boufee delirante" or "sensitiver beziehungswahn").mp.

9 exp "bipolar and related disorders"/ or bipolar disorder$1.mp.

10 ((manic or bipolar) adj depression$1).ti,ab.

11 mania.mp.

12 1 or 2 or 3 or 4 or 5 or 6 or 7 or 8 or 9 or 10 or 11

13 Cardiovascular Diseases/ or Cardiovascular disease$1.mp.

14 ((coronary or isch?emic) adj2 (disease or occlusion or stenos#s or thrombos#s)).ti,ab.

15 exp Myocardial Ischemia/

16 (myocardial adj (isch?emia or infarct$)).ti,ab.

17 exp Angina Pectoris/ or angina.mp.

18 exp Myocardial Revascularization/

19 ((coronary or myocardial or heart or cardiac) adj2 (revasculari?ation or angioplasty or atherectomy or bypass)).ti,ab.

20 exp Heart Failure/

21 ((heart or cardiac or ventricular) adj failure).ti,ab.

22 exp Ventricular Dysfunction/

23 ((ventricular or systolic or diastolic) adj (dysfunction or impairment)).ti,ab.

24 exp brain ischemia/ or exp intracranial arterial diseases/ or exp "intracranial embolism and thrombosis"/ or exp stroke/

25 (stroke or "cerebrovascular accident").mp.

26 ((brain or cerebral or intracranial) adj2 (infarct$ or thrombos?s or embolism)).ti,ab.

27 13 or 14 or 15 or 16 or 17 or 18 or 19 or 20 or 21 or 22 or 23 or 24 or 25 or 26

28 12 and 27

29 limit 28 to humans

30 limit 29 to "qualitative (best balance of sensitivity and specificity)"

31 29 not 30

32 limit 31 to "reviews (best balance of sensitivity and specificity)"

33 31 not 32

34 limit 33 to systematic reviews

35 33 not 34

36 limit 35 to yr="2019 -Current"

**Embase, via Ovid platform, 1974 to 2021 November 29**

Search dates: 19 September 2017, updated 8 March 2019 and 30 November 2021

1 ((serious or severe or chronic or persistent) adj ("mental illness*" or "mental disorder$1" or "mental disease")).ti,ab.

2 schizophrenia/ or schizophrenia.mp.

3 schizotypal personality disorder/ or schizotypal personality disorder$1.ti,ab.

4 ("dementia pr?ecox" or "dementia precox" or schizoaffective or "schizo affective" or schizophreniform or hebephrenia or paraphrenia or oneirophrenia).ti,ab.

5 affective psychosis/ or schizoaffective psychosis/ or Capgras syndrome/ or delusional parasitosis/ or Morgellons disease/ or delusional misidentification/ or delusional disorder/

6 ("psychotic affective disorder$1" or capgras or "delusional misidentification" or morgellons or "cotard$2 syndrome" or "delusional parasitosis" or "delusional disorder$1" or "psychotic disorder$1" or psychos#s).ti,ab.

7 psychosis/

8 paranoid psychosis/ or ("paranoid psychos#s" or "paranoid disorder$1" or "paranoid ideation" or paranoia).ti,ab.

9 ("sander$2 disease" or "folie a deux" or "boufee delirante" or "sensitiver beziehungswahn").ti,ab.

10 exp bipolar disorder/ or bipolar disorder$1.mp.

11 ((manic or bipolar) adj depression$1).ti,ab.

12 mania.ti,ab.

13 1 or 2 or 3 or 4 or 5 or 6 or 7 or 8 or 9 or 10 or 11 or 12

14 cardiovascular disease/ or cardiovascular disease$1.ti,ab.

15 ((coronary or isch?emic) adj2 (disease or occlusion or stenos#s or thrombos#s)).ti,ab.

16 ischemic heart disease/ or exp acute coronary syndrome/ or exp angina pectoris/ or coronary artery atherosclerosis/ or coronary artery obstruction/ or coronary artery thrombosis/ or exp heart infarction/ or heart muscle ischemia/ or ischemic cardiomyopathy/

17 (myocardial adj (isch?emia or infarct$)).ti,ab.

18 angina pectoris/ or angina.ti,ab.

19 heart muscle revascularization/

20 ((coronary or myocardial or heart or cardiac) adj2 (revasculari?ation or angioplasty or atherectomy or bypass)).ti,ab.

21 heart failure/

22 ((heart or cardiac or ventricular) adj failure).ti,ab.

23 heart ventricle function/

24 ((ventricular or systolic or diastolic) adj (dysfunction or impairment)).ti,ab.

25 cerebrovascular disease/ or exp brain infarction/ or exp brain ischemia/ or exp cerebrovascular accident/

26 (stroke or "cerebrovascular accident" or tia or "transient isc?emic attack").ti,ab.

27 ((brain or cerebral or intracranial) adj2 (infarct$ or thrombos?s or embolism)).ti,ab.

28 14 or 15 or 16 or 17 or 18 or 19 or 20 or 21 or 22 or 23 or 24 or 25 or 26 or 27

29 13 and 28

30 limit 29 to human

31 limit 30 to "qualitative (best balance of sensitivity and specificity)"

32 30 not 31

33 limit 32 to "reviews (best balance of sensitivity and specificity)"

34 32 not 33

**PsycInfo via Ovid platform, 1967 to November Week 4 2021**

Search dates: 19 September 2017, updated 8 March 2019 and 30 November 2021

1 ((serious or severe or chronic or persistent) adj ("mental illness*" or "mental disorder$1" or "mental disease")).ti,ab.

2 exp schizophrenia/ or schizophrenia.mp.

3 exp schizotypal personality disorder/ or Schizotypal Personality Disorder$1.mp.

4 ("dementia praecox" or "dementia precox" or schizoaffective or "schizo affective" or schizophreniform or hebephrenia or paraphrenia or oneirophrenia).mp.

5 affective psychosis/ or exp schizoaffective disorder/ or capgras syndrome/

6 ("psychotic affective disorder$1" or capgras or "delusional misidentification syndrome" or morgellons or "cotard$2 syndrome" or "delusional parasitosis" or "delusional disorder$1" or "psychotic disorder$1").mp.

7 exp psychosis/ or psychos#s.mp.

8 paranoid schizophrenia/ or paranoid personality disorder/ or exp "paranoia (psychosis)"/ or ("paranoid disorder$1" or "paranoid ideation" or paranoia).mp.

9 ("sander$2 disease" or "folie a deux" or "boufee delirante" or "sensitiver beziehungswahn").mp.

10 exp bipolar disorder/ or bipolar disorder$1.mp.

11 ((manic or bipolar) adj depression$1).ti,ab.

12 mania.mp.

13 1 or 2 or 3 or 4 or 5 or 6 or 7 or 8 or 9 or 10 or 11 or 12

14 cardiovascular disorders/ or exp heart disorders/ or exp arteriosclerosis/ or exp ischemia/

15 ((coronary or isch?emic) adj2 (disease or occlusion or stenos#s or thrombos#s)).ti,ab.

16 (myocardial adj (isch?emia or infarct$)).ti,ab.

17 ((coronary or myocardial or heart or cardiac) adj2 (revasculari?ation or angioplasty or atherectomy or bypass)).ti,ab.

18 ((heart or cardiac or ventricular) adj failure).ti,ab.

19 ((ventricular or systolic or diastolic) adj (dysfunction or impairment)).ti,ab.

20 cerebrovascular disorders/ or cerebral arteriosclerosis/ or exp cerebral ischemia/ or cerebrovascular accidents/

21 (stroke or "cerebrovascular accident").mp.

22 ((brain or cerebral or intracranial) adj2 (infarct$ or thrombos?s or embolism)).ti,ab.

23 14 or 15 or 16 or 17 or 18 or 19 or 20 or 21 or 22

24 13 and 23

25 limit 24 to human

**Cinahl via EBSCO platform**

Search dates: 19 September 2017, updated 8 March 2019 and 30 November 2021

| # | Query |
| --- | --- |
| S29 | S27 AND S28 |
| S28 | S13 OR S14 OR S15 OR S16 OR S17 OR S18 OR S19 OR S20 OR S21 OR S22 OR S23 OR S24 OR S25 OR S26 |
| S27 | S1 OR S2 OR S3 OR S4 OR S5 OR S6 OR S7 OR S8 OR S9 OR S10 OR S11 OR S12 |
| S26 | Tl ("brain" OR "cerebral" OR "intracranial") W2 ("infarct*" OR "thrombos?s" OR "embolism") OR AB ("brain" OR "cerebral" OR "intracranial") W2 ("infarct*" OR "thrombos?s" OR "embolism") |
| S25 | '"'stroke" OR "cerebrovascular accident"" |
| S24 | (MH "Cerebral lschemia+") OR (MH "lntracranial Arterial Diseases+") OR (MH "lntracranial Embolism and Thrombosis+") OR (MH "Stroke+") |
| S23 | Tl ("ventricular" OR "systolic" OR "diastolic") W1 ("dysfunction" OR "impairment") OR AB ("ventricular" OR "systolic" OR "diastolic") W1 ("dysfunction" OR "impairment") |
| S22 | (MH "Ventricular Dysfunction+") |
| S21 | Tl ("heart" OR "cardiac" OR "ventricular") W1 "failure" OR AB ("heart" OR "cardiac" OR "ventricular") W1 "failure" |
| S20 | (MH "Heart Failure+") |
| S19 | Tl ("coronary" OR "myocardial" OR "heart" OR "cardiac") W2 ("revasculari#ation" OR "angioplasty" OR "atherectomy" OR "bypass") OR AB ("coronary" OR "myocardial" OR "heart" OR "cardiac") W2 ("revasculari#ation" OR "angioplasty" OR "atherectomy" OR "bypass") |
| S18 | (MH "Myocardial Revascularization+") |
| S17 | ""angina"" |
| S16 | Tl "myocardial" W1 ("isch#emia" OR "infarct*") OR AB "myocardial" W1 ("isch#emia" OR "infarct*") |
| S15 | (MH "Myocardial lschemia+") |
| S14 | Tl ("coronary" OR "isch#emic") W2 ("disease*" OR "occlusion" OR "stenos?s" OR "thrombos?s") OR AB ("coronary" OR "isch#emic") W2 ("disease*" OR "occlusion" OR "stenos?s" OR "thrombos?s") |
| S13 | (MH "Cardiovascular Diseases") OR "cardiovascular disease*" |
| S12 | ""mania'"' |
| S11 | Tl ("manic" OR "bipolar") W1 "depression*" OR AB ("manic" OR "bipolar") W1 "depression*" |
| S10 | '"'bipolar disorder*'"' |
| S9 | ""folie a deux" OR "boufee delirante" OR "sensitiver beziehungswahn"" |
| S8 | ""paranoid disorder*" OR "paranoid ideation" OR "paranoia"" |
| S7 | ""paranoid disorder*" OR "paranoid ideation" OR "paranoia"" |
| S6 | ""psychotic affective disorder*" OR "capgras" OR "delusional misidentification" OR "morgellons" OR "cotard*" OR "delusional parasitosis" OR "delusional disorder*" OR "psychotic disorder*" OR "psychos?s"" |
| S5 | ""psychotic affective disorder*" OR "capgras" OR "delusional misidentification" OR "morgellons" OR "cotard*" OR "delusional parasitosis" OR "delusional disorder*" OR "psychotic disorder*" OR "psychos?s"" |
| S4 | (MH "Morgellons disease") |
| S3 | ""schizophrenia" OR "schizotypal personality disorder*" OR "dementia pr#ecox" OR "schizoaffective " OR "schizo affective" OR "schizophreniform" OR "hebephrenia" OR "paraphrenia" OR "oneirophrenia"" OR (MH "Bipolar Disorder") OR (MH "Schizoaffective Disorder") |
| S2 | (MH "Psychotic Disorders") OR (MH "Affective Disorders, Psychotic+") OR (MH "Delusions+") OR (MH "Psychoses, Substance-Induced+") OR (MH "Schizophrenia+") |
| S1 | Tl ("serious" OR "severe" OR "chronic" OR "persistent") W1 ("mental illness*" OR "mental disorder*" OR "mental disease*") OR AB ("serious" OR "severe" OR "chronic" OR "persistent") W1 ("mental illness*" OR "mental disorder*" OR "mental disease*") |

**Web of Science conference proceedings abstracts**

Search dates: 19 September 2017, updated 8 March 2019 and 30 November 2021

| 1 | TI=((severe OR serious OR chronic OR persistent) NEAR/1 ("mental illness*" OR "mental disorder*" OR "mental disease*")) |
| --- | --- |
| 2 | TI=(schizophrenia OR "schizotypal personality disorder*" OR schizoaffective OR "schizo affective" OR "dementia pr$ecox" OR schizophreniform OR hebephrenia OR paraphrenia OR oneirophrenia) |
| 3 | TI=("psychotic affective disorder*" OR capgras OR "delusional parasitosis" OR "delusional misidentification syndrome" OR morgellons OR "cotard$2 syndrome" OR "delusional disorder*" OR "psychotic disorder*" OR "psychos?s") |
| 4 | TI=("paranoid disorder" OR "paranoid ideation" OR paranoia) |
| 5 | TI=("sander$2 disease" OR "folie a deux" OR "boufee delirante" OR "sensitiver beziehungswahn") |
| 6 | TS=(bipolar NEAR/2 disorder* OR schizophrenia) |
| 7 | TI=(mania OR (manic NEAR/1 depression)) |
| 8 | #7 OR #6 OR #5 OR #4 OR #3 OR #2 OR #1 |
| 9 | TS=("cardiovascular disease") |
| 10 | TI=(coronary NEAR/2 (disease OR occlusion OR stenos?s OR thrombos?s) OR isch$emic NEAR/2 (disease OR occlusion OR stenos?s OR thrombos?s)) |
| 11 | TI=("acute coronary syndrome" OR angina OR "coronary artery atherosclerosis" OR "coronary artery obstruction" OR (heart OR myocardial infarct*)) |
| 12 | TI=("myocardial isch$emia") |
| 13 | TI=((coronary OR myocardial OR heart OR cardiac) NEAR/2 (revasculari?ation OR angioplasty OR atherectomy OR bypass)) |
| 14 | TI=((heart OR cardiac OR ventricular) NEAR/1 failure) |
| 15 | TI=((ventricular OR systolic OR diastolic) NEAR/1 (dysfunction OR impairment)) |
| 16 | TI=("cerebrovascular disease" OR "brain infarction" OR "brain ischemia" OR "cerebrovascular accident" OR stroke OR tia OR "transient isch?emic attack") |
| 17 | TI=((brain OR cerebral OR intracranial) NEAR/2 (infarct* OR thrombos?s or embolism)) |
| 18 | #17 OR #16 OR #15 OR #14 OR #13 OR #12 OR #11 OR #10 OR #9 |
| 19 | #18 AND #8 |

**Zetoc conference proceedings abstracts**

Search dates: 19 September 2017, updated 8 March 2019 and 30 November 2021

| mental cardio* |
| --- |
| mental coronary |
| mental myocardial |
| Mental angina |
| Mental heart |
| mental cardiac |
| Mental ventric* |
| schizo* cardio* |
| schizo* coronary |
| schizo* myocardial |
| Schizo* angina |
| schizo* heart |
| schizo* cardiac |
| schizo* ventric* |
| bipolar disorder cardio* |
| bipolar disorder coronary |
| bipolar disorder myocardial |
| bipolar disorder angina |
| bipolar disorder heart |
| bipolar disorder cardiac |
| bipolar disorder ventric* |
| bipolar disorder stroke |
| schizophrenia stroke |
| mental ischaemi* |
| schizo* ischaemi* |
| bipolar disorder ischemi* |
| bipolar disorder ischaemi* |
| schizo* ischemi* |
| mental ischemi* |
| bipolar disorder cerebrovascular |
| schizo* cerebrovascular |
| psychosis cardio* |
| Psychosis coronary |
| Psychosis myocardial |
| Psychosis angina |
| Psychosis heart |
| psychosis cardiac |
| psychosis ventric* |
| psychosis stroke |
| psychosis cerebrovascular |

**Cochrane database**

Search dates: 19 September 2017, updated 9 March 2019 (Cochrane reviews and systematic reviews), 10 September 2019 (CENTRAL trials register, trials added up to 8 March 2019 only) and 1 December 2021

ID Search term

#1 ((serious or severe or chronic or persistent) next ("mental illness*" or "mental disorder*" or "mental disease"))

#2 schizophrenia or schizoaffective or schizotypal or schizophreniform

#3 bipolar near/2 disorder

#4 "psychotic disorder" or psychosis or psychotic

#5 delusional (disorder or parasitosis or misidentification)

#6 paranoid (disorder or ideation) or paranoia

#7 "manic depression" or mania

#8 MeSH descriptor: [Bipolar and Related Disorders] explode all trees

#9 MeSH descriptor: [Psychoses, Substance-Induced] explode all trees

#10 MeSH descriptor: [Schizophrenia Spectrum and Other Psychotic Disorders] explode all trees

#11 [1-#10-#10-#10-#10-#10-#10-#10-#10]

#12 cardiovascular disease*

#13 (coronary or ischaemic or ischemic) near/2 (disease or occlusion or stenos?s or thrombos?s)

#14 ("coronary" or "myocardial" or "heart" or "cardiac") near/2 ("revasculari#ation" or "angioplasty" or "atherectomy" or "bypass")

#15 (heart or cardiac or ventricular) next (failure or dysfunction or impairment)

#16 stroke or "cerebrovascular accident"

#17 transient isch?emic attack

#18 MeSH descriptor: [Cardiovascular Diseases] explode all trees

#19 MeSH descriptor: [Stroke] explode all trees

#20 MeSH descriptor: [Heart Failure] explode all trees

#21 MeSH descriptor: [Myocardial Revascularization] explode all trees

#22 {or #12-#21}

#23 #11 and #22
