# Supplementary material for: Temporal trends in associations between severe mental illness and risk of cardiovascular disease: A systematic review and meta-analysis
Source: PLoS Med. 2022 Apr 19;19(4):e1003960. doi: 10.1371/journal.pmed.1003960 (PMC9017899; doi:10.1371/journal.pmed.1003960)
Supplement: S6 File — (DOCX) [file pmed.1003960.s006.docx]

# S6 File. Data extraction form

List of variables extracted from eligible mortality and incidence studies

| **Variable list** |  |  |
| --- | --- | --- |
| Date of extraction |  |  |
| Record number (Endnote) |  |  |
| Author/year |  |  |
| If same study, why is this record different? |  |  |
| Country |  |  |
| Source of Data |  |  |
| Setting |  |  |
| Period of Data Collecting |  |  |
| Period of results |  |  |
| SMI definition |  |  |
| CVD definition |  |  |
| Diagnosis type (schizophrenia, bipolar disorder, mixed SMI) |  |  |
| Control group (general population, no SMI) |  |  |
| Inclusion criteria Case |  |  |
| Inclusion Criteria Control |  |  |
| Design (cohort, case-control) |  |  |
| N Case |  |  |
| N Controls |  |  |
| Number of extra covariates used in the analysis (excluding age and sex) |  |  |
| Type of covariates used in the analysis (if SMR assumed to have matched by age, sex and year) |  |  |
| Follow-up (years mean) |  |  |
| N Cerebrovascular | P, M, F | Exposed, unexposed |
| N CHD | P, M, F | Exposed, unexposed |
| N HEART FAILURE | P, M, F | Exposed, unexposed |
| N CVD | P, M, F | Exposed, unexposed |
| N Cerebrovascular DEATH | P, M, F | Exposed, unexposed |
| N CHD DEATH | P, M, F | Exposed, unexposed |
| N CVD DEATH | P, M, F | Exposed, unexposed |
| Risk ratio Cerebrovascular | P, M, F | Adj, unadj |
| Risk ratio 95% confidence limits | P, M, F | Adj, unadj |
| Risk ratio CHD | P, M, F | Adj, unadj |
| Risk ratio 95% confidence limits | P, M, F | Adj, unadj |
| Risk ratio HF | P, M, F | Adj, unadj |
| Risk ratio 95% confidence limits | P, M, F | Adj, unadj |
| Risk ratio CVD | P, M, F | Adj, unadj |
| Risk ratio 95% confidence limits | P, M, F | Adj, unadj |
| Risk ratio DEATH Cerebrovascular | P, M, F | Adj, unadj |
| Risk ratio 95% confidence limits | P, M, F | Adj, unadj |
| Risk ratio DEATH CHD | P, M, F | Adj, unadj |
| Risk ratio 95% confidence limits | P, M, F | Adj, unadj |
| Risk ratio DEATH CVD | P, M, F | Adj, unadj |
| Risk ratio 95% confidence limits | P, M, F | Adj, unadj |
| Rate Ratio Cerebrovascular | P, M, F | Adj, unadj |
| Rate ratio 95% confidence limits | P, M, F | Adj, unadj |
| Rate Ratio CHD | P, M, F | Adj, unadj |
| Rate ratio 95% confidence limits | P, M, F | Adj, unadj |
| Rate Ratio HF | P, M, F | Adj, unadj |
| Rate ratio 95% confidence limits | P, M, F | Adj, unadj |
| Rate Ratio CVD | P, M, F | Adj, unadj |
| Rate ratio 95% confidence limits | P, M, F | Adj, unadj |
| Rate Ratio DEATH Cerebrovascular | P, M, F | Adj, unadj |
| Rate ratio 95% confidence limits | P, M, F | Adj, unadj |
| Rate Ratio DEATH CHD | P, M, F | Adj, unadj |
| Rate ratio 95% confidence limits | P, M, F | Adj, unadj |
| Rate Ratio DEATH CVD | P, M, F | Adj, unadj |
| Rate ratio 95% confidence limits | P, M, F | Adj, unadj |
| HR CEREBROVASCULAR | P, M, F | Adj, unadj |
| HR 95% confidence limits | P, M, F | Adj, unadj |
| HR CHD | P, M, F | Adj, unadj |
| HR 95% confidence limits | P, M, F | Adj, unadj |
| HR HEART FAILURE | P, M, F | Adj, unadj |
| HR 95% confidence limits | P, M, F | Adj, unadj |
| HR CVD | P, M, F | Adj, unadj |
| HR 95% confidence limits | P, M, F | Adj, unadj |
| HR DEATH Cerebrovascular | P, M, F | Adj, unadj |
| HR 95% confidence limits | P, M, F | Adj, unadj |
| HR DEATH CHD | P, M, F | Adj, unadj |
| HR 95% confidence limits | P, M, F | Adj, unadj |
| HR DEATH CVD | P, M, F | Adj, unadj |
| HR 95% confidence limits | P, M, F | Adj, unadj |
| OR Cerebrovascular | P, M, F | Adj, unadj |
| OR 95% confidence limits | P, M, F | Adj, unadj |
| OR CHD | P, M, F | Adj, unadj |
| OR 95% confidence limits | P, M, F | Adj, unadj |
| OR HEART FAILURE | P, M, F | Adj, unadj |
| OR 95% confidence limits | P, M, F | Adj, unadj |
| OR CVD | P, M, F | Adj, unadj |
| OR 95% confidence limits | P, M, F | Adj, unadj |
| SMR Cerebrovascular | P, M, F | Unadj |
| SMR 95% confidence limits | P, M, F | Unadj |
| SMR CHD | P, M, F | Unadj |
| SMR CHD 95% confidence limits | P, M, F | Unadj |
| SMR HEART FAILURE | P, M, F | Unadj |
| SMR HF 95% confidence limits | P, M, F | Unadj |
| SMR CVD | P, M, F | Unadj |
| SMR CVD 95% confidence limits | P, M, F | Unadj |
| Mean age baseline | Exposed, unexposed | |
| SD age baseline | Exposed, unexposed | |
| Male (%) | Exposed, unexposed | |
| Basal BMI | Exposed, unexposed | |
| SD BMI | Exposed, unexposed | |
| White ethnicity % | Exposed, unexposed | |
| Alcohol comorbidity % | Exposed, unexposed | |
| Substance abuse % | Exposed, unexposed | |
| Obesity % | Exposed, unexposed | |
| Diabetes % | Exposed, unexposed | |
| Congestive Heart Failure % | Exposed, unexposed | |
| Coronary Artery Disease % | Exposed, unexposed | |
| Stroke % | Exposed, unexposed | |
| Any Baseline CVD % | Exposed, unexposed | |
| Hypertension % | Exposed, unexposed | |
| Hyperlipidemia % | Exposed, unexposed | |
| Smoking % | Exposed, unexposed | |
| CKD/Renal % | Exposed, unexposed | |
| Respiratory % | Exposed, unexposed | |
| Married % | Exposed, unexposed | |
| Employed % | Exposed, unexposed | |
| Poor income % | Exposed, unexposed | |
| Least Urbanized % | Exposed, unexposed | |
| Is age at diagnosis between 16 and 65? |  |  |
| Limitations |  |  |
| Medication |  |  |
| Notes |  |  |

*SMI – severe mental illness, CHD – coronary heart disease, CVD – cardiovascular disease, N – number, HR – hazard ratio, OR – odds ratio, SMR – standardised mortality ratio, P – persons, M – males, F – females, Unad – results from models adjusted for age and sex only, Adj – fully adjusted models, SD – standard deviation, BMI – body mass index, CKD – chronic kidney disease*
