# Supplementary material for: Temporal trends in associations between severe mental illness and risk of cardiovascular disease: A systematic review and meta-analysis
Source: PLoS Med. 2022 Apr 19;19(4):e1003960. doi: 10.1371/journal.pmed.1003960 (PMC9017899; doi:10.1371/journal.pmed.1003960)
Supplement: S7 File — Table A: Risk of bias form for cohort studies. Table B: Risk of bias form for case–control studies. (DOCX) [file pmed.1003960.s007.docx]

# S7 File. Risk of bias forms for mortality and incidence studies

Table A: Risk of bias form for cohort studies

|  |  | **Assessment** | | |
| --- | --- | --- | --- | --- |
|  | **Risk of bias assessment criteria:** | **Yes** | **Unclear** | **No** |
| **Selection** | Truly or somewhat representative of the average person with SMI in the community | Community setting | People from insurance claims databases, inpatients (but not long stay) - likely to be more severe cases, but in some countries most people with SMI are admitted at some stage | Long stay inpatients, specific ethnic, sex, occupation groups. People on specific medication eg lithium, clozapine |
|  | Non-exposed drawn from the same community as the exposed cohort | Matched or unmatched controls from same database source as cases | General population for same geographic area as cases. Whole population register studies with cohort follow-up. | General population from larger or different geographic area. Any study using population mortality rates to calculate expected rates. |
|  | Ascertainment of exposure from secure record *e.g.* medical record or structured interview | Clinical records in administrative databases. Medical insurance claims databases with high population coverage eg Taiwan | Records from most insurance claims databases | Self-reported diagnosis or based on sickness or disability records without validation. |
|  | Demonstration that CVD was not present at start of study | Good methodology for excluding people with prior CVD | Some description of methods for excluding people with CVD, but unclear whether effective. May exclude people with some but not all CVD diagnoses *e.g.* stroke but not AMI | Any outcome of CVD mortality if previous CVD not accounted for |
| **Comparability** | Study controls for age and sex | All papers should control for age and sex, either by matching or in analysis | Could be CT if very broad age groups (age bands >10 years) | Shouldn't be any in this category |
|  | Study controls for any additional sociodemographic factor (Deprivation, ethnicity, other sociodemographic) | More than one factor sociodemographic factor (Deprivation, ethnicity, sociodemographic, income, employment, urban/rural, education, etc) | Only one sociodemographic factor | No sociodemographic factors |
|  | Have the authors taken account of any additional lifestyle confounding factors (in design or analysis)? | Several of: Smoking, BMI, BP, comorbidity, alcohol, medication. (Diabetes plus another relevant comorbidity would fall into this category.) | One of: Smoking, BMI, BP, comorbidity, alcohol, medication (if 2 medications *e.g.* cholesterol lowering + BP lowering, then put in this category) | None |
| **Outcome** | Assessment of CVD by independent blind assessment or record linkage. Was the outcome accurately measured to minimise bias?  • do the measurements truly reflect what you want them to (have they been validated) • has a reliable system been established for detecting all the cases (for measuring disease occurrence) • were the measurement methods similar in the different groups | For deaths or CVD events with no indication of prior CVD diagnosis we can't be sure of ascertaining all cases. Studies only fall in this category if they capture all CHD or stroke diagnoses | Linked records. Best mortality studies have autopsy-confirmed CVD deaths | Patient reported outcome |
|  | Was follow-up long enough for outcomes to occur (min of 5 years) | All follow-up at least 5 years or if likely that the majority (>90%) are over 5 years. | Mean at least 5 years | Mean less than 5 years |
|  | Adequacy of follow-up of SMI and non-SMI cohorts: complete follow- up - all subjects accounted for or subjects lost to follow-up unlikely to introduce bias or statement that loss to follow-up in both groups was similar | Small rate of loss to follow-up or statement accounting for all subjects. Scandinavian (and Taiwanese) studies could fall into this category | Studies where it is unclear if people leaving the area would have affected results or if follow-up is specifically mentioned | High levels of loss to follow-up or no mention of numbers followed up |

Table B: Risk of bias form for case-control studies

|  |  | **Assessment** | | |
| --- | --- | --- | --- | --- |
|  | **Risk of bias assessment criteria:** | **Yes** | **Unclear** | **No** |
| Selection | Adequate definition of cases with CVD | All cases of CVD captured and validated. If deaths, then are we sure that cases include all people with previous CVD diagnosis. | Some description of methods for excluding people with CVD, but unclear whether effective. May exclude people with some but not all CVD diagnoses eg stroke but not AMI | No info on case definition |
|  | Cases with CVD are representative of defined population and reliable system used for selecting cases | Cases are representative and selection bias unlikely | Unclear if selection bias | Could be selection bias |
|  | Selection of controls without CVD | Controls selected from community and representative of defined population | Hospital controls | No info on selection of controls |
|  | Definition of controls - demonstration of no history of CVD | Good methodology for selecting controls with no history of CVD | Some description of methods for excluding people with CVD, but unclear whether effective. May exclude people with some but not all CVD diagnoses eg stroke but not AMI | Any outcome of CVD mortality or CVD events if previous CVD not accounted for |
| Comparability | Study controls for age and sex | All papers should control for age and sex, either by matching or in analysis | Could be CT if very broad age groups (age bands >10 years) | Shouldn't be any in this category |
|  | Study controls for any additional sociodemographic factor (Deprivation, ethnicity, other sociodemographic) | More than one factor sociodemographic factor (Deprivation, ethnicity, sociodemographic, income, employment, urban/rural, education, etc) | Only one sociodemographic factor | No sociodemographic factors |
|  | Study controls for additional lifestyle factor | Several of: Smoking, BMI, BP, comorbidity, alcohol, medication. (Diabetes plus another relevant comorbidity would fall into this category.) | One of: Smoking, BMI, BP, comorbidity, alcohol, medication (if 2 medications eg cholesterol lowering + BP lowering, then put in this category) | None |
| Exposure | Ascertainment of exposure from secure record eg medical record or structured interview  • do the measurements truly reflect what you want them to (have they been validated) • were the measurement methods similar in cases and controls | Do we assume that clinical records in admin databases are good quality? Some studies have done more to confirm diagnoses. Are those of better quality? | Records from insurance claims databases - or are they good quality? | Self-reported diagnosis or based on sickness or disability records without validation. |
|  | Does SMI exposure precede CVD outcome? | Yes | Unclear | No |
|  | Same method of ascertainment for cases and controls | Same method used | Unclear if different methods are used | No info on method of ascertainment |
|  | Non-response rate | Same rate for both groups | Unclear if cases have a different response rate from controls | No info on non-response rate |
