# Supplementary material for: Temporal trends in associations between severe mental illness and risk of cardiovascular disease: A systematic review and meta-analysis
Source: PLoS Med. 2022 Apr 19;19(4):e1003960. doi: 10.1371/journal.pmed.1003960 (PMC9017899; doi:10.1371/journal.pmed.1003960)
Supplement: S8 File — (DOCX) [file pmed.1003960.s008.docx]

# S8 File. Decision rules for overlapping studies

Y

Do studies overlap by period of recruitment?

Keep both studies

Are studies set in different parts of the country?

Is overlap > 10% of total recruitment time for both studies?

Do both studies have a reported or estimable number of exposed cases?

Choose the study with the largest number of exposed

Do both studies cover whole country?

Choose the study covering the larger geographical area

Choose the study with the longest follow-up duration.

Could studies potentially include the same patients at risk recruited at different times?

Do studies overlap by period of outcomes?

N

Y

N

N

Y

N

Y

Y

Y

N

N

N

Y

Keep both studies

Keep both studies
