# Supplementary material for: Temporal trends in associations between severe mental illness and risk of cardiovascular disease: A systematic review and meta-analysis
Source: PLoS Med. 2022 Apr 19;19(4):e1003960. doi: 10.1371/journal.pmed.1003960 (PMC9017899; doi:10.1371/journal.pmed.1003960)
Supplement: S9 File — (DOCX) [file pmed.1003960.s009.docx]

# S9 File. List of included mortality studies

| **Study ID** | **Article** |
| --- | --- |
| Ahrens, 1995 | Ahrens, B., Müller-Oerlinghausen, B., Schou, M., Wolf, T., Alda, M., Grof, E., et al. (1995). Excess cardiovascular and suicide mortality of affective disorders may be reduced by lithium prophylaxis. *Journal of Affective Disorders, 33*(2), 67-75. |
| Ajetunmobi, 2013 | Ajetunmobi, O., Taylor, M., Stockton, D., & Wood, R. (2013). Early death in those previously hospitalised for mental healthcare in Scotland: A nationwide cohort study, 1986-2010. *BMJ Open, 3*(7), e002768. |
| Allebeck, 1986 | Allebeck, P., & Wistedt, B. (1986). Mortality in Schizophrenia: A Ten-Year Follow-up Based on the Stockholm County Inpatient Register. *Archives of General Psychiatry, 43*(7), 650-653. |
| Anderson, 1991 | Anderson, C., Connelly, J., Johnstone, E. C., & Owens, D. G. C. (1991). Disabilities and circumstances of schizophrenic patients - A follow-up study: V. Cause of death. *British Journal of Psychiatry, 159*(OCT. SUPPL. 13), 30-33. |
| Angst, 2002 | Angst, F., Stassen, H. H., Clayton, P. J., & Angst, J. (2002). Mortality of patients with mood disorders: follow-up over 34-38 years. *Journal of Affective Disorders, 68*(2-3), 167-181. |
| Bjorkenstam, 2012 | Björkenstam, E., Ljung, R., Burström, B., Mittendorfer-Rutz, E., Hallqvist, J., & Weitoft, G. R. (2012). Quality of medical care and excess mortality in psychiatric patients—a nationwide register-based study in Sweden. *BMJ Open, 2*(1). |
| Brink, 2018 | Brink, M., Green, A., Bojesen, A. B., Lamberti, J. S., Conwell, Y., & Andersen, K. (2018). Excess medical comorbidity and mortality across the lifespan in schizophrenia.: A nationwide Danish register study. *Schizophrenia Research*. |
| Brodersen, 2000 | Brodersen, A., Licht, R. W., Vestergaard, P., Olesen, A. V., & Mortensen, P. B. (2000). Sixteen-year mortality in patients with affective disorder commenced on lithium. *British Journal of Psychiatry, 176*, 429-433. |
| Brook, 1984 | Brook, O. H. (1985). Mortality in the long-stay population of Dutch mental hospitals. *Acta Psychiatr Scand, 71*(6), 626-635. |
| Brown, 2010 | Brown, S., Kim, M., Mitchell, C., & Inskip, H. (2010). Twenty-five year mortality of a community cohort with schizophrenia. *Br J Psychiatr, 196*(2), 116 - 121. |
| Buda, 1988 | Buda, M., Tsuang, M. T., & Fleming, J. A. (1988). Causes of Death in DSM-III Schizophrenics and Other Psychotics (Atypical Group): A Comparison With the General Population. *Archives of General Psychiatry, 45*(3), 283-285. |
| Callaghan, 2014 | Callaghan, R. C., Veldhuizen, S., Jeysingh, T., Orlan, C., Graham, C., Kakouris, G., et al. (2014). Patterns of tobacco-related mortality among individuals diagnosed with schizophrenia, bipolar disorder, or depression. *J Psychiatr Res, 48*(1), 102-110. |
| Casadebaig, 1999 | Casadebaig, F., & Philippe, A. (1999). Mortality among schizophrenic patients. *Encephale-Revue De Psychiatrie Clinique Biologique Et Therapeutique, 25*(4), 329-337. |
| Castagnini, 2013 | Castagnini, A., Foldager, L., & Bertelsen, A. (2013). Excess mortality of acute and transient psychotic disorders: comparison with bipolar affective disorder and schizophrenia. *Acta Psychiatr Scand, 128*(5), 370-375. |
| Chan, 2021 | Chan, J. K. N., Wong, C. S. M., Yung, N. C. L., Chen, E. Y. H., & Chang, W. C. (2021). Excess mortality and life-years lost in people with bipolar disorder: an 11-year population-based cohort study. *Epidemiology and psychiatric sciences, 30*, e39. |
| Crump, 2013a | Crump, C., Sundquist, K., & Sundquist, J. (2013). Comorbidities and mortality in persons with schizophrenia: a Swedish national cohort study. *Am J Psychiatr, 170*, 324 - 333. |
| Crump, 2013b | Crump, C., Sundquist, K., Winkleby, M. A., & Sundquist, J. (2013). Comorbidities and mortality in bipolar disorder: A swedish national cohort study. *JAMA Psychiatry, 70*(9), 931-939. |
| Cunningham, 2014* | Cunningham, R., Peterson, D., Sarfati, D., Stanley, J., & Collings, S. (2014). Premature mortality in adults using New Zealand psychiatric services. *New Zealand Medical Journal, 127*(1394), 31-41. |
| Curkendall, 2004 | Curkendall, S. M., Mo, J., Glasser, D. B., Rose Stang, M., & Jones, J. K. (2004). Cardiovascular disease in patients with schizophrenia in Saskatchewan, Canada. *J Clin Psychiatry, 65*(5), 715-720. |
| Dalgard, 1966 | Dalgard, O. S. (1966). [Mortality in functional psychosis]. *Nord Med, 75*(24), 680-684. |
| Das-Munshi, 2017 | Das-Munshi, J., Chang, C. K., Dutta, R., Morgan, C., Nazroo, J., Stewart, R., & Prince, M. J. (2017). Ethnicity and excess mortality in severe mental illness: a cohort study. *The Lancet Psychiatry, 4*(5), 389-399. |
| Daumit, 2010 | Daumit, G. L., Anthony, C. B., Ford, D. E., Fahey, M., Skinner, E. A., Lehman, A. F., et al. (2010). Pattern of mortality in a sample of Maryland residents with severe mental illness. *Psychiatry Research, 176*(2-3), 242-245. |
| Dutta, 2012 | Dutta, R., Murray, R., Allardyce, J., Jones, P., & Boydell, J. (2012). Mortality in first-contact psychosis patients in the UK: a cohort study. *Psychol Med, 42*(8), 1649 - 1661. |
| Fors, 2007 | Fors, B. M., Isacson, D., Bingefors, K., & Widerlov, B. (2007). Mortality among persons with schizophrenia in Sweden: An epidemiological study. *Nordic Journal of Psychiatry, 61*(4), 252-259. |
| Giel, 1978 | Giel, R., Dijk, S., & van Weerden-Dijkstra, J. R. (1978). Mortality in the long-stay population of all Dutch mental hospitals. *Acta Psychiatr Scand, 57*(5), 361-368. |
| Girardi, 2021 | Girardi, P., Schievano, E., Fedeli, U., Braggion, M., Nuti, M., & Amaddeo, F. (2021). Causes of mortality in a large population-based cohort of psychiatric patients in Southern Europe. *Journal of psychiatric research, 136*, 167-172. |
| Grigoletti, 2009 | Grigoletti, L., Perini, G., Rossi, A., Biggeri, A., Barbui, C., Tansella, M., & Amaddeo, F. (2009). Mortality and cause of death among psychiatric patients: a 20-year case-register study in an area with a community-based system of care. *Psychological medicine, 39*(11), 1875-1884. |
| Hansen, 2001 | Hansen, V., Jacobsen, B. K., & Arnesen, E. (2001). Cause-specific mortality in psychiatric patients after deinstitutionalisation. *Br J Psychiatry, 179*, 438-443. |
| Hayes, 2017 | Hayes, J. F., Marston, L., Walters, K., King, M. B., Osborn, D. P., & Osborn, D. P. J. (2017). Mortality gap for people with bipolar disorder and schizophrenia: UK-based cohort study 2000-2014. *British Journal of Psychiatry, 211*(3), 175-181. |
| Heiberg, 2018 | Heiberg, I. H., Jacobsen, B. K., Nesvag, R., Bramness, J. G., Reichborn-Kjennerud, T., Naess, O., et al. (2018). Total and cause-specific standardized mortality ratios in patients with schizophrenia and/or substance use disorder. *PLoS ONE, 13*(8), e0202028. |
| Heila, 2005 | Heilä, H., Haukka, J., Suvisaari, J., & Lönnqvist, J. (2005). Mortality among patients with schizophrenia and reduced psychiatric hospital care. *Psychological medicine, 35*(5), 725-732. |
| Hiroeh, 2008 | Hiroeh, U., Kapur, N., Webb, R., Dunn, G., Mortensen, P. B., & Appleby, L. (2008). Deaths from natural causes in people with mental illness: a cohort study. *Journal of Psychosomatic Research, 64*(3), 275-283. |
| Hoang, 2011 | Hoang, U., Stewart, R., & Goldacre, M. J. (2011). Mortality after hospital discharge for people with schizophrenia or bipolar disorder: retrospective study of linked English hospital episode statistics, 1999-2006. *BMJ, 343*, d5422. |
| Høye, 2011 | Høye, A., Jacobsen, B. K., & Hansen, V. (2011). Increasing mortality in schizophrenia: are women at particular risk? A follow-up of 1111 patients admitted during 1980-2006 in Northern Norway. *Schizophrenia Research, 132*(2-3), 228-232. |
| John, 2018 | John, A., McGregor, J., Jones, I., Lee, S. C., Walters, J. T. R., Owen, M. J., . . . Lloyd, K. (2018). Premature mortality among people with severe mental illness - New evidence from linked primary care data. *SCHIZOPHRENIA RESEARCH, 199*, 154-162. |
| Kelly, 2010 | Kelly, D. L., McMahon, R. P., Liu, F., Love, R. C., Wehring, H. J., Shim, J. C., et al. (2010). Cardiovascular disease mortality in patients with chronic schizophrenia treated with clozapine: A retrospective cohort study. *Journal of Clinical Psychiatry, 71*(3), 304-311. |
| Kilbourne, 2009 | Kilbourne, A. M., Morden, N. E., Austin, K., Ilgen, M., McCarthy, J. F., Dalack, G., & Blow, F. C. (2009). Excess heart-disease-related mortality in a national study of patients with mental disorders: Identifying modifiable risk factors. *Gen Hosp Psychiatry, 31*(6), 555-563. |
| Kiviniemi, 2010 | Kiviniemi, M., Suvisaari, J., Pirkola, S., Hakkinen, U., Isohanni, M., & Hakko, H. (2010). Regional differences in five-year mortality after a first episode of schizophrenia in Finland. *Psychiatr Serv, 61*(3), 272-279. |
| Kredentser, 2014* | Kredentser, M. S., Martens, P. J., Chochinov, H. M., & Prior, H. J. (2014). Cause and rate of death in people with schizophrenia across the lifespan: A population-based study in Manitoba, Canada. *Journal of Clinical Psychiatry, 75*(2), 154-160. |
| Lahti, 2012 | Lahti, M., Tiihonen, J., Wildgust, H., Beary, M., Hodgson, R., Kajantie, E., et al. (2012). Cardiovascular morbidity, mortality and pharmacotherapy in patients with schizophrenia. *Psychological medicine, 42*(11), 2275-2285. |
| Laursen, 2007 | Laursen, T. M., Munk-Olsen, T., Nordentoft, M., & Mortensen, P. B. (2007). Increased mortality among patients admitted with major psychiatric disorders: A register-based study comparing mortality in unipolar depressive disorder, bipolar affective disorder, schizoaffective disorder, and schizophrenia. *Journal of Clinical Psychiatry, 68*(6), 899-907. |
| Laursen, 2011 | Laursen, T. M., & Nordentoft, M. (2011). Heart disease treatment and mortality in schizophrenia and bipolar disorder-Changes in the Danish population between 1994 and 2006. *Journal of Psychiatric Research, 45*(1), 29-35. |
| Laursen, 2013 | Laursen, T. M., Wahlbeck, K., Hallgren, J., Westman, J., Ösby, U., Alinaghizadeh, H., et al. (2013). Life expectancy and death by diseases of the circulatory system in patients with bipolar disorder or schizophrenia in the Nordic countries. *PLoS ONE, 8*(6), e67133. |
| Laursen, 2014 | Laursen, T. M., Mortensen, P. B., MacCabe, J. H., Cohen, D., & Gasse, C. (2014). Cardiovascular drug use and mortality in patients with schizophrenia or bipolar disorder: a Danish population-based study. *Psychological Medicine, 44*(8), 1625-1637. |
| Laursen, 2019 | Laursen, T. M., Plana-Ripoll, O., Andersen, P. K., McGrath, J. J., Toender, A., Nordentoft, M., . . . Erlangsen, A. (2019). Cause-specific life years lost among persons diagnosed with schizophrenia: Is it getting better or worse? *Schizophrenia research, 206*, 284-290. |
| Lawrence, 2003 | Lawrence, D. M., Holman, C. D. a. J., Jablensky, A. V., & Hobbs, M. S. T. (2003). Death rate from ischaemic heart disease in Western Australian psychiatric patients 1980-1998. *Br J Psychiatry, 182*, 31-36. |
| Lawrence, 2013* | Lawrence, D., Hancock, K., & Kisely, S. (2013). The gap in life expectancy from preventable physical illness in psychiatric patients in Western Australia: retrospective analysis of population based registers. *BMJ, 346*, f2539. |
| Lemogne, 2013 | Lemogne, C., Nabi, H., Melchior, M., Goldberg, M., Limosin, F., Consoli, S. M., & Zins, M. (2013). Mortality associated with depression as compared with other severe mental disorders: a 20-year follow-up study of the GAZEL cohort. *Journal of Psychiatric Research, 47*(7), 851-857. |
| Lesage, 2015** | Lesage A, Rochette L, Émond V, Pelletier É, St-Laurent D, Diallo FB, et al. (2015). A Surveillance System to Monitor Excess Mortality of People With Mental Illness in Canada. *Canadian Journal of Psychiatry, 60*(12), 571-579. |
| Lumme, 2016* | Lumme, S., Pirkola, S., Manderbacka, K., & Keskimaki, I. (2016). Excess Mortality in Patients with Severe Mental Disorders in 1996-2010 in Finland. *PLoS ONE, 11*(3), e0152223. |
| Manderbacka, 2012 | Manderbacka K, Arffman M, Sund R, Haukka J, Keskimäki I, Wahlbeck K. (2012). How does a history of psychiatric hospital care influence access to coronary care: a cohort study. *BMJ Open, 2*(2), e000831. |
| Morden, 2012 | Morden, N. E., Lai, Z., Goodrich, D. E., MacKenzie, T., McCarthy, J. F., Austin, K., et al. M. (2012). Eight-year trends of cardiometabolic morbidity and mortality in patients with schizophrenia. *Gen Hosp Psychiatry, 34*(4), 368-379. |
| Mortensen, 1990 | Mortensen, P. B., & Juel, K. (1990). Mortality and causes of death in schizophrenic patients in Denmark. *Acta Psychiatr Scand, 81*(4), 372-377. |
| Mortensen, 1993 | Mortensen, P. B., & Juel, K. (1993). Mortality and causes of death in first admitted schizophrenic patients. *British Journal of Psychiatry, 163*(AUG.), 183-189. |
| Murray-Thomas, 2013 | Murray-Thomas, T., Jones, M. E., Patel, D., Brunner, E., Shatapathy, C. C., Motsko, S., & Van Staa, T. P. (2013). Risk of mortality (including sudden cardiac death) and major cardiovascular events in atypical and typical antipsychotic users: a study with the general practice research database. *Cardiovascular Psychiatry & Neurology*, 247486-247486. |
| Newman, 1991 | Newman, S. C., & Bland, R. C. (1991). Mortality in a cohort of patients with schizophrenia: a record linkage study. *Canadian Journal of Psychiatry - Revue Canadienne de Psychiatrie, 36*(4), 239-245. |
| Nilsson, 1995 | Nilsson, A. (1995). MORTALITY IN RECURRENT MOOD DISORDERS DURING PERIODS ON AND OFF LITHIUM - A COMPLETE POPULATION STUDY IN 362 PATIENTS. *Pharmacopsychiatry, 28*(1), 8-13. |
| Nordentoft, 2013 | Nordentoft, M., Wahlbeck, K., Hallgren, J., Westman, J., Ösby, U., Alinaghizadeh, H., et al. (2013). Excess mortality, causes of death and life expectancy in 270,770 patients with recent onset of mental disorders in Denmark, Finland and Sweden. *PLoS ONE, 8*(1), e55176. |
| Odegard, 1967 | Odegard, O. (1967). Mortality in Norwegian psychiatric hospitals 1950-1962. *Acta genetica et statistica medica, 17*(1), 137-153. |
| Olfson, 2015 | Olfson, M., Gerhard, T., Huang, C., Crystal, S., & Stroup, T. S. (2015). Premature Mortality Among Adults With Schizophrenia in the United States. *JAMA Psychiatry, 72*(12), 1172-1181. |
| Osborn, 2007 | Osborn, D., Levy, G., Nazareth, I., Petersen, I., Islam, A., & King, M. (2007). Relative risk of cardiovascular and cancer mortality in people with severe mental illness from the United Kingdom's general practice research database. *Arch Gen Psychiatr, 64*(2), 242 - 249. |
| Ösby, 2000a | Ösby, U., Correia, N., Brandt, L., Ekbom, A., & Sparen, P. (2000). Mortality and causes of death in schizophrenia in Stockholm county, Sweden. *Schizophrenia Research, 45*(1-2), 21-28. |
| Ösby, 2000b | Ösby, U., Correia, N., Brandt, L., Ekbom, A., & Sparen, P. (2000). Time trends in schizophrenia mortality in Stockholm County, Sweden: Cohort study. *British Medical Journal, 321*(7259), 483-484. |
| Ösby, 2001 | Ösby, U., Brandt, L., Correia, N., Ekbom, A., & Sparen, P. (2001). Excess mortality in bipolar and unipolar disorder in Sweden. *Archives of General Psychiatry, 58*(9), 844-850. |
| Ösby, 2016 | Ösby, U., Westman, J., Hallgren, J., & Gissler, M. (2016). Mortality trends in cardiovascular causes in schizophrenia, bipolar and unipolar mood disorder in Sweden 1987-2010. *Eur J Public Health*. |
| Pan, 2020 | Pan, Y.-J., Yeh, L.-L., Chan, H.-Y., & Chang, C.-K. (2020). Excess mortality and shortened life expectancy in people with major mental illnesses in Taiwan. *Epidemiology and psychiatric sciences, 29*, e156. |
| Park, 2015 | Park, S., Kim, S. Y., & Hong, J. P. (2015). Cause-specific mortality of psychiatric inpatients and outpatients in a general hospital in Korea. *Asia Pac J Public Health, 27*(2), 164-175. |
| Prior, 1996 | Prior, P., Hassall, C., & Cross, K. W. (1996). Causes of death associated with psychiatric illness. *Journal of Public Health Medicine, 18*(4), 381-389. |
| Saku, 1995 | Saku, M., Tokudome, S., Ikeda, M., Kono, S., Makimoto, K., Uchimura, H., et al. (1995). Mortality in psychiatric patients, with a specific focus on cancer mortality associated with schizophrenia. *International Journal of Epidemiology, 24*(2), 366-372. |
| Salazar-Fraile, 1998 | Salazar-Fraile, J., Gomez-Beneyto, M., Perez-Hovos, S., & Hurtado-Navarro, I. (1998). Mortality among psychiatric patients referred to the mental health services in Valencia. *Soc Psychiatry Psychiatr Epidemiol, 33*(5), 224-229. |
| Sanchez, 2021 | Sanchez, M. C., Escurriola, M. F., Sanmartin, M. I. F., Solntseva, I., Baquero, D. B., & Arno, A. G. (2021). Cardiovascular disease and mortality in people with schizophrenia or antipsychotic treatment: A cohort study in primary care. *Psychiatry Research, 306*, 114233 |
| Tanskanen, 2018 | Tanskanen, A., Tiihonen, J., & Taipale, H. (2018). Mortality in schizophrenia: 30-year nationwide follow-up study. *Acta Psychiatr Scand, 138*(6), 492-499. |
| Termorshuizen, 2013 | Termorshuizen, F., Wierdsma, A. I., Smeets, H. M., Visser, E., Drukker, M., Nijman, H., & Sytema, S. (2013). Cause-Specific Mortality Among Patients With Psychosis: Disentangling the Effects of Age and Illness Duration. *Psychosomatics, 54*(6), 536-545. |
| Torniainen, 2015 | Torniainen, M., Mittendorfer-Rutz, E., Tanskanen, A., Bjorkenstam, C., Suvisaari, J., Alexanderson, K., & Tiihonen, J. (2015). Antipsychotic treatment and mortality in schizophrenia. *Schizophr Bull, 41*(3), 656-663. |
| Tsuang, 1980 | Tsuang, M. T., Woolson, R. F., & Fleming, J. A. (1980). Causes of death in schizophrenia and manic-depression. *British Journal of Psychiatry, 136*, 239-242. |
| Vance, 2019 | Vance, M. C., Wiitala, W. L., Sussman, J. B., Pfeiffer, P., & Hayward, R. A. (2019). Increased Cardiovascular Disease Risk in Veterans With Mental Illness. *Circulation, 12*(10), e005563 |
| Weeke, 1986 | Weeke, A., & Vaeth, M. (1986). EXCESS MORTALITY OF BIPOLAR AND UNIPOLAR MANIC-DEPRESSIVE PATIENTS. *Journal of Affective Disorders, 11*(3), 227-234. |
| Weeke, 1987 | Weeke, A., Juel, K., & Vaeth, M. (1987). Cardiovascular death and manic-depressive psychosis. *Journal of Affective Disorders, 13*(3), 287-292. |
| Westman, 2013 | Westman, J., Hällgren, J., Wahlbeck, K., Erlinge, D., Alfredsson, L., & Ösby, U. (2013). Cardiovascular mortality in bipolar disorder: a population-based cohort study in Sweden. *BMJ Open, 3*(4). |
| Westman, 2017 | Westman, J., Eriksson, S. V., Gissler, M., Hallgren, J., Prieto, M. L., Bobo, W. V., et al. (2018). Increased cardiovascular mortality in people with schizophrenia: a 24-year national register study. *Epidemiology and psychiatric sciences, 27*(5), 519-527. |
| Yung, 2021 | Yung, N. C. L., Wong, C. S. M., Chan, J. K. N., Chen, E. Y. H., & Chang, W. C. (2021). Excess Mortality and Life-Years Lost in People with Schizophrenia and Other Non-affective Psychoses: An 11-Year Population-Based Cohort Study. *Schizophrenia Bulletin, 47*(2), 474-484. |
| Zilber, 1989 | Zilber, N., Schufman, N., & Lerner, Y. (1989). Mortality among psychiatric patients--the groups at risk. *Acta Psychiatr Scand, 79*(3), 248-256. |
| * extra data kindly supplied by paper’s corresponding author  ** extra data kindly supplied by paper’s second author | |
