# Supplementary material for: Temporal trends in associations between severe mental illness and risk of cardiovascular disease: A systematic review and meta-analysis
Source: PLoS Med. 2022 Apr 19;19(4):e1003960. doi: 10.1371/journal.pmed.1003960 (PMC9017899; doi:10.1371/journal.pmed.1003960)
Supplement: S10 File — (DOCX) [file pmed.1003960.s010.docx]

# S10 File. List of included incidence studies

| **Study ID** | **Article** |
| --- | --- |
| Bent-Ennakhil, 2018 | Bent-Ennakhil, N., Cecile Perier, M., Sobocki, P., Gothefors, D., Johansson, G., Milea, D., & Empana, J. P. (2018). Incidence of cardiovascular diseases and type-2-diabetes mellitus in patients with psychiatric disorders. *Nordic Journal of Psychiatry, 72*(7), 455-461. |
| Brink, 2018 | Brink, M., Green, A., Bojesen, A. B., Lamberti, J. S., Conwell, Y., & Andersen, K. (2018). Excess medical comorbidity and mortality across the lifespan in schizophrenia.: A nationwide Danish register study. *Schizophrenia Research (in press)*. |
| Carney, 2006 | Carney, C. P., Jones, L., & Woolson, R. F. (2006). Medical comorbidity in women and men with schizophrenia: a population-based controlled study. *Journal of general internal medicine, 21*(11), 1133-1137. |
| Chen, 2015 | Mu-Hong, C., Cheng-Ta, L., Ju-Wei, H., Kai-Lin, H., Wei-Chen, L., Wen-Han, C., et al. (2015). Atopic Diseases and Subsequent Ischemic Stroke Among Patients With Schizophrenia: A Nationwide Longitudinal Study. *Psychosomatic Medicine, 77*(9), 1031-1038. |
| Crump, 2013a | Crump, C., Sundquist, K., & Sundquist, J. (2013). Comorbidities and mortality in persons with schizophrenia: a Swedish national cohort study. *Am J Psychiatr, 170*, 324 - 333. |
| Crump, 2013b | Crump, C., Sundquist, K., Winkleby, M. A., & Sundquist, J. (2013). Comorbidities and mortality in bipolar disorder: A swedish national cohort study. *JAMA Psychiatry, 70*(9), 931-939. |
| Curkendall, 2004 | Curkendall, S. M., Mo, J., Glasser, D. B., Rose Stang, M., & Jones, J. K. (2004). Cardiovascular disease in patients with schizophrenia in Saskatchewan, Canada. *The Journal of clinical psychiatry, 65*(5), 715-720. |
| Foroughi, 2018 | Foroughi, M., Medina-Inojosa, J. R., Lopez-Jimenez, F., Saeidifard, F., Viniciguerra, M., Frye, M. A., & Morgan, R. J. (2018). Risk of Atherosclerotic Cardiovascular Disease in Patients With Bipolar Disorder and Accuracy of a Cardiovascular Risk Calculator. *Biological Psychiatry, 83*(9), S283-S283. |
| Foroughi, 2021 | Foroughi, M., Medina Inojosa, J. R., Lopez-Jimenez, F., Saeidifard, F., Suarez, L., Stokin, G. B., et al. (2022). Association of Bipolar Disorder With Major Adverse Cardiovascular Events: A Population-Based Historical Cohort Study. *Psychosomatic Medicine, 84*(1). |
| Gale, 2013 | Gale, C. R., Batty, G. D., Osborn, D. P. J., Tynelius, P., & Rasmussen, F. (2014). Mental disorders across the adult life course and future coronary heart disease: evidence for general susceptibility. *Circulation, 129*(2), 186-193. |
| Goldstein, 2014 | Goldstein, B. I., Schaffer, A., Wang, S., & Blanco, C. (2015). Excessive and premature new-onset cardiovascular disease among adults with bipolar disorder in the US NESARC cohort. *The Journal of clinical psychiatry, 76*(2), 163-169. |
| Gur, 2017 | Gur, S., Weizman, S., Stubbs, B., Matalon, A., Meyerovitch, J., Hermesh, H., & Krivoy, A. (2018). Mortality, morbidity and medical resources utilization of patients with schizophrenia: A case-control community-based study. *Psychiatry research, 260*, 177-181. |
| Hayes, 2017 | Hayes, J. F., Marston, L., Walters, K., King, M. B., Osborn, D. P., & Osborn, D. P. J. (2017). Mortality gap for people with bipolar disorder and schizophrenia: UK-based cohort study 2000-2014. *British Journal of Psychiatry, 211*(3), 175-181. |
| Hsu, 2021 | Hsu, J.-H., Chien, I. C., & Lin, C.-H. (2021). Increased risk of ischemic heart disease in patients with bipolar disorder: A population-based study. *Journal of affective disorders, 281*, 721-726. |
| Jackson, 2020 | Jackson, C. A., Kerssens, J., Fleetwood, K., Smith, D. J., Mercer, S. W., & Wild, S. H. (2020). Incidence of ischaemic heart disease and stroke among people with psychiatric disorders: retrospective cohort study. *British journal of psychiatry, 217*(2), 442-449. |
| Jakobsen, 2008 | Jakobsen, A. H., Foldager, L., Parker, G., & Munk-JÃ¸rgensen, P. (2008). Quantifying links between acute myocardial infarction and depression, anxiety and schizophrenia using case register databases. *Journal of Affective Disorders, 109*(1/2), 177-181. |
| Kessing, 2021 | Kessing, L. V., Ziersen, S. C., Andersen, P. K., & Vinberg, M. (2021). A nation-wide population-based longitudinal study mapping physical diseases in patients with bipolar disorder and their siblings. *Journal of affective disorders, 282*, 18-25. |
| Kugathasan, 2018 | Kugathasan P., Laursen T.M., Grontved S., Jensen S.E., Aagaard J., Nielsen R.E. (2018). Increased long-term mortality after myocardial infarction in patients with schizophrenia. *Schizophrenia Research. 2018*;199:103-8. |
| Lahti, 2012 | Lahti, M., Tiihonen, J., Wildgust, H., Beary, M., Hodgson, R., Kajantie, E., et al. (2012). Cardiovascular morbidity, mortality and pharmacotherapy in patients with schizophrenia. *Psychological Medicine, 42*(11), 2275-2285. |
| Laursen, 2010 | Laursen, T. M., Munk-Olsen, T., & Gasse, C. (2011). Chronic somatic comorbidity and excess mortality due to natural causes in persons with schizophrenia or bipolar affective disorder. *PLoS ONE, 6 (9) (no pagination)*(e24597). |
| Laursen, 2011 | Laursen, T. M., & Nordentoft, M. (2011). Heart disease treatment and mortality in schizophrenia and bipolar disorder-Changes in the Danish population between 1994 and 2006. *Journal of Psychiatric Research, 45*(1), 29-35. |
| Lawrence, 2003 | Lawrence, D. M., Holman, C. D. a. J., Jablensky, A. V., & Hobbs, M. S. T. (2003). Death rate from ischaemic heart disease in Western Australian psychiatric patients 1980-1998. *The British journal of psychiatry : the journal of mental science, 182*, 31-36. |
| Lin, 2010 | Lin, H. C., Chen, Y. H., & Lee, H. C. (2010). Increased risk of acute myocardial infarction after acute episode of schizophrenia: 6 year follow-up study. *Australian and New Zealand Journal of Psychiatry, 44*(3), 273-279. |
| Manderbacka, 2012 | Manderbacka, K., Arffman, M., Sund, R., Haukka, J., Keskimäki, I., Wahlbeck, K. (2012). How does a history of psychiatric hospital care influence access to coronary care: a cohort study. *BMJ Open, 2*(2), e000831. |
| McDermott, 2005 | McDermott, S., Moran, R., Platt, T., Isaac, T., Wood, H., & Dasari, S. (2005). Heart disease, schizophrenia, and affective psychoses: epidemiology of risk in primary care. *Community Mental Health Journal, 41*(6), 747-755. |
| Momen, 2020* | Momen, N. C., Plana-Ripoll, O., Agerbo, E., Benros, M. E., Borglum, A. D., Christensen, M. K., . . . McGrath, J. J. (2020). Association between Mental Disorders and Subsequent Medical Conditions. *The New England journal of medicine, 382*(18), 1721-1731. |
| Morden, 2012 | Morden, N. E., Lai, Z., Goodrich, D. E., MacKenzie, T., McCarthy, J. F., Austin, K., et al. (2012). Eight-year trends of cardiometabolic morbidity and mortality in patients with schizophrenia. *Gen Hosp Psychiatry, 34*(4), 368-379. |
| Munk-Jorgensen, 2000 | Munk-Jorgensen, P., Mors, O., Mortensen, P. B., & Ewald, H. (2000). The schizophrenic patient in the somatic hospital. *Acta Psychiatrica Scandinavica, 102*, 96-99. |
| Prieto, 2016 | Prieto, M. L., Schenck, L. A., Kruse, J. L., Klaas, J. P., Chamberlain, A. M., Bobo, W. V., et al. (2016). Long-term risk of myocardial infarction and stroke in bipolar I disorder: A population-based Cohort Study. *Journal of Affective Disorders, 194*, 120-127. |
| Ramsey, 2010 | Ramsey, C. M., Leoutsakos, J. M., Mayer, L. S., Eaton, W. W., Lee, H. B., Ramsey, C. M., et al. (2010). History of manic and hypomanic episodes and risk of incident cardiovascular disease: 11.5 year follow-up from the Baltimore Epidemiologic Catchment Area Study. *Journal of Affective Disorders, 125*(1-3), 35-41. |
| Sanchez, 2021 | Sanchez, M. C., Escurriola, M. F., Sanmartin, M. I. F., Solntseva, I., Baquero, D. B., & Arno, A. G. (2021). Cardiovascular disease and mortality in people with schizophrenia or antipsychotic treatment: A cohort study in primary care. *Psychiatry Research, 306*, 114233 |
| Sundquist, 2006 | Sundquist, K., & Li, X. (2006). Alcohol abuse partly mediates the association between coronary heart disease and affective or psychotic disorders: a follow-up study in Sweden. *Acta Psychiatrica Scandinavica, 113*(4), 283-289. |
| Tsai, 2012 | Tsai, K. Y., Lee, C. C., Chou, Y. M., Su, C. Y., Chou, F. H., Tsai, K.-Y., et al. (2012). The incidence and relative risk of stroke in patients with schizophrenia: a five-year follow-up study. *Schizophrenia Research, 138*(1), 41-47. |
| Vance, 2019 | Vance, M. C., Wiitala, W. L., Sussman, J. B., Pfeiffer, P., & Hayward, R. A. (2019). Increased Cardiovascular Disease Risk in Veterans With Mental Illness. *Circulation, 12*(10), e005563 |
| Westman, 2013 | Westman, J., Eriksson, S. V., Gissler, M., Hallgren, J., Prieto, M. L., Bobo, W. V., et al. (2018). Increased cardiovascular mortality in people with schizophrenia: a 24-year national register study. *Epidemiology and psychiatric sciences, 27*(5), 519-527. |
| Westman, 2017 | Westman, J., Hällgren, J., Wahlbeck, K., Erlinge, D., Alfredsson, L., & Ösby, U. (2013). Cardiovascular mortality in bipolar disorder: a population-based cohort study in Sweden. *BMJ Open, 3*(4). |
| Wium-Andersen, 2021 | (2021). An analysis of the relative and absolute incidence of somatic morbidity in patients with affective disorders-A nationwide cohort study. *Journal of Affective Disorders, 292*, 204-211. |
| Wu, 2013 | Wu, H. C., Chou, F. H. C., Tsai, K. Y., Su, C. Y., Shen, S. P., & Chung, T. C. (2013). The Incidence and Relative Risk of Stroke among Patients with Bipolar Disorder: A Seven-Year Follow-Up Study. *PLoS ONE, 8*(8), e73037. |
| Wu, 2015 | Wu, S. I., Chen, S. C., Liu, S. I., Sun, F. J., Juang, J. J., Lee, H. C., et al. (2015). Relative Risk of Acute Myocardial Infarction in People with Schizophrenia and Bipolar Disorder: A Population-Based Cohort Study. *PLoS ONE, 10*(8), e0134763. |
| Yu-Chuan Chiu, 2015 | Yu-Chuan, C., Ya-Mei, B., Tung-Ping, S., Tzeng-Ji, C., Mu-Hong, C., Chiu, Y.-C., et al. (2015). Ischemic Stroke in Young Adults and Preexisting Psychiatric Disorders: A Nationwide Case-Control Study. *Medicine, 94*(38), 1-6 |

* extra data kindly supplied by paper’s corresponding author
