# Supplementary material for: Temporal trends in associations between severe mental illness and risk of cardiovascular disease: A systematic review and meta-analysis
Source: PLoS Med. 2022 Apr 19;19(4):e1003960. doi: 10.1371/journal.pmed.1003960 (PMC9017899; doi:10.1371/journal.pmed.1003960)
Supplement: S11 File — (DOCX) [file pmed.1003960.s011.docx]

# S11 File. List of excluded studies

| **Article** | **Reason *** | |  |
| --- | --- | --- | --- |
| Ahrens, B., Grof, P., Moller, H. J., Muller-Oerlinghausen, B., & Wolf, T. (1995). Extended survival of patients on long-term lithium treatment. *Canadian Journal of Psychiatry - Revue Canadienne de Psychiatrie, 40*(5), 241-246. | 5 | |  |
| Altamura, C., Fagiolini, A., Galderisi, S., Rocca, P., & Rossi, A. (2014). Schizophrenia today: Epidemiology, diagnosis, course and models of care. *Journal of Psychopathology, 20*(3), 223-243. | 8 | |  |
| Angst, J., Hengartner, M. P., Gamma, A., von Zerssen, D., & Angst, F. (2013). Mortality of 403 patients with mood disorders 48 to 52 years after their psychiatric hospitalisation. *European Archives of Psychiatry and Clinical Neuroscience, 263*(5), 425-434. | 5 | |  |
| Anonymous. (2004). Olanzapine and cerebrovascular events. *WHO Drug Information, 18*(1), 32. | 8 | |  |
| Aslan, M., Radhakrishnan, K., Rajeevan, N., Sueiro, M., Goulet, J. L., Li, Y., et al. (2020). Suicidal ideation, behavior, and mortality in male and female US veterans with severe mental illness. *J. Affect. Disord., 267*, 144-152. | 9 | |  |
| Attar, R., Jensen, S. E., Nielsen, R. E., Polcwiartek, C., Andell, P., Pedersen, C. T., et al. (2020). Time Trends in the Use of Coronary Procedures, Guideline-Based Therapy, and All-Cause Mortality following the Acute Coronary Syndrome in Patients with Schizophrenia. *Cardiology, 145*(7), 401-409. | 3 | |  |
| Attar, R., Koul, S., Erlinge, D., Andell, P., & Jensen, S. E. (2019). Characteristics and outcomes following myocardial infarction in patients with schizophrenia. *Eur. Heart J., 40*(Supplement 1), 2231. | 3 | |  |
| Attar, R., Valentin, J. B., Freeman, P., Andell, P., Aagaard, J., & Jensen, S. E. (2019). The effect of schizophrenia on major adverse cardiac events, length of hospital stay, and prevalence of somatic comorbidities following acute coronary syndrome. *European heart journal. Quality of care & clinical outcomes, 5*(2), 121-126. | 3 | |  |
| Auger, N., Potter, B. J., Healy-Profitos, J., He, S., Schnitzer, M. E., & Paradis, G. (2020). Mood disorders in pregnant women and future cardiovascular risk. *J. Affect. Disord., 266*, 128-134. | 2 | |  |
| Azevedo Da Silva, M., Lemogne, C., Melchior, M., Zins, M., Van Der Waerden, J., Consoli, S. M., . . . Nabi, H. (2015). Excess non-psychiatric hospitalizations among employees with mental disorders: a 10-year prospective study of the GAZEL cohort. *Acta Psychiatrica Scandinavica, 131*(4), 307-317. | 2 | |  |
| Bahorik, A. L., Satre, D. D., Kline-Simon, A. H., Weisner, C. M., & Campbell, C. I. (2017). Serious mental illness and medical comorbidities: Findings from an integrated health care system. *Journal of Psychosomatic Research, 100*, 35-45. | 1 | |  |
| Baldwin, J. (1987). Schizophrenia and physical disease: a preliminary analysis of the data from the Oxford Record Linkage Study. In J. Baldwin, E. Acheson, & W. Graham (Eds.), *Textbook of Medical Record Linkage* (pp. 133-157). Oxford: Oxford University Press. | 11 | |  |
| Marche, J. C., Bannay, A., Baillot, S., Dauriac-Le Masson, V., Leveque, P., Schmitt, C., et al. (2021). Prevalence of severe cardiovascular disease in patients with schizophrenia. *L'Encéphale*. | 11 | |  |
| Barcella, C. A., Mohr, G. H., Christensen, D. M., Wissenberg, M., Gislason, G. H., Soendergaard, K. B., et al. (2019). Increased risk of out-of-hospital cardiac arrest associated with psychiatric disorders. *Eur. Heart J., 40*(Supplement 1), 1349. | 2 | |  |
| Baxter, D. N. (1996). The mortality experience of individuals on the Salford Psychiatric Case Register. I. All-cause mortality. *British Journal of Psychiatry, 168*(6), 772-779. | 10 | |  |
| Bent-Ennakhil, N., Perier, M. C., Sobocki, P., Johansson, G., Milea, D., & Empana, J. P. (2013). Burden of diabetes and cardiovascular diseases in schizophrenia and bipolar patients: Analysis of health registers in Sweden. *European Neuropsychopharmacology, 23*, S498-S499. | 9 | |  |
| Berardi, D., Stivanello, E., Chierzi, F., Musti, M. A., Perlangeli, V., Pandolfi, P., et al. (2021). Mortality in mental health patients of the Emilia-Romagna region of Italy: A registry-based study. *Psychiatry Res., 296*, 113702. | 2 | |  |
| Beresewicz, M., Bogdanowicz, E., Kalinowski, A., Bidzinska, E., Zaluska, M., Parnowski, T., et al. (1986). [Causes of death of persons with diagnosed affective disorders]. *Przyczyny zgonu osob z rozpoznaniem chorob afektywnych., 20*(3), 190-195. | 6 | |  |
| Berren, M. R., Hill, K. R., Merikle, E., Gonzalez, N., & Santiago, J. (1994). Serious mental illness and mortality rates. *Hospital and Community Psychiatry, 45*(6), 604-605. | 4 | |  |
| Berrocal-Izquierdo, N., Bioque, M., & Bernardo, M. (2018). Relationship between antipsychotic medications and cerebrovascular disease in patients with serious mental illness. *Journal of Psychiatric Practice, 24*(2), 72-78. | 4 | |  |
| Black, D. W., & Fisher, R. (1992). Mortality in DSM-IIIR schizophrenia. *Schizophrenia Research, 7*(2), 109-116. | 10 | |  |
| Black, D. W., Warrack, G., & Winokur, G. (1985). Excess mortality among psychiatric patients. The Iowa Record-Linkage Study. *JAMA, 253*(1), 58-61. | 10 | |  |
| Black, D. W., Warrack, G., & Winokur, G. (1985). The Iowa record-linkage study. II. Excess mortality among patients with organic mental disorders. *Archives of General Psychiatry, 42*(1), 78-81. | 10 | |  |
| Black, D. W., Warrack, G., & Winokur, G. (1985). The Iowa record-linkage study. III. Excess mortality among patients with 'functional' disorders. *Archives of General Psychiatry, 42*(1), 82-88. | 3 | |  |
| Black, D. W., Winokur, G., & Nasrallah, A. (1987). Is death from natural causes still excessive in psychiatric patients? A follow-up of 1593 patients with major affective disorder. *Journal of Nervous and Mental Disease, 175*(11), 674-680. | 3 | |  |
| Black, D. W., Winokur, G., & Nasrallah, A. (1987). Mortality in patients with primary unipolar depression, secondary unipolar depression, and bipolar affective disorder: a comparison with general population mortality. *International Journal of Psychiatry in Medicine, 17*(4), 351-360. | 3 | |  |
| Bralet, M. C., Yon, V., Loas, G., & Noisette, C. (2000). Mortality in schizophrenia: a 8-year follow-up study in 150 chronic schizophrenics. *Encephale-Revue De Psychiatrie Clinique Biologique Et Therapeutique, 26*(6), 32-41. | 11 | |  |
| Bresee, L., Tonelli, M., Manns, B., & Hemmelgarn, B. (2012). Temporal trends in incidence of acute myocardial infarction and revascularization in people with and without mental illness. *Circulation, 125*(10 SUPPL. 1). | 1 | |  |
| Bresee, L. C., Majumdar, S. R., Patten, S. B., & Johnson, J. A. (2011). Diabetes, cardiovascular disease, and health care use in people with and without schizophrenia. *European psychiatry : the journal of the Association of European Psychiatrists, 26*(5), 327-332. | 1 | |  |
| Bresee, L. C., Majumdar, S. R., Patten, S. B., & Johnson, J. A. (2012). Utilization of general and specialized cardiac care by people with schizophrenia. *Psychiatric Services, 63*(3), 237-242. | 1 | |  |
| Bresee, L. C., Majumdar, S. R., Patten, S. B., Johnson, J. A., Bresee, L. C., Majumdar, S. R., . . . Johnson, J. A. (2010). Prevalence of cardiovascular risk factors and disease in people with schizophrenia: a population-based study. *Schizophrenia Research, 117*(1), 75-82. | 1 | |  |
| Brown, S., Inskip, H., & Barraclough, B. (2000). Causes of the excess mortality of schizophrenia. *British Journal of Psychiatry, 177*, 212-217. | 5 | |  |
| Brown, S., & Mitchell, C. (2012). Predictors of death from natural causes in schizophrenia: 10-year follow-up of a community cohort. *Social Psychiatry and Psychiatric Epidemiology, 47*(6), 843-847. | 9 | |  |
| Bryant, L. R., Eiseman, B., Spencer, F. C., & Lieber, A. (1965). FREQUENCY OF EXTRACRANIAL CEREBROVASCULAR DISEASE IN PATIENTS WITH CHRONIC PSYCHOSIS. *The New England journal of medicine, 272*, 12-17. | 9 | |  |
| Cabassa, L., Lewis-Fernández, R., Wang, S., Blanco, C., Cabassa, L.J., Lewis-Fernández, R. (2017). Cardiovascular disease and psychiatric disorders among Latinos in the United States. *Social Psychiatry and Psychiatric Epidemiology, 52*(7), 837-846. | 1 | |  |
| Callaghan, R.C., Boire, M.D., Lazo, R.G., McKenzie, K., Cohn, T., Callaghan, R.C., et al. (2009). Schizophrenia and the incidence of cardiovascular morbidity: a population-based longitudinal study in Ontario, Canada. *Schizophrenia Research, 115*(2/3), 325-332. | 3 | |  |
| Callaghan, R. C., & Khizar, A. (2010). The incidence of cardiovascular morbidity among patients with bipolar disorder: a population-based longitudinal study in Ontario, Canada. *Journal of Affective Disorders, 122*(1/2), 118-123. | 3 | |  |
| Capasso, R. M., Lineberry, T. W., Bostwick, J. M., Decker, P. A., & St Sauver, J. (2008). Mortality in schizophrenia and schizoaffective disorder: an Olmsted County, Minnesota cohort: 1950-2005. *Schizophrenia Research, 98*(1-3), 287-294. | 3 | |  |
| Casadebaig, F., & Quemada, N. (1991). Changes in mortality among psychiatric inpatients, 1968-1982. *Social Psychiatry and Psychiatric Epidemiology, 26*(2), 78-82. | 3 | |  |
| Chang, C. K., Hayes, R. D., Broadbent, M., Fernandes, A. C., Lee, W., Hotopf, M., & Stewart, R. (2010). All-cause mortality among people with serious mental illness (SMI), substance use disorders, and depressive disorders in southeast London: A cohort study. *BMC Psychiatry, 10* (77). | 3 | |  |
| Castilho, J. L., Rebeiro, P. F., Shepherd, B. E., Nash, R., Adams, R. S., Turner, M., et al. (2020). Mood Disorders and Increased Risk of Noncommunicable Disease in Adults With HIV. *JAIDS-JOURNAL OF ACQUIRED IMMUNE DEFICIENCY SYNDROMES, 83*(4), 397-404. | 2 | |  |
| Chak Lam Yung, N., Kwun Nam Chan, J., Sau Man Wong, C., Chi Fai Or, P., & Wing Chung, C. (2020). MORTALITY IN PATIENTS WITH SCHIZOPHRENIA ADMITTED FOR INCIDENT ISCHEMIC STROKE: A POPULATION-BASED COHORT STUDY...Schizophrenia International Research Society (SIRS) 2020 Congress. *Schizophr. Bull., 46*, S261-S261. | 3 | |  |
| Chan, J. K. N., Wong, C. S. M., Or, P. C. F., Chen, E. Y. H., & Chang, W. C. (2021a). Diabetes complication burden and patterns and risk of mortality in people with schizophrenia and diabetes: A population-based cohort study with 16-year follow-up. *Eur. Neuropsychopharmacol., 53*, 79-88. | 2 | |  |
| Chan, J. K. N., Wong, C. S. M., Or, P. C. F., Chen, E. Y. H., & Chang, W. C. (2021b). Risk of mortality and complications in patients with schizophrenia and diabetes mellitus: Population-based cohort study. *British Journal of Psychiatry, 219*(1), 375-382. | 2 | |  |
| Chang, C.K., Hayes, R.D., Perera, G., Broadbent, M.T., Fernandes, A.C., Lee, W.E., et al. (2011). Life expectancy at birth for people with serious mental illness and other major disorders from a secondary mental health care case register in London. *PLoS ONE, 6*. | 3 | |  |
| Chen, P.-H., Tsai, S.-Y., Kuo, C.-J., Chung, K.-H., Huang, S.-H., & Chen, C.-C. (2016). Physiological characteristics of patients with schizophrenia prematurely dying from circulatory diseases. *Asia-Pacific psychiatry : official journal of the Pacific Rim College of Psychiatrists, 8*(3), 199-205. | 3 | |  |
| Chen, W. J., Huang, Y. J., Yeh, L. L., Rin, H., & Hwu, H. G. (1996). Excess mortality of psychiatric inpatients in Taiwan. *Psychiatry Research, 62*(3), 239-250. | 3 | |  |
| Cohen, D., Gasse, C., & Laursen, T. (2012). Premature ageing as a proposed explanation for excess of death in schizophrenia. *Schizophrenia Research, 136*, S122. | 3 | |  |
| Chen, P.-H., Tsai, S.-Y., Chang, H.-M., Chen, Y.-L., Su, S.-S., Kuo, C.-J., et al. (2020). Incidence and risk factors of sudden cardiac death in bipolar disorder across the lifespan. *J. Affect. Disord., 274*, 210-217. | 3 | |  |
| Chen, P.-H., Tsai, S.-Y., Pan, C.-H., Chang, H.-M., Chen, Y.-L., Su, S.-S., et al. (2021). Age Effect on Incidence, Physical, and Psychiatric Comorbidity for Sudden Cardiac Death in Schizophrenia: Effet de l'age sur l'incidence, la comorbidite physique et psychiatrique de la mort cardiaque subite dans la schizophrenie. *Can. J. Psychiatry., 66*(4), 367-375. | 3 | |  |
| Chen, W.-Y., Huang, S.-J., Chang, C.-K., Pan, C.-H., Su, S.-S., Yang, T.-W., et al. (2021). Excess mortality and risk factors for mortality among patients with severe mental disorders receiving home care case management. *Nordic journal of psychiatry, 75*(2), 109-117. | 3 | |  |
| Chen, C., Barnes, J., Schmidt, E. M., Trafton, J., Frayne, S., & Harris, A. H. S. (2019). Patient and Health Care Factors Associated with Long-term Diabetes Complications among Adults with and Without Mental Health and Substance Use Disorders. *JAMA Network Open, 2*(9), e1912060. | 4 | |  |
| Conley, R. R., Shim, J., Kelly, D. L., Feldman, S., Yu, Y., & McMahon, R. P. (2005). Cardiovascular disease in relation to weight in deceased persons with schizophrenia. *Comprehensive Psychiatry, 46*(6), 460-467. | 1 | |  |
| Craig, T. J. (2008). Major psychiatric disorders increase risk of mortality. *Evidence Based Mental Health, 11*(1), 9-9. | 8 | |  |
| Craig, T. J., Ye, Q., & Bromet, E. J. (2006). Mortality among first-admission patients with psychosis. *Comprehensive Psychiatry, 47*(4), 246-251. | 3 | |  |
| Crockett, A., Goldstein, M., & Bushe, C. (2005). How common are cardiovascular disease and hypertension in depot clinic outpatients with schizophrenia - The Dewsbury experience. *Journal of Psychopharmacology, 19*(5), A34-A34. | 1 | |  |
| Curkendall, S., Mo, J., Jones, J. K., & Glasser, D. (2001). P.2.074 Increased cardiovascular disease in patients with schizophrenia. *European Neuropsychopharmacology*, S274. | 5 | |  |
| Cunningham, R., Poppe, K., Peterson, D., Every-Palmer, S., Soosay, I., & Jackson, R. (2019). Prediction of cardiovascular disease risk among people with severe mental illness: A cohort study. *PLoS One, 14*(9), e0221521. | 11 | |  |
| Das-Munshi, J., Chang, C. K., Dutta, R., Morgan, C., Nazroo, J., Stewart, R., & Prince, M. (2017). Race/ethnicity and premature mortality in severe mental illness: cohort study. *Schizophrenia Bulletin, 43*, S183. | 5 | |  |
| D'Avanzo, B., La Vecchia, C., & Negri, E. (2003). Mortality in long-stay patients from psychiatric hospitals in Italy - Results from the Qualyop project. *Social Psychiatry and Psychiatric Epidemiology, 38*(7), 385-389. | 4 | |  |
| Davydow, D.S., Ribe, A.R., Pedersen, H.S., Fenger-Grøn, M., Cerimele, J.M., Vedsted, P., et al. (2016). Serious Mental Illness and Risk for Hospitalizations and Rehospitalizations for Ambulatory Care-sensitive Conditions in Denmark: A Nationwide Population-based Cohort Study. *Medical Care, 54*(1), 90-97. | 3 | |  |
| Dembling, B. P., Chen, D. T., & Vachon, L. (1999). Life expectancy and causes of death in a population treated for serious mental illness. *Psychiatric Services, 50*(8), 1036-1042. | 4 | |  |
| de Mooij, L. D., Kikkert, M., Duurkoop, P. W. R. A., Van, H. L., Theunissen, J., Beekman, A. T. F., et al. (2019). Dying Too Soon: Excess Mortality in Severe Mental Illness. *Frontiers in Psychiatry, 10*, 855. | 10 | |  |
| Dickerson, F., Stallings, C., Origoni, A., Schroeder, J., Khushalani, S., & Yolken, R. (2014). Mortality in Schizophrenia: Clinical and serological predictors. *Schizophrenia Bulletin, 40*(4), 796-803. | 3 | |  |
| Dickey, B., Dembling, B., Azeni, H., & Normand, S. L. (2004). Externally caused deaths for adults with substance use and mental disorders. *Journal of Behavioral Health Services and Research, 31*(1), 75-85. | 11 | |  |
| Dobrovolskaia, A., & Sofronov, A. (2015). Assessment of substance abuse on the course of somatic pathology in patients with schizophrenia. *European Psychiatry, 30*, 1759. | 1 | |  |
| Dobrzanski, T., & Bartoszewski, J. (1970). [Painless myocardial infarct in patients with mental disorders]. *Bezbolowy zawal serca u chorych psychicznie., 25*(4), 137-139. | 6 | |  |
| Druss, B. G., Zhao, L., Von Esenwein, S., Morrato, E. H., & Marcus, S. C. (2011). Understanding excess mortality in persons with mental illness: 17-year follow up of a nationally representative US survey. *Medical Care, 49*(6), 599-604. | 10 | |  |
| Dutta, R., Boydell, J., Kennedy, N., Van Os, J., Fearon, P., Murray, R.M. (2007). Suicide and other causes of mortality in bipolar disorder: a longitudinal study. *Psychological Medicine, 37*(6), 839-847. | 5 | |  |
| Dynes, J. B. (1969). Cause of death in schizophrenia. *Behavioral Neuropsychiatry, 1*(2), 12-14. | 6 | |  |
| Eastwood, M. R., Stiasny, S., Meier, H. M., & Woogh, C. M. (1982). Mental illness and mortality. *Comprehensive Psychiatry, 23*(4), 377-385. | 3 | |  |
| Eaton, W.W., Roth, K.B., Bruce, M., Cottler, L., Wu, L., Nestadt, G., et al. (2013). The relationship of mental and behavioral disorders to all-cause mortality in a 27-year follow-up of 4 epidemiologic catchment area samples. *American Journal of Epidemiology, 178*(9), 1366-1377. | 4 | |  |
| Enger, C., Weatherby, L., Reynolds, R. F., Glasser, D. B., & Walker, A. M. (2004). Serious cardiovascular events and mortality among patients with schizophrenia. *Journal of Nervous and Mental Disease, 192*(1), 19-27. | 2 | |  |
| Eriksson, S. V., Ösby, U., Westman, J., & Hallgren, J. (2014). Coronary heart disease and hospital admission rate in patients with schizophrenia. *European Heart Journal, 35*, 527-527. | 5 | |  |
| Fichter, M. M., Rehm, J., Elton, M., Dilling, H., & Achatz, F. (1995). Mortality risk and mental disorders: longitudinal results from the Upper Bavarian Study. *Psychological Medicine, 25*(2), 297-307. | 4 | |  |
| Fiedorowicz, J. G., He, J., Merikangas, K. R., Fiedorowicz, J. G., He, J., & Merikangas, K. R. (2011). The association between mood and anxiety disorders with vascular diseases and risk factors in a nationally representative sample. *Journal of Psychosomatic Research, 70*(2), 145-154. | 1 | |  |
| Fiedorowicz, J. G., Jancic, D., Potash, J. B., Butcher, B., & Coryell, W. H. (2014). Vascular Mortality in Participants of a Bipolar Genomics Study. *Psychosomatics, 55*(5), 485-490. | 9 | |  |
| Fiedorowicz, J. G., Solomon, D. A., Endicott, J., Leon, A. C., Li, C., Rice, J. P., & Coryell, W. H. (2009). Manic/hypomanic symptom burden and cardiovascular mortality in bipolar disorder. *Psychosomatic Medicine, 71*(6), 598-606. | 9 | |  |
| Filik, R., Sipos, A., Kehoe, P.G., Burns, T., Cooper, S.J., Steven,s H., et al. (2006). The cardiovascular and respiratory health of people with schizophrenia. *Acta Psychiatrica Scandinavica, 113*(4), 298-305. | 1 | |  |
| Gabilondo, A., Alonso-Moran, E., Nuno-Solinis, R., Orueta, J. F., & Iruin, A. (2017). Comorbidities with chronic physical conditions and gender profiles of illness in schizophrenia. Results from PREST, a new health dataset. *Journal of Psychosomatic Research, 93*, 102-109. | 1 | |  |
| Fridell, M., Backstrom, M., Hesse, M., Krantz, P., Perrin, S., & Nyhlen, A. (2019). Prediction of psychiatric comorbidity on premature death in a cohort of patients with substance use disorders: a 42-year follow-up. *BMC Psychiatry, 19*(1), 150. | 3 | |  |
| Furtjes, A. E., Lewis, C. M., Hagenaars, S. P., Coleman, J. R. I., & Tyrrell, J. (2021). Associations and limited shared genetic aetiology between bipolar disorder and cardiometabolic traits in the UK Biobank. *Psychol. Med.*, 1-10. | 1 | |  |
| Gale, C. R., Batty, G. D., Osborn, D. P., Tynelius, P., Whitley, E., & Rasmussen, F. (2012). Association of mental disorders in early adulthood and later psychiatric hospital admissions and mortality in a cohort study of more than 1 million men. *Archives of General Psychiatry, 69*(8), 823-831. | 3 | |  |
| Gasse, C., Laursen, T. M., & Mortensen, P. B. (2011). Cardiovascular treatment and excess mortality in individuals with Schizophrenia. *Pharmacoepidemiology and Drug Safety, 20*, S196. | 3 | |  |
| Gatov, E., Rosella, L., Chiu, M., & Kurdyak, P. A. (2017). Trends in standardized mortality among individuals with schizophrenia, 1993-2012: a population-based, repeated cross-sectional study. *CMAJ : Canadian Medical Association journal = journal de l'Association medicale canadienne, 189*(37), E1177-E1187. | 1 | |  |
| Gavero, G. (2015). Comorbidity of bipolar disorder and heart disease among adults in Hawaii. *Psychotherapy and Psychosomatics, 84*, 24-25. | 1 | |  |
| Goldstein, B. I., Fagiolini, A., Houck, P., & Kupfer, D. J. (2009). Cardiovascular disease and hypertension among adults with bipolar I disorder in the United States. *Bipolar disorders, 11*(6), 657-662. | 1 | |  |
| Ghani, M., Kuruppu, S., Pritchard, M., Harris, M., Weerakkody, R., Stewart, R., et al. (2021). Vascular surgery receipt and outcomes for people with serious mental illnesses: Retrospective cohort study using a large mental healthcare database in South London. *J. Psychosom. Res., 147*, N.PAG-N.PAG. | 3 | |  |
| Goldstein, B. I., Houck, P. R., & Fagiolini, A. (2009). Prevalence and correlates of coronary heart disease and hypertension in bipolar I disorder. *Bipolar Disorders, 11*, 43-44. | 1 | |  |
| Goldstein, B. I., Wang, S., Schaffer, A., & Blanco, C. (2013). Excessive risk of new-onset cardiovascular disease among adults with bipolar I disorder. *Bipolar Disorders, 15*, 111-112 | 5 | |  |
| Guan, N.C., Termorshuizen, F., Laan, W., Smeets, H.M., Zainal, N.Z., Kahn, R.S., et al. (2013). Cancer mortality in patients with psychiatric diagnoses: a higher hazard of cancer death does not lead to a higher cumulative risk of dying from cancer. *Social Psychiatry and Psychiatric Epidemiology, 48*(8), 1289-1295. | 3 | |  |
| Haklai, Z., Goldberger, N., Stein, N., Pugachova, I., & Levav, I. (2011). The mortality risk among persons with psychiatric hospitalizations. *Israel Journal of Psychiatry and Related Sciences, 48*(4), 230-239. | 4 | |  |
| Hahn, M. K., & Panda, R. (2020). Mortality Risk Following Acute Coronary Syndrome Among Patients With Schizophrenia Spectrum Disorders-Addressing the Gaps. *Schizophr. Bull., 46*(4), 743-744. | 8 | |  |
| Hamer, M., Stamatakis, E., & Steptoe, A. (2008). Psychiatric hospital admissions, behavioral risk factors, and all-cause mortality: the Scottish health survey. *Archives of Internal Medicine, 168*(22), 2474-2479. | 4 | |  |
| Hallgren, J., Osby, U., Westman, J., & Gissler, M. (2019). Mortality trends in external causes of death in people with mental health disorders in Sweden, 1987-2010. *Scandinavian journal of public health, 47*(2), 121-126. | 3 | |  |
| Hannerz, H., & Borga, P. (2000). Mortality among persons with a history as psychiatric inpatients with functional psychosis. *Social Psychiatry and Psychiatric Epidemiology, 35*(8), 380-387. | 11 | |  |
| Han, L., Doran, T., Holt, R. I. G., Hewitt, C., Jacobs, R., Prady, S. L., et al. (2021). Impact of severe mental illness on healthcare use and health outcomes for people with type 2 diabetes: a longitudinal observational study in England. *The British journal of general practice : the journal of the Royal College of General Practitioners, 71*(709), e565-e573. | 2 | |  |
| Haugland, G., Craig, T. J., Goodman, A. B., & Siegel, C. (1983). MORTALITY IN THE ERA OF DEINSTITUTIONALIZATION. *American Journal of Psychiatry, 140*(7), 848-852. | 4 | |  |
| Harrison, P. J., & Luciano, S. (2021). Incidence of Parkinson's disease, dementia, cerebrovascular disease and stroke in bipolar disorder compared to other psychiatric disorders: An electronic health records network study of 66 million people. *Bipolar disorders, 23*(5), 454-462. | 9 | |  |
| Healy, D., Le Noury, J., Harris, M., Butt, M., Linden, S., Whitaker, C., et al. (2012). Mortality in schizophrenia and related psychoses: Data from two cohorts, 1875-1924 and 1994-2010. *BMJ Open, 2*(5), e001810. | 11 | |  |
| Henderson, D. C., Nguyen, D. D., Copeland, P. M., Hayden, D. L., Borba, C. P., Louie, P. M., et al. C. (2005). Clozapine, diabetes mellitus, hyperlipidemia, and cardiovascular risks and mortality: results of a 10-year naturalistic study. *The Journal of clinical psychiatry, 66*(9), 1116-1121. | 9 | |  |
| Heiberg, I. H., Jacobsen, B. K., Balteskard, L., Bramness, J. G., Naess, O., Ystrom, E., et al. (2019). Undiagnosed cardiovascular disease prior to cardiovascular death in individuals with severe mental illness. *Acta Psychiatr. Scand., 139*(6), 558-571. | 3 | |  |
| Heiberg, I. H., Nesvag, R., Balteskard, L., Bramness, J. G., Hultman, C. M., Naess, O., et al. (2020). Diagnostic tests and treatment procedures performed prior to cardiovascular death in individuals with severe mental illness. *Acta Psychiatr. Scand., 141*(5), 439-451. | 3 | |  |
| Hendrie, H. C., Tu, W., Tabbey, R., Purnell, C. E., Ambuehl, R. J., & Callahan, C. M. (2014). Health outcomes and cost of care among older adults with schizophrenia: A 10-year study using medical records across the continuum of care. *American Journal of Geriatric Psychiatry, 22*(5), 427-436. | 11 | |  |
| Hennessy, S., Bilker, W. B., Knauss, J. S., Margolis, D. J., Kimmel, S. E., Reynolds, R. F., et al. (2002). Cardiac arrest and ventricular arrhythmia in patients taking antipsychotic drugs: Cohort study using administrative data. *BMJ: British Medical Journal, 325*(7372), 1070-1072. | 11 | |  |
| Henriksson, K. M., Malfert, M., Bergman, E. M., Terent, A., & Asberg, S. (2013). Stroke Morbidity And Mortality In Patients With A History Of Psychosis-an Observational Study Of 158478 Stroke Cases In The National Swedish Riks-Stroke Cohort. *Stroke, 44*(2), 1. | 1 | |  |
| Herrmann, H. E., Baldwin, J. A., & Christie, D. (1983). A RECORD-LINKAGE STUDY OF MORTALITY AND GENERAL-HOSPITAL DISCHARGE IN PATIENTS DIAGNOSED AS SCHIZOPHRENIC. *Psychological Medicine, 13*(3), 581-593. | 11 | |  |
| Henriques, F., Ferreira, A. R., Goncalves-Pinho, M., Freitas, A., & Fernandes, L. (2022). Bipolar disorder and medical comorbidities: A Portuguese population-based observational retrospective study (2008-2015). *J. Affect. Disord., 298*, 232-238. | 9 | |  |
| Hewer, W., & Rossler, W. (1997). Mortality among inpatients with functional psychiatric disorder. *Fortschritte Der Neurologie Psychiatrie, 65*(4), 171-181. | 10 | |  |
| Hewer, W., Rossler, W., Fatkenheuer, B., & Loffler, W. (1995). Mortality among patients in psychiatric hospitals in Germany. *Acta Psychiatrica Scandinavica, 91*(3), 174-179. | 10 | |  |
| Hoang, U., Goldacre, M. J., & Stewart, R. (2013). Avoidable mortality in people with schizophrenia or bipolar disorder in England. *Acta Psychiatrica Scandinavica, 127*(3), 195-201. | 10 | |  |
| Honkonen, H., Mattila, A. K., Lehtinen, K., Elo, T., Haataja, R., & Joukamaa, M. (2008). Mortality of Finnish acute psychiatric hospital patients. *Social Psychiatry and Psychiatric Epidemiology, 43*(8), 660-666. | 3 | |  |
| Hou, P. Y., Hung, G. C. L., Jhong, J. R., Tsai, S. Y., Chen, C. C., & Kuo, C. J. (2015). Risk factors for sudden cardiac death among patients with schizophrenia. *Schizophrenia Research, 168*(1-2), 395-401. | 3 | |  |
| Høyer, E. H., Mortensen, P. B., & Olesen, A. V. (2000). Mortality and causes of death in a total national sample of patients with affective disorders admitted for the first time between 1973 and 1993. *British Journal of Psychiatry, 176*, 76-82. | 4 | |  |
| Hsu, W.-Y., Lin, C.-L., & Kao, C.-H. (2016). A Population-Based Cohort Study on Peripheral Arterial Disease in Patients with Schizophrenia. *PLoS ONE, 11*(2), e0148759. | 3 | |  |
| Huang, K.-L., Su, T.-P., Tzeng-Ji, C., Chou, Y.-H., & Bai, Y.-M. (2009). Comorbidity of cardiovascular diseases with mood and anxiety disorder: A population based 4-year study. *Psychiatry and Clinical Neurosciences, 63*(3), 401-409. | 1 | |  |
| Hussar, A. E. (1965). CORONARY HEART DISEASE IN CHRONIC SCHIZOPHRENIC PATIENTS: A CLINICOPATHOLOGIC STUDY. *Circulation, 31*, 919-929. | 1 | |  |
| Hussar, A. E. (1966). Leading causes of death in institutionalized chronic schizophrenic patients: a study of 1,275 autopsy protocols. *The Journal of nervous and mental disease, 142*(1), 45-57. | 1 | |  |
| Jayatilleke, N., Hayes, R. D., Dutta, R., Shetty, H., Hotopf, M., Chang, C. K., & Stewart, R. (2017). Contributions of specific causes of death to lost life expectancy in severe mental illness. *European Psychiatry, 43*, 109-115. | 11 | |  |
| Ishida, T., Takahashi, K., Sugiyama, K., Hamabe, Y., Mimura, M., Suzuki, T., et al. (2020). How common is pulmonary embolism compared to acute myocardial infarction among patients with severe mental illnesses? *Psychiatry Clin. Neurosci., 74*(4), 277-278. | 9 | |  |
| Jensen, S., Torp-Pedersen, C. T. P., Skals, R. K., Aagaard, J., & Jensen, S. E. (2017). Increased prevalence of heart failure in patients with schizophrenia. *European Heart Journal, 38*(Supplement 1), 912. | 1 | |  |
| Jerrell, J. M., & McIntyre, R. S. (2007). Cerebro- and cardiovascular conditions in adults with schizophrenia treated with antipsychotic medications. *Human psychopharmacology, 22*(6), 361-364. | 9 | |  |
| Joukamaa, M., Heliovaara, M., Knekt, P., Aromaa, A., Raitasalo, R., & Lehtinen, V. (2001). Mental disorders and cause-specific mortality. *British Journal of Psychiatry, 179*, 498-502. | 2 | |  |
| Joukamaa, M., Heliovaara, M., Knekt, P., Aromaa, A., Raitasalo, R., & Lehtinen, V. (2006). Schizophrenia, neuroleptic medication and mortality. *British Journal of Psychiatry, 188*, 122-127. | 2 | |  |
| Keinanen, J., Mantere, O., Markkula, N., Partti, K., Perala, J., Saarni, S. I., et al. (2017). Mortality in people with psychotic disorders in Finland: A population-based 13-year follow-up study. *Schizophrenia Research*. | 3 | |  |
| Kilbourne, A. M., Ignacio, R. V., Kim, H. M., & Blow, F. C. (2009). Are VA patients with serious mental illness dying younger? *Psychiatric Services, 60*(5), 589 | 1 | |  |
| Kendall, K. M., John, A., Lee, S. C., Rees, E., Pardinas, A. F., Banos, M. D. P., et al. (2020). Impact of schizophrenia genetic liability on the association between schizophrenia and physical illness: Data-linkage study. *BJPsych Open, 6*. | 1 | |  |
| Kerner, B., & Lambert, C. G. (2017). Cardiovascular risk factor in bipolar disorder assessed through claims data and large population based genetic studies. *Bipolar disorders, 19*, 40‐. | 8 | |  |
| Kessing, L. V., Ziersen, S. C., Andersen, P. K., & Vinberg, M. (2021). A nation-wide population-based longitudinal study on life expectancy and cause specific mortality in patients with bipolar disorder and their siblings. *J. Affect. Disord., 294*, 472-476. | 3 | |  |
| Kisely, S., Hancock, K., & Lawrence, D. (2012). Life expectancy and causes of excess mortality in patents of mental health services in Western Australia. A population based study. *Australian and New Zealand Journal of Psychiatry, 46*, 44. | 4 | |  |
| Kisely, S., Smith, M., Lawrence, D., & Maaten, S. (2005). Mortality in individuals who have had psychiatric treatment: population-based study in Nova Scotia. *British Journal of Psychiatry, 187*, 552-558. | 10 | |  |
| Kjelsberg, E. (2000). Adolescent psychiatric in-patients. A high-risk group for premature death. *British Journal of Psychiatry, 176*, 121-125. | 4 | |  |
| Kleijer, B. C., Koek, H. L., van Marum, R. J., Jansen, P. A., Egberts, T. C., & Heerdink, E. R. (2012). Risk of acute coronary syndrome in elderly users of antipsychotic drugs: a nested case-control study. *Heart, 98*(16), 1166-1171. | 4 | |  |
| Kondo, S., Kumakura, Y., Kanehara, A., Nagato, D., Ueda, T., Matsuoka, T., et al. (2017). Premature deaths among individuals with severe mental illness after discharge from long-term hospitalisation in Japan: A naturalistic observation during a 24-year period. *BJPsych Open, 3*(4), 193-495. | 4 | |  |
| Koskenvuo, M., Kaprio, J., Kesaniemi, A., & Poikolainen, K. (1986). Alcohol-related diseases associated with ischaemic heart disease: a three-year follow-up of middle-aged male hospital patients. *Alcohol and alcoholism (Oxford, Oxfordshire), 21*(3), 251-256. | 4 | |  |
| Kugathasan, P., Jensen Eggert, S., & Aagaard, J. (2017). Mortality after myocardial infarction in patients with schizophrenia: A national register-based cohort study. *Journal of Psychosomatic Research, 97*, 157-157. | 11 | |  |
| Kowal, C., Peyre, H., Amad, A., Pelissolo, A., Leboyer, M., Schurhoff, F., et al. (2020). Psychotic, mood, and anxiety disorders and venous thromboembolism: A systematic review and meta-analysis. *Psychosom. Med., 82*(9), 838-849. | 8 | |  |
| Kowalec, K., Lu, Y., Song, J., Dalman, C., Hultman, C. M., Larsson, H., et al. (2021). The association between family history and genomic burden with schizophrenia mortality: a Swedish population-based register and genetic sample study. *Translational psychiatry, 11*(1), 163. | 9 | |  |
| Krieger, I., Tzur Bitan, D., Comaneshter, D., Cohen, A., & Feingold, D. (2019). Increased risk of smoking-related illnesses in schizophrenia patients: A nationwide cohort study. *Schizophr. Res., 212*, 121-125. | 2 | |  |
| Krupchanka, D., Mladá, K., Winkler, P., Khazaal, Y., & Albanese, E. (2018). Mortality in people with mental disorders in the Czech Republic: a nationwide, register-based cohort study. *The Lancet Public Health, 3*(6), e289-e295. | 10 | |  |
| Kugathasan, P., Jensen, S. E., Laursen, T. M., Aagaard, J., & Nielsen, R. E. (2017). Increased mortality following myocardial infarction in patients with schizophrenia: A national population based cohort study. *European Heart Journal, 38*(Supplement 1), 1408. | 5 | |  |
| Kugathasan, P., Laursen, T. M., Grontved, S., Jensen, S. E., Aagaard, J., & Nielsen, R. E. (2018). Increased long-term mortality after myocardial infarction in patients with schizophrenia. *Schizophrenia Research, 199*, 103-108. | 5 | |  |
| Kumar, C. T. S. (2004). Physical illness and schizophrenia. *The British Journal of Psychiatry, 184*(6), 541. | 8 | |  |
| Kugathasan, P., Johansen, M. B., Jensen, M. B., Aagaard, J., Nielsen, R. E., & Jensen, S. E. (2019). Coronary Artery Calcification and Mortality Risk in Patients With Severe Mental Illness. *Circulation. Cardiovascular imaging, 12*(3), e008236. | 2 | |  |
| Kugathasan, P., Stubbs, B., Aagaard, J., Jensen, S. E., Munk Laursen, T., & Nielsen, R. E. (2019). Increased mortality from somatic multimorbidity in patients with schizophrenia: a Danish nationwide cohort study. *Acta Psychiatr. Scand., 140*(4), 340-348. | 3 | |  |
| Larsen, J. I., Andersen, U. A., Becker, T., Bickel, G. G., Bork, B., Cordes, J., et al. (2013). Cultural diversity in physical diseases among patients with mental illnesses. *Australian and New Zealand Journal of Psychiatry, 47*(3), 250-258. | 1 | |  |
| Lai, F. T. T., Guthrie, B., Mercer, S. W., Smith, D. J., Yip, B. H. K., Chung, G. K. K., et al. (2020). Association between antipsychotic use and acute ischemic heart disease in women but not in men: a retrospective cohort study of over one million primary care patients. *BMC Med., 18*(1), 289. | 4 | |  |
| Laursen, T. M. (2011). Life expectancy among persons with schizophrenia or bipolar affective disorder. *Schizophrenia Research, 131*(1–3), 101-104. | 11 | |  |
| Laursen, T. M., Mortensen, P. B., MacCabe, J. H., Cohen, D., & Gasse, C. (2012). Cardiovascular drug use in patients with schizophrenia or bipolar disorder. *Schizophrenia Research, 136*, S219. | 5 | |  |
| Laursen, T. M., Munk-Olsen, T., Agerbo, E., Gasse, C., & Mortensen, P. B. (2009). Somatic hospital contacts, invasive cardiac procedures, and mortality from heart disease in patients with severe mental disorder. *Archives of General Psychiatry, 66*(7), 713-720. | 5 | |  |
| Lawrence, D., Jablensky, A. V., Holman, C. D. J., & Pinder, T. J. (2000). Mortality in Western Australian psychiatric patients. *Social Psychiatry and Psychiatric Epidemiology, 35*(8), 341-347. | 10 | |  |
| Lesage, A. D., Trapani, V., & Tansella, M. (1990). EXCESS MORTALITY BY NATURAL CAUSES OF ITALIAN SCHIZOPHRENIC-PATIENTS. *European Archives of Psychiatry and Clinical Neuroscience, 239*(6), 361-365. | 10 | |  |
| Lim, L. C., Sim, L. P., & Chiam, P. C. (1993). Mortality of public mental health patients: a Singapore experience. *Australian and New Zealand Journal of Psychiatry, 27*(1), 36-41. | 3 | |  |
| Limosin, F., Loze, J. Y., Philippe, A., Casadebaig, F., & Rouillon, F. (2007). Ten-year prospective follow-up study of the mortality by suicide in schizophrenic patients. *Schizophrenia Research, 94*(1-3), 23-28. | 3 | |  |
| Lin, H. C., Huang, C. C., Chen, S. F., Chen, Y. H., Lin, H.-C., Huang, C.-C., et al. (2011). Increased risk of avoidable hospitalization among patients with schizophrenia. *Canadian Journal of Psychiatry, 56*(3), 171-178. | 2 | |  |
| Lindelius, R., & Kay, D. W. (1973). Some changes in the pattern of mortality in schizophrenia, in Sweden. *Acta Psychiatrica Scandinavica, 49*(3), 315-323. | 11 | |  |
| Lin, H.-C., Tsai, S.-Y., & Lee, H.-C. (2007). Increased risk of developing stroke among patients with bipolar disorder after an acute mood episode: a six-year follow-up study. *J. Affect. Disord., 100*(1-3), 49-54. | 9 | |  |
| Lin, H., Tsai, S., & Lee, H. (2008). No higher risk of myocardial infarction among bipolar patients in a 6-year follow-up of acute mood episodes. *Psychosom. Med., 70*(1), 73-76. | 9 | |  |
| Lin, H. C., Hsiao, F. H., Pfeiffer, S., Hwang, Y. T., & Lee, H. C. (2008). An increased risk of stroke among young schizophrenia patients. *Schizophr. Res., 101*(1-3), 234-241. | 9 | |  |
| L'Italien, G. J. (2007). Double jeopardy for the mentally ill: Higher cardiovascular risk and reduced frequency of certain interventional procedures. *Future Cardiology, 3*(3), 239-242. | 8 | |  |
| Liu, H. C., Yang, S. Y., Liao, Y. T., Chen, C. C., & Kuo, C. J. (2016). Antipsychotic medications and risk of acute coronary syndrome in schizophrenia: A nested case-control study. *PLoS ONE, 11*(9), e0163533. | 9 | |  |
| Madarasz, W., Manzardo, A., Mortensen, E. L., Penick, E., Knop, J., Sorensen, H., et al. (2012). Forty-five-year mortality rate as a function of the number and type of psychiatric diagnoses found in a large Danish birth cohort. *Canadian Journal of Psychiatry. Revue Canadienne de Psychiatrie, 57*(8), 505-511. | 3 | |  |
| Makikyro, T., Karvonen, J. T., Hakko, H., Nieminen, P., Joukamaa, M., Isohanni, M., et al. (1998). Comorbidity of hospital-treated psychiatric and physical disorders with special reference to schizophrenia: A 28 year follow-up of the 1966 Northern Finland general population birth cohort. *Public Health, 112*(4), 221-228. | 11 | |  |
| Mansuri, Z., Patel, A., Mahuwala, Z., Mansuri, U., Goswami, R., Mehta, T., et al. (2016). Burden and Outcomes of Acute Ischemic Stroke in Patients with Psychosis. *Annals of Neurology, 80*, S125-S125. | 1 | |  |
| Mansuri, Z., Patel, A., Nadkarni, G., Mahuwala, Z., Mehta, T., Mansuri, U., . . . Onteddu, S. R. (2016). Burden and Outcomes of Acute Hemorrhagic Stroke in Patients with Psychosis. *Annals of Neurology, 80*, S36-S36. | 1 | |  |
| Manu, P. (2015). Sudden death in psychiatric settings. *Handbook of medicine in psychiatry., 2nd ed.*, 3-11. | 1 | |  |
| Martin, J. L., McLean, G., Park, J., Martin, D. J., Connolly, M., Mercer, S. W., & Smith, D. J. (2014). Impact of socioeconomic deprivation on rate and cause of death in severe mental illness. *BMC Psychiatry, 14*(1), 261. | 11 | |  |
| Martin, R. L., Cloninger, C. R., Guze, S. B., & Clayton, P. J. (1985). Mortality in a follow-up of 500 psychiatric outpatients. I. Total mortality. *Archives of General Psychiatry, 42*(1), 47-54. | 3 | |  |
| Martin, R. L., Cloninger, C. R., Guze, S. B., & Clayton, P. J. (1985). Mortality in a follow-up of 500 psychiatric outpatients. II. Cause-specific mortality. *Archives of General Psychiatry, 42*(1), 58-66. | 10 | |  |
| McLean, G., Martin, J. L., Martin, D. J., Guthrie, B., Mercer, S. W., & Smith, D. J. (2014). Standard cardiovascular disease risk algorithms underestimate the risk of cardiovascular disease in schizophrenia: evidence from a national primary care database. *Schizophrenia Research, 159*(1), 176-181. | 1 | |  |
| Melle, I., Olav Johannesen, J., Haahr, U. H., ten Velden Hegelstad, W., Joa, I., Langeveld, J., et al. (2017). Causes and predictors of premature death in first-episode schizophrenia spectrum disorders. *World Psychiatry, 16*(2), 217-218. | 11 | |  |
| Medici, C. R., Videbech, P., Gustafsson, L. N., & Munk-Jorgensen, P. (2015). Mortality and secular trend in the incidence of bipolar disorder. *J. Affect. Disord., 183*, 39-44. | 10 | |  |
| Meloni, D., Miccinesi, G., Bencini, A., Conte, M., Crocetti, E., Zappa, M., & Ferrara, M. (2006). Mortality among discharged psychiatric patients in Florence, Italy. *Psychiatric Services, 57*(10), 1474-1481. | 4 | |  |
| Miller, B., Paschall, C., & Svendsen, D. (2006). Mortality and Medical Comorbidity Among Patients With Serious Mental Illness. *Psychiatric Services, 57*(10), 1482-1487. | 10 | |  |
| Miller, C., & Bauer, M. S. (2014). Excess Mortality in Bipolar Disorders. *Current Psychiatry Reports, 16*(11), 1-7. | 8 | |  |
| Mittendorfer-Rutz, E., Kjeldgard, L., Runeson, B., Perski, A., Melchior, M., Head, J., & Alexanderson, K. (2012). Sickness absence due to specific mental diagnoses and all-cause and cause-specific mortality: a cohort study of 4.9 million inhabitants of Sweden. *PLoS ONE, 7*(9), e45788. | 10 | |  |
| Mohamed, M. O., Rashid, M., Shoaib, A., Mamas, M. A., Farooq, S., Chew-Graham, C., et al. (2019). Acute Myocardial Infarction in Severe Mental Illness: Prevalence, Clinical Outcomes, and Process of Care in U.S. Hospitalizations. *Can. J. Cardiol., 35*(7), 821-830. | 3 | |  |
| Moore, D. T., & Rosenheck, R. A. (2019). Medical-Surgical Hospitalization Among Veterans With Psychiatric and Substance Use Disorders. *Psychosomatics, 60*(6), 591-598. | 3 | |  |
| Moreno-Kustner, B., Guzman-Parra, J., Pardo, Y., Sanchidrian, Y., Diaz-Ruiz, S., & Mayoral-Cleries, F. (2021). Excess mortality in patients with schizophrenia spectrum disorders in Malaga (Spain): A cohort study. *Epidemiology and psychiatric sciences, 30*, e11. | 10 | |  |
| Morgan, M. G., Scully, P. J., Youssef, H. A., Kinsella, A., Owens, J. M., & Waddington, J. L. (2003). Prospective analysis of premature mortality in schizophrenia in relation to health service engagement: A 7.5-year study within an epidemiologically complete, homogeneous population in rural Ireland. *Psychiatry Research, 117*(2), 127-135. | 11 | |  |
| Mosley, J. C. (2016). The economic burden of comorbid serious mental illness and chronic non-communicable disease in working age adults in the United States. *Dissertation Abstracts International: Section B: The Sciences and Engineering, 77*(4-B(E)), | 1 | |  |
| Muller-Oerlinghausen, B., Wolf, T., Ahrens, B., Schou, M., Grof, E., Grof, P., et al. (1994). Mortality during initial and during later lithium treatment. A collaborative study by the International Group for the Study of Lithium-treated Patients. *Acta Psychiatrica Scandinavica, 90*(4), 295-297. | 5 | |  |
| Nemani, K. L., Greene, M. C., Ulloa, M., Vincenzi, B., Copeland, P. M., Al-Khadari, S., et al. (2019). Clozapine, Diabetes Mellitus, Cardiovascular Risk and Mortality: Results of a 21-Year Naturalistic Study in Patients with Schizophrenia and Schizoaffective Disorder. *Clin. Schizophr. Relat. Psychoses, 12*(4), 168-176. | 9 | |  |
| Newman, S. C., & Bland, R. C. (1991). Suicide risk varies by subtype of affective disorder. *Acta Psychiatrica Scandinavica, 83*(6), 420-426. | 10 | |  |
| Nielsen, A. T., Vestergaard, M., Munk-Olsen, T., Kristensen, J. K., & Laursen, T. M. (2018). Risk of diabetic complications and subsequent mortality among individuals with schizophrenia and diabetes mellitus: A nationwide population-based register study. *Schizophrenia Bulletin, 44*(Supplement 1), S88-S89. | 2 | |  |
| Nielsen, R. E., Uggerby, A. S., Jensen, S. O. W., & McGrath, J. J. (2013). Increasing mortality gap for patients diagnosed with schizophrenia over the last three decades - A Danish nationwide study from 1980 to 2010. *Schizophrenia Research, 146*(1-3), 22-27. | 11 | |  |
| Nielsen, R. E., Banner, J., & Jensen, S. E. (2021). Cardiovascular disease in patients with severe mental illness. *Nature Reviews Cardiology, 18*(2), 136-145. | 8 | |  |
| Nielsen, R. E., Straszek, S., Kovacs, Z., & Licht, R. W. (2020). Excess mortality in subjects with severe mental illness: A matter of serious concern. *Bipolar Disorders, 22*(2), 107-108. | 8 | |  |
| Nilsson, F. M., & Kessing, L. V. (2004). Increased risk of developing stroke for patients with major affective disorder: A registry study. *European Archives of Psychiatry and Clinical Neuroscience, 254*(6), 387-391. | 9 | |  |
| Niswander, G. D., Haslerud, G. M., & Mitchell, G. D. (1963). Changes in Cause of Death of Schizophrenic Patients: A Cross-Sectional and Longitudinal Study Over a 60-Year Period. *Archives of General Psychiatry, 9*(3), 229-234. | 3 | |  |
| Nordentoft, M., & Laursen, T. M. (2010). P02-411 - Development of heart disease treatment, and mortality, from 1994 to 2006 among persons with schizophrenia or bipolar disorder. *European Psychiatry, 25, SUPP 1*, 781. | 1 | |  |
| O'Connor, N., Hunt, G. E., O'Hara-Aarons, M., Hall, A., Snars, J., Storm, V., & Lambert, T. (2014). The Sydney mental health client mortality audit: What does it tell us and what are we to do? *Australasian Psychiatry, 22*(2), 154-159. | 1 | |  |
| Osborn, D., Levy, G., Nazareth, I., & King, M. (2008). Suicide and severe mental illnesses. Cohort study within the UK general practice research database. *Schizophrenia Research, 99*(1–3), 134-138. | 3 | |  |
| Osborn, D. J., Hardoon, S., Omar, R. Z., & et al. (2015). Cardiovascular risk prediction models for people with severe mental illness: Results from the prediction and management of cardiovascular risk in people with severe mental illnesses (primrose) research program. *JAMA Psychiatry, 72*(2), 143-151. | 9 | |  |
| Osborn, D. P. J. (2001). The poor physical health of people with mental illness. *West. J. Med., 175*(5), 329‐332. | 8 | |  |
| Ösby, U., Eriksson, S., Morrato, E., Newcomer, J., Erlinge, D., & Jonas, H. (2010). Excess mortality in coronary and cerebrovascular disease in Swedish patients with bipolar disorder. *Scandinavian Cardiovascular Journal, 44*, 28. | 5 | |  |
| Overs, B., Toma, C., Schofield, P., Fullerton, J., Havard, A., Green, M., et al. (2021). Medical morbidity and mortality in Australians with bipolar disorder from linked administrative data. *Bipolar Disorders, 23*(SUPPL 1), 51-52. | 11 | |  |
| Pal, S. G., Grillo, E. B., Salazar, G. F., & Odio, S. O. F. (2005). Causes of death on patients suffering from epilepsy and associated psychosis. A comparison made with schicophrenic patients and non psychiatric patients. *Revista del Hospital Psiquiatrico de la Habana, 2*(1). | 6 | |  |
| Pan, Y. J., Yeh, L. L., Chan, H. Y., & Chang, C. K. (2017). Transformation of excess mortality in people with schizophrenia and bipolar disorder in Taiwan. *Psychological Medicine, 47*(14), 2483-2493. | 10 | |  |
| Pandiani, J. A., Banks, S. M., Bramley, J., & Moore, R. (2002). Mortality of mental health service recipients in Vermont and Oklahoma. *Psychiatric Services, 53*(8), 1025-1027. | 3 | |  |
| Perez-Carceles, M. D., Inigo, C., Luna, A., & Osuna, E. (2001). Mortality in maximum security psychiatric hospital patients. *Forensic Science International, 119*(3), 279-283. | 10 | |  |
| Piatt, E. E., Munetz, M. R., & Ritter, C. (2010). An examination of premature mortality among decedents with serious mental illness and those in the general population. *Psychiatric Services, 61*(7), 663-668. | 4 | |  |
| Polcwiartek, C., Atwater, B. D., Kragholm, K., Friedman, D. J., Barcella, C. A., Attar, R., et al. (2021). Association Between ECG Abnormalities and Fatal Cardiovascular Disease Among Patients With and Without Severe Mental Illness. *Journal of the American Heart Association, 10*(2), e019416. | 4 | |  |
| Politi, P., Piccinelli, M., Klersy, C., Madini, S., Lusignani, G. S., Fratti, C., & Barale, F. (2002). Mortality in psychiatric patients 5 to 21 years after hospital admission in Italy. *Psychological Medicine, 32*(2), 227-237. | 10 | |  |
| Prieto, M. L., Schenck, L. A., Kruse, J. L., Klaas, J. P., Chamberlain, A. M., Bobo, W. V., . . . Frye, M. A. (2015). Risk of myocardial infarction and stroke in bipolar disorder: A population-based cohort study. *Bipolar Disorders, 17*, 53. | 5 | |  |
| Protty, M. B. (2019). Coronary artery disease and schizophrenia: the interplay of heart and mind. *European heart journal. Quality of care & clinical outcomes, 5*(2), 90-91. | 8 | |  |
| Rantanen, H., Koivisto, A. M., Salokangas, R. K., Helminen, M., Oja, H., Pirkola, S., et al. (2009). Five-year mortality of Finnish schizophrenia patients in the era of deinstitutionalization. *Social Psychiatry and Psychiatric Epidemiology, 44*(2), 135-142. | 9 | |  |
| Rasanen, P., Tiihonen, J., Isohanni, M., Moring, J., & Koiranen, M. (1998). Juvenile mortality, mental disturbances and criminality: a prospective study of the Northern Finland 1966 birth cohort. *Acta Psychiatrica Scandinavica, 97*(1), 5-9. | 3 | |  |
| Rasanen, S., Hakko, H., Viilo, K., Meyer-Rochow, V. B., & Moring, J. (2003). Excess mortality among long-stay psychiatric patients in Northern Finland. *Social Psychiatry and Psychiatric Epidemiology, 38*(6), 297-304. | 4 | |  |
| Rasanen, S., Meyer-Rochow, V. B., Moring, J., & Hakko, H. (2007). Hospital-treated physical illnesses and mortality: An 11-year follow-up study of long-stay psychiatric patients. *Eur. Psychiatry, 22*(4), 211-218. | 9 | |  |
| Rasoul, D., Wong, S., Chandran, S., Uppal, H., Sarma, J., & Potluri, R. (2016). Psychiatric co-morbidities and tendencies in patients with non-ischaemic heart failure (NIHF) - A large observational cohort study spanning 14 years. *Heart, 102*, A7. | 4 | |  |
| Rassidakis, N. C., & et al. (1975). Illnesses of the psychosomatic continuum and coronary heart disease. *International Mental Health Research Newsletter, 17*(3), 6. | 6 | |  |
| Ribe, A. R., Laursen, T. M., Sandbaek, A., Charles, M., Nordentoft, M., & Vestergaard, M. (2014). Long-term mortality of persons with severe mental illness and diabetes: a population-based cohort study in Denmark. *Psychol. Med., 44*(14), 3097-3107. | 9 | |  |
| Richmond-Rakerd, L. S., D'Souza, S., Milne, B. J., Caspi, A., & Moffitt, T. E. (2021). Longitudinal Associations of Mental Disorders with Physical Diseases and Mortality among 2.3 Million New Zealand Citizens. *JAMA Network Open, 4*(1), e2033448. | 4 | |  |
| Ringback Weitoft, G., Gullberg, A., & Rosen, M. (1998). Avoidable mortality among psychiatric patients. *Social Psychiatry and Psychiatric Epidemiology, 33*(9), 430-437. | 3 | |  |
| Risgaard, B., Waagstein, K., Winkel, B. G., Jabbari, R., Lynge, T. H., Glinge, C., et al. (2014). Sudden cardiac death in young adults with psychiatric disorders-a danish nationwide study. *Heart Rhythm, 11*(5 SUPPL. 1), S388. | 1 | |  |
| Rorsman, B. (1974). Mortality among psychiatric patients. *Acta Psychiatrica Scandinavica, 50*(3), 354-375. | 4 | |  |
| Rorsman, B., Hagnell, O., & Lanke, J. (1982). Mortality in the Lundby study. Natural death in different forms of mental disorder in a total population investigated during a 25-year period. *Neuropsychobiology, 8*(4), 188-197. | 3 | |  |
| Rose, E., Chen, S., Turrion, C., Jenkins, C., Cardinal, R. N., & Fernandez-Egea, E. (2020). Causes of death in clozapine-treated patients in a catchment area: a 10-year retrospective case-control study. *European neuropsychopharmacology : the journal of the European College of Neuropsychopharmacology, 36*, 160-166. | 9 | |  |
| Roy, B., Jianheng, L., Lally, C., Shah, A., Bloomgren, G., & Wenten, M. (2020). DISEASE PREVALENCE, COMORBID CONDITIONS, AND MEDICATION UTILIZATION AMONG PATIENTS WITH SCHIZOPHRENIA IN THE UNITED STATES...Schizophrenia International Research Society (SIRS) 2020 Congress. *Schizophr. Bull., 46*, S182-S183. | 10 | |  |
| Ruschena, D., Mullen, P. E., Burgess, P., Cordner, S. M., Barry-Walsh, J., Drummer, O. H., et al. (1998). Sudden death in psychiatric patients. *British Journal of Psychiatry, 172*, 331-336. | 3 | |  |
| Saugstad, L., & Odegard, O. (1985). Recent rise in supposedly stress dependent causes of death in psychiatric hospitals in Norway indicating increased 'stress' in hospitals? *Acta Psychiatrica Scandinavica, 71*(4), 402-409. | 1 | |  |
| Saugstad, L. F., & Odegard, O. (1979). Mortality in psychiatric hospitals in Norway 1950--74. *Acta Psychiatrica Scandinavica, 59*(4), 431-447. | 1 | |  |
| Scheuer, S. H., Kosjerina, V., Lindekilde, N., Pouwer, F., Carstensen, B., Jorgensen, M. E., et al. (2021). Incidence of micro- and macrovascular complications among persons with type 2 diabetes with and without severe mental illness: A nationwide study. *Diabetes, 70*(SUPPL 1). | 2 | |  |
| Schneider, B., Muller, M. J., & Philipp, M. (2001). Mortality in affective disorders. *Journal of Affective Disorders, 65*(3), 263-274. | 10 | |  |
| Schoepf, D., & Heun, R. (2014). Bipolar disorder and comorbidity: increased prevalence and increased relevance of comorbidity for hospital-based mortality during a 12.5-year observation period in general hospital admissions. *Journal of Affective Disorders, 169*, 170-178. | 3 | |  |
| Schoepf, D., Potluri, R., Uppal, H., Natalwala, A., Narendran, P., & Heun, R. (2012). Type-2 diabetes mellitus in schizophrenia: increased prevalence and major risk factor of excess mortality in a naturalistic 7-year follow-up. *European Psychiatry: the Journal of the Association of European Psychiatrists, 27*(1), 33-42. | 3 | |  |
| Schoepf, D., Uppal, H., Potluri, R., & Heun, R. (2014). Physical comorbidity and its relevance on mortality in schizophrenia: a naturalistic 12-year follow-up in general hospital admissions. *European Archives of Psychiatry and Clinical Neuroscience, 264*(1), 3-28. | 3 | |  |
| Schulman-Marcus, J., Goyal, P., Swaminathan, R. V., Feldman, D. N., Wong, S.-C., Singh, H. S., . . . Kim, L. K. (2016). Comparison of Trends in Incidence, Revascularization, and In-Hospital Mortality in ST-Elevation Myocardial Infarction in Patients With Versus Without Severe Mental Illness. *American Journal of Cardiology, 117*(9), 1405-1410. | 1 | |  |
| Schwalb, H. (1975). [Risk factors of coronary heart diseases in hospitalized psychic patients]. *Risikofaktoren der koronaren Herzkrankheiten bei hospitalisierten psychisch Kranken, 117*(28), 1181-1188. | 6 | |  |
| Schwalb, H., Schomann, C., Bruninghaus, H., Eckmann, F., Jungling, D., Reinhold, H., . . . von Waldenfels, W. (1980). [Mortality in hospitalized psychiatric patients (results from a 5-year study) (author's transl)]. *Mortalitat hospitalisierter psychiatrischer Patienten--Ergebnisse einer 5-Jahres-Studie., 48*(11), 616-627. | 6 | |  |
| Scorza, C. A., Guimaraes-Marques, M. M., Nejm, M., Scorza, F. A., Finsterer, J., & Cysneiros, R. M. (2020). Increased Risk of Sudden Cardiac Death in Schizophrenia. *Psychosomatics, 61*(6), 864-866. | 8 | |  |
| Segal, S. P., & Burgess, P. M. (2006). Effect of conditional release from hospitalization on mortality risk. *Psychiatric Services, 57*(11), 1607-1613. | 3 | |  |
| Shah, A. J., Veledar, E., Hong, Y., Bremner, J. D., & Vaccarino, V. (2011). Depression and history of attempted suicide as risk factors for heart disease mortality in young individuals. *Archives of General Psychiatry, 68*(11), 1135-1142. | 4 | |  |
| Sharma, R., & Markar, H. R. (1994). Mortality in affective disorder. *Journal of Affective Disorders, 31*(2), 91-96. | 1 | |  |
| Shen, H. N., Lu, C. L., & Yang, H. H. (2011). Increased risks of acute organ dysfunction and mortality in intensive care unit patients with schizophrenia: A nationwide population-based study. *Psychosomatic Medicine, 73*(7), 620-626. | 3 | |  |
| Simpson, J. C., & Tsuang, M. T. (1996). Mortality among patients with schizophrenia. *Schizophrenia Bulletin, 22*(3), 485-499. | 3 | |  |
| Skala, J. A. (2007). Severe mental illness increases the risk of death from coronary heart disease or stroke. *Evidence Based Mental Health, 10*(4), 105-105. | 8 | |  |
| Sohlman, B., & Lehtinen, V. (1999). Mortality among discharged psychiatric patients in Finland. *Acta Psychiatrica Scandinavica, 99*(2), 102-109. | 4 | |  |
| Sokal, J., Messias, E., Dickerson, F. B., Kreyenbuhl, J., Brown, C. H., Goldberg, R. W., & Dixon, L. B. (2004). Comorbidity of medical illnesses among adults with serious mental illness who are receiving community psychiatric services. *Journal of Nervous and Mental Disease, 192*(6), 421-427. | 1 | |  |
| Stark, C., MacLeod, M., Hall, D., O'Brien, F., & Pelosi, A. (2003). Mortality after discharge from long-term psychiatric care in Scotland, 1977-94: a retrospective cohort study. *BMC Public Health, 3*, 30. | | 4 | |
| Staudt Hansen, P., Frahm Laursen, M., Grontved, S., Puggard Vogt Straszek, S., Licht, R. W., & Nielsen, R. E. (2019). Increasing mortality gap for patients diagnosed with bipolar disorder-A nationwide study with 20 years of follow-up. *Bipolar disorders, 21*(3), 270-275. | 3 | |  |
| Stolz, P. A., Wehring, H. J., Liu, F., Love, R. C., Ellis, M., DiPaula, B. A., & Kelly, D. L. (2019). Effects of Cigarette Smoking and Clozapine Treatment on 20-Year All-Cause & Cardiovascular Mortality in Schizophrenia. *Psychiatric Quarterly*. | 9 | |  |
| Strydom, A. (2002). Mental disorders predicted all cause and cause specific deaths in Finnish adults. *Evidence Based Mental Health, 5*(3), 93-93. | 8 | |  |
| Suvisaari, J., Partti, K., Perala, J., Viertio, S., Saarni, S., Lonngvist, J., et al. (2013). Mortality and its determinants in people with psychotic disorder. *Psychosomatic Medicine, 75*(1), 60 - 67. | 10 | |  |
| Suvisaari, J., Perala, J., Saarni, S. I., Kattainen, A., Lonnqvist, J., & Reunanen, A. (2010). Coronary heart disease and cardiac conduction abnormalities in persons with psychotic disorders in a general population. *Psychiatry Research, 175*(1-2), 126-132. | 1 | |  |
| Swain, N. R., Lim, C. C. W., Levinson, D., Fiestas, F., de Girolamo, G., Moskalewicz, J., et al. (2015). Associations between DSM-IV mental disorders and subsequent non-fatal, self-reported stroke. *Journal of Psychosomatic Research, 79*(2), 130-136. | 2 | |  |
| Sweeting, J., Duflou, J., & Semsarian, C. (2013). Cardiovascular deaths in schizophrenia: A postmortem analysis. *Heart Lung and Circulation, 22*, S227. | 1 | |  |
| Sweeting, J., Duflou, J., & Semsarian, C. (2013). Postmortem analysis of cardiovascular deaths in schizophrenia: a 10-year review. *Schizophrenia Research, 150*(2-3), 398-403. | 1 | |  |
| Tidemalm, D., Waern, M., Stefansson, C. G., Elofsson, S., & Runeson, B. (2008). Excess mortality in persons with severe mental disorder in Sweden: a cohort study of 12 103 individuals with and without contact with psychiatric services. *Clinical Practice and Epidemiology in Mental Health, 4*, 23. | 10 | |  |
| Tiihonen, J., Lonnqvist, J., Wahlbeck, K., Klaukka, T., Niskanen, L., Tanskanen, A., & Haukka, J. (2009). 11-year follow-up of mortality in patients with schizophrenia: a population-based cohort study (FIN11 study). *The Lancet, 374*(9690), 620-627. | 3 | |  |
| Tiihonen, J., Mittendorfer-Rutz, E., Torniainen, M., Alexanderson, K., & Tanskanen, A. (2016). Mortality and cumulative exposure to antipsychotics, antidepressants, and benzodiazepines in patients with schizophrenia: An observational follow-up study. *American Journal of Psychiatry, 173*(6), 600-606. | 10 | |  |
| Toender, A., Vestergaard, M., Munk-Olsen, T., Larsen, J. T., Kristensen, J. K., & Laursen, T. M. (2020). Risk of diabetic complications and subsequent mortality among individuals with schizophrenia and diabetes - a population-based register study. *Schizophr. Res., 218*, 99-106. | 2 | |  |
| Tokuda, Y., Obara, H., Nakazato, N., & Stein, G. H. (2008). Acute care hospital mortality of schizophrenic patients. *Journal of Hospital Medicine, 3*(2), 110-116. | 1 | |  |
| Trapencieris, M., Snikere, S., & Pulmanis, T. (2013). Mortality among alcohol treatment patients in Latvia. *Alcohol and Alcoholism, 48*. | 4 | |  |
| Trivedi, R., Post, E. P., Piegari, R. I., Simonetti, J. A., Boyko, E. J., Asch, S., et al. (2017). Preliminary analysis of life expectancy and common causes of death among veterans with mental illnesses. *Journal of General Internal Medicine, 32*(2 Supplement 1), S284. | 10 | |  |
| Trivedi, R. B., Post, E. P., Piegari, R., Simonetti, J., Boyko, E. J., Asch, S. M., et al. (2020). Mortality Among Veterans with Major Mental Illnesses Seen in Primary Care: Results of a National Study of Veteran Deaths. *J. Gen. Intern. Med., 35*(1), 112-118. | 11 | |  |
| Truyers, C., Buntinx, F., De Lepeleire, J., De Hert, M., Van Winkel, R., Aertgeerts, B., et al. (2011). Incident somatic comorbidity after psychosis: results from a retrospective cohort study based on Flemish general practice data. *BMC Family Practice, 12*, 132. | 2 | |  |
| Tsai, S. Y., Lee, C. H., Chen, P. H., Chung, K. H., Huang, S. H., Kuo, C. J., & Wu, W. C. (2017). Risk factors for early cardiovascular mortality in patients with bipolar disorder. *Psychiatry and Clinical Neurosciences*. | 1 | |  |
| Tsuang, M. T., Woolson, R. F., & Fleming, J. A. (1980). Premature deaths in schizophrenia and affective disorders: An analysis of survival curves and variables affecting a shortened survival. *Archives of General Psychiatry, 37*(9), 979-983. | 10 | |  |
| Tsuzuki, H., & Yuasa, S. (1981). [An epidemiological study of deaths in mental hospitals (author's transl)]. *Seishin Shinkeigaku Zasshi. Psychiatria et Neurologia Japonica, 83*(5), 275-304. | 7 | |  |
| Valenti, M., Necozione, S., Busellu, G., Borrelli, G., Lepore, A. R., Madonna, R., et al. (1997). Mortality in psychiatric hospital patients: a cohort analysis of prognostic factors. *International Journal of Epidemiology, 26*(6), 1227-1235. | 4 | |  |
| Vataire, A., Ferchichi, S., Desroziers, K., Cadi-Soussi, N., Murthy, V., & Toumi, M. (2013). Association between metabolic disease and cardiovascular risk in patients with schizophrenia treated with antipsychotics. *European Neuropsychopharmacology, 23*, S495. | 9 | |  |
| Vestergaard, P., & Aagaard, J. (1991). Five-year mortality in lithium-treated manic-depressive patients. *Journal of Affective Disorders, 21*(1), 33-38. | 5 | |  |
| Walter, F., Carr, M. J., Mok, P. L. H., Astrup, A., Antonsen, S., Pedersen, C. B., et al. (2017). Premature Mortality Among Patients Recently Discharged From Their First Inpatient Psychiatric Treatment. *JAMA Psychiatry, 74*(5), 485-492. | 10 | |  |
| Westaby, J., Pankajakshan, C. N., & Sheppard, M. N. (2017). Incidence of psychiatric illnesses in sudden cardiac deaths. *Laboratory Investigation, 97*, 85A. | 1 | |  |
| Wolpert, A., Yaryura-Tobias, J. A., & Kertzner, L. (1971). Silent myocardial infarction in a chronic psychotic population. *Diseases of the Nervous System, 32*(4), 280-283. | 1 | |  |
| Wood, J. B., Evenson, R. C., Cho, D. W., & Hagan, B. J. (1985). MORTALITY VARIATIONS AMONG PUBLIC MENTAL-HEALTH PATIENTS. *Acta Psychiatrica Scandinavica, 72*(3), 218-229. | 1 | |  |
| Wu, C. S., Lai, M. S., & Gau, S. S. F. (2015). Complications and mortality in patients with schizophrenia and diabetes: Populationbased cohort study. *Br. J. Psychiatry, 207*(5). | 2 | |  |

** Reason for exclusion: 1 - Unsuitable study design or methodology unclear, 2 - Unsuitable population, 3 - Unsuitable or inadequately defined outcome, 4 - Unsuitable or inadequately defined exposure, 5 - Duplicate study, 6 - Full text not available, 7 - Not able to translate, 8 - Not a primary study, 9 - No suitable comparator, 10 - No CVD x SMI results,*

*11 - No useable measure*
