# Supplementary material for: Temporal trends in associations between severe mental illness and risk of cardiovascular disease: A systematic review and meta-analysis
Source: PLoS Med. 2022 Apr 19;19(4):e1003960. doi: 10.1371/journal.pmed.1003960 (PMC9017899; doi:10.1371/journal.pmed.1003960)
Supplement: S12 File — (DOCX) [file pmed.1003960.s012.docx]

# S12 File. Characteristics of included mortality studies

| **Study, year** | **SMI diagnosis** | **Country** | **Setting** | **Period of SMI exposure** | **Period of outcomes** | **Age at start of study (years)** | **Number of cases** | **Number of controls** | **Covariates adjusted for** | **Mean follow-up (years)** | **Outcomes (mortality)** | **Sex** | **Type of results** |
| --- | --- | --- | --- | --- | --- | --- | --- | --- | --- | --- | --- | --- | --- |
| Ahrens, 1995 | BD | Denmark, Germany, Canada, Austria | Inpatient | 1967-1989 | 1967-1989 | Mean 41 | 440 | Gen pop | Age, sex, country, year of death | 7.2 | CVD | P, M, F | SMR |
| Ajetunmobi, 2013 | SCZ, BD | UK | Inpatient | 1986-2009 | 1986-2010 | 15+, median 42.6 | 44546 | Gen pop | Age, sex, year of death, deprivation | Up to 25 | CVD | P | SMR |
| Allebeck, 1986 | SCZ | Sweden | Inpatient | 1971 | 1971-1981 | Mean 45 (est) | 1190 | Gen pop | Age, sex | 11 | CVA, CHD | P | SMR |
| Anderson, 1991 | SCZ | UK | Inpatient | 1975-1984 | 1975-1988 | NR | 532 | Gen pop | Age, sex, year of death | up to 14 | CVA, CHD | P, M, F | SMR |
| Angst, 2002 | BD | Switzerland | Inpatient | 1959-1963 | 1959-1997 | NR | 158 | Gen pop | Age, sex, year | 34-38 | CVA, CHD, CVD | P | SMR |
| Bjorkenstam, 2012 | SCZ | Sweden | Community and inpatient | 2004-2005 | 2006-2007 | 20-79, mean 47.9 | 23158 | 6101893 | Age, sex | Up to 2 | CHD | M, F | RRatio |
| Brink, 2018 | SCZ | Denmark | Inpatient | 1970-1979 | 1980-2012 | 18-40 | 4544 | 22597 | Age, sex, year, Charlson score, education, marital status | Up to 41 | CVD | P | RRatio |
| Brodersen, 2000 | BD | Denmark | Inpatient | 1981-1983 | 1981-1997 | 15-70, mean 40 | 133 | Gen pop | Age, sex, year of death | 16 | CVD | P, M, F | SMR |
| Brook, 1984 | SCZ | Netherlands | Inpatient | 29220 | 1980-1981 | 20+ | 5226 | Gen pop | Age, sex | 2 | CVA, CHD | P, M, F | SMR |
| Brown, 2010 | SCZ | UK | Community | 1981 | 1981-2006 | 16-65, mean 40.7 | 370 | Gen pop | Age, sex, year | Up to 25 | CVA, CHD, CVD | P, M, F | SMR |
| Buda, 1988 | SCZ | USA | Inpatient | 1934-1945 | 1934-1974 | NR | 332 | Gen pop | Age, sex | Up to 40 | CVD | P | SMR |
| Callaghan, 2014 | SCZ, BD | USA | Inpatient | 1990-2005 | 1990-2005 | 35+ at mid-point of follow-up | 248820 | Gen pop | Age, sex, race | SCZ 7.4, BD 7.6 | CVA, CHD, CVD | P, M, F | SMR |
| Casadebaig, 1999 | SCZ | France | Community and inpatient | 1993 | 1993-1996 | 18-64 | 3470 | Gen pop | Age, sex | Up to 3 | CVA, CHD | P | SMR |
| Castagnini, 2013 | SCZ, BD | Denmark | Community and inpatient | 1995-2008 | 1995-2008 | 15-64, mean SCZ: 32.6, BD: 41.8 | 7776 | Gen pop | Age, sex | Up to 14 | CVA, CHD | P | SMR |
| Chan, 2021 | BD | Hong Kong | Community and inpatient | 2002-2018 | 2008-2018 | 15+ | 12556 | Gen pop | Age, sex and calendar year | 8.5 | CVA, CHD, CVD | P, M, F | SMR |
| Crump, 2013a | SCZ | Sweden | Community and inpatient | 2001-2002 | 2003-2007 | 25+ | 8277 | 6097834 | Age, sex, marital status, education, employment status, income | 5 | CVA, CHD, CVD | M, F | HR |
| Crump, 2013b | BD | Sweden | Community and inpatient | 2001-2002 | 2003-2008 | 20+ | 6618 | 6587036 | Age, sex, marital status, education, employment status, income | 6 | CVA, CHD, CVD | M, F | HR |
| Cunningham, 2014 | SCZ, BD | New Zealand | Community and inpatient | 2002-2010 | 2002-2010 | 18-64, mean SCZ: 37.6, BD: 39.9 | 32139 | Gen pop | Age, sex | Up to 9 | CVD | P, M, F | SMR |
| Curkendall, 2004 | SCZ | Canada | Community | 1994-1995 | 1996-March 1999 | 18+, mean 49.6 | 2405 | 9175 | Age, sex, >1 of HT, hyperlipidaemia, DM, CVD, COPD | NR | CVD | P | RRatio |
| Dalgard, 1966 | SMI | Norway | Inpatient | 1951-1955 | 1951-1961 | NR | 1126 | 1126 | Age, sex, marital status, occupation | 6.5 | CVA, CHD | P | RRatio |
| Das-Munshi, 2017 | SMI | UK | Community | 2007-2014 | 2007-2014 | 15+ | 18201 | Gen pop | Age, sex, length of follow-up | 6.36 (median) IQR 3.26-9.92 | CVD | P | SMR |
| Daumit, 2010 | SCZ, BD | USA | Community | July 1992-June 1993 | 1994-2001 | 21-62, mean 41.6 | 3361 (est) | Gen pop | Age, sex, race | Up to 8.5 | CHD | P | RRatio |
| Dutta, 2012 | SMI | UK | Community | 1965-2004 | 1965-Mar 2007 | 16+ | 2723 | Gen pop | Age, sex, year | 11.5 | CVD | P, M, F | SMR |
| Fors, 2007 | SCZ | Sweden | Community | 1981-1990 | 1991-2000 | 18+ | 255 | 1275 | Age, sex, living area | Up to 10 | CVD | P, M, F | RRatio |
| Giel, 1978 | SCZ | Netherlands | Inpatient | 12/31/1969 | 1970-1971 | 20+ | 4172 | Gen pop | Age, sex | Up to 2 | CVA, CHD | P, M, F | SMR |
| Girardi, 2021 | SCZ | Italy | Community and inpatient | 2008 | 2008-2018 | 18-84, median 48 | 12996 | Gen pop | Age, sex and calendar year | 9.6 | CVA, CHD, CVD | P, M, F | SMR |
| Grigoletti, 2009 | SCZ | Italy | Community | 1982-2001 | 1982-2001 | 14+ | 556 | Gen pop | Age, sex, year | Up to 20 | CVD | P | SMR |
| Hansen, 2001 | SMI | Norway | Inpatient | Aug 1980-1992 | Aug 1980-1992 | NR | NR | NR | Age, sex | Up to 12.5 | CVD | M, F | SMR |
| Hayes, 2017 | SCZ, BD | UK | Community | 2000-2014 | 2000-2014 | 16+, median SCZ: 42.5, BD: 42.8 | 39838 | 219387 | Age, sex, time, deprivation, ethnicity, primary care contacts | median: SCZ 2.47, BD 2.32 | CVD | P | HR |
| Heiberg, 2018 | SCZ | Norway | Community and inpatient | 2009-2015 | 2009-2015 | 20-79, mean 45.6 | 20537 | Gen pop | Age, sex | Up to 6 | CVD | P, M, F | SMR |
| Heila, 2005 | SCZ | Finland | Inpatient | 1980-1996 | 1980-1996 | All ages | 58761 | Gen pop | Age, sex, year | Over 10 | CHD | M, F | RRatio |
| Hiroeh, 2008 | SCZ | Denmark | Inpatient | 1973-1993 | 1973-1993 | 15+ | 13000 (est) | 4100000 | Age, sex | Up to 21 | CVD | P, M, F | SMR |
| Hoang, 2011 | SCZ, BD | UK | Inpatient | 1999-2006 | 2000-2007 | Median: SCZ: 36 (M), 44 (F), BD: 44 (M), 48 (F) | 283227 patients discharged over study period (may be included more than once) | Gen pop | Age, sex | 1 | CVD | P | SMR |
| Høye, 2011 | SCZ | Norway | Inpatient | 1980-1992 | 1980-2006 | Mean 35.2 | 1111 | Gen pop | Age, sex, year | 14.5 | CVD | P, M, F | SMR |
| John, 2018 | SMI | UK | Community and inpatient | 2004-2013 | 2004-2013 | <45, 45-64 | 22359 | NR | Age, sex, calendar year | 5.1 | CVA, CHD, CVD | P | SMR |
| Kelly, 2010 | SCZ | USA | Community and inpatient | 1994-May 2000 | 1994-2004 | 20-69, mean 39 | 1686 | Gen pop | Age, sex, race | 6 to 10 | CHD | P, M, F | SMR |
| Kilbourne, 2009 | SCZ, BD | USA | Community | 1998 - 1999 | 2000-2006 | Mean SCZ: 56, BD: 55.5 | 38020 | 16072 | Age, sex, race, education, marital status, social support, financial hardship, living alone, service connected disability, non-VA care | Up to 8 | CHD | P | HR |
| Kiviniemi, 2010 | SCZ | Finland | Inpatient | 1995-2001 | 2000s | Mean 33.5 | 7591 | Gen pop | Age, sex, district, year | 5 | CVD | P, M, F | SMR |
| Kredentser, 2014 | SCZ | Canada | Community and inpatient | 1987-1998 | 1999-2008 | 10+, Mean 48.7 | 9038 | 969090 | Age, sex | Up to 10 | CVD | P | RRatio |
| Lahti, 2012 | SCZ | Finland | Inpatient | 1969-2004 | 1969-2004 | 25-70, median 35.3 | 204 | 12735 | Birth year, sex, socioeconomic status in childhood | Up to 35 | CVA, CHD | P, M, F | HR |
| Laursen, 2007 | SCZ, BD | Denmark | Inpatient | 1973-2000 | 1973-2000 | 15+ | 29308 | 5191544 | Age, sex | Up to 27 | CHD | M, F | RRatio |
| Laursen, 2010 | SCZ, BD | Denmark | Inpatient | 1994-2006 | 1994-2006 | 15+ | NR | NR | Age, sex | Up to 13 | CHD | P, M, F | RRatio |
| Laursen, 2013 | SCZ, BD | Denmark, Finland, Sweden | Inpatient | 1987-1999 | 2000-2006 | 15+ | 105463 | Gen pop | Age, sex | Up to 20 | CVA, CHD, CVD | P, M, F | SMR |
| Laursen, 2014 | SCZ, BD | Denmark | Community and inpatient | 1995-2006 | 1998-2008 | 10+ | 4500 (est) | 1061532 | Age, sex, time, Charlson index | Up to 14 | CHD | P | RRatio |
| Laursen, 2019 | SCZ | Denmark | Community and inpatient | 1995-2015 | 1995-2015 | 10+ | NR | NR | Age, sex, calender time | Up to 21 | CVD | M, F | RRatio |
| Lawrence, 2003 | SCZ | Australia | Community and inpatient | 1980-1998 | 1980-1998 | NR | NR | NR | Age, sex, year | Up to 18 | CHD | M, F | RRatio |
| Lawrence, 2013 | SCZ | Australia | Community and inpatient | 1985-2005 | 1985-2005 | NR | NR | NR | Age, sex, year | Up to 20 | CVA, CHD | P | SMR |
| Lemogne, 2013 | SMI | France | Community | 1978-2010 | 1990-2010 | 35-50, mean 44 | 124 | 16261 | Age, sex, occupational grade, alcohol, smoking, BMI | 19.8 | CVD | P | HR |
| Lesage, 2015 | SCZ | Canada | Community | Apr 1999 to Mar 2012 | Apr 1999 to Mar 2012 | NR | 33660 (annual est) | 7525000 | Age, sex | Up to 12 | CVD | M, F | RRatio |
| Lumme, 2016 | SCZ, BD | Finland | Inpatient | 1990-2010 | 1996-2010 | 25-64 | NR | NR | Age, sex, year | Up to 20 | CHD | M, F | RRatio |
| Manderbacka, 2012 | SCZ | Finland | Inpatient | 1998-2009 | 1998-2009 | 40+ | 67659 | Gen pop | Age, sex, year | Up to 12 | CHD | P | RRatio |
| Morden, 2012 | SCZ | USA | Community and inpatient | Oct 1999 to Sep 2007 | Oct 1999 to Sep 2007 | Mean 53.4 | 65362 | 65362 | Age, sex, location | Up to 8 | CHD, CVD | P | RRatio |
| Mortensen, 1990 | SCZ | Denmark | Inpatient | 21089 | 1957-1986 | NR | 6178 | Gen pop | Age, sex, calendar period | Up to 29 | CVA, CHD | P, M, F | SMR |
| Mortensen, 1993 | SCZ | Denmark | Inpatient | Apr 1970-1987 | 1970-Mar 1988 | NR | 9156 | Gen pop | Age, sex, calendar period | Up to 18 | CVA, CHD | P, M, F | SMR |
| Murray-Thomas, 2013 | SCZ | UK | Community | 1995-Jan 2011 | 2001-Jan 2011 | 18+ | 7779 | NR | Age, sex, year, GP practice | 4.1 | CHD | P | RRatio |
| Newman, 1991 | SCZ | Canada | Community and inpatient | 1976-1985 | 1976-1985 | 9-88, mean 37.3 | 3623 | Gen pop | Age, sex | 6 | CVA, CHD, CVD | P, M, F | SMR |
| Nilsson, 1995 | BD | Sweden | Inpatient | 1970-1977 | 1970-Jul 1991 | Mean 48 | 362 | Gen pop | Age, sex | 14.3 | CVA, CHD | P, M, F | SMR |
| Nordentoft, 2013 | SCZ | Denmark, Finland, Sweden | Inpatient | 2000-2006 | 2000-2006 | NR | 40970 | Gen pop | Age, sex | Up to 9 | CVD | M, F | SMR |
| Odegard, 1967 | SCZ | Norway | Inpatient | 1950-1962 | 1950-1962 | 15+ | 5200 approx | Gen pop | Age, sex | Up to 12 years of death, but not followed up | CVD | M, F | RRatio |
| Olfson, 2015 | SCZ | USA | Community and inpatient | 2001-2007 | 2001-2007 | 20-64 | 1138853 | Gen pop | Age, sex, ethnicity/race, geographic region | 4.2 | CVA, CHD, CVD | P, M, F | SMR |
| Osborn, 2007 | SCZ, BD | UK | Community | June 1987-Apr 2002 | June 1987-Apr 2002 | 18+ | 29297 | 300246 | Age, sex, calendar period, smoking, social deprivation | SMI: 4.7, controls: 4.3 | CVA, CHD | P | HR |
| Ösby, 2000a | SCZ | Sweden | Inpatient | 1973-1995 | 1973-1995 | All ages | 7784 | Gen pop | Age, sex, calendar period | Up to 23 | CVA, CHD | P, M, F | SMR |
| Ösby, 2000b | SCZ | Sweden | Inpatient | 1976-1995 | 1976-1995 | NR | 5802 (first admissions) | Gen pop | Age, sex, calendar period | Up to 20 | CVD | M, F | SMR |
| Ösby, 2001 | BD | Sweden | Inpatient | 1973-1995 | 1973-1995 | <70, mean 43.1 | 15386 | Gen pop | Age, sex, calendar period | 10.5 (M), 11.5 (F) | CVA, CHD | P, M, F | SMR |
| Ösby, 2016 | SCZ, BD | Sweden | Inpatient | 1987-2010 | 1987-2010 | 15+, mean SCZ: 46.3, BD: 50.9 | 121875 hospitalisations (patients may be included more than once) | Gen pop | Age, sex, calendar year, length of follow-up | 2 | CVA, CHD, CVD | P | RRatio |
| Pan, 2020 | SCZ, BD | Taiwan | Community and inpatient | 2005 | 2005-2008 | Mean 49.95 | 162878 | Gen pop | Age, sex, calendar period | Up to 3 | CVD | P, M, F | SMR |
| Park, 2015 | SCZ | South Korea | Community and inpatient | 1995-2006 | 1995-2009 | 18+ | NR | NR | Age (sex?) | Up to 15 | CVD | P | SMR |
| Prior, 1996 | SCZ, BD | UK | Community and inpatient | 1974-1984 | 1974-1985 | 15-89 | 1835 | Gen pop | Age, sex | Up to 11 | CVD | P, M, F | SMR |
| Saku, 1995 | SCZ, BD | Japan | Inpatient | 1948-Mar 1982 | 1948-Aug 1985 | All ages | 2455 | Gen pop | Age, sex, year | Up to 36 | CVA, CHD | P, M, F | SMR |
| Salazar-Fraile, 1998 | SCZ | Spain | Community | April-Nov 1986 | 1986-1993 | 16+ | 186 | 2382 | Age, sex | Up to 11 | CVD | P | HR |
| Sanchez, 2021 | SCZ | Spain | Community | 2006-2007 | 2008-2011 | 18+, mean 43.4 | 6472 | 94694 | Age, sex, primary care utilisation, alcohol, HTN, DM, obesity, dyslipidaemia, smoking (mortality excludes alcohol, obesity, DM but included cocaine use) | Up to 5 | CVD | P | HR |
| Tanskanen, 2018 | SCZ | Finland | Inpatient | 1984-2014 | 1984-2014 | 16+ | 42343 | 4515838 | Age, sex | Up to 30 | CVD | P | SMR |
| Termorshuizen, 2013 | SCZ | Netherlands | Community and inpatient | 2000-2008 | 2000-2010 | 18-64 | NR | NR | Age, sex, ethnicity | <2, 2-5, >5 | CVD | P | RRatio |
| Torniainen, 2015 | SCZ | Sweden | Community and inpatient | 1-Jan-06 | 2006-2010 | 17-65, mean 46 | 21492 | 214920 | Age, sex | 5 | CVD | P | HR |
| Tsuang, 1980 | SCZ, BD | USA | Inpatient | 1934-1944 | 1934-1974 | NR | 300 | Gen pop | Age, sex | Up to 40 | CVD | P, M, F | SMR |
| Vance, 2019 | SCZ, BD | USA | Community | 2009 | 2010-2014 | 45-80, mean 61.6 | NR | NR | Age, sex, smoking, race, BP, cholesterol, HDL, HTN medication, DM | Up to 5 | CVD | M, F | OR |
| Weeke, 1986 | BD | Denmark | Inpatient | Apr 1970- Mar 1972 | Apr 1970-Mar 1977 | Mean 50.5 | 2168 | Gen pop | Age, sex | Up to 7 | CVD | P, M, F | SMR |
| Weeke, 1987 | BD | Denmark | Inpatient | 1950-1956, 1969-1976 | 1950-1957, 1969-1977 | Mean 46.1 | 3431 | Gen pop | Age | 4.5 | CVD | M | SMR |
| Westman, 2013 | BD | Sweden | Inpatient | 1987-2006 | 1987-2006 | All ages | 17101 | 10631208 | Age, sex, calendar year, year of follow-up | Up to 20 | CVA, CHD, CVD | P, M, F | RRatio |
| Westman, 2017 | SCZ | Sweden | Inpatient | 1987-2010 | 1987-2010 | 15+ | 46911 | 10631817 | Age, sex, calendar period | Up to 24 | CVA, CHD, CVD, HF | P, M, F | RRatio |
| Yung, 2021 | SCZ | Hong Kong | Community and inpatient | 2001-2016 | 2006-2016 | 18+ | 46896 | Gen pop | Age, sex, calendar year | 8.7 | CVA, CHD, CVD | P, M, F | SMR |
| Zilber, 1989 | SCZ | Israel | Inpatient | 1978 | 1978-1983 | 10+ | 8900 (est) | Gen pop | Age, sex, year | 5.15 | CVD | P | SMR |

SCZ – schizophrenia, BD – bipolar disorder, NR – not reported, est – estimated, gen pop – general population, DM – diabetes, HT – hypertension, COPD – chronic obstructive pulmonary disease, TIA – transient ischaemic attack, BMI – body mass index, CVA – cerebrovascular accident, CHD – coronary heart disease, CVD – all circulatory disease, HF – heart failure, P – persons, M – males, F – females, HR – hazard ratio, RRatio – rate ratio, SMR – standardised mortality ratio
