# Supplementary material for: Temporal trends in associations between severe mental illness and risk of cardiovascular disease: A systematic review and meta-analysis
Source: PLoS Med. 2022 Apr 19;19(4):e1003960. doi: 10.1371/journal.pmed.1003960 (PMC9017899; doi:10.1371/journal.pmed.1003960)
Supplement: S13 File — (DOCX) [file pmed.1003960.s013.docx]

# S13 File. Characteristics of included incidence studies

| **Study, year** | **SMI diagnosis** | **Country** | **Setting** | **Period of SMI exposure** | **Period of outcomes** | **Age at start of study (years)** | **Number of cases** | **Number of controls** | **Covariates adjusted for** | **Mean follow up (years)** | **Outcomes** | **Sex** | **Type of results** |
| --- | --- | --- | --- | --- | --- | --- | --- | --- | --- | --- | --- | --- | --- |
| Bent-Ennakhil, 2018 | SCZ, BD | Sweden | Community and inpatient | 1980-2012 | 1980-2012 | 18+, mean SCZ: 42.7, BD: 44 | 4823 | 200486 | Age, sex | Up to 32, mean time to event: SCZ 8.95, BD 10.6, controls 5.95 | CVD | P, M, F | RRatio |
| Brink, 2018 | SCZ | Denmark | Inpatient | 1970-1979 | 1980-2012 | 18-40 | 4544 | 22597 | Age, sex, year, Charlson score, education, marital status | Up to 41 | CVD | P | RRatio |
| Carney, 2006 | SCZ | USA | Community and inpatient | 1996-2001 | 1996-2001 | 18-64, mean 40.2 | 1074 | 726262 | Age, sex, urbanization, healthcare utilization | (3.3 cases, 2.2 controls) | CVA, CHD, HF | P | OR |
| Chen, 2015 | SCZ | Taiwan | Community and inpatient | 2002-2008 | 2002-2011 | Mean 36.5 | 63913 | 63913 | Age, sex, enrolment time | 3 to 10 | CVA | P | RR |
| Crump, 2013a | SCZ | Sweden | Community and inpatient | 2001-2002 | 2003-2009 | 25+ | 8277 | 6097834 | Age, sex, marital status, education, employment status, income | 7 | CVA, CHD, CVD | M, F | HR |
| Crump, 2013b | BD | Sweden | Community and inpatient | 2001-2002 | 2003-2009 | 20+ | 6618 | 6587036 | Age, sex, marital status, education, employment status, income | 7 | CVA, CHD, CVD | M, F | HR |
| Curkendall, 2004 | SCZ | Canada | Community | 1994-1995 | 1996-March 1999 | 18+, mean 49.6 | 2405 | 9175 | Age, sex, >1 of: HT, hyperlipidaemia, DM, CVD, COPD | NR | CVA, CHD, HF | P | RR |
| Foroughi, 2018 | BD | USA | Community | 1998-2000 | 1998-2018? | Mean 53.8 | 378 | 23251 | Age, sex, CVD risk | 16.1 | CVD | P | HR |
| Foroughi, 2021 | BD | USA | Community | 1998-2003 | 1998- Feb 2016 | Mean 49.8 | 288 | 35326 | Age, sex, smoking, DM, CKD, HTN, alcohol, BMI, HDL, substance abuse, major depression | 16.5 (median), 14.6-17.5 IQR | CVD | P | HR |
| Gale, 2013 | SCZ, BD | Sweden | Inpatient | 1969-1994 | 1969-2004 | Mean 18.3 | 8527 | 1099304 | Age, sex, parental SES, BMI, DM, BP, intelligence | NR | CHD | M | HR |
| Goldstein, 2014 | BD | USA | Community | 2001-2002 | 2004-2005 | 18+, mean 36.6 | 1439 | 26266 | Age, sex, race, smoking, HT, obesity, alcohol, drug use | 3.08 | CVD | P | OR |
| Gur, 2017 | SCZ | Israel | Community and inpatient | 2005-2013 | 2005-2013 | Mean 37.5 | 1389 | 4095 | Age, sex, SES | Up to 9 | CHD, HF | P | OR |
| Hayes, 2017 | SCZ, BD | UK | Community | 2000-2014 | 2000-2014 | 16+, median SCZ: 42.5, BD: 42.8 | 39838 | 219387 | Age, sex, calendar period, deprivation, ethnicity, primary care contacts | median: SCZ 2.47, BD 2.32 | CVD | P | HR |
| Hsu, 2021 | BD | Taiwan | Community and inpatient | 2005 | 2006-2010 | 18+ | 5544 | 764579 | Age, sex, income, region, urbanicity | 4.3 | CHD | P, M, F | RRatio |
| Jackson, 2020 | SCZ, BD | UK | Inpatient | 1991-2015 | 1991-2015 | 40+ | NR | NR | Age, sex | Up to 25 | CVA, CHD | M, F | RRatio |
| Jakobsen, 2008 | SCZ | Denmark | Inpatient | 1977-2000 | 1977-2001 | 15+ | 15710 | 69447 | Age, sex | Up to 24 | CHD | P | RRatio |
| Kessing, 2021 | BD | Denmark | Community and inpatient | 1995-2017 | 1995-2017 | Median 44.8 | 18981 | 366420 | Age, sex, calendar year, employment | Up to 22 | CHD | P | HR |
| Kugathasan, 2018 | SCZ | Denmark | Community and inpatient | 1980-2015 | 1980-2015 | 18+, mean 33.9 | 36962 | 184810 | Age, sex | Up to 36 years | CHD | P | RR |
| Lahti, 2012 | SCZ | Finland | Inpatient | 1969-2004 | 1969-2004 | 25-70, median 35.3 | 204 | 12735 | Birth year, sex, childhood SES | Up to 35 | CVA, CHD | P, M, F | HR |
| Laursen, 2010 | SCZ, BD | Denmark | Inpatient | 1994-2006 | 1994-2006 | 15+ | NR | NR | Age, sex | Up to 13 | CHD | P, M, F | RRatio |
| Laursen, 2011 | SCZ, BD | Denmark | Inpatient | 1995-Jun 2007 | 1995-Jun 2007 | 15-52 | 22294 | 2428518 | Age, sex, year | Up to 12.5 | CVA, CHD, HF | P | RRatio |
| Lawrence, 2003 | SCZ | Australia | Community and inpatient | 1966-1998 | 1980-1998 | NR | NR | NR | Age, sex, year | Up to 18 | CHD | M, F | RRatio |
| Lin, 2010 | SCZ | Taiwan | Community and inpatient | 2000 | 2000-2006 | 18+ | 7353 | 22059 | Age, sex, HT, DM, hyperlipidaemia, income, urbanization, region | 6 | CHD | P | HR |
| Manderbacka, 2012 | SCZ | Finland | Inpatient | 1998-2009 | 1998-2009 | 40+ | 67659 | Gen pop | Age, sex, year | Up to 12 | CHD | P | RRatio |
| McDermott, 2005 | SCZ, BD | USA | Community | 1990-2003 | 1990-2003 | Mean SCZ: 45.6, BD: 43.8 | 503 | 2083 | Age, sex, race, smoking, HT, obesity, depression, coronary artery disease/congestive HF/TIA, dementia, DM | SCZ 7.9, BD 7.5, controls 6.7 | CVA, CHD, HF | P | RR |
| Momen, 2020 | SCZ | Denmark | Community and inpatient | 1969-2015 | 2000-2016 | Median 32.1 | NR | NR | Age, sex, calendar time, other mental disorder | 14.1 | CVA, CHD | P, M, F | HR |
| Morden, 2012 | SCZ | USA | Community and inpatient | Oct 1999 to Sep 2007 | Oct 1999 to Sep 2007 | Mean 53.4 | 65362 | 65362 | Age, sex, location | Up to 8 | CHD, CVD | P | RRatio |
| Munk-Jorgensen, 2000 | SCZ | Denmark | Inpatient | 1-Jan-78 | 1978-1993 | NR | 20495 | 204912 | Age, sex | Up to 16 | CVA, CHD | P | RRatio |
| Prieto, 2016 | BD | USA | Community and inpatient | 1966-1996 | 1966-2013 | Median 37 | 334 | 334 | Age, sex, alcohol, HT, DM, smoking | 20 (cases), 15 (controls) | CVA, CHD, CVD | P | HR |
| Ramsey, 2010 | BD | USA | Community | 1981-1982 | 1993-1996 | Mean 43.1 | 58 | 1339 | Age, sex, education, marital status, depression | 11.5 | CHD | P | OR |
| Sanchez, 2021 | SCZ | Spain | Community | 2006-2007 | 2008-2011 | 18+, Mean 43.4 | 6472 | 94694 | Age, sex, primary care utilisation, alcohol, HT, DM, obesity, dyslipidaemia, smoking | Up to 5 | CVD | P | HR |
| Sundquist, 2006 | SCZ | Sweden | Inpatient | 1987-2001 | 1987-2001 | 25-64 | NR | NR | Age, sex, SES, region, time period | Up to 15 | CHD | M, F | SIR |
| Tsai, 2012 | SCZ | Taiwan | Community and inpatient | 1999-2003 | 2004-2008 | Mean 56.8 | 80569 | 241707 | Age, sex, socioeconomic status, diabetes, hypertension, hyperlipidaemia | Up to 5 years | CVA | P | RR |
| Vance, 2019 | SCZ, BD | USA | Community | 2009 | 2010-2014 | 45-80, Mean 61.6 | NR | NR | Age, sex, smoking, race, BP, cholesterol, HDL, HT medication,, DM | Up to 5 | CVD | M, F | OR |
| Westman, 2013 | BD | Sweden | Inpatient | 1987-2006 | 1987-2006 | All ages | 17101 | 10631208 | Age, sex, calendar year, year of follow up | Up to 20 years | CVA, CHD, CVD | P, M, F | RRatio |
| Westman, 2017 | SCZ | Sweden | Inpatient | 1987-2010 | 1987-2010 | 15+ | 46911 | 10631817 | Age, sex, calendar period | Up to 24 years | CVA, CHD, CVD, HF | P, M, F | RRatio |
| Wium-Andersen, 2021 | BD | Denmark | Community and inpatient | 1997-2014 | 1997-2018 | 18+, Median 43.8 | 13725 | 167562 | Age, sex, calendar time, marital status, education, statin, NSAID, acetylsalicylic acid, paracetamol, anticontraception pills, antidepressant medication, antipsychotic medication, lithium, benzodiazepines | 9.5 (range 0-21) | CVA, CHD | P | HR |
| Wu, 2013 | BD | Taiwan | Community and inpatient | 1999-2003 | 2004-2010 | Mean 43.2 | 16821 | 67284 | Age, sex, DM, HT, hyperlipidaemia, SES, urbanization, CHD | 7 | CVA | P | RR |
| Wu, 2015 | SCZ, BD | Taiwan | Community and inpatient | 1996-2007 | 1996-2007 | 18+ | 70225 | 207592 | Age, sex, income, urbanization | 11 | CHD | P, M, F | HR |
| Yu-Chuan Chiu, 2015 | SCZ, BD | Taiwan | Community and inpatient | 1995-2011 | 1998-2011 | 18-45 | 115 | 10200 | Age, sex, HT, dyslipidaemia, obesity, DM, renal, IHD, arrhythmia, urbanization, income | Up to 14 | CVA | P | OR |

SCZ – schizophrenia, BD – bipolar disorder, NR – not reported, est – estimated, gen pop – general population, DM – diabetes, HT – hypertension, COPD – chronic obstructive pulmonary disease, TIA – transient ischaemic attack, BMI – body mass index, SES – socioeconomic status, CVA – cerebrovascular accident, CHD – coronary heart disease, CVD – all circulatory disease, HF – heart failure, P – persons, M – males, F – females, HR – hazard ratio, RRatio – rate ratio, OR – odds ratio, RR – risk ratio, SIR – standardised incidence ratio
