# Supplementary material for: Temporal trends in associations between severe mental illness and risk of cardiovascular disease: A systematic review and meta-analysis
Source: PLoS Med. 2022 Apr 19;19(4):e1003960. doi: 10.1371/journal.pmed.1003960 (PMC9017899; doi:10.1371/journal.pmed.1003960)
Supplement: S14 File — Fig A: Schizophrenia, mortality from CVA, SMRs. Fig B: Schizophrenia, mortality from CHD, SMRs. Fig C: Schizophrenia, mortality from all circulatory disease, SMRs. Fig D: BD, mortality from CVA, SMRs. Fig E: BD, mortality from CHD, SMRs. Fig F: BD, mortality from all circulatory disease, SMRs. Fig G: Schizophrenia, mortality from CVA, HRs, rate ratios and ORs. Fig H: Schizophrenia, mortality from CHD, HRs, rate ratios and ORs. Fig I: Schizophrenia, mortality from all circulatory disease, HRs, rate ratios and ORs. Fig J: Schizophrenia, mortality from heart failure, HRs, rate ratios and ORs. Fig K: BD, mortality from CVA, HRs, rate ratios and ORs. Fig L: BD, mortality from CHD, HRs, rate ratios and ORs. Fig M: BD, mortality from all circulatory disease, HRs, rate ratios and ORs. Fig N: Mixed SMI, mortality from CVDs, HRs, rate ratios, ORs, SMRs. BD, bipolar disorder; CHD, coronary heart disease; CVA, cerebrovascular accident; HR, hazard ratio; OR, odds ratio; SMR, standardised mortality ratio. (PDF) [file pmed.1003960.s014.pdf]

S14 File. Forest plots reporting cardiovascular mortality outcomes, across decades

Study identifier	Gender	Type of effect	Region	Setting	Events/Total Exposed	Events/Total Unexposed	Effect size (95% CI)
**1950s - 70s**							
Allebeck, 1986	P	SMR	Scandinavia	Inpatient	NR / 1190	NR / Gen pop	2.00 (1.24, 3.21)
Giel, 1978	P	SMR	Other Europe/Israel	Inpatient	9 / 4172	3 / Gen pop	3.00 (1.58, 5.71)
Mortensen, 1990	P	SMR	Scandinavia	Inpatient	326 / 6178	472 / Gen pop	0.69 (0.62, 0.77)
Mortensen, 1993	P	SMR	Scandinavia	Inpatient	29 / 9156	24 / Gen pop	1.23 (0.86, 1.78)
Saku, 1995	P	SMR	Far East	Inpatient	32 / 2268	32 / Gen pop	1.00 (0.71, 1.41)
**1980s**							
Anderson, 1991	P	SMR	UK	Inpatient	7 / 532	2 / Gen pop	3.71 (1.77, 7.79)
Brook, 1984	P	SMR	Other Europe/Israel	Inpatient	52 / 5226	36 / Gen pop	1.44 (1.10, 1.90)
Newman, 1991	P	SMR	N. America	Community and inpatient	10 / 3623	8 / Gen pop	1.30 (0.70, 2.41)
Osby, 2000a	P	SMR	Scandinavia	Inpatient	107 / 7784	63 / Gen pop	1.70 (1.41, 2.06)
**1990s**							
Brown, 2010 (1981-2006)	P	SMR	UK	Community	13 / 370	4 / Gen pop	3.08 (1.72, 5.52)
Callaghan, 2014	P	SMR	N. America	Inpatient	2887 / 172722	1375 / Gen pop	2.10 (2.00, 2.20)
Casadebaig, 1999	P	SMR	Other Europe/Israel	Community and inpatient	3 / 3470	2 / Gen pop	2.00 (0.65, 6.18)
Lawrence, 2013	P	SMR	Oceania	Community and inpatient	256 / NR	81 / Gen pop	3.16 (2.79, 3.58)
**2000s**							
Castagnini, 2013	P	SMR	Scandinavia	Community and inpatient	2 / 4576	2 / Gen pop	1.20 (0.30, 4.75)
Laursen, 2013 (Denmark)	P	SMR	Scandinavia	Inpatient	108 / 20430	62 / Gen pop	1.74 (1.44, 2.10)
Laursen, 2013 (Finland)	P	SMR	Scandinavia	Inpatient	184 / 20835	70 / Gen pop	2.61 (2.26, 3.02)
Laursen, 2013 (Sweden)	P	SMR	Scandinavia	Inpatient	280 / 24823	123 / Gen pop	2.28 (2.03, 2.57)
Olfson, 2015	P	SMR	N. America	Community and inpatient	1561 / 1138853	710 / Gen pop	2.20 (2.10, 2.30)
**2010s**							
Girardi, 2021	P	SMR	Other Europe/Israel	Community and inpatient	94 / 12996	45 / Gen pop	2.10 (1.70, 2.60)
Yung, 2021	P	SMR	Far East	Community and inpatient	214 / 46896	188 / Gen pop	1.14 (0.99, 1.31)

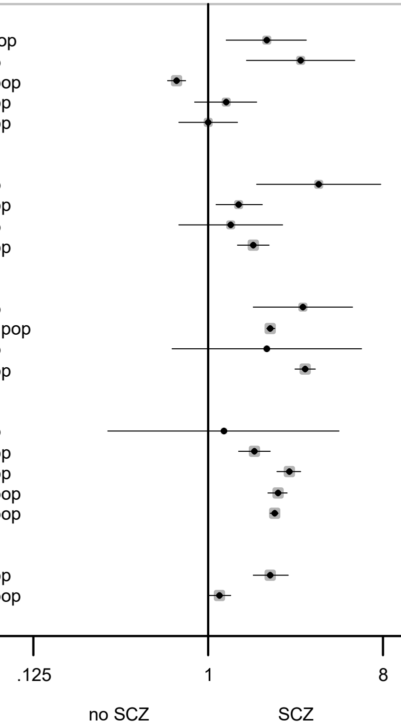

SCZ – schizophrenia, NR – not reported, gen pop – general population, SMR – standardised mortality ratio, P – persons

Fig A: Schizophrenia, mortality from cerebrovascular accident, standardised mortality ratios

Study identifier	Gender	Type of effect	Region	Setting	Events/Total Exposed	Events/Total Unexposed	Effect size (95% CI)
**1950s - 70s**							
Allebeck, 1986	P	SMR	Scandinavia	Inpatient	NR / 1190	NR / Gen pop	1.70 (1.31, 2.21)
Giel, 1978	P	SMR	Other Europe/Israel	Inpatient	31 / 4172	18 / Gen pop	1.72 (1.22, 2.44)
Mortensen, 1990	P	SMR	Scandinavia	Inpatient	1596 / 6178	1438 / Gen pop	1.11 (1.06, 1.17)
Mortensen, 1993	P	SMR	Scandinavia	Inpatient	128 / 9156	85 / Gen pop	1.51 (1.27, 1.80)
Saku, 1995	P	SMR	Far East	Inpatient	37 / 2268	21 / Gen pop	1.79 (1.29, 2.48)
**1980s**							
Anderson, 1991	P	SMR	UK	Inpatient	13 / 532	7 / Gen pop	1.94 (1.13, 3.35)
Brook, 1984	P	SMR	Other Europe/Israel	Inpatient	40 / 5226	69 / Gen pop	0.58 (0.41, 0.82)
Newman, 1991	P	SMR	N. America	Community and inpatient	36 / 3623	26 / Gen pop	1.40 (1.02, 1.93)
Osby, 2000a	P	SMR	Scandinavia	Inpatient	563 / 7784	257 / Gen pop	2.19 (2.01, 2.38)
**1990s**							
Brown, 2010 (1981-2006)	P	SMR	UK	Community	37 / 370	16 / Gen pop	2.25 (1.61, 3.15)
Callaghan, 2014	P	SMR	N. America	Inpatient	10861 / 172722	4023 / Gen pop	2.70 (2.60, 2.80)
Casadebaig, 1999	P	SMR	Other Europe/Israel	Community and inpatient	7 / 3470	3 / Gen pop	2.41 (1.15, 5.06)
Kelly, 2010 (Clozapine)	P	SMR	N. America	Community	32 / 1084	7 / Gen pop	5.01 (3.46, 7.24)
Kelly, 2010 (Risperidone)	P	SMR	N. America	Inpatient	11 / 602	4 / Gen pop	2.80 (1.43, 5.48)
Lawrence, 2013	P	SMR	Oceania	Community and inpatient	452 / NR	197 / Gen pop	2.29 (2.10, 2.50)
**2000s**							
Castagnini, 2013	P	SMR	Scandinavia	Community and inpatient	22 / 4576	5 / Gen pop	4.20 (2.78, 6.35)
Laursen, 2013 (Sweden)	P	SMR	Scandinavia	Inpatient	746 / 24823	289 / Gen pop	2.58 (2.40, 2.77)
Laursen, 2013 (Finland)	P	SMR	Scandinavia	Inpatient	511 / 20835	201 / Gen pop	2.55 (2.34, 2.78)
Laursen, 2013 (Denmark)	P	SMR	Scandinavia	Inpatient	215 / 20430	119 / Gen pop	1.81 (1.58, 2.06)
Olfson, 2015	P	SMR	N. America	Community and inpatient	10096 / 1138853	2729 / Gen pop	3.70 (3.60, 3.80)
**2010s**							
Girardi, 2021	P	SMR	Other Europe/Israel	Community and inpatient	227 / 12996	70 / Gen pop	3.20 (2.82, 3.63)
Yung, 2021	P	SMR	Far East	Community and inpatient	367 / 46896	243 / Gen pop	1.51 (1.36, 1.67)

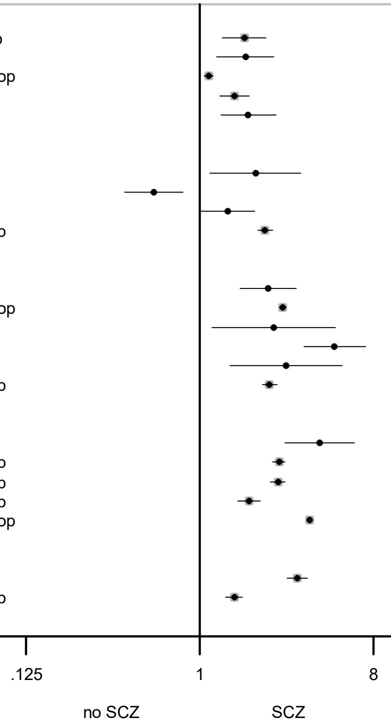

SCZ – schizophrenia, NR – not reported, gen pop – general population, SMR – standardised mortality ratio, P – persons

Fig B: Schizophrenia, mortality from coronary heart disease, standardised mortality ratios

Study identifier	Gender	Type of effect	Region	Events/Total Exposed	Events/Total Unexposed	Effect size (95% CI)
**1950s - 70s**						
Buda, 1988	P	SMR	N. America	46 / 332	22 / Gen pop	2.08 (1.56, 2.77)
Osby, 2000b (M)	M	SMR	Scandinavia	NR / NR	NR / NR	1.70 (1.26, 2.30)
Osby, 2000b (F)	F	SMR	Scandinavia	NR / NR	NR / NR	1.70 (1.39, 2.08)
Prior, 1996	P	SMR	UK	38 / 745	30 / Gen pop	1.28 (0.93, 1.76)
Tsuang, 1980	P	SMR	N. America	23 / 200	18 / Gen pop	1.28 (0.85, 1.93)
**1980s**						
Hiroeh, 2008	P	SMR	Scandinavia	996 / 13,000 (est)	568 / 4100000	1.75 (1.65, 1.86)
Newman, 1991	P	SMR	N. America	61 / 3623	44 / Gen pop	1.40 (1.09, 1.79)
Osby, 2000b (M)	M	SMR	Scandinavia	NR / NR	NR / NR	4.20 (2.92, 6.04)
Osby, 2000b (M)	M	SMR	Scandinavia	NR / NR	NR / NR	2.00 (1.44, 2.78)
Osby, 2000b (F)	F	SMR	Scandinavia	NR / NR	NR / NR	2.10 (1.62, 2.73)
Osby, 2000b (F)	F	SMR	Scandinavia	NR / NR	NR / NR	3.10 (2.17, 4.44)
Zilber, 1989	P	SMR	Other Europe/Israel	52 / 8900 (est)	144 / Gen pop	0.36 (0.27, 0.47)
**1990s**						
Ajetunmobi, 2013	P	SMR	UK	3647 / 34892	2218 / Gen pop	1.64 (1.57, 1.71)
Brown, 2010 (1981-2006)	P	SMR	UK	57 / 370	22 / Gen pop	2.58 (1.97, 3.38)
Callaghan, 2014	P	SMR	N. America	16133 / 172722	6558 / Gen pop	2.46 (2.42, 2.51)
Grigoletti, 2009	P	SMR	Other Europe/Israel	NR / 556	NR / Gen pop	1.27 (0.78, 2.06)
Hoye, 2011 (1980-2006)	P	SMR	Scandinavia	89 / 1111	33 / Gen pop	2.74 (2.23, 3.37)
Osby, 2000b (M)	M	SMR	Scandinavia	NR / NR	NR / NR	8.30 (3.65, 18.89)
Osby, 2000b (F)	F	SMR	Scandinavia	NR / NR	NR / NR	5.00 (3.49, 7.15)
Tanskanen, 2018	P	SMR	Scandinavia	416 / 42343	19551 / 4515838	2.56 (2.32, 2.82)
**2000s**						
Cunningham, 2014	P	SMR	Oceania	266 / 22517	76 / Gen pop	3.49 (3.09, 3.93)
Hoang, 2011 (2006)	P	SMR	UK	NR / 24205	NR / Gen pop	2.50 (Insufficient data)
Hoang, 2011 (2004)	P	SMR	UK	NR / 27425	NR / Gen pop	2.20 (Insufficient data)
Hoang, 2011 (2003)	P	SMR	UK	NR / 25856	NR / Gen pop	2.50 (Insufficient data)
Hoang, 2011 (2001)	P	SMR	UK	NR / 25870	NR / Gen pop	1.60 (Insufficient data)
Hoang, 2011 (2000)	P	SMR	UK	NR / 25785	NR / Gen pop	2.00 (Insufficient data)
Hoang, 2011 (2005)	P	SMR	UK	NR / 25709	NR / Gen pop	2.40 (Insufficient data)
Hoang, 2011 (2002)	P	SMR	UK	NR / 25524	NR / Gen pop	1.90 (Insufficient data)
Hoang, 2011 (1999)	P	SMR	UK	NR / 27133	NR / Gen pop	1.60 (Insufficient data)
Kiviniemi, 2010	P	SMR	Scandinavia	85 / 7591	22 / Gen pop	3.92 (3.15, 4.88)
Laursen, 2013 (Sweden)	P	SMR	Scandinavia	1484 / 24823	583 / Gen pop	2.55 (2.42, 2.68)
Laursen, 2013 (Denmark)	P	SMR	Scandinavia	531 / 20430	266 / Gen pop	1.99 (1.83, 2.17)
Laursen, 2013 (Finland)	P	SMR	Scandinavia	943 / 20835	337 / Gen pop	2.80 (2.62, 2.98)
Nordentoft, 2013 (Sweden) (M)	M	SMR	Scandinavia	NR / NR	NR / NR	2.90 (Insufficient data)
Nordentoft, 2013 (Finland) (M)	M	SMR	Scandinavia	NR / NR	NR / NR	2.50 (Insufficient data)
Nordentoft, 2013 (Denmark) (M)	M	SMR	Scandinavia	NR / NR	NR / NR	2.40 (Insufficient data)
Nordentoft, 2013 (Finland) (F)	F	SMR	Scandinavia	NR / NR	NR / NR	2.20 (Insufficient data)
Nordentoft, 2013 (Sweden) (F)	F	SMR	Scandinavia	NR / NR	NR / NR	2.40 (Insufficient data)
Nordentoft, 2013 (Denmark) (F)	F	SMR	Scandinavia	NR / NR	NR / NR	2.30 (Insufficient data)
Olfson, 2015	P	SMR	N. America	19381 / 1138853	5384 / Gen pop	3.60 (3.55, 3.65)
Pan, 2020	P	SMR	Far East	753 / 104561	249 / Gen pop	3.02 (2.81, 3.25)
Park, 2015	P	SMR	Far East	NR / NR	NR / Gen pop	0.55 (0.21, 1.47)
**2010s**						
Girardi, 2021	P	SMR	Other Europe/Israel	560 / 12996	194 / Gen pop	2.90 (2.66, 3.16)
Heiberg, 2018	P	SMR	Scandinavia	350 / 20537	86 / Gen pop	4.06 (3.66, 4.51)
Yung, 2021	P	SMR	Far East	808 / 46896	586 / Gen pop	1.38 (1.29, 1.48)

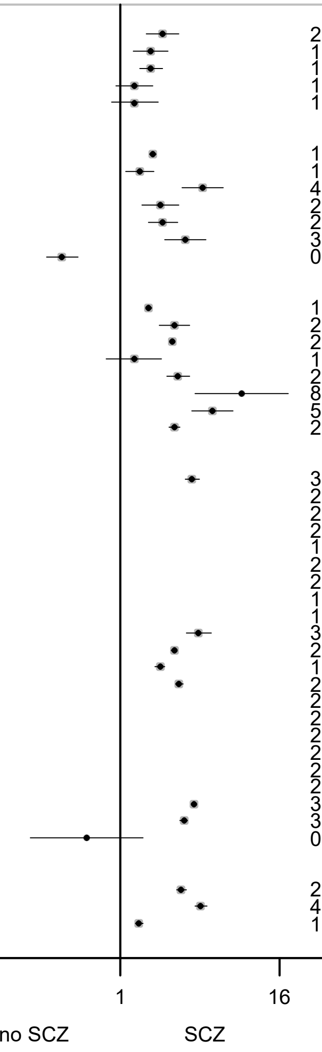

SCZ – schizophrenia, NR – not reported, gen pop – general population, SMR – standardised mortality ratio, P – persons, M – males, F - females

Fig C: Schizophrenia, mortality from all circulatory disease, standardised mortality ratios

Study identifier	Gender	Type of effect	Region	Setting	Events/Total Exposed	Events/Total Unexposed	Effect size (95% CI)
**1950s - 70s**							
Angst, 2002	P	SMR	Other Europe/Israel	Inpatient	21 / 158	15 / Gen pop	1.37 (0.89, 2.10)
Saku, 1995 (M)	M	SMR	Far East	Inpatient	NR / 76	NR / NR	1.28 (Insufficient data)
**1980s**							
Nilsson, 1995	P	SMR	Scandinavia	Inpatient	9 / 362	6 / Gen pop	1.58 (0.82, 3.03)
Osby, 2001	P	SMR	Scandinavia	Inpatient	219 / 15386	111 / Gen pop	1.97 (1.73, 2.25)
**1990s**							
Callaghan, 2014	P	SMR	N. America	Inpatient	672 / 76098	517 / Gen pop	1.30 (1.20, 1.40)
**2000s**							
Castagnini, 2013	P	SMR	Scandinavia	Community and inpatient	8 / 3200	2 / Gen pop	3.50 (1.76, 6.95)
Laursen, 2013 (Finland)	P	SMR	Scandinavia	Inpatient	44 / 9919	23 / Gen pop	1.92 (1.43, 2.59)
Laursen, 2013 (Sweden)	P	SMR	Scandinavia	Inpatient	227 / 18355	132 / Gen pop	1.73 (1.52, 1.97)
Laursen, 2013 (Denmark)	P	SMR	Scandinavia	Inpatient	137 / 11101	79 / Gen pop	1.73 (1.47, 2.05)
**2010s**							
Chan, 2021	P	SMR	Far East	Community and inpatient	36 / 12556	28 / Gen pop	1.30 (0.93, 1.81)

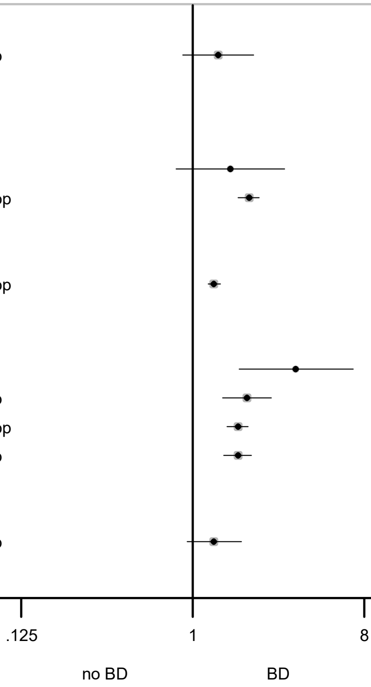

BD – bipolar disorder, NR – not reported, gen pop – general population, SMR – standardised mortality ratio, P – persons, M - males

Fig D: Bipolar disorder, mortality from cerebrovascular accident, standardised mortality ratios

Study identifier	Gender	Type of effect	Region	Setting	Events/Total Exposed	Events/Total Unexposed	Effect size (95% CI)
**1950s - 70s**							
Angst, 2002	P	SMR	Other Europe/Israel	Inpatient	59 / 158	32 / Gen pop	1.84 (1.43, 2.37)
Saku, 1995 (M)	M	SMR	Far East	Inpatient	NR / 76	NR / NR	2.22 (Insufficient data)
Saku, 1995 (F)	F	SMR	Far East	Inpatient	NR / 25	NR / NR	1.18 (Insufficient data)
**1980s**							
Nilsson, 1995	P	SMR	Scandinavia	Inpatient	28 / 362	28 / Gen pop	1.00 (0.69, 1.44)
Osby, 2001	P	SMR	Scandinavia	Inpatient	1073 / 15386	481 / Gen pop	2.23 (2.10, 2.37)
**1990s**							
Callaghan, 2014	P	SMR	N. America	Inpatient	2569 / 76098	1548 / Gen pop	1.66 (1.59, 1.74)
**2000s**							
Castagnini, 2013	P	SMR	Scandinavia	Community and inpatient	15 / 3200	7 / Gen pop	2.10 (1.28, 3.45)
Laursen, 2013 (Denmark)	P	SMR	Scandinavia	Inpatient	206 / 11101	119 / Gen pop	1.72 (1.50, 1.98)
Laursen, 2013 (Sweden)	P	SMR	Scandinavia	Inpatient	455 / 18355	259 / Gen pop	1.76 (1.60, 1.92)
Laursen, 2013 (Finland)	P	SMR	Scandinavia	Inpatient	82 / 9919	41 / Gen pop	1.98 (1.59, 2.46)
**2010s**							
Chan, 2021	P	SMR	Far East	Community and inpatient	47 / 12556	35 / Gen pop	1.33 (1.00, 1.77)

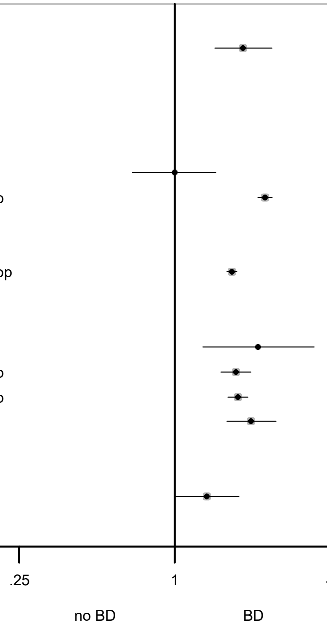

BD – bipolar disorder, NR – not reported, gen pop – general population, SMR – standardised mortality ratio, P – persons, M – males, F - females

Fig E: Bipolar disorder, mortality from coronary heart disease, standardised mortality ratios

Study identifier	Gender	Type of effect	Region	Setting	Events/Total Exposed	Events/Total Unexposed	Effect size (95% CI)
**1950s - 70s**							
Ahrens, 1995	P	SMR	Other Europe/Israel	Inpatient	11 / 440	9 / Gen pop	1.16 (0.59, 2.26)
Angst, 2002	P	SMR	Other Europe/Israel	Inpatient	80 / 158	47 / Gen pop	1.69 (1.36, 2.10)
Prior, 1996	P	SMR	UK	Community and inpatient	71 / 1090	54 / Gen pop	1.31 (1.04, 1.66)
Tsuang, 1980	P	SMR	N. America	Inpatient	24 / 100	18 / Gen pop	1.35 (0.91, 2.01)
Weeke, 1986	P	SMR	Scandinavia	Inpatient	120 / 2168	87 / Gen pop	1.38 (1.16, 1.66)
Weeke, 1987 (1950-1957) (M)	M	SMR	Scandinavia	Inpatient	32 / 1070	17 / NR	1.89 (1.31, 2.73)
Weeke, 1987 (1969-1977) (M)	M	SMR	Scandinavia	Inpatient	87 / 2361	53 / NR	1.65 (1.33, 2.05)
**1980s**							
Brodersen, 2000	P	SMR	Scandinavia	Inpatient	7 / 133	6 / Gen pop	1.23 (0.54, 2.79)
**1990s**							
Ajetunmobi, 2013	P	SMR	UK	Inpatient	929 / 9654	623 / Gen pop	1.49 (1.40, 1.59)
Callaghan, 2014	P	SMR	N. America	Inpatient	3891 / 76098	2494 / Gen pop	1.56 (1.50, 1.62)
**2000s**							
Cunningham, 2014	P	SMR	Oceania	Community and inpatient	67 / 9622	36 / Gen pop	1.86 (1.46, 2.36)
Hoang, 2011 (2006)	P	SMR	UK	Inpatient	NR / 9086	NR / Gen pop	2.50 (Insufficient data)
Hoang, 2011 (2000)	P	SMR	UK	Inpatient	NR / 9217	NR / Gen pop	1.40 (Insufficient data)
Hoang, 2011 (2004)	P	SMR	UK	Inpatient	NR / 10102	NR / Gen pop	1.80 (Insufficient data)
Hoang, 2011 (2003)	P	SMR	UK	Inpatient	NR / 9723	NR / Gen pop	1.80 (Insufficient data)
Hoang, 2011 (2002)	P	SMR	UK	Inpatient	NR / 9612	NR / Gen pop	1.80 (Insufficient data)
Hoang, 2011 (2005)	P	SMR	UK	Inpatient	NR / 9284	NR / Gen pop	2.10 (Insufficient data)
Hoang, 2011 (2001)	P	SMR	UK	Inpatient	NR / 9384	NR / Gen pop	1.60 (Insufficient data)
Hoang, 2011 (1999)	P	SMR	UK	Inpatient	NR / 9312	NR / Gen pop	1.60 (Insufficient data)
Laursen, 2013 (Finland)	P	SMR	Scandinavia	Inpatient	249 / 9919	116 / Gen pop	2.15 (1.90, 2.43)
Laursen, 2013 (Sweden)	P	SMR	Scandinavia	Inpatient	974 / 18355	554 / Gen pop	1.76 (1.65, 1.87)
Laursen, 2013 (Denmark)	P	SMR	Scandinavia	Inpatient	530 / 11101	292 / Gen pop	1.81 (1.67, 1.98)
Pan, 2020	P	SMR	Far East	Community and inpatient	438 / 58317	234 / Gen pop	1.87 (1.70, 2.05)
**2010s**							
Chan, 2021	P	SMR	Far East	Community and inpatient	115 / 12556	86 / Gen pop	1.33 (1.11, 1.60)

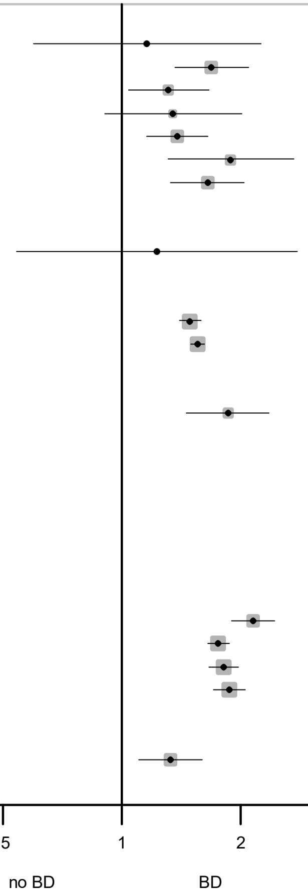

Bipolar disorder, NR – not reported, gen pop – general population, SMR – standardised mortality ratio, P – persons, M - males

Fig F: Bipolar disorder, mortality from all circulatory disease, standardised mortality ratios

Study identifier	Gender	Type of effect	Region	Setting	Events/Total Exposed	Events/Total Unexposed	Effect size (95% CI)
**1980s**							
Lahti, 2012	P	HR	Scandinavia	Inpatient	3 / 204	131 / 12735	1.65 (0.52, 5.22)
Osby, 2016 (1987-1990)	P	RRatio	Scandinavia	Inpatient	NR / 15744	NR / Gen pop	2.61 (Insufficient data)
**1990s**							
Osborn, 2007 (Ages 50-75)	P	HR	UK	Community	44 / Approx 6000	275 / Approx 100000	2.00 (1.45, 2.76)
Osborn, 2007 (Ages 18-49)	P	HR	UK	Community	3 / Approx 11000	16 / Approx 200000	2.87 (0.82, 10.04)
Osby, 2016 (1991-1994)	P	RRatio	Scandinavia	Inpatient	NR / 15096	NR / Gen pop	2.22 (Insufficient data)
Osby, 2016 (1995-1998)	P	RRatio	Scandinavia	Inpatient	NR / 13774	NR / Gen pop	1.97 (Insufficient data)
Westman, 2017 (All ages)	P	RRatio	Scandinavia	Inpatient	985 / 46911	NR / 10631817	2.40 (2.25, 2.55)
Westman, 2017 (Ages 15-59)	P	RRatio	Scandinavia	Inpatient	97 / NR	NR / NR	3.07 (2.51, 3.75)
**2000s**							
Crump, 2013a (M)	M	HR	Scandinavia	Community and inpatient	26 / 2765	12196 / 1446217	2.70 (1.84, 3.97)
Crump, 2013a (F)	F	HR	Scandinavia	Community and inpatient	25 / 1474	17520 / 1604761	2.12 (1.43, 3.14)
Osby, 2016 (2003-2006)	P	RRatio	Scandinavia	Inpatient	NR / 11477	NR / Gen pop	1.86 (Insufficient data)
Osby, 2016 (1999-2002)	P	RRatio	Scandinavia	Inpatient	NR / 11880	NR / Gen pop	2.04 (Insufficient data)
Osby, 2016 (2007-2010)	P	RRatio	Scandinavia	Inpatient	NR / 10940	NR / Gen pop	3.32 (Insufficient data)

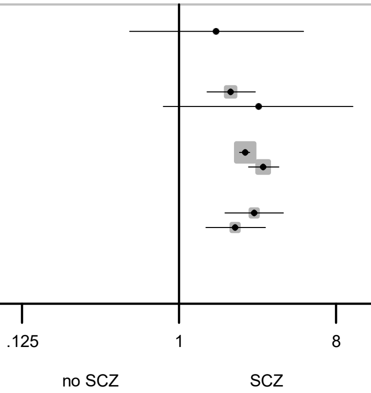

SCZ – schizophrenia, NR – not reported, gen pop – general population, HR – hazard ratio, RRatio – rate ratio, P – persons, M – males, F - females

Fig G: Schizophrenia, mortality from cerebrovascular accident, hazard ratios, rate ratios and odds ratios

Study identifier	Gender	Type of effect	Region	Setting	Events/Total Exposed	Events/Total Unexposed	Effect size (95% CI)
**1980s**							
Heila, 2005 (M)	M	RRatio	Scandinavia	Inpatient	1666 / NR	125573 / NR	2.08 (1.98, 2.18)
Heila, 2005 (F)	F	RRatio	Scandinavia	Inpatient	1146 / NR	104482 / NR	1.63 (1.54, 1.73)
Lahti, 2012	P	HR	Scandinavia	Inpatient	14 / 204	297 / 12735	2.92 (1.70, 5.01)
Laursen, 2007 (M)	M	RRatio	Scandinavia	Inpatient	NR / NR	NR / NR	2.07 (1.85, 2.32)
Laursen, 2007 (F)	F	RRatio	Scandinavia	Inpatient	NR / NR	NR / NR	1.72 (1.53, 1.93)
Lawrence, 2003 (M)	M	RRatio	Oceania	Community and inpatient	NR / NR	NR / NR	1.78 (1.21, 2.61)
Lawrence, 2003 (F)	F	RRatio	Oceania	Community and inpatient	NR / NR	NR / NR	0.86 (0.50, 1.48)
Osby, 2016 (1987-1990)	P	RRatio	Scandinavia	Inpatient	NR / 15744	NR / Gen pop	2.58 (Insufficient data)
**1990s**							
Daumit, 2010	P	RRatio	N. America	Community	NR / 2303 (est)	NR / Gen pop	1.80 (1.70, 1.90)
Laursen, 2010 (1999-2000)	P	RRatio	Scandinavia	Inpatient	150 / NR	24614 / NR	2.42 (2.05, 2.85)
Laursen, 2010 (1997-1998)	P	RRatio	Scandinavia	Inpatient	134 / NR	25488 / NR	2.07 (1.74, 2.46)
Laursen, 2010 (1994-1996)	P	RRatio	Scandinavia	Inpatient	221 / NR	43350 / NR	1.94 (1.69, 2.22)
Lumme, 2016 (1999) (M)	M	RRatio	Scandinavia	Inpatient	56 / NR	3092 / NR	2.91 (2.23, 3.79)
Lumme, 2016 (1996) (M)	M	RRatio	Scandinavia	Inpatient	39 / NR	3434 / NR	2.07 (1.51, 2.84)
Lumme, 2016 (1998) (M)	M	RRatio	Scandinavia	Inpatient	63 / NR	3434 / NR	3.73 (2.91, 4.79)
Lumme, 2016 (1997) (M)	M	RRatio	Scandinavia	Inpatient	53 / NR	3434 / NR	2.83 (2.16, 3.71)
Lumme, 2016 (1998) (F)	F	RRatio	Scandinavia	Inpatient	28 / NR	1138 / NR	3.63 (2.50, 5.28)
Lumme, 2016 (1999) (F)	F	RRatio	Scandinavia	Inpatient	24 / NR	921 / NR	3.67 (2.45, 5.51)
Lumme, 2016 (1996) (F)	F	RRatio	Scandinavia	Inpatient	22 / NR	1138 / NR	2.68 (1.76, 4.09)
Lumme, 2016 (1997) (F)	F	RRatio	Scandinavia	Inpatient	20 / NR	1138 / NR	3.45 (2.22, 5.37)
Osborn, 2007 (Ages 50-75)	P	HR	UK	Community	135 / Approx 6000	896 / Approx 100000	1.96 (1.63, 2.35)
Osborn, 2007 (Ages 18-49)	P	HR	UK	Community	15 / Approx 11000	49 / Approx 200000	3.61 (2.00, 6.50)
Osby, 2016 (1995-1998)	P	RRatio	Scandinavia	Inpatient	NR / 13774	NR / Gen pop	2.42 (Insufficient data)
Osby, 2016 (1991-1994)	P	RRatio	Scandinavia	Inpatient	NR / 15096	NR / Gen pop	2.53 (Insufficient data)
Westman, 2017 (Ages 15-59)	P	RRatio	Scandinavia	Inpatient	596 / NR	NR / NR	6.11 (5.64, 6.62)
Westman, 2017 (All ages)	P	RRatio	Scandinavia	Inpatient	2671 / 46911	NR / 10631817	2.83 (2.73, 2.94)
**2000s**							
Bjorkenstam, 2012 (M)	M	RRatio	Scandinavia	Community and inpatient	79 / 6214	7878 / 1537701	4.30 (3.44, 5.37)
Bjorkenstam, 2012 (F)	F	RRatio	Scandinavia	Community and inpatient	42 / 5380	3279 / 1513294	4.10 (3.00, 5.60)
Crump, 2013a (M)	M	HR	Scandinavia	Community and inpatient	113 / 2765	47405 / 1446217	2.96 (2.46, 3.56)
Crump, 2013a (F)	F	HR	Scandinavia	Community and inpatient	100 / 1474	40764 / 1604761	3.73 (3.07, 4.54)
Kilbourne, 2009	P	HR	N. America	Community	1579 / 22817	1380 / 16072	1.37 (1.26, 1.49)
Laursen, 2010 (2001-2002)	P	RRatio	Scandinavia	Inpatient	150 / NR	23464 / NR	2.52 (2.14, 2.97)
Laursen, 2010 (2005-2006)	P	RRatio	Scandinavia	Inpatient	178 / NR	18209 / NR	3.52 (3.00, 4.13)
Laursen, 2010 (2003-2004)	P	RRatio	Scandinavia	Inpatient	164 / NR	20779 / NR	3.16 (2.70, 3.69)
Laursen, 2014	P	RRatio	Scandinavia	Community and inpatient	26 / 3000 (est)	2229 / 1061532	2.67 (1.81, 3.93)
Lumme, 2016 (2005) (M)	M	RRatio	Scandinavia	Inpatient	61 / NR	2499 / NR	3.79 (2.94, 4.88)
Lumme, 2016 (2006) (M)	M	RRatio	Scandinavia	Inpatient	54 / NR	2499 / NR	3.25 (2.48, 4.25)
Lumme, 2016 (2002) (M)	M	RRatio	Scandinavia	Inpatient	58 / NR	2763 / NR	3.53 (2.72, 4.58)
Lumme, 2016 (2007) (M)	M	RRatio	Scandinavia	Inpatient	63 / NR	2499 / NR	3.95 (3.08, 5.08)
Lumme, 2016 (2008) (M)	M	RRatio	Scandinavia	Inpatient	72 / NR	2377 / NR	4.50 (3.56, 5.69)
Lumme, 2016 (2004) (M)	M	RRatio	Scandinavia	Inpatient	48 / NR	2763 / NR	2.90 (2.18, 3.85)
Lumme, 2016 (2003) (M)	M	RRatio	Scandinavia	Inpatient	61 / NR	2763 / NR	3.77 (2.93, 4.86)
Lumme, 2016 (2001) (M)	M	RRatio	Scandinavia	Inpatient	53 / NR	3092 / NR	3.30 (2.51, 4.32)
Lumme, 2016 (2009) (M)	M	RRatio	Scandinavia	Inpatient	63 / NR	2377 / NR	4.12 (3.21, 5.29)
Lumme, 2016 (2004) (F)	F	RRatio	Scandinavia	Inpatient	48 / NR	752 / NR	8.36 (6.24, 11.19)
Lumme, 2016 (2006) (F)	F	RRatio	Scandinavia	Inpatient	29 / NR	622 / NR	6.38 (4.40, 9.26)
Lumme, 2016 (2009) (F)	F	RRatio	Scandinavia	Inpatient	28 / NR	570 / NR	6.44 (4.41, 9.41)
Lumme, 2016 (2002) (F)	F	RRatio	Scandinavia	Inpatient	45 / NR	752 / NR	7.97 (5.90, 10.77)
Lumme, 2016 (2003) (F)	F	RRatio	Scandinavia	Inpatient	32 / NR	752 / NR	5.59 (3.93, 7.97)
Lumme, 2016 (2005) (F)	F	RRatio	Scandinavia	Inpatient	34 / NR	622 / NR	7.42 (5.25, 10.48)
Lumme, 2016 (2007) (F)	F	RRatio	Scandinavia	Inpatient	22 / NR	622 / NR	4.50 (2.94, 6.88)
Lumme, 2016 (2008) (F)	F	RRatio	Scandinavia	Inpatient	32 / NR	570 / NR	6.96 (4.88, 9.94)
Lumme, 2016 (2001) (F)	F	RRatio	Scandinavia	Inpatient	27 / NR	921 / NR	4.50 (3.07, 6.59)
Lumme, 2016 (2000) (M)	M	RRatio	Scandinavia	Inpatient	63 / NR	3092 / NR	3.68 (2.87, 4.72)
Lumme, 2016 (2000) (F)	F	RRatio	Scandinavia	Inpatient	26 / NR	921 / NR	4.15 (2.81, 6.13)
Manderbacka, 2012	P	RRatio	Scandinavia	Inpatient	NR / 67659	NR / Gen pop	1.35 (1.30, 1.41)
Murray-Thomas, 2013	P	RRatio	UK	Community	8 / 7779	66 / NR	2.04 (0.98, 4.25)
Osby, 2016 (2003-2006)	P	RRatio	Scandinavia	Inpatient	NR / 11477	NR / Gen pop	3.14 (Insufficient data)
Osby, 2016 (1999-2002)	P	RRatio	Scandinavia	Inpatient	NR / 11880	NR / Gen pop	3.05 (Insufficient data)
Osby, 2016 (2007-2010)	P	RRatio	Scandinavia	Inpatient	NR / 10940	NR / Gen pop	3.07 (Insufficient data)
**2010s**							
Lumme, 2016 (2010) (M)	M	RRatio	Scandinavia	Inpatient	63 / NR	2377 / NR	4.32 (3.36, 5.54)
Lumme, 2016 (2010) (F)	F	RRatio	Scandinavia	Inpatient	24 / NR	570 / NR	5.65 (3.76, 8.51)

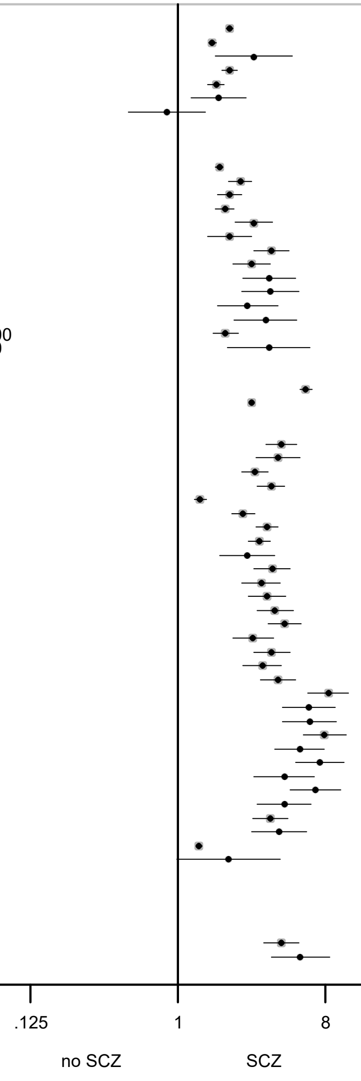

SCZ – schizophrenia, NR – not reported, gen pop – general population, HR – hazard ratio, RRatio – rate ratio, P – persons, M – males, F - females

Fig H: Schizophrenia, mortality from coronary heart disease, hazard ratios, rate ratios and odds ratios

Study identifier	Gender	Type of effect	Region	Setting	Events/Total Exposed	Events/Total Unexposed	Effect size (95% CI)
**1950s - 70s**							
Odegard, 1967 (M)	M	RRatio	Scandinavia	Inpatient	NR / NR	NR / NR	1.27 (Insufficient data)
Odegard, 1967 (F)	F	RRatio	Scandinavia	Inpatient	NR / NR	NR / NR	1.59 (Insufficient data)
**1980s**							
Osby, 2016 (1987-1990)	P	RRatio	Scandinavia	Inpatient	NR / 15744	NR / Gen pop	2.83 (Insufficient data)
Salazar-Fraile, 1998	P	HR	Other Europe/Israel	Community	17 / 186	54 / 2382	1.20 (0.47, 3.04)
**1990s**							
Brink, 2018 (Ages 50-59)	P	RRatio	Scandinavia	Inpatient	NR / 3700	NR / 19900	3.21 (2.67, 3.86)
Brink, 2018 (Ages 30-39)	P	RRatio	Scandinavia	Inpatient	NR / 2500	NR / 12300	3.80 (1.75, 8.27)
Brink, 2018 (Ages 60-69)	P	RRatio	Scandinavia	Inpatient	NR / 1800	NR / 11000	3.02 (2.51, 3.64)
Brink, 2018 (Ages 70-79)	P	RRatio	Scandinavia	Inpatient	NR / 400	NR / 2300	3.23 (2.40, 4.34)
Brink, 2018 (Ages 40-49)	P	RRatio	Scandinavia	Inpatient	NR / 3900	NR / 20200	2.51 (1.86, 3.38)
Curkendall, 2004	P	RRatio	N. America	Community	NR / 2405	NR / 9175	2.00 (1.60, 2.50)
Fors, 2007	P	RRatio	Scandinavia	Community	NR / 255	NR / 1275	2.20 (1.38, 3.51)
Osby, 2016 (1991-1994)	P	RRatio	Scandinavia	Inpatient	NR / 15096	NR / Gen pop	2.64 (Insufficient data)
Osby, 2016 (1995-1998)	P	RRatio	Scandinavia	Inpatient	NR / 13774	NR / Gen pop	2.47 (Insufficient data)
Westman, 2017 (All ages)	P	RRatio	Scandinavia	Inpatient	5245 / 46911	NR / 10631817	2.80 (2.73, 2.88)
Westman, 2017 (Ages 15-59)	P	RRatio	Scandinavia	Inpatient	1073 / NR	NR / NR	6.16 (5.80, 6.55)
**2000s**							
Crump, 2013a (M)	M	HR	Scandinavia	Community and inpatient	216 / 2765	91678 / 1446217	3.03 (2.65, 3.46)
Crump, 2013a (F)	F	HR	Scandinavia	Community and inpatient	204 / 1474	101179 / 1604761	3.11 (2.71, 3.57)
Hayes, 2017	P	HR	UK	Community	106 / 22497	437 / 219387	1.43 (1.16, 1.77)
Kredentser, 2014	P	RRatio	N. America	Community and inpatient	593 / 9038	30321 / 969090	1.64 (1.50, 1.80)
Laursen, 2019 (M)	M	RRatio	Scandinavia	Community and inpatient	1409 / 15668	183383 / 1641926	2.85 (2.69, 3.02)
Laursen, 2019 (F)	F	RRatio	Scandinavia	Community and inpatient	1345 / 8630	195846 / 1655114	2.13 (2.00, 2.26)
Lesage, 2015 (M)	M	RRatio	N. America	Community	14643 / NR	76178 / NR	1.70 (1.49, 1.94)
Lesage, 2015 (F)	F	RRatio	N. America	Community	18929 / NR	78208 / NR	1.64 (1.45, 1.84)
Morden, 2012	P	RRatio	N. America	Community and inpatient	NR / 65362	NR / 65362	1.18 (1.12, 1.24)
Osby, 2016 (1999-2002)	P	RRatio	Scandinavia	Inpatient	NR / 11880	NR / Gen pop	3.01 (Insufficient data)
Osby, 2016 (2007-2010)	P	RRatio	Scandinavia	Inpatient	NR / 10940	NR / Gen pop	3.62 (Insufficient data)
Osby, 2016 (2003-2006)	P	RRatio	Scandinavia	Inpatient	NR / 11477	NR / Gen pop	3.14 (Insufficient data)
Sanchez, 2021	P	HR	Other Europe/Israel	Community	NR / 6472	NR / 94694	1.66 (1.21, 2.27)
Termorshuizen, 2013 (Age <40, 2-5y FU)	P	RRatio	Other Europe/Israel	Community and inpatient	NR / NR	NR / NR	3.09 (1.09, 8.75)
Termorshuizen, 2013 (Age 40-60, >5y FU)	P	RRatio	Other Europe/Israel	Community and inpatient	NR / NR	NR / NR	3.88 (2.17, 6.92)
Termorshuizen, 2013 (Age 40-60, 2-5y FU)	P	RRatio	Other Europe/Israel	Community and inpatient	NR / NR	NR / NR	4.56 (2.98, 6.98)
Termorshuizen, 2013 (Age 40-60, <2y FU)	P	RRatio	Other Europe/Israel	Community and inpatient	NR / NR	NR / NR	4.31 (2.56, 7.24)
Termorshuizen, 2013 (Age >60, <2y FU)	P	RRatio	Other Europe/Israel	Community and inpatient	NR / NR	NR / NR	4.37 (2.47, 7.73)
Termorshuizen, 2013 (Age >60, 2-5y FU)	P	RRatio	Other Europe/Israel	Community and inpatient	NR / NR	NR / NR	2.59 (1.50, 4.48)
Termorshuizen, 2013 (Age >60, >5y FU)	P	RRatio	Other Europe/Israel	Community and inpatient	NR / NR	NR / NR	3.84 (2.14, 6.89)
Termorshuizen, 2013 (Age <40, >5y FU)	P	RRatio	Other Europe/Israel	Community and inpatient	NR / NR	NR / NR	4.98 (1.18, 20.96)
Termorshuizen, 2013 (Age <40, <2y FU)	P	RRatio	Other Europe/Israel	Community and inpatient	NR / NR	NR / NR	4.93 (1.42, 17.06)
Torniainen, 2015 (Mod antipsychotic)	P	HR	Scandinavia	Community and inpatient	178 / 8468	NR / 84680 (est)	5.63 (4.79, 6.62)
Torniainen, 2015 (Low antipsychotic)	P	HR	Scandinavia	Community and inpatient	72 / 4110	NR / 41100 (est)	4.68 (3.68, 5.96)
Torniainen, 2015 (No antipsychotics)	P	HR	Scandinavia	Community and inpatient	57 / 2077	NR / 20770 (est)	7.59 (5.80, 9.93)
Torniainen, 2015 (High antipsychotic)	P	HR	Scandinavia	Community and inpatient	213 / 6837	NR / 68370 (est)	8.45 (7.27, 9.83)
**2010s**							
Vance, 2019 (M)	M	OR	N. America	Community	2293 / 64437	16284 / 810153	2.13 (2.04, 2.23)
Vance, 2019 (F)	F	OR	N. America	Community	100 / 487	229 / 1407	3.15 (2.54, 3.91)

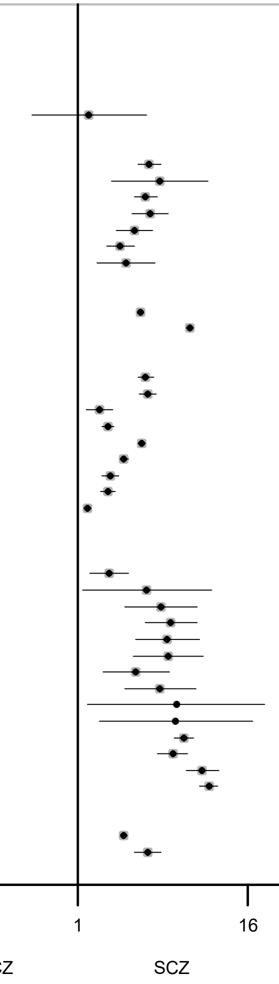

SCZ – schizophrenia, NR – not reported, gen pop – general population, est – estimated, HR – hazard ratio, RRatio – rate ratio, OR – odds ratio, P – persons, M – males, F – females, FU – follow up

Fig I: Schizophrenia, mortality from all circulatory disease, hazard ratios, rate ratios and odds ratios

Study identifier	Gender	Type of effect	Region	Setting	Events/Total Exposed	Events/Total Unexposed	Effect size (95% CI)
**1990s**							
Westman, 2017 (Ages 15-59)	P	RRatio	Scandinavia	Inpatient	NR / NR	NR / NR	13.56 (9.18, 20.02)
Westman, 2017 (All ages)	P	RRatio	Scandinavia	Inpatient	376 / 46911	NR / 10631817	3.25 (2.94, 3.60)

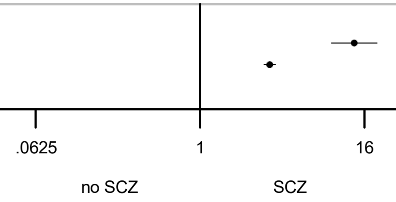

SCZ – schizophrenia, NR – not reported, RRatio – rate ratio, P – persons

Fig J: Schizophrenia, mortality from heart failure, hazard ratios, rate ratios and odds ratios

Study identifier	Gender	Type of effect	Region	Setting	Events/Total Exposed	Events/Total Unexposed	Effect size (95% CI)
**1980s**							
Osby, 2016 (1987-1990)	P	RRatio	Scandinavia	Inpatient	NR / 6564	NR / Gen pop	1.52 (Insufficient data)
**1990s**							
Osborn, 2007 (Ages 50-75)	P	HR	UK	Community	23 / Approx 4000	275 / Approx 100000	1.63 (1.07, 2.49)
Osborn, 2007 (Ages 18-49)	P	HR	UK	Community	2 / Approx 6000	16 / Approx 200000	3.34 (0.76, 14.67)
Osby, 2016 (1991-1994)	P	RRatio	Scandinavia	Inpatient	NR / 6307	NR / Gen pop	2.12 (Insufficient data)
Osby, 2016 (1995-1998)	P	RRatio	Scandinavia	Inpatient	NR / 6257	NR / Gen pop	1.38 (Insufficient data)
Westman, 2013	P	RRatio	Scandinavia	Inpatient	368 / 17101	NR / 10631208	2.00 (1.81, 2.21)
**2000s**							
Crump, 2013b (M)	M	HR	Scandinavia	Community and inpatient	18 / 1102	14560 / 1575099	1.77 (1.12, 2.80)
Crump, 2013b (F)	F	HR	Scandinavia	Community and inpatient	48 / 2319	20924 / 1720013	2.63 (1.98, 3.49)
Osby, 2016 (1999-2002)	P	RRatio	Scandinavia	Inpatient	NR / 6245	NR / Gen pop	1.64 (Insufficient data)
Osby, 2016 (2007-2010)	P	RRatio	Scandinavia	Inpatient	NR / 9995	NR / Gen pop	2.65 (Insufficient data)
Osby, 2016 (2003-2006)	P	RRatio	Scandinavia	Inpatient	NR / 7596	NR / Gen pop	1.86 (Insufficient data)

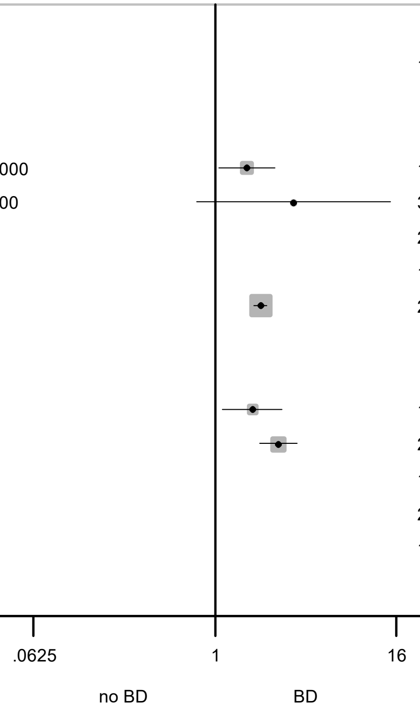

BD – bipolar disorder, NR – not reported, gen pop – general population, HR – hazard ratio, RRatio – rate ratio, P – persons, M – males, F - females

Fig K: Bipolar disorder, mortality from cerebrovascular accident, hazard ratios, rate ratios and odds ratios

Study identifier	Gender	Type of effect	Region	Setting	Events/Total Exposed	Events/Total Unexposed	Effect size (95% CI)
**1980s**							
Laursen, 2007 (M)	M	RRatio	Scandinavia	Inpatient	NR / NR	NR / NR	1.58 (1.43, 1.75)
Laursen, 2007 (F)	F	RRatio	Scandinavia	Inpatient	NR / NR	NR / NR	1.67 (1.52, 1.83)
Osby, 2016 (1987-1990)	P	RRatio	Scandinavia	Inpatient	NR / 6564	NR / Gen pop	1.72 (Insufficient data)
**1990s**							
Daumit, 2010	P	RRatio	N. America	Community	NR / 1058 (est)	NR / Gen pop	3.10 (3.00, 3.20)
Laursen, 2010 (1999-2000)	P	RRatio	Scandinavia	Inpatient	123 / NR	24614 / NR	1.64 (1.37, 1.96)
Laursen, 2010 (1994-1996)	P	RRatio	Scandinavia	Inpatient	209 / NR	43350 / NR	1.89 (1.64, 2.18)
Laursen, 2010 (1997-1998)	P	RRatio	Scandinavia	Inpatient	141 / NR	25488 / NR	1.93 (1.63, 2.28)
Lumme, 2016 (1999) (M)	M	RRatio	Scandinavia	Inpatient	9 / NR	3092 / NR	1.08 (0.56, 2.08)
Lumme, 2016 (1997) (M)	M	RRatio	Scandinavia	Inpatient	6 / NR	3434 / NR	0.57 (0.25, 1.26)
Lumme, 2016 (1996) (M)	M	RRatio	Scandinavia	Inpatient	5 / NR	3434 / NR	1.00 (0.42, 2.40)
Lumme, 2016 (1998) (M)	M	RRatio	Scandinavia	Inpatient	7 / NR	3434 / NR	1.55 (0.74, 3.26)
Lumme, 2016 (1999) (F)	F	RRatio	Scandinavia	Inpatient	6 / NR	921 / NR	3.21 (1.44, 7.16)
Lumme, 2016 (1997) (F)	F	RRatio	Scandinavia	Inpatient	3 / NR	1138 / NR	2.12 (0.68, 6.58)
Lumme, 2016 (1996) (F)	F	RRatio	Scandinavia	Inpatient	4 / NR	1138 / NR	3.21 (1.20, 8.56)
Lumme, 2016 (1998) (F)	F	RRatio	Scandinavia	Inpatient	4 / NR	1138 / NR	3.17 (1.19, 8.47)
Osborn, 2007 (Ages 50-75)	P	HR	UK	Community	66 / Approx 4000	896 / Approx 100000	1.52 (1.18, 1.95)
Osborn, 2007 (Ages 18-49)	P	HR	UK	Community	4 / Approx 6000	49 / Approx 200000	2.13 (0.77, 5.91)
Osby, 2016 (1995-1998)	P	RRatio	Scandinavia	Inpatient	NR / 6257	NR / Gen pop	1.92 (Insufficient data)
Osby, 2016 (1991-1994)	P	RRatio	Scandinavia	Inpatient	NR / 6307	NR / Gen pop	1.93 (Insufficient data)
Westman, 2013	P	RRatio	Scandinavia	Inpatient	776 / 17101	NR / 10631208	1.95 (1.81, 2.10)
**2000s**							
Crump, 2013b (M)	M	HR	Scandinavia	Community and inpatient	92 / 1102	56062 / 1575099	2.29 (1.87, 2.81)
Crump, 2013b (F)	F	HR	Scandinavia	Community and inpatient	89 / 2319	48312 / 1720013	2.16 (1.76, 2.66)
Kilbourne, 2009	P	HR	N. America	Community	921 / 15203	1380 / 16072	1.24 (1.13, 1.36)
Laursen, 2010 (2001-2002)	P	RRatio	Scandinavia	Inpatient	145 / NR	23464 / NR	1.93 (1.63, 2.28)
Laursen, 2010 (2005-2006)	P	RRatio	Scandinavia	Inpatient	125 / NR	18209 / NR	2.09 (1.76, 2.48)
Laursen, 2010 (2003-2004)	P	RRatio	Scandinavia	Inpatient	133 / NR	20779 / NR	1.99 (1.67, 2.37)
Laursen, 2014	P	RRatio	Scandinavia	Community and inpatient	22 / 1500 (est)	2229 / 1061532	2.96 (1.95, 4.50)
Lumme, 2016 (2001) (M)	M	RRatio	Scandinavia	Inpatient	14 / NR	3092 / NR	2.47 (1.46, 4.17)
Lumme, 2016 (2005) (M)	M	RRatio	Scandinavia	Inpatient	20 / NR	2499 / NR	1.92 (1.24, 2.98)
Lumme, 2016 (2006) (M)	M	RRatio	Scandinavia	Inpatient	13 / NR	2499 / NR	0.97 (0.56, 1.68)
Lumme, 2016 (2003) (M)	M	RRatio	Scandinavia	Inpatient	13 / NR	2763 / NR	2.18 (1.27, 3.76)
Lumme, 2016 (2008) (M)	M	RRatio	Scandinavia	Inpatient	21 / NR	2377 / NR	2.08 (1.35, 3.19)
Lumme, 2016 (2004) (M)	M	RRatio	Scandinavia	Inpatient	13 / NR	2763 / NR	1.38 (0.80, 2.38)
Lumme, 2016 (2007) (M)	M	RRatio	Scandinavia	Inpatient	23 / NR	2499 / NR	2.87 (1.90, 4.32)
Lumme, 2016 (2002) (M)	M	RRatio	Scandinavia	Inpatient	8 / NR	2763 / NR	1.24 (0.62, 2.49)
Lumme, 2016 (2009) (M)	M	RRatio	Scandinavia	Inpatient	25 / NR	2377 / NR	2.66 (1.79, 3.95)
Lumme, 2016 (2002) (F)	F	RRatio	Scandinavia	Inpatient	7 / NR	752 / NR	4.62 (2.20, 9.73)
Lumme, 2016 (2006) (F)	F	RRatio	Scandinavia	Inpatient	6 / NR	622 / NR	2.05 (0.92, 4.58)
Lumme, 2016 (2001) (F)	F	RRatio	Scandinavia	Inpatient	5 / NR	921 / NR	3.24 (1.34, 7.79)
Lumme, 2016 (2005) (F)	F	RRatio	Scandinavia	Inpatient	10 / NR	622 / NR	3.14 (1.68, 5.87)
Lumme, 2016 (2008) (F)	F	RRatio	Scandinavia	Inpatient	8 / NR	570 / NR	2.98 (1.48, 5.99)
Lumme, 2016 (2007) (F)	F	RRatio	Scandinavia	Inpatient	7 / NR	622 / NR	2.05 (0.97, 4.32)
Lumme, 2016 (2009) (F)	F	RRatio	Scandinavia	Inpatient	10 / NR	570 / NR	3.74 (2.00, 6.98)
Lumme, 2016 (2004) (F)	F	RRatio	Scandinavia	Inpatient	2 / NR	752 / NR	0.98 (0.24, 3.92)
Lumme, 2016 (2003) (F)	F	RRatio	Scandinavia	Inpatient	6 / NR	752 / NR	3.38 (1.51, 7.54)
Lumme, 2016 (2000) (M)	M	RRatio	Scandinavia	Inpatient	11 / NR	3092 / NR	1.06 (0.59, 1.91)
Lumme, 2016 (2000) (F)	F	RRatio	Scandinavia	Inpatient	4 / NR	921 / NR	3.86 (1.45, 10.32)
Osby, 2016 (2003-2006)	P	RRatio	Scandinavia	Inpatient	NR / 7596	NR / Gen pop	1.78 (Insufficient data)
Osby, 2016 (2007-2010)	P	RRatio	Scandinavia	Inpatient	NR / 9995	NR / Gen pop	2.02 (Insufficient data)
Osby, 2016 (1999-2002)	P	RRatio	Scandinavia	Inpatient	NR / 6245	NR / Gen pop	2.62 (Insufficient data)
**2010s**							
Lumme, 2016 (2010) (M)	M	RRatio	Scandinavia	Inpatient	23 / NR	2377 / NR	2.08 (1.38, 3.13)
Lumme, 2016 (2010) (F)	F	RRatio	Scandinavia	Inpatient	5 / NR	570 / NR	1.21 (0.50, 2.92)

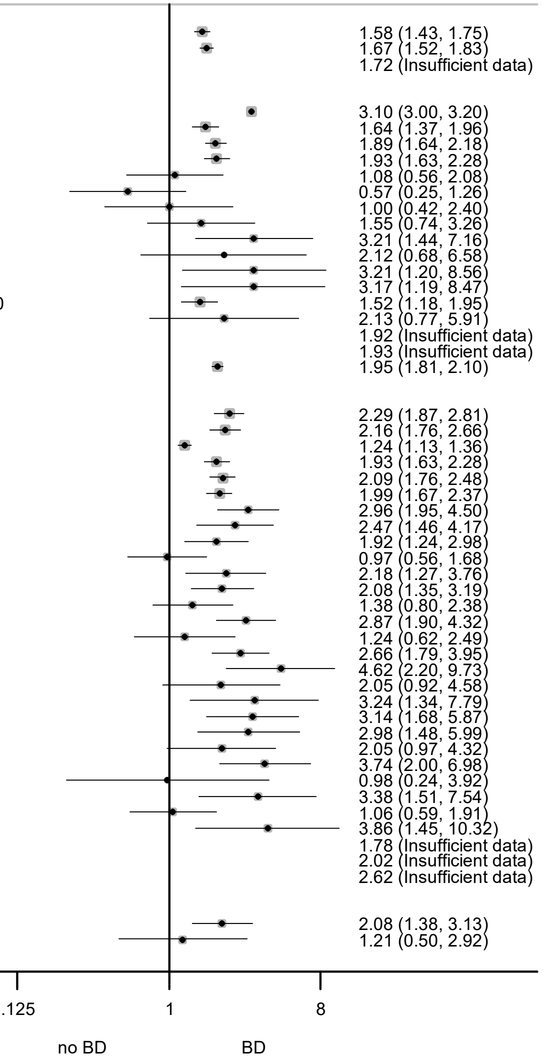

BD – bipolar disorder, NR – not reported, gen pop – general population, est – estimated, HR – hazard ratio, RRatio – rate ratio, P – persons, M – males, F - females

Fig L: Bipolar disorder, mortality from coronary heart disease, hazard ratios, rate ratios and odds ratios

Study identifier	Gender	Type of effect	Region	Setting	Events/Total Exposed	Events/Total Unexposed	Effect size (95% CI)
**1980s**							
Osby, 2016 (1987-1990)	P	RRatio	Scandinavia	Inpatient	NR / 6564	NR / Gen pop	1.72 (Insufficient data)
**1990s**							
Osby, 2016 (1995-1998)	P	RRatio	Scandinavia	Inpatient	NR / 6257	NR / Gen pop	1.87 (Insufficient data)
Osby, 2016 (1991-1994)	P	RRatio	Scandinavia	Inpatient	NR / 6307	NR / Gen pop	2.08 (Insufficient data)
Westman, 2013	P	RRatio	Scandinavia	Inpatient	1625 / 17101	NR / 10631208	2.03 (1.93, 2.13)
**2000s**							
Crump, 2013b (M)	M	HR	Scandinavia	Community and inpatient	162 / 1102	109079 / 1575099	2.16 (1.85, 2.52)
Crump, 2013b (F)	F	HR	Scandinavia	Community and inpatient	219 / 2319	120703 / 1720013	2.19 (1.92, 2.50)
Hayes, 2017	P	HR	UK	Community	59 / 17341	437 / 219387	1.11 (0.85, 1.45)
Osby, 2016 (2003-2006)	P	RRatio	Scandinavia	Inpatient	NR / 7596	NR / Gen pop	1.96 (Insufficient data)
Osby, 2016 (2007-2010)	P	RRatio	Scandinavia	Inpatient	NR / 9995	NR / Gen pop	2.18 (Insufficient data)
Osby, 2016 (1999-2002)	P	RRatio	Scandinavia	Inpatient	NR / 6245	NR / Gen pop	2.35 (Insufficient data)
**2010s**							
Vance, 2019 (M)	M	OR	N. America	Community	NR / 71471	16284 / 810153	1.73 (1.65, 1.81)
Vance, 2019 (F)	F	OR	N. America	Community	NR / 1406	229 / 1407	2.04 (1.70, 2.45)

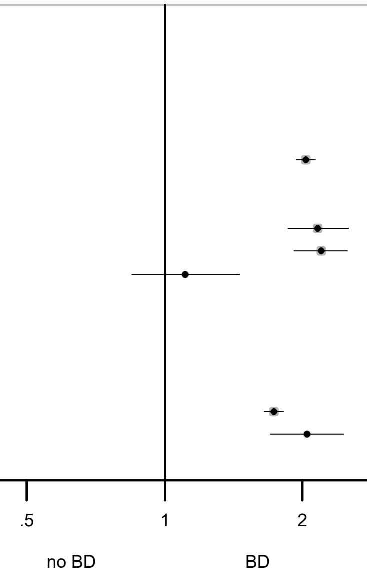

BD – bipolar disorder, NR – not reported, gen pop – general population, HR – hazard ratio, RRatio – rate ratio, OR – odds ratio, P – persons, M – males, F - females

Fig M: Bipolar disorder, mortality from all circulatory disease, hazard ratios, rate ratios and odds ratios

Study identifier	Gender	Type of effect	Region	Setting	Events/Total Exposed	Events/Total Unexposed	Effect size (95% CI)
**Cerebrovascular disease, 1950s - 70s**							
Dalgard, 1966	P	RRatio	Scandinavia	Inpatient	NR / 1126	NR / 1126	1.30 (Insufficient data)
**Cerebrovascular disease, 2000s**							
John, 2018 (Ages <45)	P	SMR	UK	Community and inpatient	NR / 12961	NR / NR	0.80 (0.16, 4.08)
John, 2018 (Ages 45-64)	P	SMR	UK	Community and inpatient	NR / 9398	NR / NR	3.60 (2.73, 4.75)
**Coronary heart disease, 1950s - 70s**							
Dalgard, 1966	P	RRatio	Scandinavia	Inpatient	NR / 1126	NR / 1126	1.70 (Insufficient data)
**Coronary heart disease, 2000s**							
John, 2018 (Ages 45-64)	P	SMR	UK	Community and inpatient	NR / 9398	NR / NR	2.90 (2.45, 3.43)
John, 2018 (Ages <45)	P	SMR	UK	Community and inpatient	NR / 12961	NR / NR	3.70 (2.47, 5.55)
**All circulatory disease, 1980s**							
Dutta, 2012 (1965-2007)	P	SMR	UK	Community	142 / 2723	102 / Gen pop	1.39 (1.17, 1.65)
Hansen, 2001 (M)	M	SMR	Scandinavia	Inpatient	NR / NR	NR / NR	2.10 (1.48, 2.97)
Hansen, 2001 (F)	F	SMR	Scandinavia	Inpatient	NR / NR	NR / NR	2.30 (1.53, 3.46)
**All circulatory disease, 2000s**							
John, 2018 (Ages 45-64)	P	SMR	UK	Community and inpatient	NR / 9398	NR / NR	3.40 (3.02, 3.83)
John, 2018 (Ages <45)	P	SMR	UK	Community and inpatient	NR / 12961	NR / NR	3.70 (2.80, 4.89)
Lemogne, 2013	P	HR	Other Europe/Israel	Community	6 / 124	199 / 16261	5.41 (2.40, 12.20)
**All circulatory disease, 2010s**							
Das-Munshi, 2017	P	SMR	UK	Community	647 / 18201	244 / Gen pop	2.65 (2.45, 2.86)

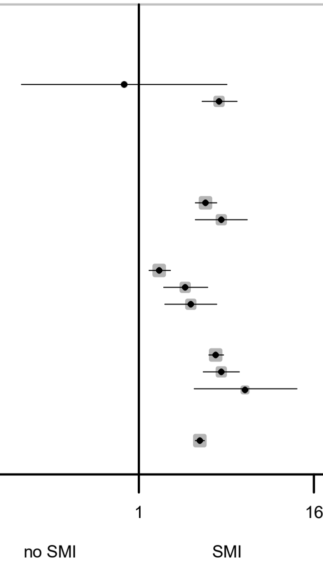

SMI – severe mental illness, NR – not reported, gen pop – general population, HR – hazard ratio, RRatio – rate ratio, SMR – standardised mortality ratio, P – persons, M – males, F - females

Fig N: Mixed SMI, mortality from cardiovascular diseases, hazard ratios, rate ratios, odds ratios, standardised mortality ratios
