# Supplementary material for: Temporal trends in associations between severe mental illness and risk of cardiovascular disease: A systematic review and meta-analysis
Source: PLoS Med. 2022 Apr 19;19(4):e1003960. doi: 10.1371/journal.pmed.1003960 (PMC9017899; doi:10.1371/journal.pmed.1003960)
Supplement: S15 File — Fig A: Schizophrenia, incidence of CVA, HRs, rate ratios and ORs. Fig B: Schizophrenia, incidence of CHD, HRs, rate ratios and ORs. Fig C: Schizophrenia, incidence of major cardiovascular events, HRs, rate ratios and ORs. Fig D: Schizophrenia, incidence of heart failure, HRs, rate ratios and odds ratios. Fig E: BD, incidence of CVA, HRs, rate ratios, and ORs. Fig F: BD, incidence of CHD, HRs, rate ratios and ORs. Fig G: BD, incidence of major cardiovascular events, HRs, rate ratios and ORs. Fig H: BD, incidence of heart failure, HRs, rate ratios and ORs. BD, bipolar disorder; CHD, coronary heart disease; CVA, cerebrovascular accident; HR, hazard ratio; OR, odds ratio. (PDF) [file pmed.1003960.s015.pdf]

S15 File. Forest plots reporting cardiovascular incidence outcomes, across decades

Study identifier	Gender	Type of effect	Region	Setting	Events/Total Exposed	Events/Total Unexposed	Effect size (95% CI)
**1980s**							
Lahti, 2012	P	HR	Scandinavia	Inpatient	10 / 204	464 / 12735	1.41 (0.75, 2.65)
Munk-Jorgensen, 2000	P	RRatio	Scandinavia	Inpatient	258 / 20495	NR / 204912	0.88 (0.78, 1.00)
**1990s**							
Carney, 2006	P	OR	N. America	Community and inpatient	14 / 1074	3187 / 726262	2.11 (1.22, 3.65)
Curkendall, 2004	P	RR	N. America	Community	NR / 2405	NR / 9175	1.30 (0.97, 1.74)
McDermott, 2005	P	RR	N. America	Community	NR / 357	NR / 2083	1.53 (0.74, 3.17)
Westman, 2017 (All ages)	P	RRatio	Scandinavia	Inpatient	926 / 46911	NR / 10631817	1.25 (1.17, 1.33)
**2000s**							
Chen, 2015	P	RR	Far East	Community and inpatient	1088 / 63913	552 / 63913	1.97 (1.78, 2.18)
Crump, 2013a (M)	M	HR	Scandinavia	Community and inpatient	138 / 2765	111779 / 1446217	1.28 (1.08, 1.51)
Crump, 2013a (F)	F	HR	Scandinavia	Community and inpatient	113 / 1474	108457 / 1604761	1.33 (1.11, 1.60)
Jackson, 2020 (M)	M	RRatio	UK	Inpatient	NR / NR	NR / NR	1.87 (1.48, 2.37)
Jackson, 2020 (F)	F	RRatio	UK	Inpatient	NR / NR	NR / NR	2.26 (1.79, 2.86)
Laursen, 2011	P	RRatio	Scandinavia	Inpatient	167 / 16079	14501 / 2428518	1.66 (1.42, 1.94)
Momen, 2020	P	HR	Scandinavia	Community and inpatient	NR / NR	NR / NR	1.66 (1.62, 1.71)
Tsai, 2012	P	RR	Far East	Community and inpatient	1380 / 80569	2954 / 241707	1.40 (1.32, 1.49)
Yu-Chuan Chiu, 2015	P	OR	Far East	Community and inpatient	24 / 80	2039 / 10235	1.72 (1.07, 2.79)

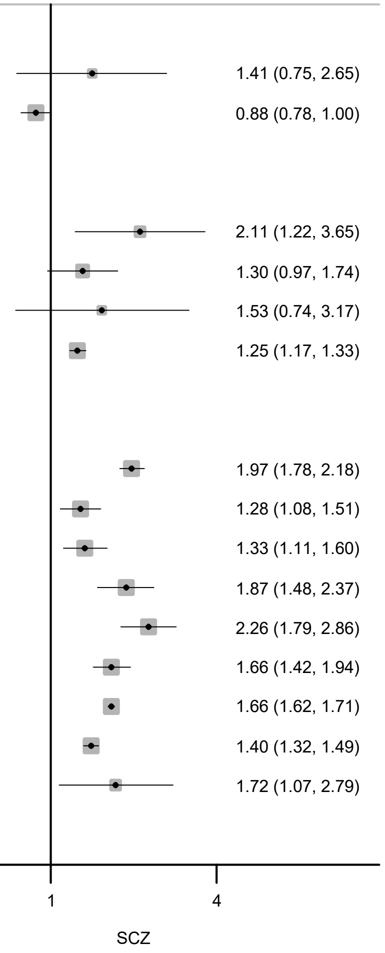

SCZ – schizophrenia, NR – not reported, HR – hazard ratio, RRatio – rate ratio, OR – odds ratio, RR – risk ratio, P – persons, M – males, F - females

Fig A: Schizophrenia, incidence of cerebrovascular accident, hazard ratios, rate ratios and odds ratios

Study identifier	Gender	Type of effect	Region	Setting	Events/Total Exposed	Events/Total Unexposed	Effect size (95% CI)
**1980s**							
Gale, 2013 (M)	M	HR	Scandinavia	Inpatient	NR / 5961	NR / 1099304	1.49 (1.24, 1.80)
Jakobsen, 2008	P	RRatio	Scandinavia	Inpatient	173 / 15710	829 / 69447	0.77 (0.65, 0.91)
Lahti, 2012	P	HR	Scandinavia	Inpatient	19 / 204	758 / 12735	1.63 (1.03, 2.57)
Lawrence, 2003 (M)	M	RRatio	Oceania	Community and inpatient	NR / NR	NR / NR	0.59 (0.49, 0.71)
Lawrence, 2003 (F)	F	RRatio	Oceania	Community and inpatient	NR / NR	NR / NR	0.60 (0.47, 0.76)
Munk-Jorgensen, 2000	P	RRatio	Scandinavia	Inpatient	473 / 20495	NR / 204912	0.80 (0.73, 0.87)
**1990s**							
Carney, 2006	P	OR	N. America	Community and inpatient	25 / 1074	13567 / 726262	0.86 (0.56, 1.32)
Curkendall, 2004	P	RR	N. America	Community	NR / 2405	NR / 9175	1.10 (0.92, 1.32)
Kugathasan, 2018	P	RR	Scandinavia	Community and inpatient	537 / 36962	2938 / 184810	0.91 (0.83, 1.00)
Laursen, 2010 (1999-2000)	P	RRatio	Scandinavia	Inpatient	NR / NR	NR / NR	0.96 (0.83, 1.11)
Laursen, 2010 (1997-1998)	P	RRatio	Scandinavia	Inpatient	NR / NR	NR / NR	0.90 (0.77, 1.05)
Laursen, 2010 (1994-1996)	P	RRatio	Scandinavia	Inpatient	NR / NR	NR / NR	0.83 (0.72, 0.95)
McDermott, 2005	P	RR	N. America	Community	29 / 357	183 / 2083	0.57 (0.36, 0.91)
Sundquist, 2006 (M)	M	SIR	Scandinavia	Inpatient	1092 / NR	277111 / NR	1.39 (1.31, 1.48)
Sundquist, 2006 (F)	F	SIR	Scandinavia	Inpatient	577 / NR	133460 / NR	1.59 (1.46, 1.73)
Westman, 2017 (All ages)	P	RRatio	Scandinavia	Inpatient	850 / 46911	NR / 10631817	0.88 (0.83, 0.94)
**2000s**							
Crump, 2013a (M)	M	HR	Scandinavia	Community and inpatient	267 / 2765	253118 / 1446217	1.02 (0.90, 1.15)
Crump, 2013a (F)	F	HR	Scandinavia	Community and inpatient	183 / 1474	188319 / 1604761	1.20 (1.04, 1.39)
Gur, 2017	P	OR	Other Europe/Israel	Community and inpatient	67 / 1389	267 / 4095	0.73 (0.55, 0.96)
Jackson, 2020 (M)	M	RRatio	UK	Inpatient	NR / NR	NR / NR	1.91 (1.61, 2.27)
Jackson, 2020 (F)	F	RRatio	UK	Inpatient	NR / NR	NR / NR	2.50 (2.10, 2.98)
Laursen, 2010 (2001-2002)	P	RRatio	Scandinavia	Inpatient	NR / NR	NR / NR	1.07 (0.93, 1.23)
Laursen, 2010 (2005-2006)	P	RRatio	Scandinavia	Inpatient	NR / NR	NR / NR	1.20 (1.06, 1.36)
Laursen, 2010 (2003-2004)	P	RRatio	Scandinavia	Inpatient	NR / NR	NR / NR	1.11 (0.97, 1.27)
Laursen, 2011	P	RRatio	Scandinavia	Inpatient	74 / 16079	6156 / 2428518	1.41 (1.12, 1.78)
Lin, 2010	P	HR	Far East	Community and inpatient	30 / 7353	100 / 22059	1.86 (1.61, 2.14)
Manderbacka, 2012	P	RRatio	Scandinavia	Inpatient	4991 / 67659	370452 / Gen pop	1.04 (1.01, 1.07)
Momen, 2020	P	HR	Scandinavia	Community and inpatient	NR / NR	NR / NR	1.31 (1.28, 1.35)
Morden, 2012	P	RRatio	N. America	Community and inpatient	14706 / 65362	17713 / 65362	0.83 (0.50, 1.38)
Wu, 2015	P	HR	Far East	Community and inpatient	591 / 58106	2527 / 207592	1.41 (1.27, 1.56)

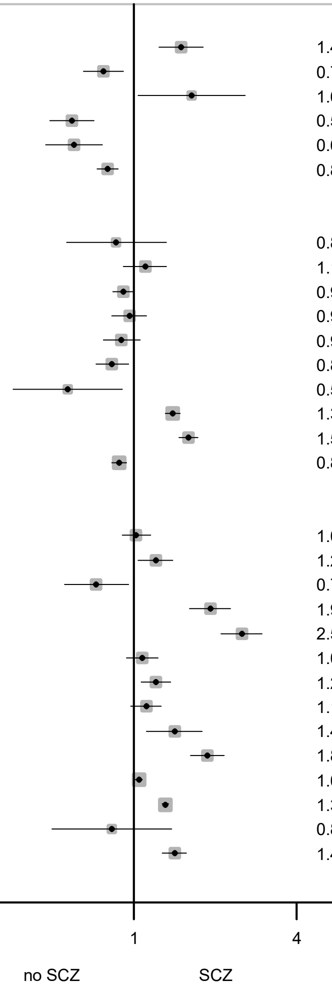

SCZ – schizophrenia, NR – not reported, gen pop – general population, HR – hazard ratio, RRatio – rate ratio, OR – odds ratio, RR – risk ratio, SIR – standardised incidence ratio, P – persons, M – males, F - females

Fig B: Schizophrenia, incidence of coronary heart disease, hazard ratios, rate ratios and odds ratios

Study identifier	Gender	Type of effect	Region	Setting	Events/Total Exposed	Events/Total Unexposed	Effect size (95% CI)
**1990s**							
Bent-Ennakhil, 2018	P	RRatio	Scandinavia	Community and inpatient	NR / 2450	NR / 200486	2.86 (Insufficient data)
Brink, 2018 (Ages 50-59)	P	RRatio	Scandinavia	Inpatient	300 / 3700	1537 / 19900	1.18 (1.05, 1.33)
Brink, 2018 (Ages 30-39)	P	RRatio	Scandinavia	Inpatient	30 / 2500	114 / 12300	1.25 (0.84, 1.87)
Brink, 2018 (Ages 60-69)	P	RRatio	Scandinavia	Inpatient	253 / 1800	1519 / 11000	1.22 (1.07, 1.39)
Brink, 2018 (Ages 70-79)	P	RRatio	Scandinavia	Inpatient	75 / 400	476 / 2300	1.29 (1.03, 1.61)
Brink, 2018 (Ages 40-49)	P	RRatio	Scandinavia	Inpatient	122 / 3900	634 / 20200	1.08 (0.90, 1.30)
**2000s**							
Hayes, 2017	P	HR	UK	Community	551 / 22497	2445 / 219387	1.47 (1.34, 1.61)
Sanchez, 2021	P	HR	Other Europe/Israel	Community	NR / 6472	NR / 94694	1.09 (0.89, 1.34)
**2010s**							
Vance, 2019 (M)	M	OR	N. America	Community	NR / 64437	NR / 810153	1.50 (1.45, 1.55)
Vance, 2019 (F)	F	OR	N. America	Community	NR / 487	NR / 1407	2.20 (1.88, 2.58)

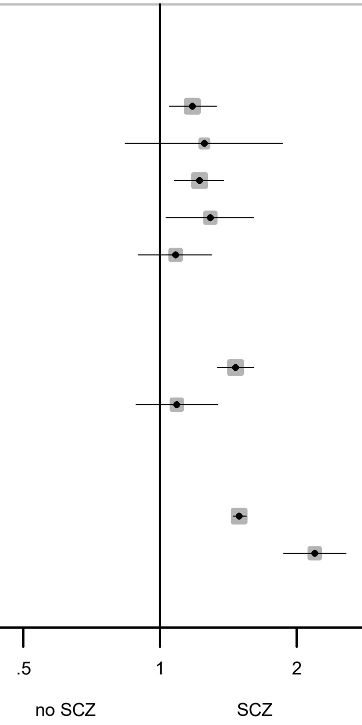

SCZ – schizophrenia, NR – not reported, HR – hazard ratio, RRatio – rate ratio, OR – odds ratio, P – persons, M – males, F - females

Fig C: Schizophrenia, incidence of major cardiovascular events, hazard ratios, rate ratios and odds ratios

Study identifier	Gender	Type of effect	Region	Setting	Events/Total Exposed	Events/Total Unexposed	Effect size (95% CI)
**1990s**							
Carney, 2006	P	OR	N. America	Community and inpatient	16 / 1074	3117 / 726262	2.38 (1.42, 4.00)
Curkendall, 2004	P	RR	N. America	Community	NR / 2405	NR / 9175	1.40 (1.13, 1.74)
McDermott, 2005	P	RR	N. America	Community	45 / 357	121 / 2083	2.27 (1.49, 3.45)
**2000s**							
Gur, 2017	P	OR	Other Europe/Israel	Community and inpatient	25 / 1389	54 / 4095	1.37 (0.85, 2.21)
Laursen, 2011	P	RRatio	Scandinavia	Inpatient	91 / 16079	3065 / 2428518	3.82 (3.10, 4.71)

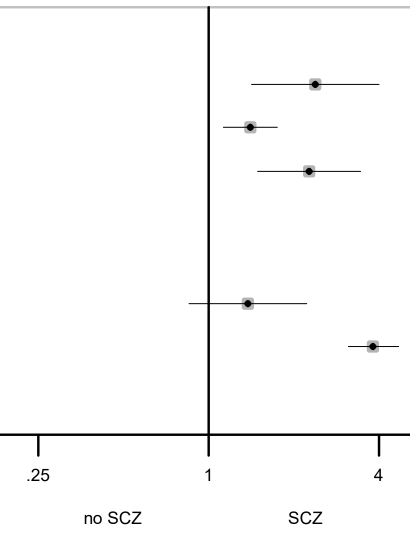

SCZ – schizophrenia, NR – not reported, RRatio – rate ratio, OR – odds ratio, RR – risk ratio, P – persons

Fig D: Schizophrenia, incidence of heart failure, hazard ratios, rate ratios and odds ratios

Study identifier	Gender	Type of effect	Region	Setting	Events/Total Exposed	Events/Total Unexposed	Effect size (95% CI)
**1980s**							
Prieto, 2016	P	HR	N. America	Community and inpatient	29 / 334	29 / 334	1.57 (0.93, 2.65)
**1990s**							
McDermott, 2005	P	RR	N. America	Community	NR / 146	NR / 2083	1.19 (0.34, 4.16)
Westman, 2013	P	RRatio	Scandinavia	Inpatient	450 / 17101	NR / 10631208	1.39 (1.27, 1.53)
**2000s**							
Crump, 2013b (M)	M	HR	Scandinavia	Community and inpatient	129 / 1102	112001 / 1575099	1.39 (1.17, 1.65)
Crump, 2013b (F)	F	HR	Scandinavia	Community and inpatient	197 / 2319	108675 / 1720013	1.70 (1.48, 1.96)
Jackson, 2020 (M)	M	RRatio	UK	Inpatient	NR / NR	NR / NR	1.80 (1.48, 2.18)
Jackson, 2020 (F)	F	RRatio	UK	Inpatient	NR / NR	NR / NR	1.97 (1.63, 2.39)
Laursen, 2011	P	RRatio	Scandinavia	Inpatient	77 / 6215	14501 / 2428518	2.03 (1.63, 2.53)
Wium-Andersen, 2021	P	HR	Scandinavia	Community and inpatient	748 / 13725	8509 / 167562	1.50 (1.38, 1.63)
Wu, 2013	P	RR	Far East	Community and inpatient	472 / 16821	1443 / 67284	1.31 (1.18, 1.45)
Yu-Chuan Chiu, 2015	P	OR	Far East	Community and inpatient	16 / 35	2047 / 10280	3.39 (1.91, 6.01)

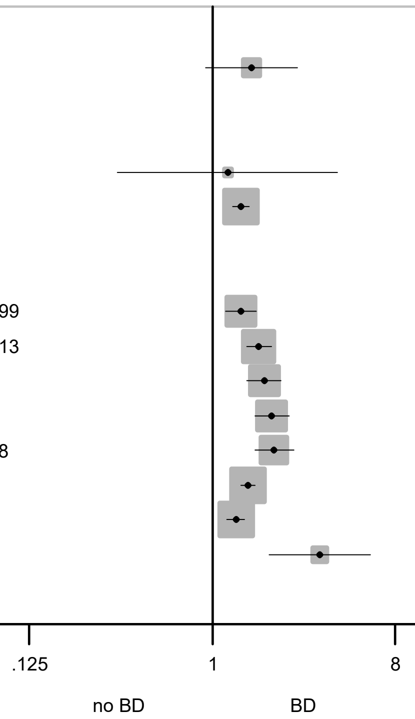

BD – bipolar disorder, NR – not reported, HR – hazard ratio, RRatio – rate ratio, OR – odds ratio, RR – risk ratio, P – persons, M – males, F - females

Fig E: Bipolar disorder, incidence of cerebrovascular accident, hazard ratios, rate ratios, and odds ratios

Study identifier	Gender	Type of effect	Region	Setting	Events/Total Exposed	Events/Total Unexposed	Effect size (95% CI)
**1980s**							
Gale, 2013 (M)	M	HR	Scandinavia	Inpatient	NR / 2566	NR / 1099304	1.62 (1.21, 2.17)
Prieto, 2016	P	HR	N. America	Community and inpatient	19 / 334	21 / 334	1.39 (0.75, 2.59)
**1990s**							
Laursen, 2010 (1999-2000)	P	RRatio	Scandinavia	Inpatient	NR / NR	NR / NR	1.14 (0.99, 1.32)
Laursen, 2010 (1994-1996)	P	RRatio	Scandinavia	Inpatient	NR / NR	NR / NR	1.08 (0.95, 1.23)
Laursen, 2010 (1997-1998)	P	RRatio	Scandinavia	Inpatient	NR / NR	NR / NR	1.22 (1.05, 1.41)
McDermott, 2005	P	RR	N. America	Community	13 / 146	183 / 2083	0.97 (0.50, 1.88)
Ramsey, 2010	P	OR	N. America	Community	5 / 58	59 / 1339	2.68 (1.51, 4.76)
Westman, 2013	P	RRatio	Scandinavia	Inpatient	419 / 17101	NR / 10631208	1.04 (0.94, 1.15)
**2000s**							
Crump, 2013b (M)	M	HR	Scandinavia	Community and inpatient	249 / 1102	253216 / 1575099	1.13 (1.00, 1.28)
Crump, 2013b (F)	F	HR	Scandinavia	Community and inpatient	258 / 2319	188367 / 1720013	1.22 (1.08, 1.38)
Hsu, 2021 (Ages 40-59)	P	RRatio	Far East	Community and inpatient	139 / 1848	40303 / 764579	1.88 (1.51, 2.34)
Hsu, 2021 (All ages)	P	RRatio	Far East	Community and inpatient	139 / 1848	40303 / 764579	1.60 (1.35, 1.89)
Hsu, 2021 (Ages 18-39)	P	RRatio	Far East	Community and inpatient	139 / 1848	40303 / 764579	2.62 (1.84, 3.73)
Jackson, 2020 (M)	M	RRatio	UK	Inpatient	NR / NR	NR / NR	1.60 (1.37, 1.87)
Jackson, 2020 (F)	F	RRatio	UK	Inpatient	NR / NR	NR / NR	2.50 (2.10, 2.98)
Kessing, 2021	P	HR	Scandinavia	Community and inpatient	901 / 18981	16267 / 366420	1.17 (1.09, 1.25)
Laursen, 2010 (2001-2002)	P	RRatio	Scandinavia	Inpatient	NR / NR	NR / NR	1.04 (0.89, 1.21)
Laursen, 2010 (2005-2006)	P	RRatio	Scandinavia	Inpatient	NR / NR	NR / NR	1.28 (1.12, 1.47)
Laursen, 2010 (2003-2004)	P	RRatio	Scandinavia	Inpatient	NR / NR	NR / NR	1.14 (0.99, 1.32)
Laursen, 2011	P	RRatio	Scandinavia	Inpatient	23 / 6215	6156 / 2428518	1.32 (0.88, 1.98)
Wium-Andersen, 2021	P	HR	Scandinavia	Community and inpatient	707 / 13725	10341 / 167562	1.07 (0.99, 1.16)
Wu, 2015	P	HR	Far East	Community and inpatient	243 / 12119	2527 / 207592	1.51 (1.28, 1.79)

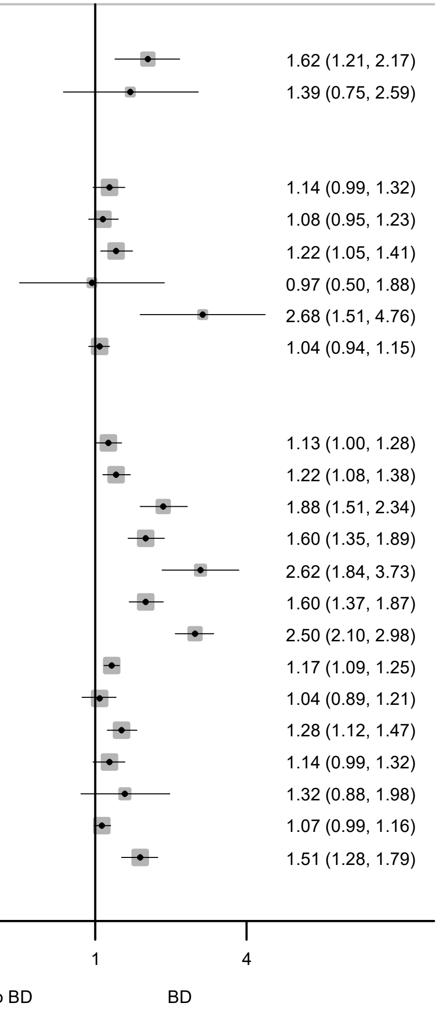

BD – bipolar disorder, NR – not reported, HR – hazard ratio, RRatio – rate ratio, OR – odds ratio, RR – risk ratio, P – persons, M – males, F - females

Fig F: Bipolar disorder, incidence of coronary heart disease, hazard ratios, rate ratios and odds ratios

Study identifier	Gender	Type of effect	Region	Setting	Events/Total Exposed	Events/Total Unexposed	Effect size (95% CI)
**1980s**							
Prieto, 2016	P	HR	N. America	Community and inpatient	45 / 334	46 / 334	1.54 (1.02, 2.33)
**1990s**							
Bent-Ennakhil, 2018	P	RRatio	Scandinavia	Community and inpatient	NR / 2373	NR / 200486	3.14 (Insufficient data)
**2000s**							
Foroughi, 2018	P	HR	N. America	Community	NR / 378	NR / 23251	1.30 (0.92, 1.84)
Foroughi, 2021	P	HR	N. America	Community	NR / 288	NR / 35326	1.78 (1.29, 2.46)
Goldstein, 2014 (BD II)	P	OR	N. America	Community	23 / 392	972 / 26266	2.76 (1.60, 4.75)
Goldstein, 2014 (BD I)	P	OR	N. America	Community	66 / 1047	972 / 26266	2.58 (1.84, 3.61)
Hayes, 2017	P	HR	UK	Community	382 / 17341	2445 / 219387	1.46 (1.31, 1.63)
**2010s**							
Vance, 2019 (M)	M	OR	N. America	Community	NR / 71471	NR / 810153	1.39 (1.35, 1.44)
Vance, 2019 (F)	F	OR	N. America	Community	NR / 1406	NR / 1407	2.03 (1.79, 2.31)

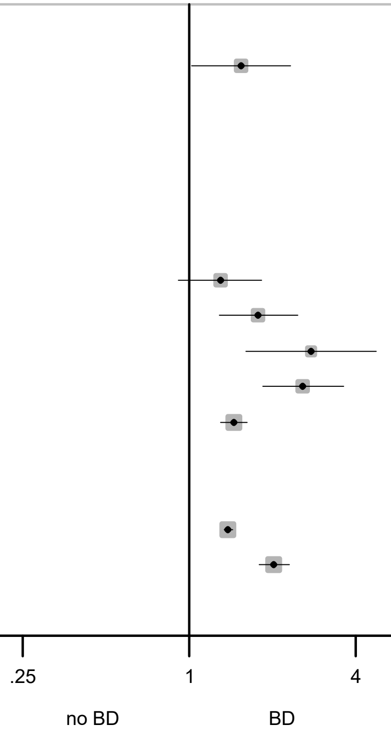

BD – bipolar disorder, NR – not reported, HR – hazard ratio, RRatio – rate ratio, OR – odds ratio, P – persons, M – males, F - females

Fig G: Bipolar disorder, incidence of major cardiovascular events, hazard ratios, rate ratios and odds ratios

Study identifier	Gender	Type of effect	Region	Setting	Events/Total Exposed	Events/Total Unexposed	Effect size (95% CI)
**1990s**							
McDermott, 2005	P	RR	N. America	Community	13 / 146	121 / 2083	1.73 (0.88, 3.40)
**2000s**							
Laursen, 2011	P	RRatio	Scandinavia	Inpatient	28 / 6215	3065 / 2428518	3.30 (2.27, 4.79)

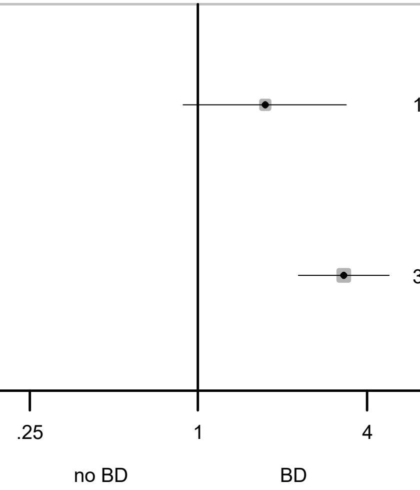

BD – bipolar disorder, RRatio – rate ratio, RR – risk ratio, P – persons

Fig H: Bipolar disorder, incidence of heart failure, hazard ratios, rate ratios and odds ratios
