# Supplementary material for: Temporal trends in associations between severe mental illness and risk of cardiovascular disease: A systematic review and meta-analysis
Source: PLoS Med. 2022 Apr 19;19(4):e1003960. doi: 10.1371/journal.pmed.1003960 (PMC9017899; doi:10.1371/journal.pmed.1003960)
Supplement: S16 File — Table A: Risk of bias assessment of studies reporting cardiovascular mortality outcomes, cohort studies. (DOCX) [file pmed.1003960.s016.docx]

# S16 File. Risk of bias assessment for included mortality studies

Table A: Risk of bias assessment of studies reporting cardiovascular mortality outcomes, cohort studies

|  | **Selection** | | | | **Comparability** | | | **Outcome** | | | |  |  |
| --- | --- | --- | --- | --- | --- | --- | --- | --- | --- | --- | --- | --- | --- |
| Study ID | Truly or somewhat representative of the average person with SMI in the community | Non-exposed drawn from the same community as the exposed cohort | Ascertainment of exposure from secure record *e.g.* medical record or structured interview | Demonstration that CVD was not present at start of study | Study controls for age and sex | Study controls for socio-demographic factor | Study controls for additional cardio-vascular risk factor | | Outcome accurately measured & validated, similar methods used for cases and controls, reliable system for measuring CVD occurrence | Follow-up long enough for outcomes to occur (min of 5 years) | Adequacy of follow-up of SMI and non-SMI cohorts | | |
| Ahrens, 1995 | N | N | Y | N | Y | N | N | | CT | CT | N | | |
| Ajetunmobi, 2013 | CT | N | Y | N | Y | Y | N | | CT | CT | CT | | |
| Allebeck, 1986 | CT | N | Y | N | y | N | N | | CT | Y | Y | | |
| Anderson, 1991 | Y | CT | Y | N | Y | N | N | | CT | CT | N | | |
| Angst, 2002 | N- | N | CT | N | Y | N | CT | | CT | Y | Y | | |
| Bjorkenstam, 2012 | CT | CT | Y | N | Y | N | CT | | CT | N | Y | | |
| Brink, 2018 | CT | Y | Y | Y | Y | Y | CT | | Y | Y | CT | | |
| Brodersen, 2000 | N | N | Y | N | Y | N | N | | CT | Y | Y | | |
| Brook, 1984 | N | N | Y | N | CT | N | N | | N | N | N | | |
| Brown, 2010 | Y | N | Y | N | Y | N | N | | CT | Y | Y | | |
| Buda, 1988 | CT | N | Y | N | Y | N | N | | CT | Y | Y | | |
| Callaghan, 2014 | CT | N | Y | N | Y | CT | N | | CT | CT | CT | | |
| Casadebaig, 1999 | CT | N | Y | N | Y | N | N | | CT | N | N | | |
| Castagnini, 2013 | Y | N | Y | N | Y | N | N | | CT | CT | Y | | |
| Chan, 2021 | Y | N | Y | N | Y | N | N | | CT | CT | CT | | |
| Crump, 2013a | Y | CT | Y | CT | Y | Y | Y | | CT | Y | Y | | |
| Crump, 2013b | Y | CT | Y | CT | Y | Y | Y | | CT | Y | Y | | |
| Cunningham, 2014 | Y | N | Y | N | Y | N | N | | CT | N | CT | | |
| Curkendall, 2004 | CT | Y | CT | CT | Y | N | Y | | CT | N | N | | |
| Dalgard, 1966 | CT | CT | CT | N | Y | Y | N | | CT | Y | Y | | |
| Das-Munshi, 2017 | Y | N | Y | N | Y | CT | N | | CT | CT | CT | | |
| Daumit, 2010 | CT | N | CT | N | Y | Y | N | | CT | Y | N | | |
| Dutta, 2012 | Y | N | Y | N | Y | N | N | | CT | CT | CT | | |
| Fors, 2007 | Y | Y | Y | N | Y | CT | N | | CT | Y | Y | | |
| Giel, 1978 | N | N | CT | N | CT | N | N | | N | N | N | | |
| Girardi, 2021 | Y | N | Y | N | Y | N | N | | CT | Y | CT | | |
| Grigoletti, 2009 | Y | N | Y | N | CT | N | N | | CT | Y | N | | |
| Hansen, 2001 | CT | N | Y | N | Y | N | N | | CT | CT | Y | | |
| Hayes, 2017 | Y | Y | Y | N | Y | Y | Y | | CT | N | N | | |
| Heiberg, 2018 | CT | N | Y | N | Y | N | N | | CT | N | Y | | |
| Heila, 2005 | CT | N | Y | N | Y | N | N | | CT | CT | Y | | |
| Hiroeh, 2008 | CT | CT | CT | N | Y | N | N | | CT | Y | Y | | |
| Hoang, 2011 | CT | N | Y | N | Y | N | N | | CT | N | N | | |
| Hoye, 2011 | CT | N | Y | N | Y | N | N | | CT | Y | Y | | |
| John, 2018 | Y | N | Y | N | Y | N | N | | CT | CT | N | | |
| Kelly, 2010 | N | N | Y | N | Y | CT | N | | CT | Y | N | | |
| Kilbourne, 2009 | N | CT | Y | N | CT | Y | Y | | CT | CT | N | | |
| Kiviniemi, 2010 | CT | N | Y | N | Y | CT | N | | CT | Y | Y | | |
| Kredentser, 2014 | CT | N | Y | N | CT | N | N | | CT | Y | N | | |
| Lahti, 2012 | CT | CT | Y | CT | Y | CT | CT | | CT | Y | N | | |
| Laursen, 2007 | CT | CT | Y | N | Y | N | N | | CT | Y | Y | | |
| Laursen, 2010 | CT | CT | Y | Y | Y | N | N | | CT | CT | Y | | |
| Laursen, 2013 | CT | N | Y | N | Y | N | N | | CT | CT | Y | | |
| Laursen, 2014 | CT | CT | Y | CT | Y | N | CT | | CT | Y | Y | | |
| Laursen, 2019 | Y | CT | Y | N | Y | N | N | | CT | Y | Y | | |
| Lawrence, 2003 | Y | N | Y | N | Y | N | N | | CT | Y | N | | |
| Lawrence, 2013 | Y | N | Y | N | Y | N | N | | CT | Y | N | | |
| Lemogne, 2013 | N | Y | CT | N | Y | CT | Y | | CT | Y | Y | | |
| Lesage, 2015 | CT | CT | N | N | Y | N | N | | CT | CT | N | | |
| Lumme, 2016 | CT | N | Y | N | Y | N | N | | CT | CT | Y | | |
| Manderbacka, 2012 | CT | N | Y | N | Y | N | N | | CT | Y | Y | | |
| Morden, 2012 | N | Y | Y | N | Y | N | N | | CT | CT | N | | |
| Mortensen, 1990 | N | N | Y | N | Y | N | N | | CT | Y | Y | | |
| Mortensen, 1993 | CT | N | Y | N | Y | N | N | | CT | y | Y | | |
| Murray-Thomas, 2013 | CT | Y | Y | CT | Y | N | N | | CT | N | CT | | |
| Newman, 1991 | Y | N | CT | N | Y | N | N | | CT | CT | CT | | |
| Nilsson, 1995 | N | N | Y | N | Y | N | N | | CT | Y | Y | | |
| Nordentoft, 2013 | CT | N | Y | N | Y | N | N | | CT | N | Y | | |
| Odegard, 1967 | N | N | Y | N | Y | N | N | | N | N | N | | |
| Olfson, 2015 | CT | N | CT | N | Y | CT | N | | CT | N | CT | | |
| Osborn, 2007 | Y | Y | Y | N | Y | CT | CT | | CT | N | N | | |
| Osby, 2000a | CT | N | Y | N | Y | N | N | | CT | Y | Y | | |
| Osby, 2000b | CT | N | Y | N | Y | N | N | | CT | Y | Y | | |
| Osby, 2001 | CT | N | CT | N | Y | N | N | | CT | CT | Y | | |
| Osby, 2016 | CT | N | Y | N | Y | N | N | | CT | N | Y | | |
| Pan, 2020 | Y | CT | Y | N | Y | N | N | | CT | N | Y | | |
| Park, 2015 | CT | N | Y | N | CT | N | N | | CT | Y | CT | | |
| Prior, 1996 | Y | N | CT | N | Y | N | N | | CT | CT | CT | | |
| Saku, 1995 | N | N | Y | N | Y | N | N | | CT | Y | N | | |
| Salazar-Fraile, 1998 | Y | CT | Y | N | Y | N | N | | CT | Y | Y | | |
| Sanchez, 2021 | Y | CT | Y | Y | Y | N | Y | | Y | N | N | | |
| Tanskanen, 2018 | CT | N | Y | N | CT | N | N | | CT | Y | Y | | |
| Termorshuizen, 2013 | Y | Y | CT | N | Y | CT | N | | CT | CT | N | | |
| Torniainen, 2015 | Y | Y | Y | N | Y | N | N | | CT | Y | Y | | |
| Tsuang, 1980 | N | N | Y | N | Y | N | N | | CT | Y | Y | | |
| Vance, 2019 | N | CT | Y | Y | Y | CT | Y | | Y | N | N | | |
| Weeke, 1986 | CT | N | CT | N | Y | N | N | | CT | Y | Y | | |
| Weeke, 1987 | N | N | Y | N | Y | N | N | | CT | N | Y | | |
| Westman, 2013 | CT | CT | Y | CT | Y | N | N | | CT | Y | Y | | |
| Westman, 2017 | CT | CT | Y | CT | Y | N | N | | CT | Y | Y | | |
| Yung, 2021 | Y | N | Y | N | Y | N | N | | CT | CT | CT | | |
| Zilber, 1989 | N | N | Y | N | Y | N | N | | CT | N | N | | |
| *Y – yes, N – no, CT – unclear*  Cohort studies with low risk of bias were denoted by a rating of “yes” or “can’t tell” on the “selection” and “outcome” criteria (excluding selection criteria on representativeness of people with SMI), together with adjustment for age, sex and at least two other confounding factors in the “comparability” category | | | | | | | | | | | | |  |
