# Supplementary material for: Temporal trends in associations between severe mental illness and risk of cardiovascular disease: A systematic review and meta-analysis
Source: PLoS Med. 2022 Apr 19;19(4):e1003960. doi: 10.1371/journal.pmed.1003960 (PMC9017899; doi:10.1371/journal.pmed.1003960)
Supplement: S17 File — Table A: Risk of bias assessment of studies reporting cardiovascular incidence outcomes, cohort studies. Table B: Risk of bias assessment of studies reporting cardiovascular incidence outcomes, case–control studies. (DOCX) [file pmed.1003960.s017.docx]

# S17 File. Risk of bias assessment for included incidence studies

Table A: Risk of bias assessment of studies reporting cardiovascular incidence outcomes, cohort studies

|  | **Selection** | | | | | | | | **Comparability** | | | | | | **Outcome** | | | | | |  |
| --- | --- | --- | --- | --- | --- | --- | --- | --- | --- | --- | --- | --- | --- | --- | --- | --- | --- | --- | --- | --- | --- |
| Study ID | Truly or somewhat representative of the average person with SMI in the community | | Non-exposed drawn from the same community as the exposed cohort | | Ascertainment of exposure from secure record *eg* medical record or structured interview | | Demonstration that CVD was not present at start of study | | Study controls for age and sex | | Study controls for socio-demographic factors | | Study controls for additional cardio-vascular risk factors | | Outcome accurately measured & validated, similar methods used for cases and controls, reliable system for measuring CVD occurrence | | Follow-up long enough for outcomes to occur (min of 5 years) | | Adequacy of follow up of SMI and non-SMI cohorts | |  |
| Bent-Ennakhil, 2018 | | Y | | CT | | Y | | Y | | Y | | N | | N | | Y | | Y | | Y | |
| Brink, 2018 | | CT | | Y | | Y | | Y | | Y | | Y | | CT | | Y | | Y | | CT | |
| Carney, 2006 | | CT | | Y | | CT | | N | | Y | | Y | | N | | CT | | N | | N | |
| Chen, 2015 | | Y | | Y | | Y | | CT | | Y | | N | | N | | CT | | CT | | Y | |
| Crump, 2013a | | Y | | CT | | Y | | CT | | Y | | Y | | Y | | CT | | Y | | Y | |
| Crump, 2013b | | Y | | CT | | Y | | CT | | Y | | Y | | Y | | CT | | Y | | Y | |
| Curkendall, 2004 | | CT | | Y | | CT | | CT | | Y | | N | | Y | | CT | | N | | N | |
| Foroughi, 2018 | | Y | | Y | | CT | | CT | | Y | | N | | N | | CT | | Y | | N | |
| Foroughi, 2021 | | Y | | CT | | Y | | Y | | Y | | N | | Y | | Y | | Y | | CT | |
| Gale, 2013 | | N | | Y | | Y | | CT | | Y | | Y | | Y | | CT | | Y | | CT | |
| Goldstein, 2014 | | CT | | Y | | N | | CT | | CT | | CT | | Y | | N | | N | | N | |
| Gur, 2017 | | Y | | Y | | Y | | N | | Y | | CT | | N | | Y | | CT | | N | |
| Hayes, 2017 | | Y | | Y | | Y | | N | | Y | | Y | | Y | | CT | | N | | N | |
| Hsu, 2021 | | Y | | CT | | Y | | CT | | CT | | Y | | N | | CT | | N | | Y | |
| Jackson, 2020 | | CT | | CT | | Y | | CT | | Y | | CT | | N | | CT | | Y | | N | |
| Jakobsen, 2008 | | CT | | Y | | Y | | Y | | Y | | N | | N | | CT | | Y | | Y | |
| Kessing, 2021 | | Y | | Y | | Y | | CT | | Y | | CT | | N | | CT | | Y | | Y | |
| Kugathasan, 2018 | | Y | | Y | | Y | | CT | | Y | | N | | N | | CT | | Y | | Y | |
| Lahti, 2012 | | CT | | CT | | Y | | CT | | Y | | CT | | CT | | CT | | Y | | N | |
| Laursen, 2010 | | CT | | CT | | Y | | Y | | Y | | N | | N | | CT | | CT | | Y | |
| Laursen, 2011 | | CT | | CT | | Y | | CT | | Y | | N | | N | | CT | | Y | | Y | |
| Lawrence, 2003 | | Y | | N | | Y | | N | | Y | | N | | N | | CT | | Y | | N | |
| Lin, 2010 | | CT | | CT | | Y | | CT | | Y | | Y | | Y | | CT | | Y | | Y | |
| Manderbacka, 2012 | | CT | | N | | Y | | N | | Y | | N | | N | | CT | | Y | | Y | |
| McDermott, 2005 | | Y | | Y | | Y | | N | | Y | | CT | | Y | | CT | | CT | | N | |
| Momen, 2020 | | Y | | Y | | Y | | CT | | Y | | N | | N | | CT | | Y | | Y | |
| Morden, 2012 | | N | | Y | | Y | | N | | Y | | N | | N | | CT | | CT | | N | |
| Munk-Jorgensen, 2000 | | CT | | Y | | Y | | N | | Y | | N | | N | | N | | Y | | Y | |
| Prieto, 2016 | | Y | | Y | | Y | | CT | | Y | | N | | Y | | Y | | Y | | CT | |
| Ramsey, 2010 | | CT | | Y | | N | | CT | | Y | | CT | | Y | | N | | Y | | N | |
| Sanchez, 2021 | | Y | | CT | | Y | | Y | | Y | | N | | Y | | Y | | N | | N | |
| Sundquist, 2006 | | CT | | CT | | Y | | CT | | Y | | Y | | CT | | CT | | Y | | Y | |
| Tsai, 2012 | | CT | | Y | | Y | | CT | | Y | | Y | | Y | | CT | | N | | Y | |
| Vance, 2019 | | N | | CT | | Y | | Y | | Y | | CT | | Y | | Y | | N | | N | |
| Westman, 2013 | | CT | | CT | | Y | | CT | | Y | | N | | N | | CT | | Y | | Y | |
| Westman, 2017 | | CT | | CT | | Y | | CT | | Y | | N | | N | | CT | | Y | | Y | |
| Wium-Andersen, 2021 | | Y | | Y | | Y | | CT | | Y | | Y | | CT | | CT | | CT | | Y | |
| Wu, 2013 | | CT | | Y | | Y | | CT | | Y | | Y | | Y | | CT | | N | | Y | |
| Wu, 2015 | | Y | | CT | | Y | | N | | Y | | Y | | Y | | CT | | Y | | Y | |

Y= yes, N – no, CT – can’t tell

Cohort studies with low risk of bias were denoted by a rating of “yes” or “can’t tell” on the “selection” and “outcome” criteria (excluding selection criteria on representativeness of people with SMI), together with adjustment for age, sex and at least two other confounding factors in the “comparability” category

Table B: Risk of bias assessment of studies reporting cardiovascular incidence outcomes, case-control studies

|  | **Selection** | | | | **Comparability** | | | **Exposure** | | | |
| --- | --- | --- | --- | --- | --- | --- | --- | --- | --- | --- | --- |
| Study ID | Adequate definition of cases with CVD | Cases with CVD are representative of defined population and reliable system used for selecting cases | Selection of controls without CVD | Definition of controls - demonstration of no history of CVD | Study controls for age and sex | Study controls for additional sociodemographic factors | Study controls for additional lifestyle factors | Ascertainment of exposure from secure record *eg* medical record or structured interview | Does SMI exposure precede CVD outcome? | Same method of ascertainment for cases and controls | Non-response rate |
| Yu-Chuan Chiu, 2015 | Y | CT | Y | CT | Y | Y | Y | Y | Y | Y | CT |

Y= yes, N – no, CT – can’t tell

*Case-control studies with low risk of bias were denoted by “yes” or “can’t tell” in “selection” and “exposure” categories, plus adjustment for age, sex and at least two other confounding factors in the “comparability” category*
