# Supplementary material for: Temporal trends in associations between severe mental illness and risk of cardiovascular disease: A systematic review and meta-analysis
Source: PLoS Med. 2022 Apr 19;19(4):e1003960. doi: 10.1371/journal.pmed.1003960 (PMC9017899; doi:10.1371/journal.pmed.1003960)
Supplement: S18 File — Fig A: Forest plots showing relative risk of CVD incidence in those with versus without schizophrenia, studies included in meta-analysis. Fig B: Forest plots showing relative risk of CVD incidence in those with versus without BD, studies included in meta-analysis. BD, bipolar disorder; CVD, cardiovascular disease. (PDF) [file pmed.1003960.s018.pdf]

S18 File. Meta-analysis of CVD incidence outcomes

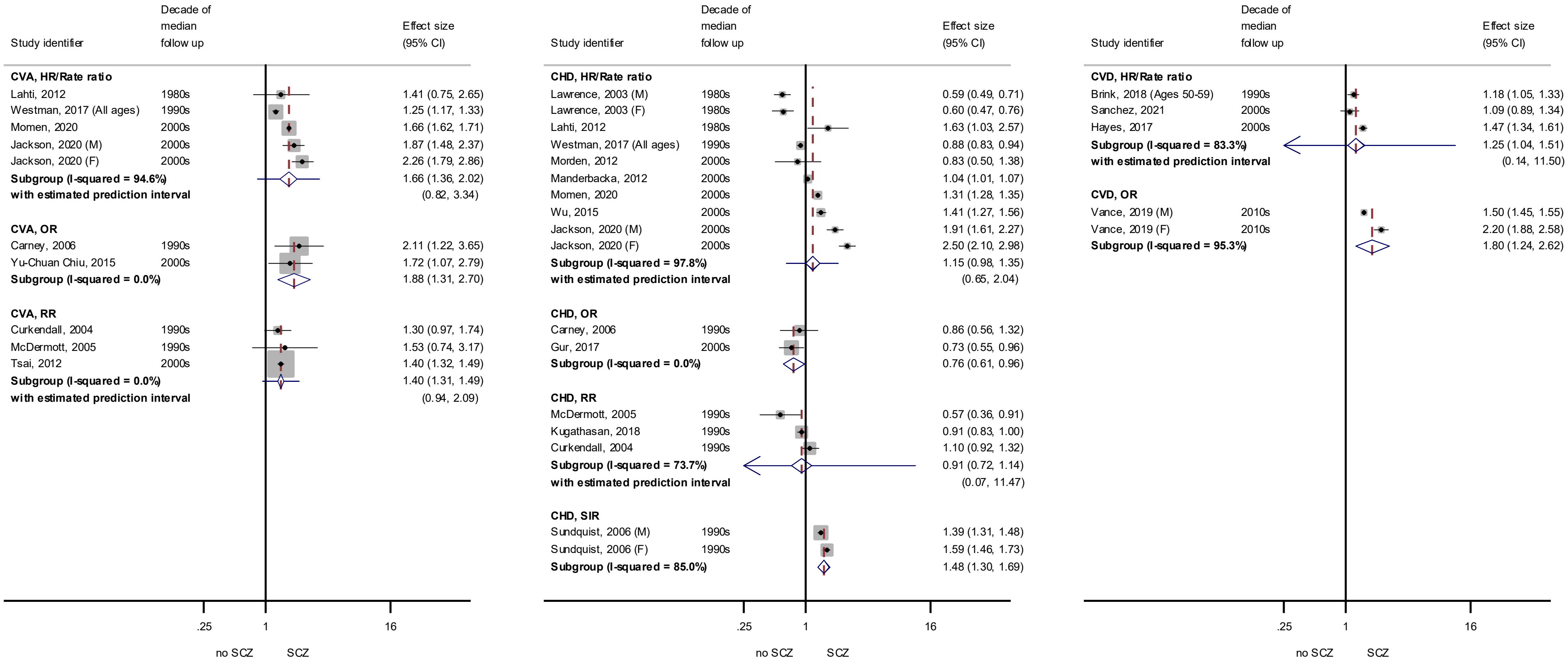

Weights are from random effects mode. Dashed lines show pooled estimated risks within subgroups
Studies were allocated to decades according to the median year of follow-up
SCZ – schizophrenia, CVA – cerebrovascular accident, CHD – coronary heart disease, CVD – major cardiovascular events, HR – hazard ratio, OR – odds ratio, SIR – standardised incidence ratio, RR – risk ratio, M – males, F – females

Fig A: Forest plots showing relative risk of CVD incidence in those with vs without schizophrenia, studies included in meta-analysis

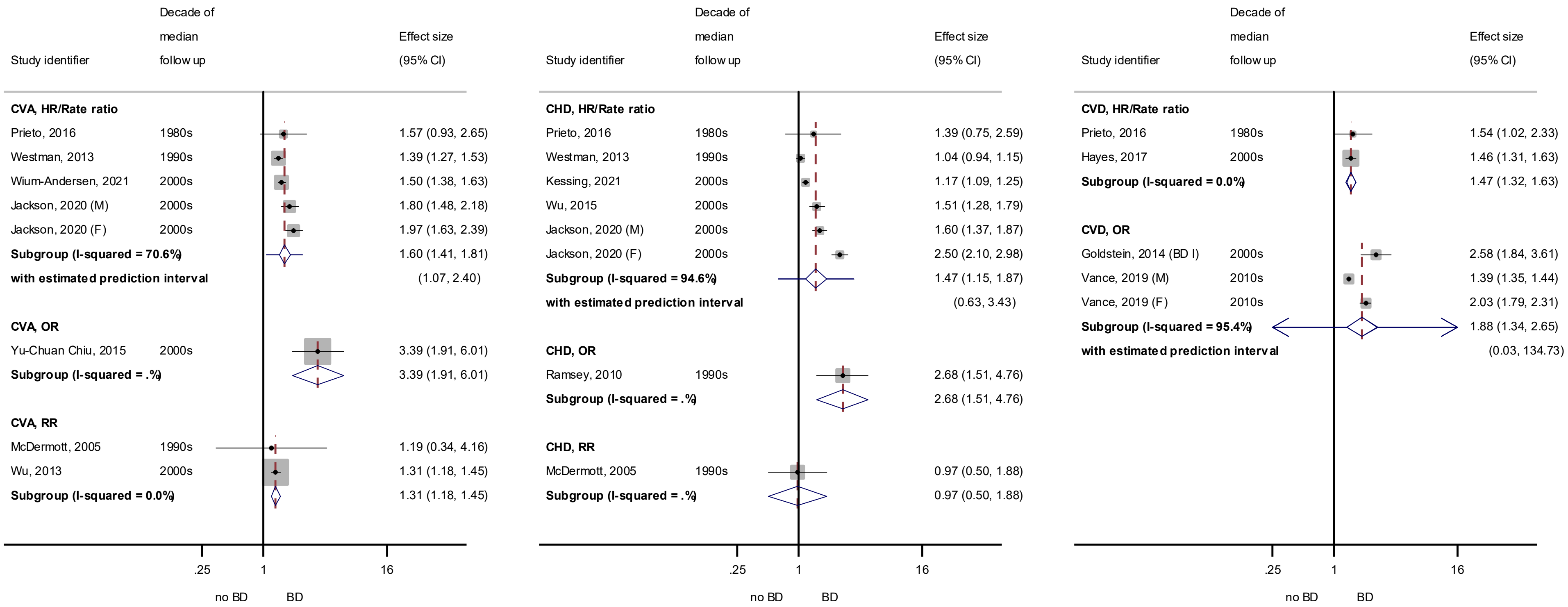

Weights are from random effects mode. Dashed lines show pooled estimated risks within subgroups
Studies were allocated to decades according to the median year of follow-up
BD – bipolar disorder, CVA – cerebrovascular accident, CHD – coronary heart disease, CVD – major cardiovascular events, HR – hazard ratio, OR – odds ratio, RR – risk ratio, M – males, F – females

Fig B: Forest plots showing relative risk of CVD incidence in those with vs without bipolar disorder, studies included in meta-analysis
