# Supplementary material for: Temporal trends in associations between severe mental illness and risk of cardiovascular disease: A systematic review and meta-analysis
Source: PLoS Med. 2022 Apr 19;19(4):e1003960. doi: 10.1371/journal.pmed.1003960 (PMC9017899; doi:10.1371/journal.pmed.1003960)
Supplement: S19 File — Fig A: Forest plot showing pooled estimates of cardiovascular mortality ratios by SMI, outcome and decade. Fig B: Forest plot showing pooled estimates of cardiovascular incidence ratios by SMI, outcome and decade. Table A: Meta-analysis: pooled results by SMI and cardiovascular mortality. Table B: Meta-analysis: pooled results by SMI and cardiovascular incidence. Table C: Results of meta-regressions: estimates of increased effect size relative to the reference category by median 5-year calendar period of outcome, exponentiated regression coefficients (95% CIs,) p-values. Fig C: Trend in risk of CVD mortality for SMI compared with controls, by median 5-year calendar period of outcome. Fig D: Trend in risk of CVD incidence for SMI compared with controls, by median 5-year calendar period of outcome. CI, confidence interval; CVD, cardiovascular disease; SMI, severe mental illness. (PDF) [file pmed.1003960.s019.pdf]

S19 File. Analysis of temporal trends

SMI	Outcome	Measure	Decade	Studies	Results	Effect size (95% CI)
SCZ	CVA	HR/rate ratio	1980s	1	1	1.65 (0.52, 5.22)
SCZ	CVA	HR/rate ratio	1990s	2	2	2.40 (2.26, 2.56)
SCZ	CVA	SMR	1950s-70s	4	4	1.56 (1.01, 2.39)
SCZ	CVA	SMR	1980s	3	3	1.78 (1.05, 3.00)
SCZ	CVA	SMR	1990s	3	3	3.14 (2.78, 3.55)
SCZ	CVA	SMR	2000s	2	4	2.21 (1.98, 2.48)
SCZ	CVA	SMR	2010s	2	2	1.54 (0.85, 2.80)
SCZ	CHD	HR/rate ratio	1980s	3	6	1.78 (1.55, 2.05)
SCZ	CHD	HR/rate ratio	1990s	2	2	2.83 (2.73, 2.94)
SCZ	CHD	HR/rate ratio	2000s	2	2	1.35 (1.30, 1.40)
SCZ	CHD	SMR	1950s-70s	4	4	1.62 (1.43, 1.83)
SCZ	CHD	SMR	1980s	3	3	1.14 (0.57, 2.30)
SCZ	CHD	SMR	1990s	3	3	2.29 (2.11, 2.49)
SCZ	CHD	SMR	2000s	2	4	2.59 (1.93, 3.47)
SCZ	CHD	SMR	2010s	2	2	2.20 (1.05, 4.58)
SCZ	CVD	HR/rate ratio	1980s	1	1	1.20 (0.47, 3.04)
SCZ	CVD	HR/rate ratio	1990s	2	2	2.41 (1.74, 3.35)
SCZ	CVD	HR/rate ratio	2000s	7	9	1.86 (1.44, 2.40)
SCZ	CVD	SMR	1950s-70s	3	4	1.68 (1.41, 2.00)
SCZ	CVD	SMR	1980s	2	2	0.71 (0.19, 2.69)
SCZ	CVD	SMR	1990s	5	5	2.12 (1.59, 2.84)
SCZ	CVD	SMR	2000s	5	6	2.68 (2.15, 3.34)
SCZ	CVD	SMR	2010s	3	3	2.53 (1.32, 4.84)
SCZ	CVD	OR	2010s	1	2	2.55 (1.74, 3.74)
SCZ	HF	HR/rate ratio	1990s	1	1	3.25 (2.94, 3.60)
BD	CVA	HR/rate ratio	1990s	2	2	2.00 (1.81, 2.22)
BD	CVA	SMR	1950s-70s	1	1	1.37 (0.89, 2.10)
BD	CVA	SMR	1980s	1	1	1.58 (0.82, 3.03)
BD	CVA	SMR	1990s	1	1	1.30 (1.20, 1.40)
BD	CVA	SMR	2000s	1	3	1.75 (1.59, 1.93)
BD	CVA	SMR	2010s	1	1	1.30 (0.93, 1.81)
BD	CHD	HR/rate ratio	1980s	1	2	1.63 (1.52, 1.74)
BD	CHD	HR/rate ratio	1990s	3	4	1.93 (1.59, 2.35)
BD	CHD	HR/rate ratio	2000s	1	1	1.24 (1.13, 1.36)
BD	CHD	SMR	1950s-70s	1	1	1.84 (1.43, 2.37)
BD	CHD	SMR	1980s	1	1	1.00 (0.69, 1.44)
BD	CHD	SMR	1990s	1	1	1.66 (1.59, 1.74)
BD	CHD	SMR	2000s	1	3	1.77 (1.65, 1.90)
BD	CHD	SMR	2010s	1	1	1.33 (1.00, 1.77)
BD	CVD	HR/rate ratio	1990s	1	1	2.03 (1.93, 2.13)
BD	CVD	HR/rate ratio	2000s	1	1	1.11 (0.85, 1.45)
BD	CVD	SMR	1950s-70s	6	6	1.47 (1.31, 1.65)
BD	CVD	SMR	1990s	2	2	1.54 (1.47, 1.60)
BD	CVD	SMR	2000s	3	5	1.86 (1.75, 1.99)
BD	CVD	SMR	2010s	1	1	1.33 (1.11, 1.60)
BD	CVD	OR	2010s	1	2	1.83 (1.57, 2.14)
SMI	CVA	SMR	2000s	1	2	2.12 (0.52, 8.67)
SMI	CHD	SMR	2000s	1	2	3.05 (2.51, 3.69)
SMI	CVD	HR/rate ratio	2000s	1	1	5.41 (2.40, 12.20)
SMI	CVD	SMR	1980s	2	3	1.82 (1.28, 2.58)
SMI	CVD	SMR	2000s	1	2	3.44 (3.09, 3.84)
SMI	CVD	SMR	2010s	1	1	2.65 (2.45, 2.86)

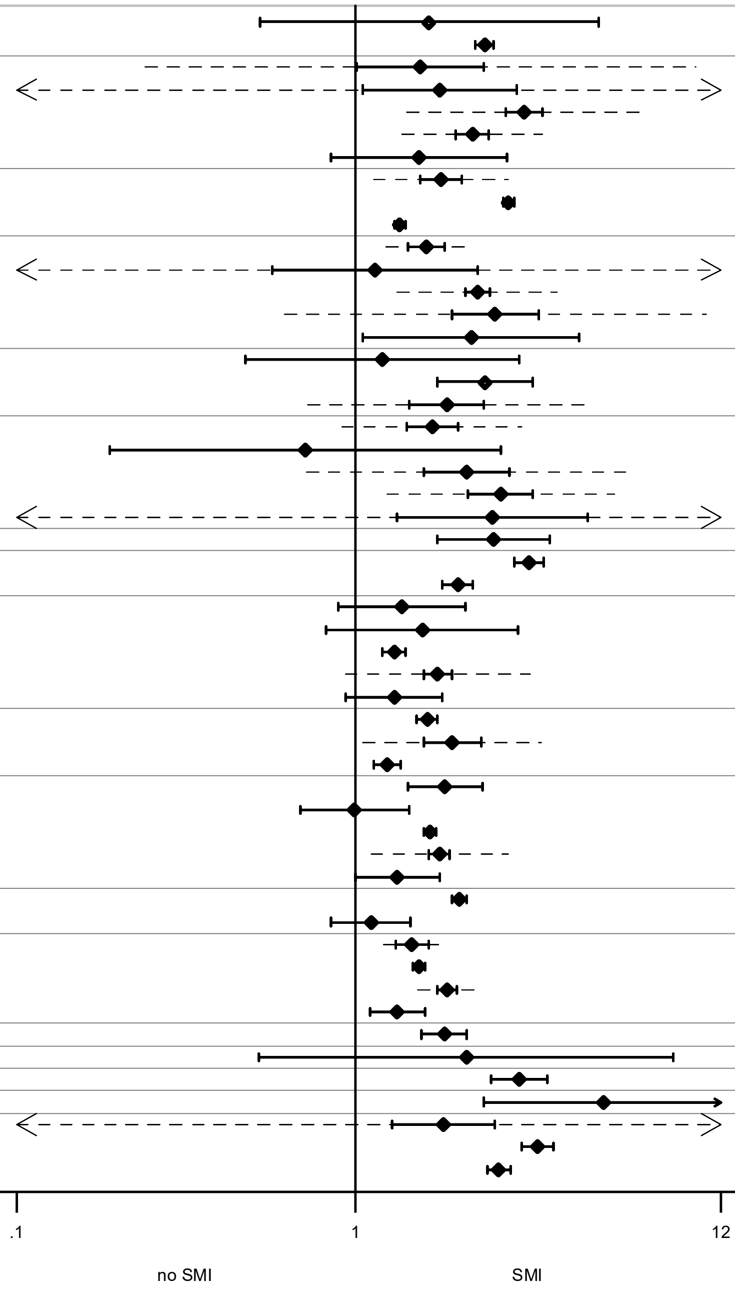

SCZ – schizophrenia, BD – bipolar disorder, SMI – mixed schizophrenia and bipolar disorder, CHD – coronary heart disease, CVA – cerebrovascular accident, CVD – all circulatory disease, HR – hazard ratio, OR – odds ratio, SMR – standardised mortality ratio

Pooled estimates with 95% confidence intervals (solid lines) and 95% prediction intervals if at least 3 results (dashed lines)

Fig A: Forest plot showing pooled estimates of cardiovascular mortality ratios by severe mental illness, outcome and decade

SMI	Outcome	Measure	Decade	Studies	Results	Effect size (95% CI)
SCZ	CVA	HR/rate ratio	1980s	1	1	1.41 (0.75, 2.65)
SCZ	CVA	HR/rate ratio	1990s	1	1	1.25 (1.17, 1.33)
SCZ	CVA	HR/rate ratio	2000s	2	3	1.86 (1.54, 2.25)
SCZ	CVA	OR	1990s	1	1	2.11 (1.22, 3.65)
SCZ	CVA	OR	2000s	1	1	1.72 (1.07, 2.79)
SCZ	CVA	RR	1990s	2	2	1.33 (1.01, 1.75)
SCZ	CVA	RR	2000s	1	1	1.40 (1.32, 1.49)
SCZ	CHD	HR/rate ratio	1980s	2	3	0.79 (0.50, 1.24)
SCZ	CHD	HR/rate ratio	1990s	1	1	0.88 (0.83, 0.94)
SCZ	CHD	HR/rate ratio	2000s	5	6	1.46 (1.22, 1.74)
SCZ	CHD	OR	1990s	1	1	0.86 (0.56, 1.32)
SCZ	CHD	OR	2000s	1	1	0.73 (0.55, 0.96)
SCZ	CHD	RR	1990s	3	3	0.91 (0.72, 1.14)
SCZ	CHD	SIR	1990s	1	2	1.48 (1.30, 1.69)
SCZ	CVD	HR/rate ratio	1990s	1	1	1.18 (1.05, 1.33)
SCZ	CVD	HR/rate ratio	2000s	2	2	1.29 (0.96, 1.72)
SCZ	CVD	OR	2010s	1	2	1.80 (1.24, 2.62)
SCZ	HF	HR/rate ratio	2000s	1	1	3.82 (3.10, 4.71)
SCZ	HF	OR	1990s	1	1	2.38 (1.42, 4.00)
SCZ	HF	OR	2000s	1	1	1.37 (0.85, 2.21)
SCZ	HF	RR	1990s	2	2	1.72 (1.08, 2.75)
BD	CVA	HR/rate ratio	1980s	1	1	1.57 (0.93, 2.65)
BD	CVA	HR/rate ratio	1990s	1	1	1.39 (1.27, 1.53)
BD	CVA	HR/rate ratio	2000s	2	3	1.72 (1.43, 2.06)
BD	CVA	OR	2000s	1	1	3.39 (1.91, 6.01)
BD	CVA	RR	1990s	1	1	1.19 (0.34, 4.16)
BD	CVA	RR	2000s	1	1	1.31 (1.18, 1.45)
BD	CHD	HR/rate ratio	1980s	1	1	1.39 (0.75, 2.59)
BD	CHD	HR/rate ratio	1990s	1	1	1.04 (0.94, 1.15)
BD	CHD	HR/rate ratio	2000s	3	4	1.62 (1.17, 2.24)
BD	CHD	OR	1990s	1	1	2.68 (1.51, 4.76)
BD	CHD	RR	1990s	1	1	0.97 (0.50, 1.88)
BD	CVD	HR/rate ratio	1980s	1	1	1.54 (1.02, 2.33)
BD	CVD	HR/rate ratio	2000s	1	1	1.46 (1.31, 1.63)
BD	CVD	OR	2000s	1	1	2.58 (1.84, 3.61)
BD	CVD	OR	2010s	1	2	1.67 (1.15, 2.42)
BD	HF	HR/rate ratio	2000s	1	1	3.30 (2.27, 4.79)
BD	HF	RR	1990s	1	1	1.73 (0.88, 3.40)

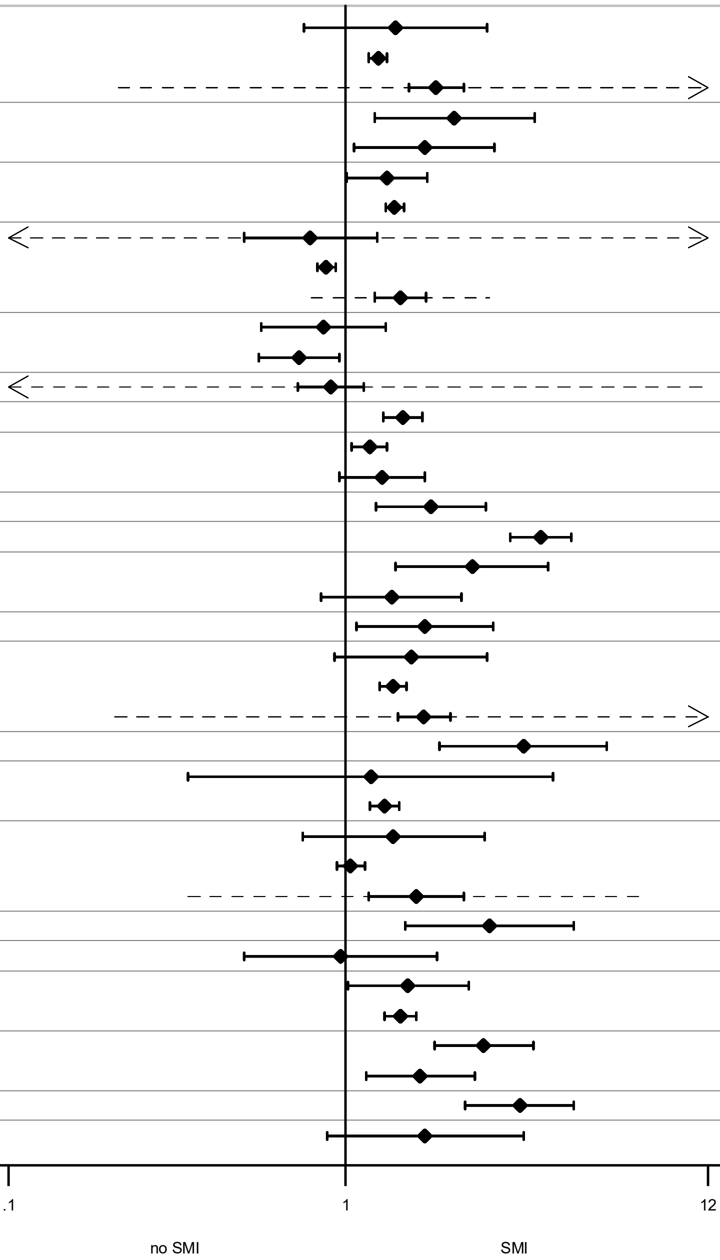

SCZ – schizophrenia, BD – bipolar disorder, CHD – coronary heart disease, CVA – cerebrovascular accident, CVD – major cardiovascular events, HF – heart failure, HR – hazard ratio, OR – odds ratio, RR – risk ratio, SIR – standardised incidence ratio
Pooled estimates with 95% confidence intervals (solid lines) and 95% prediction intervals if at least 3 results (dashed lines)

Fig B: Forest plot showing pooled estimates of cardiovascular incidence ratios by severe mental illness, outcome and decade

Table A: Meta-analysis: pooled results by severe mental illness and cardiovascular mortality

SMI	Outcome	Measure	Decade	Studies	Results	Effect	95% CI	p	Lower prediction interval	Upper prediction interval	I^2	Q	Q df
SCZ	CVA	HR/RRatio	1980s	1	1	1.65	(0.52 - 5.22)	0.394	.	.	.	0.0	0
SCZ	CVA	HR/RRatio	1990s	2	2	**2.40**	(2.26 - 2.56)	<0.001	.	.	0.0	0.1	1
SCZ	CVA	SMR	1950-70s	4	4	**1.56**	(1.01 - 2.40)	0.045	0.2	10.1	74.2	11.6	3
SCZ	CVA	SMR	1980s	3	3	**1.78**	(1.05 - 3.00)	0.032	0.0	620.7	66.1	5.9	2
SCZ	CVA	SMR	1990s	3	3	**3.14**	(2.78 - 3.55)	<0.001	1.4	6.9	0.0	0.6	2
SCZ	CVA	SMR	2000s	2	4	**2.21**	(1.98 - 2.48)	<0.001	1.4	3.6	74.4	11.7	3
SCZ	CVA	SMR	2010s	2	2	1.54	(0.85 - 2.80)	0.158	.	.	95.6	22.5	1
SCZ	CHD	HR/RRatio	1980s	3	6	**1.78**	(1.55 - 2.05)	<0.001	1.1	2.8	90.6	53.1	5
SCZ	CHD	HR/RRatio	1990s	2	2	**2.83**	(2.73 - 2.94)	<0.001	.	.	0.0	0.7	1
SCZ	CHD	HR/RRatio	2000s	2	2	**1.35**	(1.31 - 1.41)	<0.001	.	.	0.0	0.1	1
SCZ	CHD	SMR	1950-70s	4	4	**1.62**	(1.43 - 1.83)	<0.001	1.2	2.1	0.0	1.2	3
SCZ	CHD	SMR	1980s	3	3	1.14	(0.57 - 2.30)	0.710	0.0	6779.8	89.6	19.3	2
SCZ	CHD	SMR	1990s	3	3	**2.29**	(2.11 - 2.49)	<0.001	1.3	3.9	0.0	0.0	2
SCZ	CHD	SMR	2000s	2	4	**2.59**	(1.93 - 3.47)	<0.001	0.6	10.8	98.7	222.9	3
SCZ	CHD	SMR	2010s	2	2	**2.20**	(1.05 - 4.59)	0.036	.	.	98.8	82.3	1
SCZ	CVD	HR/RRatio	1980s	1	1	1.20	(0.47 - 3.04)	0.700	.	.	.	0.0	0
SCZ	CVD	HR/RRatio	1990s	2	2	**2.41**	(1.74 - 3.35)	<0.001	.	.	88.4	8.6	1
SCZ	CVD	HR/RRatio	2000s	7	9	**1.86**	(1.44 - 2.40)	<0.001	0.7	4.8	98.4	512.9	8
SCZ	CVD	OR	2010s	1	2	**2.55**	(1.74 - 3.74)	<0.001	.	.	91.8	12.1	1
SCZ	CVD	SMR	1950-70s	3	4	**1.68**	(1.41 - 2.00)	<0.001	0.9	3.1	39.1	4.9	3
SCZ	CVD	SMR	1980s	2	2	0.71	(0.19 - 2.69)	0.616	.	.	98.1	51.6	1
SCZ	CVD	SMR	1990s	5	5	**2.13**	(1.59 - 2.84)	<0.001	0.7	6.3	95.7	93.1	4
SCZ	CVD	SMR	2000s	5	6	**2.68**	(2.15 - 3.34)	<0.001	1.2	5.8	98.6	357.9	5
SCZ	CVD	SMR	2010s	3	3	**2.53**	(1.32 - 4.84)	0.005	0.0	11000.0	99.4	350.0	2
SCZ	HF	HR/RRatio	1990s	1	1	**3.25**	(2.94 - 3.60)	<0.001	.	.	.	0.0	0
BD	CVA	HR/RRatio	1990s	2	2	**2.01**	(1.81 - 2.22)	<0.001	.	.	0.0	0.5	1
BD	CVA	SMR	1950s-70s	1	1	1.37	(0.89 - 2.10)	0.151	.	.	.	0.0	0
BD	CVA	SMR	1980s	1	1	1.58	(0.82 - 3.03)	0.172	.	.	.	0.0	0
BD	CVA	SMR	1990s	1	1	**1.30**	(1.20 - 1.40)	<0.001	.	.	.	0.0	0
BD	CVA	SMR	2000s	1	3	**1.75**	(1.59 - 1.93)	<0.001	0.9	3.3	0.0	0.5	2
BD	CVA	SMR	2010s	1	1	1.30	(0.94 - 1.81)	0.119	.	.	.	0.0	0
BD	CHD	HR/RRatio	1980s	1	2	**1.63**	(1.52 - 1.74)	<0.001	.	.	0.0	0.6	1
BD	CHD	HR/RRatio	1990s	3	4	**1.93**	(1.59 - 2.35)	<0.001	1.1	3.5	7.7	3.3	3
BD	CHD	HR/RRatio	2000s	1	1	**1.24**	(1.13 - 1.36)	<0.001	.	.	.	0.0	0
BD	CHD	SMR	1950s-70s	1	1	**1.84**	(1.43 - 2.37)	<0.001	.	.	.	0.0	0
BD	CHD	SMR	1980s	1	1	1.00	(0.69 - 1.44)	0.980	.	.	.	0.0	0
BD	CHD	SMR	1990s	1	1	**1.66**	(1.59 - 1.74)	<0.001	.	.	.	0.0	0

SMI	Outcome	Measure	Decade	Studies	Results	Effect	95% CI	p	Lower prediction interval	Upper prediction interval	I^2	Q	Q df
BD	CHD	SMR	2000s	1	3	**1.77**	(1.65 - 1.90)	<0.001	1.1	2.8	0.0	1.2	2
BD	CHD	SMR	2010s	1	1	**1.33**	(1.00 - 1.77)	0.052	.	.	.	0.0	0
BD	CVD	HR/RRatio	1990s	1	1	**2.03**	(1.93 - 2.13)	<0.001	.	.	.	0.0	0
BD	CVD	HR/RRatio	2000s	1	1	1.11	(0.85 - 1.46)	0.450	.	.	.	0.0	0
BD	CVD	OR	2010s	1	2	**1.83**	(1.57 - 2.14)	<0.001	.	.	65.2	2.9	1
BD	CVD	SMR	1950s-70s	6	6	**1.47**	(1.31 - 1.65)	<0.001	1.2	1.8	7.4	5.4	5
BD	CVD	SMR	1990s	2	2	**1.54**	(1.47 - 1.60)	<0.001	.	.	31.7	1.5	1
BD	CVD	SMR	2000s	3	5	**1.86**	(1.75 - 1.99)	<0.001	1.5	2.3	52.1	8.4	4
BD	CVD	SMR	2010s	1	1	**1.33**	(1.11 - 1.60)	0.002	.	.	.	0.0	0
SMI	CHD	SMR	2000s	1	1	**3.70**	(2.47 - 5.55)	<0.001	.	.	.	0.0	0
SMI	CVA	SMR	2000s	1	1	0.80	(0.16 - 4.08)	0.788	.	.	.	0.0	0
SMI	CVD	HR/RRatio	2000s	1	1	**5.41**	(2.40 - 12.20)	<0.001	.	.	.	0.0	0
SMI	CVD	SMR	1980s	2	3	**1.82**	(1.28 - 2.58)	0.001	0.0	105.6	75.1	8.0	2
SMI	CVD	SMR	2000s	1	1	**3.70**	(2.80 - 4.90)	<0.001	.	.	.	0.0	0
SMI	CVD	SMR	2010s	1	1	**2.65**	(2.45 - 2.86)	<0.001	.	.	.	0.0	0

SCZ – schizophrenia, BD – bipolar disorder, SMI – mixed severe mental illness, HR – hazard ratio, RRatio – rate ratio, SMR – standardised mortality ratio, OR – odds ratio, CVA – cerebrovascular accident, CHD – coronary heart disease, CVD – all circulatory disease, HF – heart failure

Results where 95% confidence intervals exclude the null highlighted in ***bold***

Table B: Meta-analysis: pooled results by severe mental illness and cardiovascular incidence

SMI	Outcome	Measure	Decade	Studies	Results	Effect	95% CI					p	Lower prediction interval	Upper prediction interval	I^2	Q	Q df
SCZ	CVA	HR/RRatio	1980s	1	1	1.41	(	0.75	-	2.65	)	0.285	.	.	.	0.0	0
SCZ	CVA	HR/RRatio	1990s	1	1	1.25	(	1.17	-	1.33	)	<0.001	.	.	.	0.0	0
SCZ	CVA	HR/RRatio	2000s	2	3	1.86	(	1.54	-	2.25	)	<0.001	0.2	16.4	73.3	7.5	2
SCZ	CVA	OR	1990s	1	1	2.11	(	1.22	-	3.65	)	0.008	.	.	.	0.0	0
SCZ	CVA	OR	2000s	1	1	1.72	(	1.07	-	2.79	)	0.027	.	.	.	0.0	0
SCZ	CVA	RR	1990s	2	2	1.33	(	1.01	-	1.75	)	0.040	.	.	0.0	0.2	1
SCZ	CVA	RR	2000s	1	1	1.40	(	1.32	-	1.49	)	<0.001	.	.	.	0.0	0
SCZ	CHD	HR/RRatio	1980s	2	3	0.79	(	0.50	-	1.24	)	0.307	0.0	205.5	88.2	17.0	2
SCZ	CHD	HR/RRatio	1990s	1	1	0.88	(	0.83	-	0.94	)	<0.001	.	.	.	0.0	0
SCZ	CHD	HR/RRatio	2000s	5	6	1.46	(	1.22	-	1.74	)	<0.001	0.8	2.7	98.0	243.4	5
SCZ	CHD	OR	1990s	1	1	0.86	(	0.56	-	1.32	)	0.487	.	.	.	0.0	0
SCZ	CHD	OR	2000s	1	1	0.73	(	0.55	-	0.96	)	0.023	.	.	.	0.0	0
SCZ	CHD	RR	1990s	3	3	0.91	(	0.72	-	1.14	)	0.403	0.1	11.5	73.7	7.6	2
SCZ	CHD	SIR	1990s	1	2	1.48	(	1.30	-	1.69	)	<0.001	.	.	85.0	6.7	1
SCZ	CVD	HR/RRatio	1990s	1	1	1.18	(	1.05	-	1.33	)	0.006	.	.	.	0.0	0
SCZ	CVD	HR/RRatio	2000s	2	2	1.29	(	0.96	-	1.72	)	0.092	.	.	84.9	6.6	1
SCZ	CVD	OR	2010s	1	2	1.80	(	1.24	-	2.62	)	0.002	.	.	95.3	21.4	1
SCZ	HF	HR/RRatio	2000s	1	1	3.82	(	3.10	-	4.71	)	<0.001	.	.	.	0.0	0
SCZ	HF	OR	1990s	1	1	2.38	(	1.42	-	4.00	)	0.001	.	.	.	0.0	0
SCZ	HF	OR	2000s	1	1	1.37	(	0.85	-	2.21	)	0.195	.	.	.	0.0	0
SCZ	HF	RR	1990s	2	2	1.72	(	1.08	-	2.75	)	0.023	.	.	75.1	4.0	1
BD	CVA	HR/RRatio	1980s	1	1	1.57	(	0.93	-	2.65	)	0.090	.	.	.	0.0	0
BD	CVA	HR/RRatio	1990s	1	1	1.39	(	1.27	-	1.53	)	<0.001	.	.	.	0.0	0
BD	CVA	HR/RRatio	2000s	2	3	1.72	(	1.43	-	2.06	)	<0.001	0.2	14.2	76.3	8.4	2
BD	CVA	OR	2000s	1	1	3.39	(	1.91	-	6.01	)	<0.001	.	.	.	0.0	0
BD	CVA	RR	1990s	1	1	1.19	(	0.34	-	4.16	)	0.785	.	.	.	0.0	0
BD	CVA	RR	2000s	1	1	1.31	(	1.18	-	1.45	)	<0.001	.	.	.	0.0	0
BD	CHD	HR/RRatio	1980s	1	1	1.39	(	0.75	-	2.59	)	0.299	.	.	.	0.0	0
BD	CHD	HR/RRatio	1990s	1	1	1.04	(	0.94	-	1.15	)	0.425	.	.	.	0.0	0
BD	CHD	HR/RRatio	2000s	3	4	1.62	(	1.17	-	2.24	)	0.004	0.3	7.7	95.8	70.7	3
BD	CHD	OR	1990s	1	1	2.68	(	1.51	-	4.76	)	0.001	.	.	.	0.0	0
BD	CHD	RR	1990s	1	1	0.97	(	0.50	-	1.88	)	0.928	.	.	.	0.0	0
BD	CVD	HR/RRatio	1980s	1	1	1.54	(	1.02	-	2.33	)	0.040	.	.	.	0.0	0
BD	CVD	HR/RRatio	2000s	1	1	1.46	(	1.31	-	1.63	)	<0.001	.	.	.	0.0	0

SMI	Outcome	Measure	Decade	Studies	Results	Effect	95% CI	p	Lower prediction interval	Upper prediction interval	I^2	Q	Q df
BD	CVD	OR	2000s	1	1	2.58	(1.84 - 3.61)	<0.001	.	.	.	0.0	0
BD	CVD	OR	2010s	1	2	1.67	(1.15 - 2.42)	0.007	.	.	96.9	31.8	1
BD	HF	HR/RRatio	2000s	1	1	3.30	(2.27 - 4.79)	<0.001	.	.	.	0.0	0
BD	HF	RR	1990s	1	1	1.73	(0.88 - 3.40)	0.111	.	.	.	0.0	0

SCZ – schizophrenia, BD – bipolar disorder, SMI – mixed severe mental illness, HR – hazard ratio, RRatio – rate ratio, OR – odds ratio, RR – risk ratio, SIR – standardised incidence ratio, CVA – cerebrovascular accident, CHD – coronary heart disease, CVD – major cardiovascular events, HF – heart failure
Results where 95% confidence intervals exclude the null highlighted in ***bold***

Table C: Results of meta-regressions: estimates of increased effect size relative to the reference category by median 5-year calendar period of outcome, exponentiated regression coefficients (95% confidence intervals,) p values

Explanatory variable (if no. of studies/results ≥10)			Schizophrenia			Bipolar disorder
			**CVA**	**CHD**	**CVD**	**CVD**
5-year calendar period	SMR	1950-69 (ref)	1	1	1	1
		1970-74	3.00 (0.85-10.54), 0.079	0.96 (0.25-3.71), 0.951	-	0.85 (0.55-1.33), 0.430
		1975-79	1.53 (0.60-3.92), 0.328	0.90 (0.29-2.81), 0.830	0.75 (0.20-2.85), 0.643	0.91 (0.60-1.38), 0.611
		1980-84	1.71 (0.69-4.24), 0.208	0.63 (0.21-1.90), 0.360	0.34 (0.08-1.42), 0.127	-
		1985-89	-	-	-	-
		1990-94	2.73 (0.91-8.22), 0.069	1.29 (0.38-4.34), 0.638	1.03 (0.27-3.94), 0.964	-
		1995-99	**3.16** (1.18-8.44), 0.027	1.28 (0.35-4.65), 0.667	0.99 (0.24-4.00), 0.983	0.94 (0.65-1.37), 0.718
		2000-04	2.19 (0.97-4.95), 0.057	1.44 (0.51-4.10), 0.440	1.02 (0.28-3.77), 0.969	1.15 (0.80-1.67), 0.395
		2005-09	-	-	1.56 (0.38-6.35), 0.502	1.15 (0.78-1.70), 0.420
		2010-14	1.52 (0.63-3.68), 0.302	1.23 (0.40-3.81), 0.687	1.22 (0.32-4.59), 0.751	0.82 (0.52-1.28), 0.332
	Model p-value, residual I^2, adjusted R^2		*0.210, 81.4%, 45.8%*	*0.394, 97.5%, 12.4%*	*0.244, 98.6%, 24.9%*	*0.067, 50.2%, 78.8%*
	HR/rate ratio	1980-84 (ref)	Insufficient observations	Insufficient observations	1	Insufficient observations
		1985-89			-	
		1990-94			-	
		1995-99			2.00 (0.46-8.59), 0.306	
		2000-04			1.16 (0.27-4.95), 0.823	
		2005-09			1.68 (0.42-6.77), 0.417	
		2010-14			-	
	Model p-value, residual I^2, adjusted R^2				*0.328, 95.6%, 19.3%*	
Random effects meta-analysis I^2		*SMRs*	*91.5%*	*98.0%*	*99.2%*	*80.4%*
		HR/rate ratio			*98.9%*	

CVA – cerebrovascular accident, CHD – coronary heart disease, CVD – all circulatory disease, SMR – standardised mortality ratio, HR – hazard ratio, ref – reference group. Results where 95% confidence intervals exclude the null highlighted in bold. Models adjusted for age and sex and minimum number of additional factors.

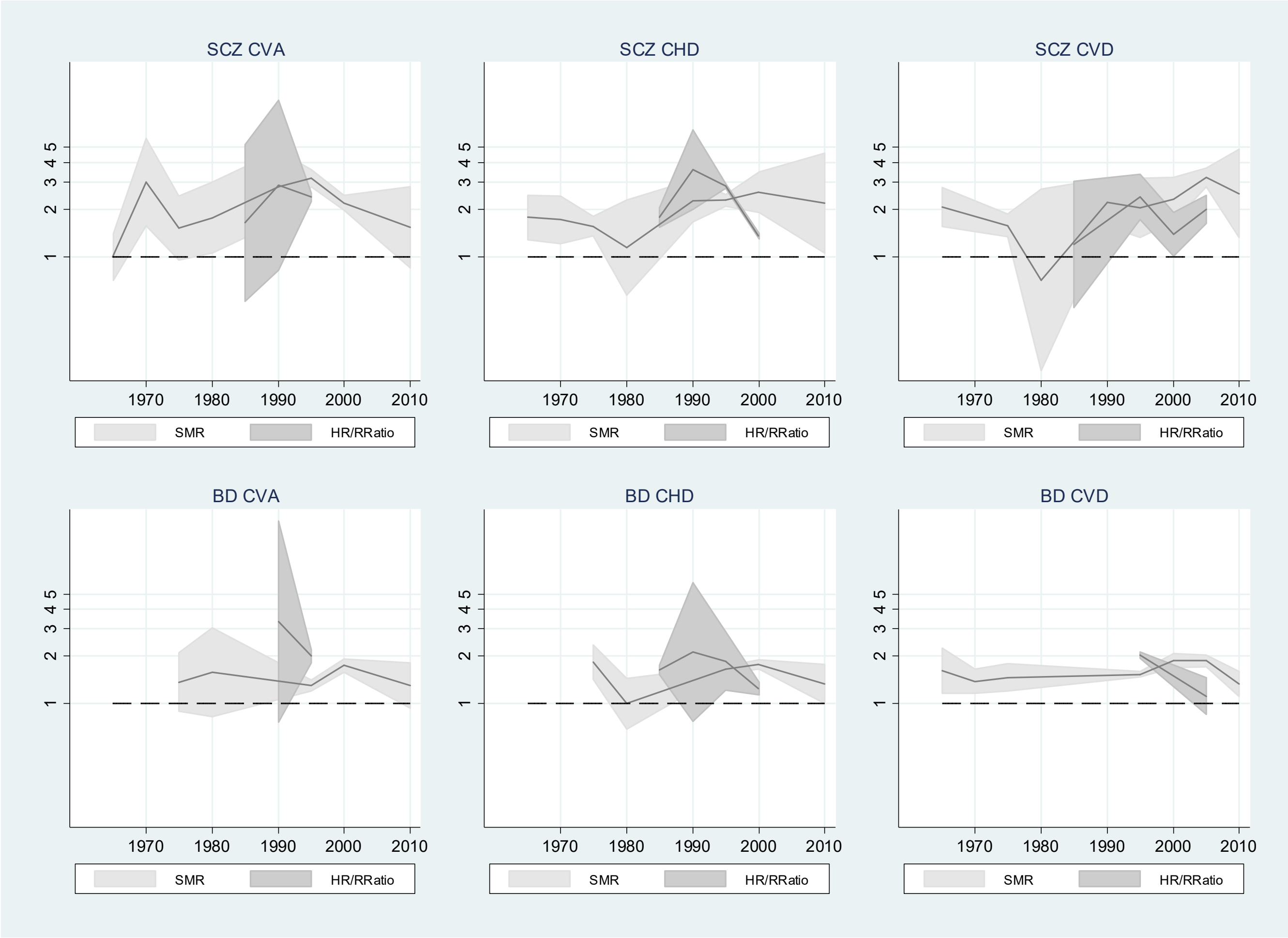

SCZ – schizophrenia, BD – bipolar disorder, CVA – cerebrovascular accident, CHD – coronary heart disease, CVD – all circulatory disease,
SMR – standardised mortality ratio, HR – hazard ratio, RRatio – rate ratio
y-axes show estimate of effect size on log scale, shaded areas show 95% confidence intervals for risk estimates

Fig C: Trend in risk of CVD mortality for severe mental illness compared with controls, by median 5-year calendar period of outcome

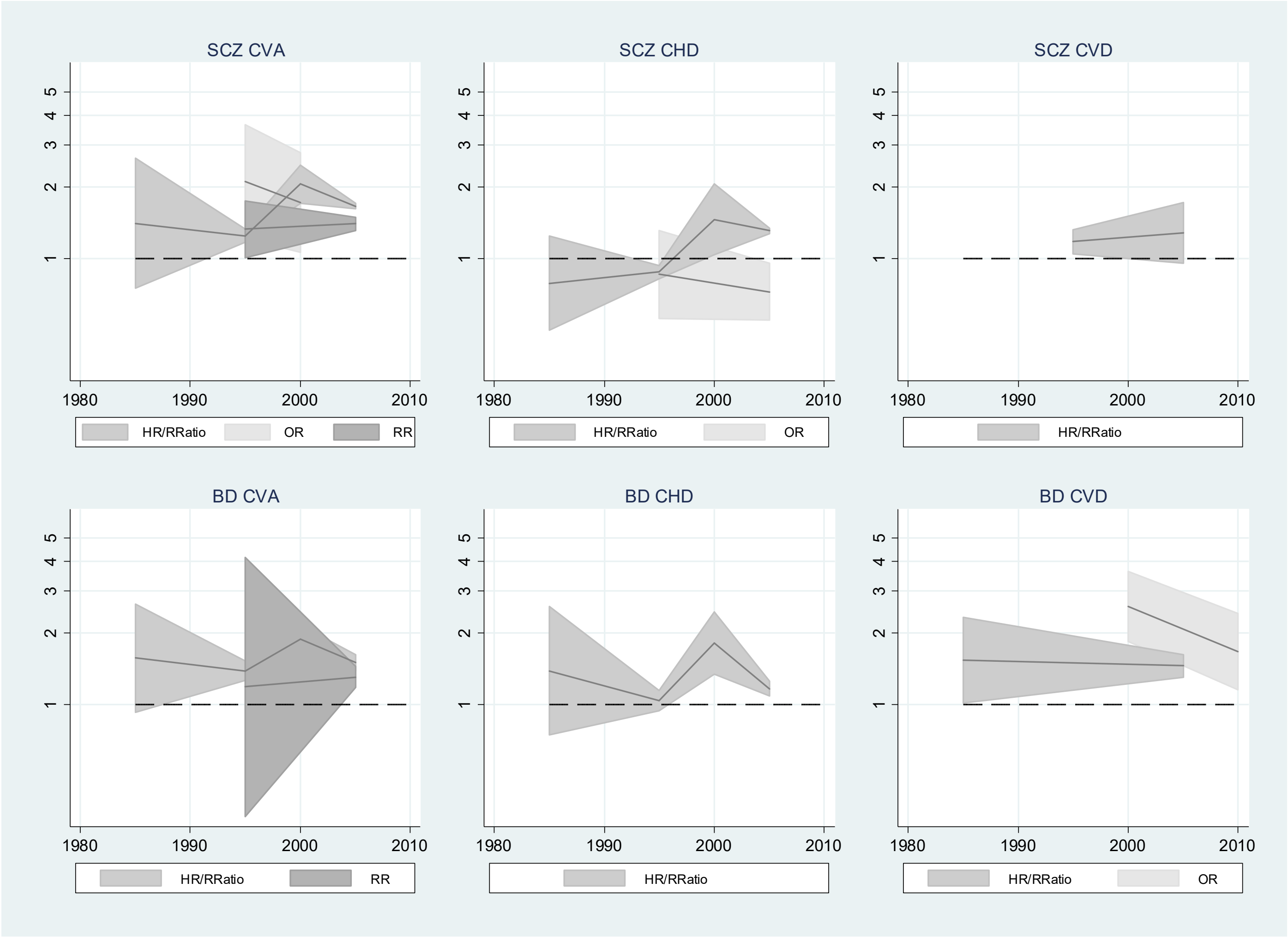

SCZ – schizophrenia, BD – bipolar disorder, CVA – cerebrovascular accident, CHD – coronary heart disease, CVD – major cardiovascular events, HR – hazard ratio, RRatio – rate ratio, OR – odds ratio, RR – risk ratio
y-axes show estimates of effect size on log scale, shaded areas show 95% confidence intervals for risk estimates

Fig D: Trend in risk of CVD incidence for severe mental illness compared with controls, by median 5-year calendar period of outcome
