# Supplementary material for: Temporal trends in associations between severe mental illness and risk of cardiovascular disease: A systematic review and meta-analysis
Source: PLoS Med. 2022 Apr 19;19(4):e1003960. doi: 10.1371/journal.pmed.1003960 (PMC9017899; doi:10.1371/journal.pmed.1003960)
Supplement: S20 File — Fig A: Forest plots showing studies reporting CVD mortality risk for SMI versus controls for (a) age groups ≤60; (b) older age groups or all ages. Fig B: Forest plots showing relative risk of CVD mortality in those with versus without schizophrenia, studies included in meta-analysis for (a) males; (b) females. Fig C: Forest plots showing relative risk of CVD mortality in those with versus without BD, studies included in meta-analysis for (a) males; (b) females. Fig D: Forest plots showing relative risk of CVD mortality in those with versus without mixed SMI, studies included in meta-analysis for (a) males; (b) females. Fig E: Forest plots of CVD incidence for (a) studies with low risk of bias, (b) all studies included in meta-analysis, schizophrenia and BD. BD, bipolar disorder; CVD, cardiovascular disease; SMI, severe mental illness. (PDF) [file pmed.1003960.s020.pdf]

S20 File. Subgroup analysis

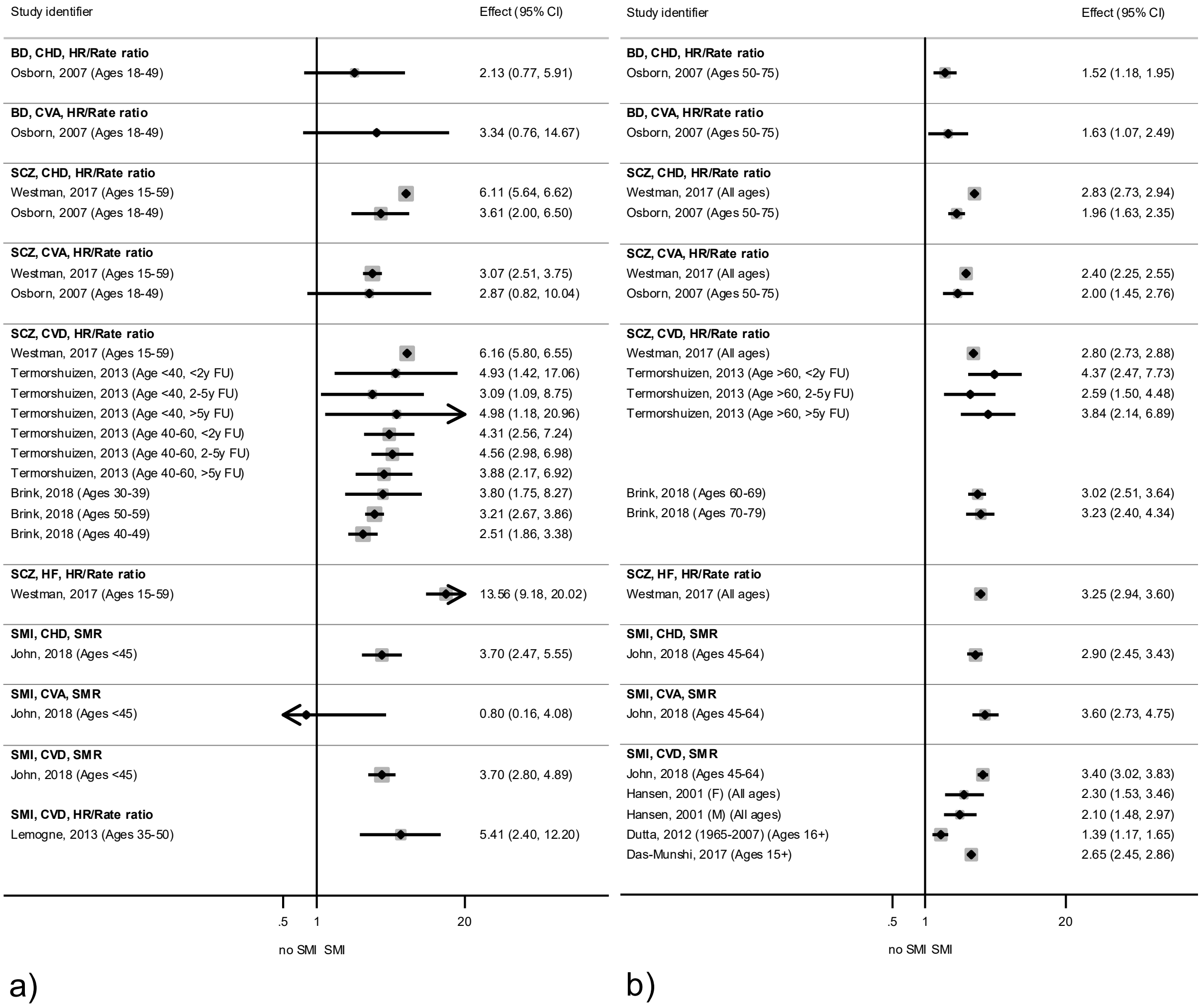

BD – bipolar disorder, SCZ – schizophrenia, SMI – mixed severe mental illness, CHD – coronary heart disease, CVA – cerebrovascular accident, CVD – all circulatory disease, HF – heart failure, HR – hazard ratio, SMR – standardised mortality ratio, FU – follow-up

Fig A: Forest plots showing studies reporting CVD mortality risk for severe mental illness vs controls for a) age groups ≤60; b) older age groups or all ages

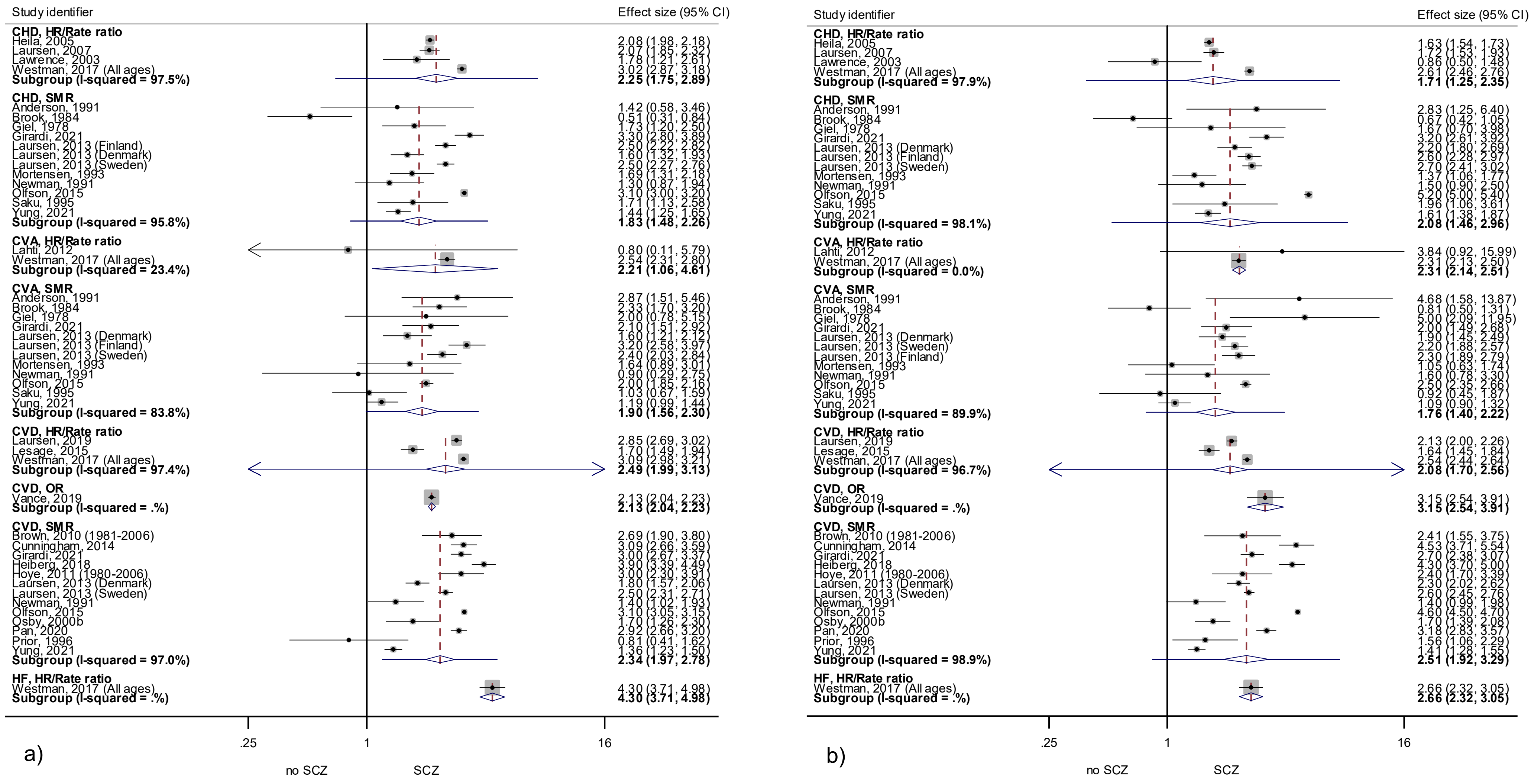

SCZ – schizophrenia, HR – hazard ratio, SMR – standardised mortality ratio, OR – odds ratio, CHD – coronary heart disease, CVA – cerebrovascular accident, CVD – all circulatory disease, HF – heart failure

Fig B: Forest plots showing relative risk of CVD mortality in those with vs without schizophrenia, studies included in meta-analysis for a) males; b) females

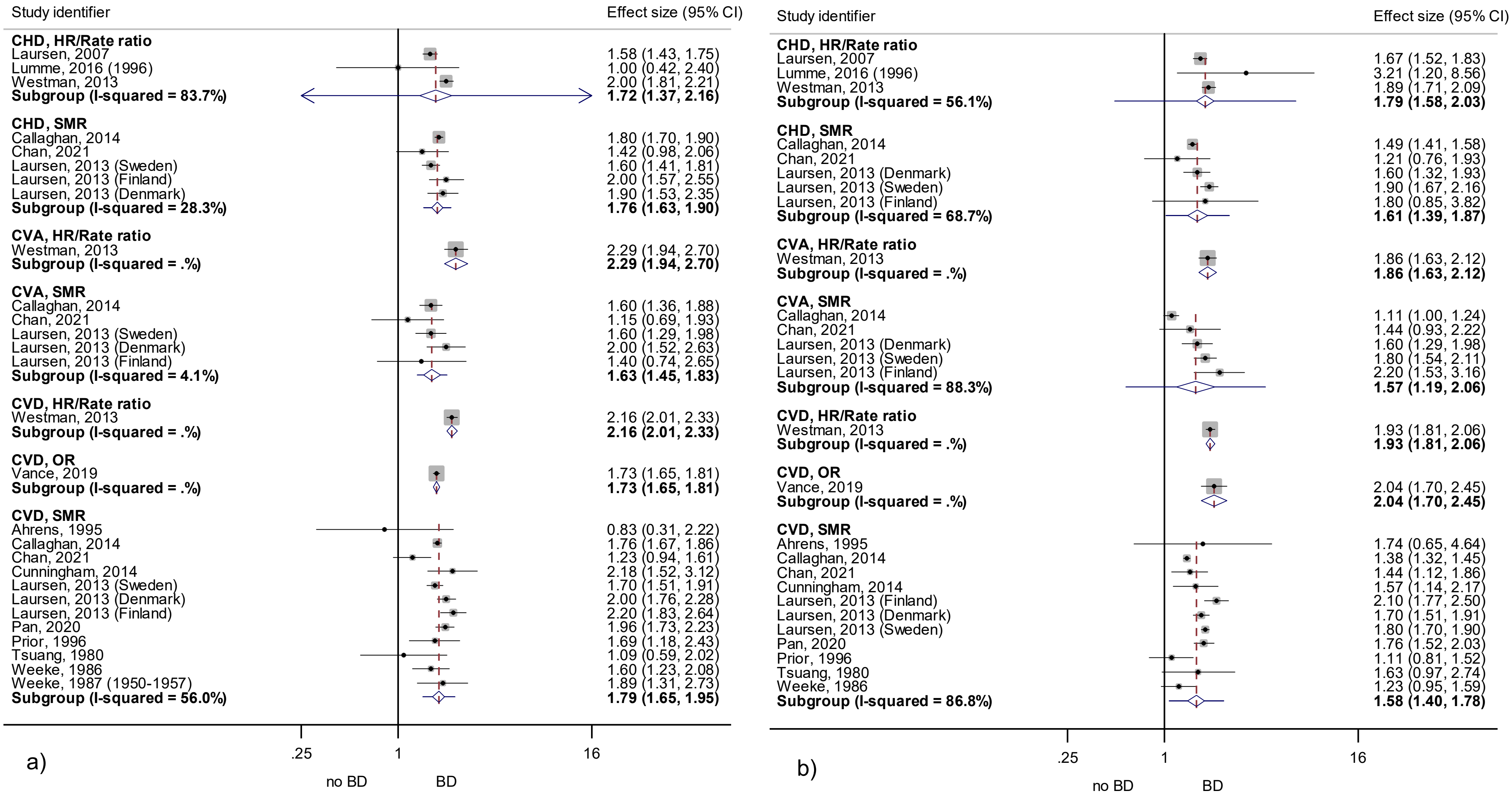

BD – bipolar disorder, HR – hazard ratio, SMR – standardised mortality ratio, OR – odds ratio, CHD – coronary heart disease, CVA – cerebrovascular accident, CVD – all circulatory disease

Fig C: Forest plots showing relative risk of CVD mortality in those with vs without bipolar disorder, studies included in meta-analysis for a) males; b) females

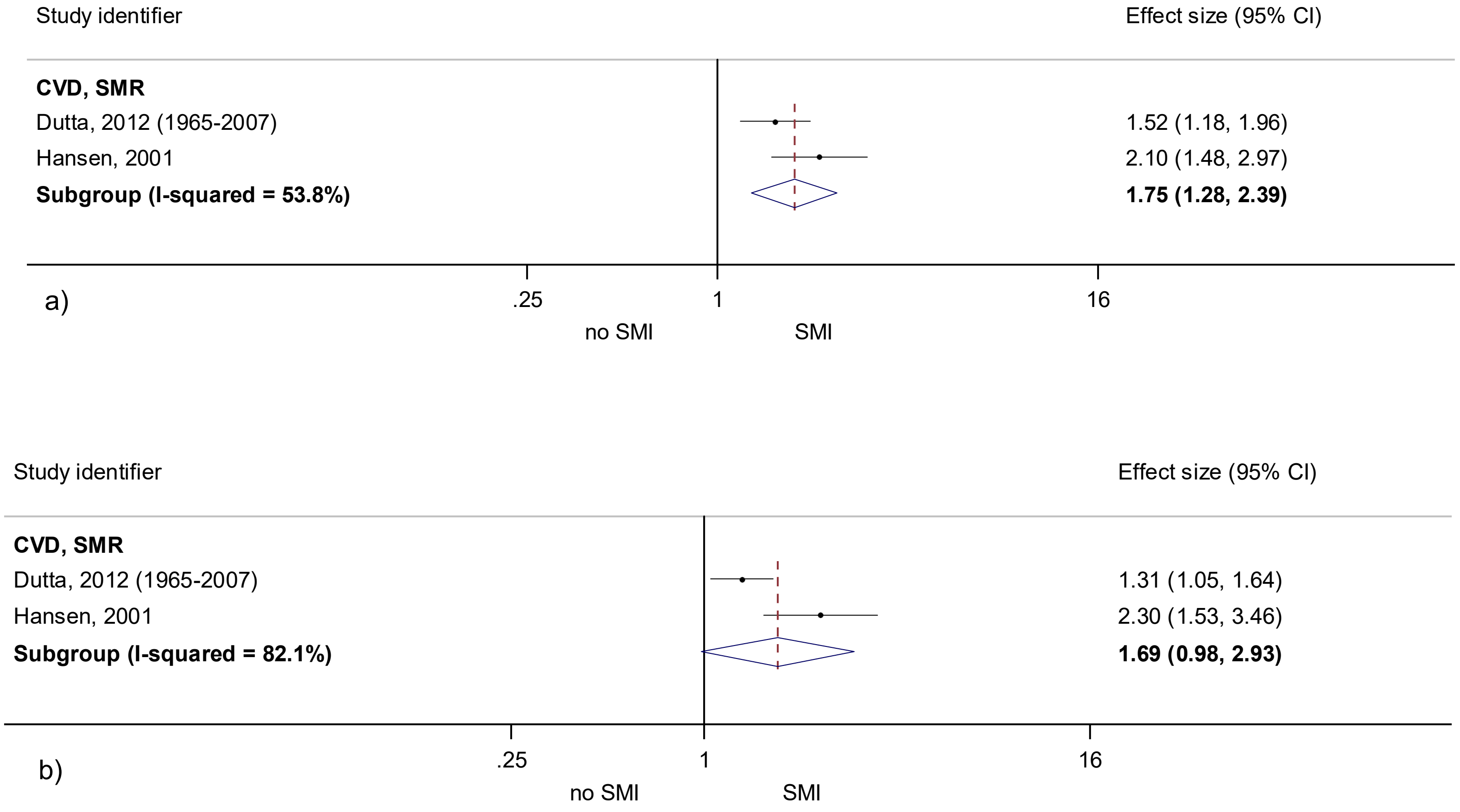

SMI – severe mental illness, SMR – standardised mortality ratio, CVD – all circulatory disease

Fig D: Forest plots showing relative risk of CVD mortality in those with vs without mixed SMI, studies included in meta-analysis for a) males; b) females

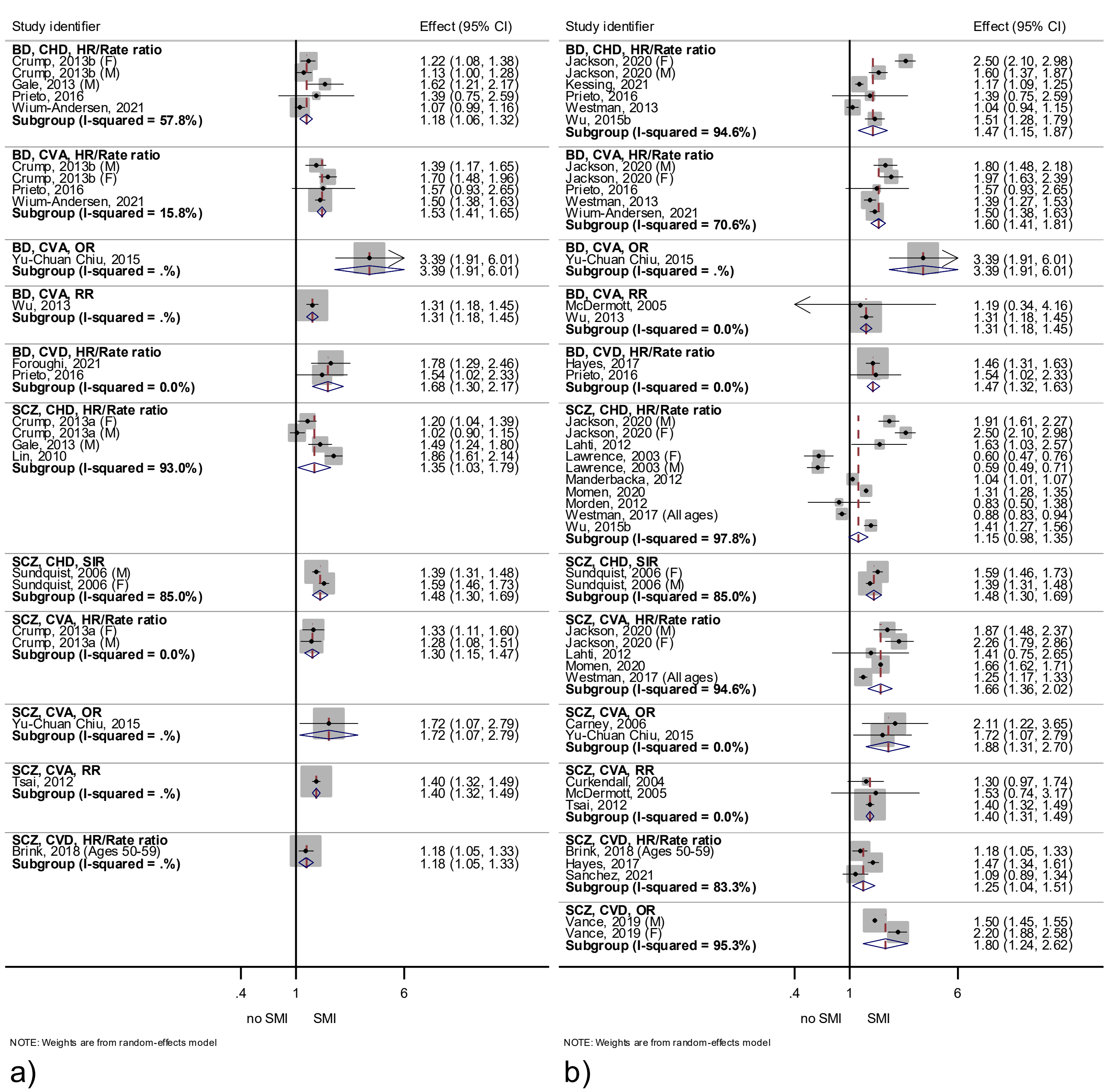

BD – bipolar disorder, SCZ – schizophrenia, CHD – coronary heart disease, CVA – cerebrovascular accident, CVD – major cardiovascular events, HR – hazard ratio, OR – odds ratio, SIR – standardised incidence ratio,

Fig E: Forest plots of CVD incidence for a) studies with low risk of bias, b) all studies included in meta-analysis, schizophrenia and bipolar disorder
