# Supplementary material for: Temporal trends in associations between severe mental illness and risk of cardiovascular disease: A systematic review and meta-analysis
Source: PLoS Med. 2022 Apr 19;19(4):e1003960. doi: 10.1371/journal.pmed.1003960 (PMC9017899; doi:10.1371/journal.pmed.1003960)
Supplement: S21 File — Table A: Sensitivity analyses showing effect of replacing included with excluded results, studies reporting mortality outcomes. Table B: Sensitivity analyses showing effect of replacing included with excluded results, studies reporting incidence outcomes. Table C: Pooled relative risks of cardiovascular incidence by SMI and CVD outcome, comparison of minimally adjusted, and fully adjusted models reported by included studies. CVD, cardiovascular disease; SMI, severe mental illness. (DOCX) [file pmed.1003960.s021.docx]

# S21 File. Sensitivity analysis

**Studies excluded from meta-analysis**

Results of some studies not included in the meta-analysis were outside the prediction intervals of included studies. Studies excluded from the meta-analysis due to overlapping periods of recruitment or multiple results from the same study are considered in sensitivity analyses (see Tables Q1 and Q2).

Seven additional studies (one reporting incidence [1], and six reporting CVD mortality [2-7]) that were excluded from the meta-analysis due to unreported confidence intervals for effect estimates are considered here. For schizophrenia, there were six reported rate ratios that were within the prediction intervals of studies included in the meta-analysis for both CVA and CHD, also nine rate ratios and 14 SMRs within the prediction intervals for CVD. For bipolar disorder, there were six rate ratios for CVA with unreported confidence intervals with effect sizes between 1.38 and 2.65. No prediction interval was able to be calculated as there were only two studies included in this meta-analysis. However, the effect sizes for the excluded studies extend beyond both the upper and lower confidence interval of the pooled estimate. Similarly, no prediction interval was calculated for the incidence of major CVD events but the effect size of one study [1] was above the range of the pooled confidence interval. There was one SMR with unreported confidence intervals for CVA and six rate ratios for CHD that fell within the prediction intervals of the respective meta-analyses. SMRs for one study [4] fell below the prediction interval of the pooled estimate for females and above for males. For all circulatory disease mortality, there were six rate ratios with unreported confidence intervals: all were within the confidence interval of the pooled estimate. Of the eight SMRs with unreported confidence intervals for all CVD, one [2] was above the prediction interval. One study [7] reported CVA and CHD rate ratios for mixed SMI that were within the confidence intervals for SMRs of the respective outcomes.

**Borderline studies**

The decision to exclude studies at full-text screening was, in some cases, borderline due to lack of clarity in the methodology [8-10], definition of SMI [11], outcome [12] or a high proportion with CVD at baseline [13,14]. However, results were similar to those for included studies, *i.e* higher relative risks of CVD mortality for people with SMI.

Table A: Sensitivity analyses showing effect of replacing included with excluded results, studies reporting mortality outcomes

| **SMI** | **Mortality  outcome** | **Effect type** | **Change** | **Meta-analysis pooled effect (95% CI)** | **New pooled effect (95% CI)** | **Comment** |
| --- | --- | --- | --- | --- | --- | --- |
| SCZ | CVA | HR/RRatio | Replace Westman with Crump | **2.40** (2.25-2.55) | **2.37** (1.82-3.08) | Slightly smaller effect size, but wider CI |
| SCZ | CVA | HR/RRatio | Replace Osborn ages 18-49 with ages 50-75 | **2.40** (2.25-2.55) | **2.38** (2.24-2.53) | No difference in scale or direction of effect |
| SCZ | CVA | HR/RRatio | Replace Westman all ages with ages 15-59 | **2.40** (2.25-2.55) | **3.01** (2.48-3.66) | Larger effect size, but wider CI |
| SCZ | CVA | SMR | Replace Mortensen, 1993 with Mortensen, 1990 | **1.93** (1.63-2.28) | **1.86** (1.44-2.40) | Slightly smaller effect size, but wider CI |
| SCZ | CVA | SMR | Replace Laursen (Denmark) with Castagnini | **1.93** (1.63-2.28) | **1.94** (1.62-2.31) | No difference in scale or direction of effect |
| SCZ | CVA | SMR | Replace Allebeck with Ösby, 2000 | **1.93** (1.63-2.28) | **1.91** (1.62-2.25) | No difference in scale or direction of effect |
| SCZ | CVA | SMR | Retain Callaghan as well as Olfson | **1.93** (1.63-2.28) | **1.95** (1.71-2.21) | No difference in scale or direction of effect |
| SCZ | CHD | HR/RRatio | Replace Laursen, 2007 with Laursen, 2010 (1994-96) | **1.79** (1.43-2.24) | **1.79** (1.40-2.28) | No difference in scale or direction of effect |
| SCZ | CHD | HR/RRatio | Replace Laursen, 2007 with Laursen, 2010 (2005-2006) | **1.79** (1.43-2.24) | **1.92** (1.49-2.47) | Slightly larger effect size, but wider CI |
| SCZ | CHD | HR/RRatio | Replace Laursen, 2007 with Laursen, 2014 | **1.79** (1.43-2.24) | **1.84** (1.43-2.37) | No difference in scale or direction of effect |
| SCZ | CHD | HR/RRatio | Replace Heila and Manderbacka with Lahti | **1.79** (1.43-2.24) | **1.95** (1.47-2.63) | Slightly larger effect size, but wider CI |
| SCZ | CHD | HR/RRatio | Replace Heila with Lumme (1990-96) | **1.79** (1.43-2.24) | **1.88** (1.41-2.49) | Slightly larger effect size, but wider CI |
| SCZ | CHD | HR/RRatio | Replace Helia with last year of Lumme | **1.79** (1.43-2.24) | **2.17** (1.62-2.91) | Slightly larger effect size, but wider CI |
| SCZ | CHD | HR/RRatio | Replace Westman with Crump | **1.79** (1.43-2.24) | **1.92** (1.63-2.27) | Slightly larger effect size and narrower CI |
| SCZ | CHD | HR/RRatio | Replace Westman with Bjorkenstam | **1.79** (1.43-2.24) | **1.98** (1.67-2.35) | Slightly larger effect size |
| SCZ | CHD | HR/RRatio | Replace Osborn ages 18-49 with ages 50-75 | **1.79** (1.43-2.24) | **1.74** (1.40-2.16) | No difference in scale or direction of effect |
| SCZ | CHD | HR/RRatio | Replace Osborn with Murray-Thomas | **1.79** (1.43-2.24) | **1.73** (1.38-2.16) | No difference in scale or direction of effect |
| SCZ | CHD | HR/RRatio | Replace Kilbourne with Daumit | **1.79** (1.43-2.24) | **1.85** (1.51-2.28) | No difference in scale or direction of effect |
| SCZ | CHD | HR/RRatio | Replace Westman all ages with ages 15-59 | **1.79** (1.43-2.24) | **1.95** (1.44-2.63) | Slightly larger effect size, but wider CI |
| SCZ | CHD | HR/RRatio | Replace Heila follow-up >10 years with 0-5 years | **1.79** (1.43-2.24) | **1.90** (1.45-2.48) | Slightly larger effect size, but wider CI |
| SCZ | CHD | SMR | Replace Mortensen, 1993 with Mortensen, 1990 | **1.94** (1.58-2.37) | **1.88** (1.42-2.51) | No difference in scale or direction of effect, but wider CI |
| SCZ | CHD | SMR | Replace Laursen (Denmark) with Castagnini | **1.94** (1.58-2.37) | **2.03** (1.66-2.49) | Slightly larger effect size |
| SCZ | CHD | SMR | Replace Allebeck with Ösby, 2000 | **1.94** (1.58-2.37) | **1.97** (1.62-2.40) | No difference in scale or direction of effect |
| SCZ | CHD | SMR | Replace Olfson with Callaghan and Kelly (Clozapine) | **1.94** (1.58-2.37) | **2.01** (1.75-2.31) | Slightly larger effect size and narrower CI |
| SCZ | CHD | SMR | Replace Olfson with Callaghan and Kelly (Risperidone) | **1.94** (1.58-2.37) | **1.94** (1.69-2.22) | No difference in scale or direction of effect, but narrower CI |
| SCZ | CVD | HR/RRatio | Replace Westman with Crump and Fors | **1.91** (1.52-2.41) | **2.01** (1.63-2.49) | Slightly larger effect size and narrower CI |
| SCZ | CVD | HR/RRatio | Replace Westman with Crump, Fors and Tornainen (moderate level of antipsychotics) | **1.91** (1.52-2.41) | **2.17** (1.73-2.72) | Larger effect size |
| SCZ | CVD | HR/RRatio | Replace Westman with Crump, Fors and Tornainen (no antipsychotics) | **1.91** (1.52-2.41) | **2.20** (1.77-2.74) | Larger effect size |
| SCZ | CVD | HR/RRatio | Replace Westman with Crump, Fors and Tornainen (low level of antipsychotics) | **1.91** (1.52-2.41) | **2.13** (1.73-2.64) | Larger effect size |
| SCZ | CVD | HR/RRatio | Replace Westman with Crump, Fors and Tornainen (moderate level of antipsychotics) | **1.91** (1.52-2.41) | **2.23** (1.74-2.86) | Larger effect size |
| SCZ | CVD | HR/RRatio | Replace Westman all ages with ages 15-59 | **1.91** (1.52-2.41) | **2.05** (1.43-2.93) | Slightly larger effect size, but wider CI |
| SCZ | CVD | HR/RRatio | Replace Laursen, 2019 with Brink ages 50-59 | **1.91** (1.52-2.41) | **1.91** (1.42-2.57) | No difference in scale or direction of effect, but wider CI |
| SCZ | CVD | HR/RRatio | Replace Brink ages 50-59 with ages 40-49 | **1.91** (1.52-2.41) | **1.87** (1.39-2.51) | No difference in scale or direction of effect, but wider CI |
| SCZ | CVD | HR/RRatio | Replace Brink ages 50-59 with ages 30-39 | **1.91** (1.52-2.41) | **1.90** (1.40-2.57) | No difference in scale or direction of effect, but wider CI |
| SCZ | CVD | HR/RRatio | Replace Brink ages 50-59 with ages 60-69 | **1.91** (1.52-2.41) | **1.90** (1.42-2.55) | No difference in scale or direction of effect, but wider CI |
| SCZ | CVD | HR/RRatio | Replace Brink ages 50-59 with ages 70-79 | **1.91** (1.52-2.41) | **1.91** (1.42-2.57) | No difference in scale or direction of effect, but wider CI |
| SCZ | CVD | HR/RRatio | Replace Termorshuizen with min effect size for age/follow-up category | **1.91** (1.52-2.41) | **1.85** (1.47-2.33) | No difference in scale or direction of effect |
| SCZ | CVD | HR/RRatio | Replace Termorshuizen with max effect size for age/follow-up category | **1.91** (1.52-2.41) | **1.85** (1.46-2.34) | No difference in scale or direction of effect |
| SCZ | CVD | SMR | Replace Laursen (Denmark with Hiroeh) | **2.02** (1.69-2.41) | **2.00** (1.67-2.40) | No difference in scale or direction of effect |
| SCZ | CVD | SMR | Replace Tanskanen with Kiviniemi | **2.02** (1.69-2.41) | **2.05** (1.71-2.46) | No difference in scale or direction of effect |
| SCZ | CVD | SMR | Replace Tanskanen with Laursen 2013 (Finland) | **2.02** (1.69-2.41) | **2.03** (1.70-2.42) | No difference in scale or direction of effect |
| SCZ | CVD | SMR | Replace Laursen, 2013 (Sweden) with Ösby, 2000 (1991-95) | **2.02** (1.69-2.41) | **2.19** (1.80-2.66) | Slightly larger effect size, but wider CI |
| SCZ | CVD | SMR | Replace Buda with Tsuang | **2.02** (1.69-2.41) | **1.98** (1.65-2.37) | No difference in scale or direction of effect |
| SCZ | CVD | SMR | Retain Callaghan as well as Olfson | **2.02** (1.69-2.41) | **2.06** (1.79-2.38) | No difference in scale or direction of effect |
| SCZ | CVD | SMR | Replace Tanskanen data for 2014 with 1984 | **2.02** (1.69-2.41) | **2.00** (1.67-2.39) | No difference in scale or direction of effect |
| SCZ | CVD | SMR | Replace Høye data for 1980-1992 with 1993-2006 | **2.02** (1.69-2.41) | **2.03** (1.70-2.43) | No difference in scale or direction of effect |
| BD | CVA | HR/RRatio | Replace Osborn ages 18-49 with ages 50-75 | **2.00** (1.81-2.22) | **1.98** (1.79-2.18) | No difference in scale or direction of effect |
| BD | CVA | HR/RRatio | Replace Westman with Crump | **2.00** (1.81-2.22) | **2.35** (1.78-3.09) | Larger effect size, but wider CI |
| BD | CVA | SMR | Replace Laursen (Sweden) and Nilsson with Ösby | **1.55** (1.33-1.81) | **1.59** (1.30-1.95) | No difference in scale or direction of effect |
| BD | CVA | SMR | Retain Ösby as well as Laursen (Sweden) | **1.55** (1.33-1.81) | **1.61** (1.38-1.89) | No difference in scale or direction of effect |
| BD | CVA | SMR | Replace Laursen with Castagnini | **1.55** (1.33-1.81) | **1.59** (1.32-1.92) | No difference in scale or direction of effect |
| BD | CHD | HR/RRatio | Replace Laursen, 2007 with Laursen, 2010 (1994-96) | **1.61** (1.34-1.94) | **1.69** (1.28-2.23) | No difference in scale or direction of effect |
| BD | CHD | HR/RRatio | Replace Laursen, 2007 with Laursen, 2010 (2005-2006) | **1.61** (1.34-1.94) | **1.73** (1.29-2.33) | Slightly larger effect size, but wider CI |
| BD | CHD | HR/RRatio | Replace Laursen, 2007 with Laursen, 2014 | **1.61** (1.34-1.94) | **1.84** (1.32-2.56) | Slightly larger effect size, but wider CI |
| BD | CHD | HR/RRatio | Replace first year of Lumme with last year | **1.61** (1.34-1.94) | **1.63** (1.37-1.94) | No difference in scale or direction of effect |
| BD | CHD | HR/RRatio | Replace Westman with Crump | **1.61** (1.34-1.94) | **1.72** (1.44-2.07) | Slightly larger effect size |
| BD | CHD | HR/RRatio | Replace Osborn ages 18-49 with ages 50-75 | **1.61** (1.34-1.94) | **1.59** (1.34-1.88) | No difference in scale or direction of effect |
| BD | CHD | HR/RRatio | Replace Kilbourne with Daumit | **1.61** (1.34-1.94) | **1.96** (1.42-2.72) | Larger effect size, but wider CI |
| BD | CHD | SMR | Replace Laursen with Castagnini | **1.67** (1.54-1.82) | **1.67** (1.51-1.85) | No difference in scale or direction of effect |
| BD | CHD | SMR | Replace Laursen (Sweden) and Nilsson with Ösby | **1.67** (1.54-1.82) | **1.80** (1.54-2.11) | Slightly larger effect size, but wider CI |
| BD | CHD | SMR | Retain Ösby as well as Laursen (Sweden) | **1.67** (1.54-1.82) | **1.80** (1.58-2.04) | Slightly larger effect size, but wider CI |
| BD | CVD | HR/RRatio | Replace Westman with Crump | 1.52 (0.84-2.75) | **1.78** (1.28-2.47) | Larger effect size, achieved statistical significance |
| BD | CVD | SMR | Replace Ahrens with Brodersen | **1.68** (1.58-1.79) | **1.68** (1.58-1.79) | No difference in scale or direction of effect |

SMI – severe mental illness, SCZ – schizophrenia, BD – bipolar disorder, HR – hazard ratio, RRatio – rate ratio, SMR – standardised mortality ratio, CI – confidence interval, CVA – cerebrovascular accident, CHD – coronary heart disease, CVD – all circulatory disease

Results where 95% confidence intervals exclude the null highlighted in **bold**

Table B: Sensitivity analyses showing effect of replacing included with excluded results, studies reporting incidence outcomes

| **SMI** | **Incidence  outcome** | **Effect type** | **Change** | **Meta-analysis pooled effect (95% CI)** | **New pooled effect (95% CI)** | **Comment** |
| --- | --- | --- | --- | --- | --- | --- |
| SCZ | CVA | HR/RRatio | Replace Momen with Laursen, 2011 and Munk-Jorgensen | **1.65** (1.36-2.02) | **1.48** (1.14-1.91) | Slightly smaller effect, but wider CI |
| SCZ | CVA | HR/RRatio | Replace Westman with Crump | **1.65** (1.36-2.02) | **1.61** (1.38-1.87) | No difference in scale or direction of effect |
| SCZ | CVA | RR | Replace Tsai with Chen | **1.40** (1.31-1.49) | **1.63** (1.17-2.27) | Slightly larger effect, but wider CI |
| SCZ | CHD | HR/RRatio | Replace Momen with Munk-Jorgensen and Laursen, 2011 | 1.15 (0.99-1.35) | 1.11 (0.93-1.33) | Slightly smaller effect, CI further from null |
| SCZ | CHD | HR/RRatio | Replace Momen with Jakobsen | 1.15 (0.99-1.35) | 1.09 (0.89-1.32) | Slightly smaller effect, CI further from null |
| SCZ | CHD | HR/RRatio | Replace Laursen, 2011, with Laursen, 2010 (1994-96) | 1.15 (0.99-1.35) | 1.06 (0.89-1.26) | Slightly smaller effect, CI further from null |
| SCZ | CHD | HR/RRatio | Replace Laursen, 2011, with Laursen, 2010 (2005-2006) | 1.15 (0.99-1.35) | 1.10 (0.93-1.31) | Slightly smaller effect, CI further from null |
| SCZ | CHD | HR/RRatio | Replace Westman by Crump and Gale | 1.15 (0.99-1.35) | **1.20** (1.05-1.38) | Increased effect size, CI now excludes the null |
| SCZ | CHD | HR/RRatio | Replace Wu, 2015 with Lin, 2010 | 1.15 (0.99-1.35) | **1.19** (1.01-1.40) | Increased effect size, CI now excludes the null |
| SCZ | CHD | RR | Replace Curkendall IHD outcome with AMI | 0.91 (0.72-1.14) | 0.83 (0.65-1.06) | No difference in scale or direction of effect |
| SCZ | CVD | HR/RRatio | Replace Brink ages 50-59 with ages 40-49 | **1.25** (1.04-1.51) | 1.22 (0.96-1.54) | Reduced effect size, CI now includes the null |
| SCZ | CVD | HR/RRatio | Replace with ages 30-39 | **1.25** (1.04-1.51) | **1.38** (1.03-1.61) | Slightly larger effect, but wider CI |
| BD | CVA | HR/RRatio | Replace Westman with Crump | **1.60** (1.41-1.81) | **1.63** (1.47-1.81) | No difference in scale or direction of effect |
| BD | CVA | HR/RRatio | Replace Wium-Andersen with Laursen, 2011 | **1.60** (1.41-1.81) | **1.74** (1.43-2.10) | Slightly larger effect, but wider CI |
| BD | CHD | HR/RRatio | Replace Kessing with Wium-Andersen | **1.47** (1.16-1.87) | **1.45** (1.11-1.88) | No difference in scale or direction of effect |
| BD | CHD | HR/RRatio | Replace Kessing with Laursen, 2011 | **1.47** (1.16-1.87) | **1.52** (1.01-2.06) | Slightly larger effect, but wider CI |
| BD | CHD | HR/RRatio | Replace Kessing, with Laursen, 2010 (1994-96) | **1.47** (1.16-1.87) | **1.45** (1.10-1.92) | No difference in scale or direction of effect |
| BD | CHD | HR/RRatio | Replace Kessing, with Laursen, 2010 (2005-2006) | **1.47** (1.16-1.87) | **1.50** (1.15-1.96) | No difference in scale or direction of effect |
| BD | CHD | HR/RRatio | Replace Westman by Crump and Gale | **1.47** (1.16-1.87) | **1.46** (1.22-1.75) | No difference in scale or direction of effect |
| BD | CHD | HR/RRatio | Replace Wu, 2015 with Hsu (all ages) | **1.47** (1.16-1.87) | **1.49** (1.16-1.90) | No difference in scale or direction of effect |
| BD | CHD | HR/RRatio | Replace Wu, 2015 with Hsu (ages 40-59) | **1.47** (1.16-1.87) | **1.53** (1.18-1.98) | Slightly larger effect, but wider CI |
| BD | CHD | HR/RRatio | Replace Wu, 2015 with Hsu (ages 18-39) | **1.47** (1.16-1.87) | **1.60** (1.21-2.10) | Slightly larger effect, but wider CI |
| BD | CVD | HR/RRatio | Replace Prieto with Foroughi, 2021 | **1.47** (1.32-1.63) | **1.52** (1.30-1.77) | No difference in scale or direction of effect |
| BD | CVD | HR/RRatio | Replace Prieto with Foroughi, 2018 | **1.47** (1.32-1.63) | **1.45** (1.30-1.60) | No difference in scale or direction of effect |
| BD | CVD | OR | Replace Goldstein BD I with BD II | **1.88** (1.34-2.66) | **1.85** (1.31-2.62) | No difference in scale or direction of effect |

SMI – severe mental illness, SCZ – schizophrenia, BD – bipolar disorder, HR – hazard ratio, RRatio – rate ratio, OR - odds ratio, CI – confidence interval,

CVA – cerebrovascular accident, CHD – coronary heart disease, CVD – major cardiovascular events

Results where 95% confidence intervals exclude the null highlighted in **bold**

Table C: Pooled relative risks of cardiovascular incidence by SMI and CVD outcome, comparison of minimally adjusted and fully adjusted models reported by included studies

| **SMI** | **Incidence outcome** | **Models adjusted for age and sex only, or min number of factors** | | | **Models adjusted for more factors** | | |
| --- | --- | --- | --- | --- | --- | --- | --- |
|  |  | **No. of studies/results** | **Pooled effect (95% CI)** | **p** | **No. of studies/results** | **Pooled effect (95% CI)** | **p** |
| Schizophrenia | CVA | 4/5  2/2  3/3 | HR/Rate ratio: **1.66** (1.36-2.02)  OR: **1.88** (1.31-2.70)  RR: **1.40** (1.31-1.49) | <0.001  0.001  <0.001 | 5/6  2/2  3/3 | HR/Rate ratio: **1.46** (1.27-1.66)  OR: **1.70** (1.15-2.52)  RR: **1.40** (1.31-1.49) | <0.001  0.008  <0.001 |
| Schizophrenia | CHD | 8/10  2/2  3/3  1/2 | HR/Rate ratio: 1.15 (0.99-1.35)  OR: **0.76** (0.61-0.96)  RR: 0.91 (0.72-1.14)  SIR: **1.48** (1.30-1.69) | 0.077  0.022  0.403  <0.001 | 8/10  2/2  3/3  1/2 | HR/Rate ratio: 1.11 (0.97-1.27)  OR: **0.76** (0.61-0.96)  RR: 0.90 (0.71-1.14)  SIR: **1.15** (1.07-1.26) | 0.117  0.022  0.379  0.001 |
| Schizophrenia | CVD | 3/3  1/2 | HR/Rate ratio: **1.25** (1.04-1.51)  OR: **1.80** (1.24-2.62) | 0.016  0.002 | 3/3  1/2 | HR/Rate ratio: **1.18** (1.09-1.27)  OR: **1.52** (1.15-2.01) | <0.001  0.003 |
| Bipolar disorder | CVA | 4/5  1/1  2/2 | HR/Rate Ratio: **1.60** (1.41-1.81)  OR: **3.39** (1.91-6.01)  RR: **1.31** (1.18-1.45) | <0.001  <0.001  <0.001 | 4/5  1/1  1/1 | HR/Rate Ratio: **1.51** (1.33-1.71)  OR: **2.23** (1.06-4.68)  RR: 1.19 (0.34-4.16) | <0.001  0.034  0.785 |
| Bipolar disorder | CHD | 5/6  1/1  1/1 | HR/Rate ratio: **1.47** (1.16-1.87)  OR: **2.68** (1.51-4.76)  RR: 0.97 (0.50-1.88) | 0.002  0.001  0.928 | 5/6  1/1  1/1 | HR/Rate ratio: **1.46** (1.14-1.87)  OR: **2.50** (1.32-4.73)  RR: 0.97 (0.50-1.88) | 0.003  0.005  0.928 |
| Bipolar disorder | CVD | 2/2  2/3 | HR/Rate ratio: **1.47** (1.32-1.63)  OR: **1.88** (1.34-2.66) | <0.001  <0.001 | 2/2  2/3 | HR/Rate ratio: **1.26** (1.12-1.40)  OR: **1.65** (1.20-2.25) | <0.001  0.002 |

SMI – severe mental illness, CVA – cerebrovascular accident, CHD – coronary heart disease, CVD – all circulatory disease, HR – hazard ratio, OR – odds ratio,

RR – risk ratio, SIR – standardised incidence ratio

Results where 95% confidence intervals exclude the null highlighted in **bold**

## References

1. Bent-Ennakhil N, Cecile Perier M, Sobocki P, Gothefors D, Johansson G, Milea D, et al. Incidence of cardiovascular diseases and type-2-diabetes mellitus in patients with psychiatric disorders. Nordic Journal of Psychiatry. 2018;72(7):455-61. doi: <http://dx.doi.org/10.1080/08039488.2018.1463392>.

2. Hoang U, Stewart R, Goldacre MJ. Mortality after hospital discharge for people with schizophrenia or bipolar disorder: retrospective study of linked English hospital episode statistics, 1999-2006. BMJ. 2011;343:d5422. PubMed PMID: 21914766; PubMed Central PMCID: PMCPMC3172324.

3. Nordentoft M, Wahlbeck K, Hallgren J, Westman J, Osby U, Alinaghizadeh H, et al. Excess mortality, causes of death and life expectancy in 270,770 patients with recent onset of mental disorders in Denmark, Finland and Sweden. PLoS One. 2013;8(1):e55176. Epub 2013/02/02. doi: 10.1371/journal.pone.0055176. PubMed PMID: 23372832; PubMed Central PMCID: PMCPMC3555866.

4. Saku M, Tokudome S, Ikeda M, Kono S, Makimoto K, Uchimura H, et al. Mortality in psychiatric patients, with a specific focus on cancer mortality associated with schizophrenia. International Journal of Epidemiology. 1995;24(2):366-72. PubMed PMID: 7635598.

5. Ösby U, Westman J, Hällgren J, Gissler M. Mortality trends in cardiovascular causes in schizophrenia, bipolar and unipolar mood disorder in Sweden 1987-2010. European journal of public health. 2016;26(5):867-71. Epub 2016/01/10. doi: 10.1093/eurpub/ckv245. PubMed PMID: 26748100; PubMed Central PMCID: PMCPMC5054269.

6. Odegard O. Mortality in Norwegian psychiatric hospitals 1950-1962. Acta genetica et statistica medica. 1967;17(1):137-53.

7. Dalgard OS. [Mortality in functional psychosis]. Nordisk medicin. 1966;75(24):680-4. Epub 1966/06/16. PubMed PMID: 5943275.

8. Bresee L, Tonelli M, Manns B, Hemmelgarn B. Temporal trends in incidence of acute myocardial infarction and revascularization in people with and without mental illness. Circulation. 2012;125(10 SUPPL. 1).

9. Wood JB, Evenson RC, Cho DW, Hagan BJ. MORTALITY VARIATIONS AMONG PUBLIC MENTAL-HEALTH PATIENTS. Acta psychiatrica Scandinavica. 1985;72(3):218-29. doi: 10.1111/j.1600-0447.1985.tb02598.x. PubMed PMID: WOS:A1985ATY8700002.

10. Schwalb H, Schomann C, Bruninghaus H, Eckmann F, Jungling D, Reinhold H, et al. [Mortality in hospitalized psychiatric patients (results from a 5-year study) (author's transl)]. Mortalitat hospitalisierter psychiatrischer Patienten--Ergebnisse einer 5-Jahres-Studie. 1980;48(11):616-27. doi: <https://dx.doi.org/10.1055/s-2007-1005820>.

11. Kondo S, Kumakura Y, Kanehara A, Nagato D, Ueda T, Matsuoka T, et al. Premature deaths among individuals with severe mental illness after discharge from long-term hospitalisation in Japan: A naturalistic observation during a 24-year period. BJPsych Open. 2017;3(4):193-495. doi: <http://dx.doi.org/10.1192/bjpo.bp.117.004937>.

12. Cohen D, Gasse C, Laursen T. Premature ageing as a proposed explanation for excess of death in schizophrenia. Schizophrenia Research. 2012;136:S122.

13. Enger C, Weatherby L, Reynolds RF, Glasser DB, Walker AM. Serious cardiovascular events and mortality among patients with schizophrenia. Journal of Nervous & Mental Disease. 2004;192(1):19-27. PubMed PMID: 14718772.

14. Joukamaa M, Heliovaara M, Knekt P, Aromaa A, Raitasalo R, Lehtinen V. Mental disorders and cause-specific mortality. British Journal of Psychiatry. 2001;179:498-502. PubMed PMID: 11731351.
