# Supplementary material for: Temporal trends in associations between severe mental illness and risk of cardiovascular disease: A systematic review and meta-analysis
Source: PLoS Med. 2022 Apr 19;19(4):e1003960. doi: 10.1371/journal.pmed.1003960 (PMC9017899; doi:10.1371/journal.pmed.1003960)
Supplement: S22 File — Fig A: Funnel plots for visual assessment of publication bias for studies reporting relative risk of CVD mortality for schizophrenia compared with controls, CVA. Fig B: Funnel plots for visual assessment of publication bias for studies reporting relative risk of CVD mortality for schizophrenia compared with controls, CHD. Fig C: Funnel plots for visual assessment of publication bias for studies reporting relative risk of CVD mortality for schizophrenia compared with controls, all circulatory disease. Fig D: Funnel plots for visual assessment of publication bias for studies reporting relative risk of CVD mortality for BD compared with controls, CVA. Fig E: Funnel plots for visual assessment of publication bias for studies reporting relative risk of CVD mortality for BD compared with controls, CHD. Fig F: Funnel plots for visual assessment of publication bias for studies reporting relative risk of CVD mortality for BD compared with controls, all circulatory disease. Table A: Results of Egger tests for publication bias, mortality outcomes. Fig G: Funnel plot of schizophrenia and CHD mortality including 5 unpublished studies estimated from trim and fill. Fig H: Funnel plot of schizophrenia and all circulatory disease mortality including 2 unpublished studies estimated from trim and fill. BD, bipolar disorder; CHD, coronary heart disease; CVA, cerebrovascular accident; CVD, cardiovascular disease. (PDF) [file pmed.1003960.s022.pdf]

S22 File. Assessment of publication bias: mortality studies

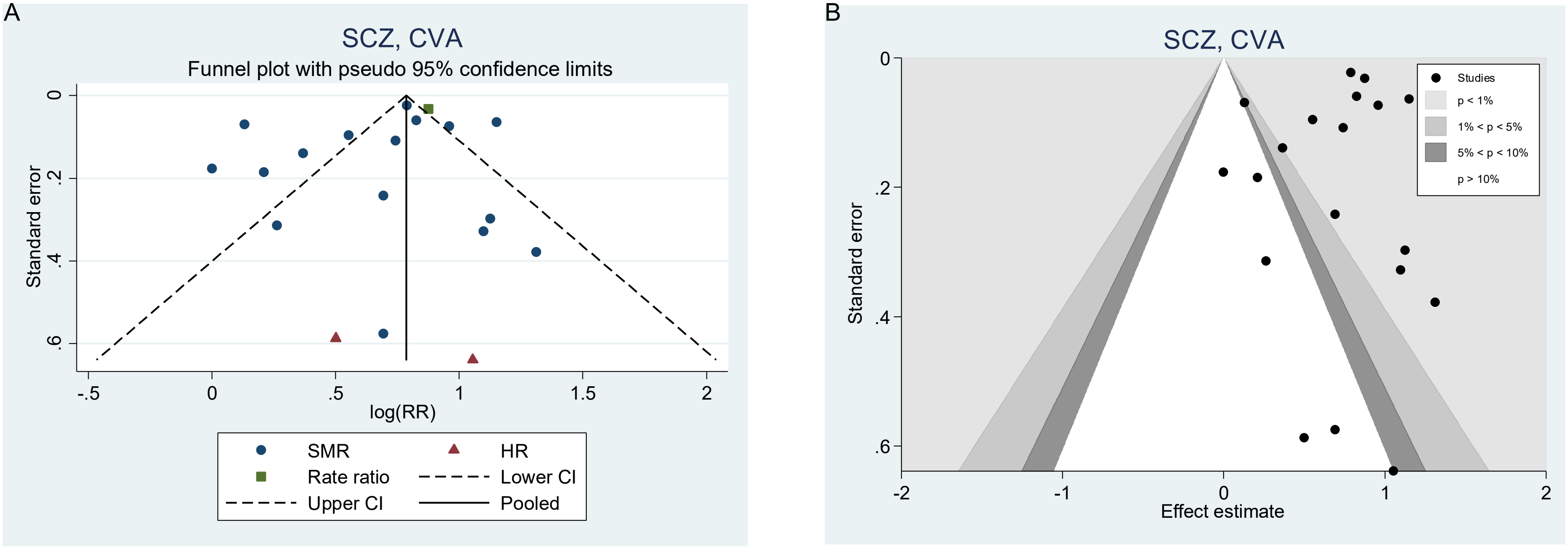

SCZ – schizophrenia, CVA – cerebrovascular accident, SMR – standardised mortality ratio, HR – hazard ratio

Fig A: Funnel plots for visual assessment of publication bias for studies reporting relative risk of CVD mortality for schizophrenia compared with controls, cerebrovascular accident

There is some asymmetry in studies reporting SMRs on the upper right-hand side of the funnel plot (A), suggesting missing studies falling outside the pseudo 95% confidence limits. In the contour-enhanced funnel plot (B) the "missing" studies would be in the shaded area of the graph, so would be expected to report significant effects, thus publication bias is unlikely [1]. Further, Egger's test [2] (Table S1) is non-significant. Publication bias is therefore not suspected.

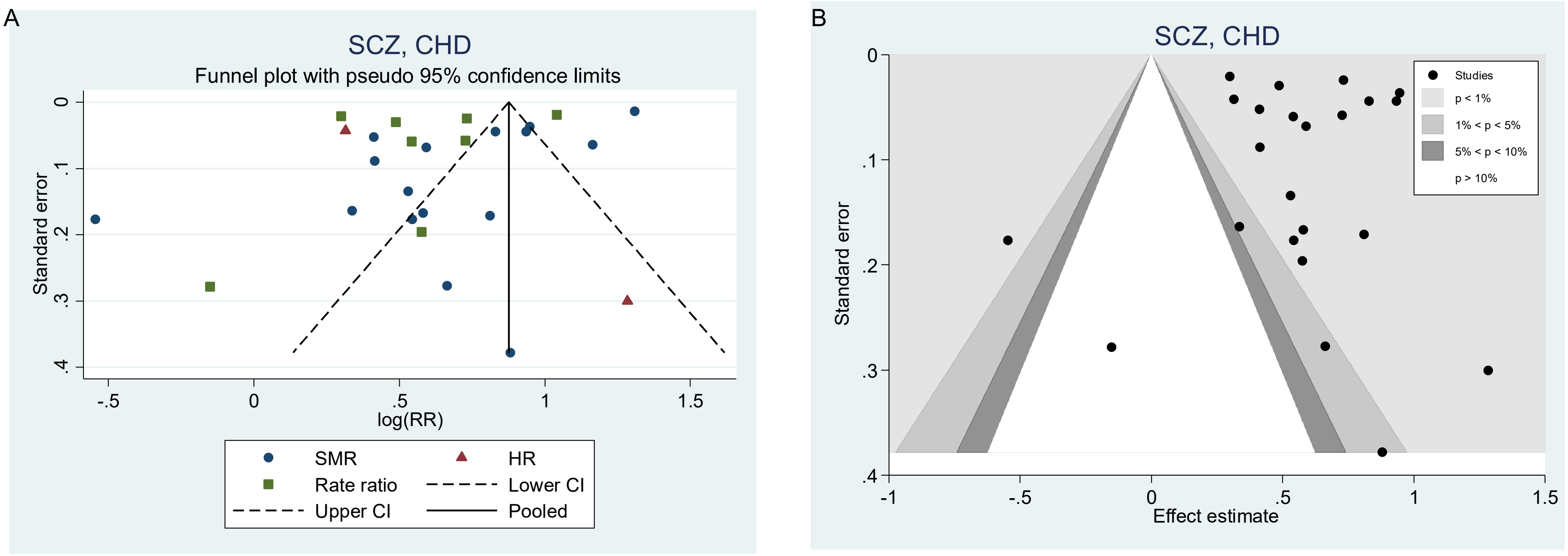

SCZ – schizophrenia, CHD – coronary heart disease, SMR – standardised mortality ratio, HR – hazard ratio

Fig B: Funnel plots for visual assessment of publication bias for studies reporting relative risk of CVD mortality for schizophrenia compared with controls, coronary heart disease

There is asymmetry on the right-hand side of the funnel plot (A), affecting SMRs and rate ratios outside the pseudo 95% confidence limits and SMRs inside the limits, suggesting missing studies. In the contour-enhanced funnel plot (B) the "missing" studies would be in the shaded area of the graph, so would be expected to report significant effects, therefore publication bias is unlikely. However, Egger's test (Table P1) for publication bias is significant for studies reporting SMRs *i.e.* the confidence interval for the intercept does not include zero (bias: -7.01, 95% CI: -10.51 to -3.50, p=0.001). Applying a trim and fill procedure (Fig G) suggests five missing unpublished studies reporting the association between CHD mortality and schizophrenia.

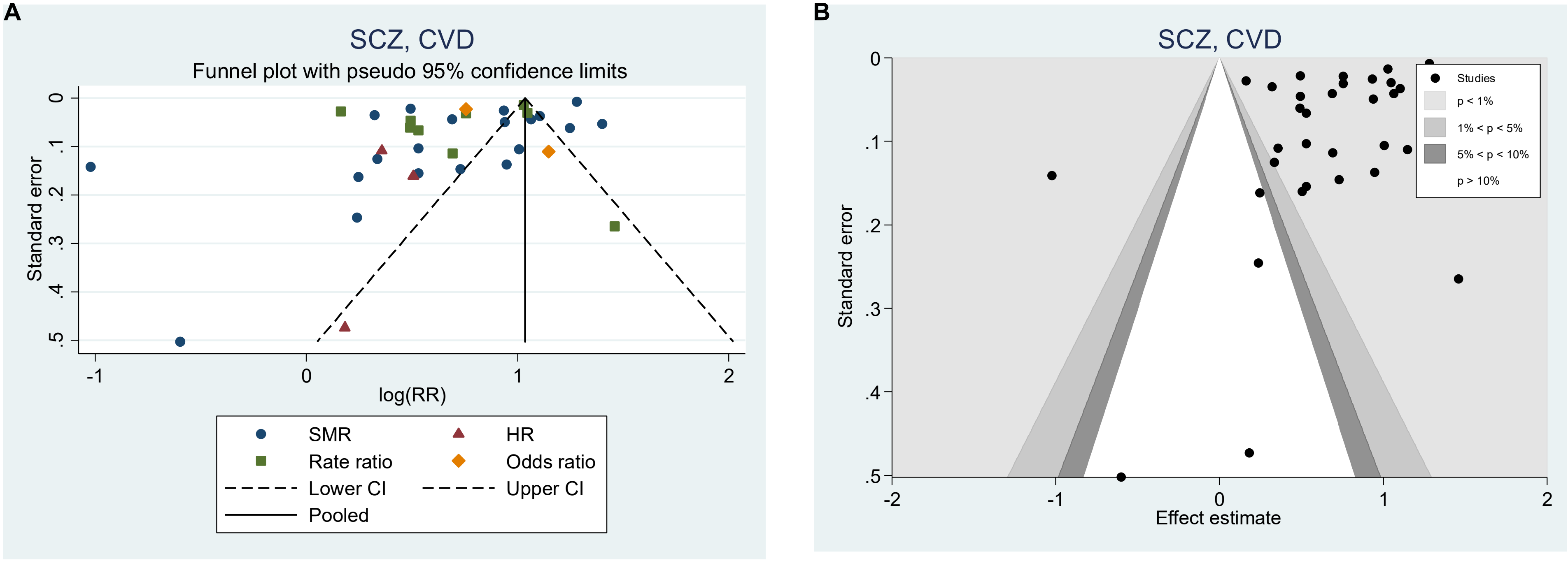

SCZ – schizophrenia, CVD – all circulatory disease, SMR – standardised mortality ratio, HR – hazard ratio

Fig C: Funnel plots for visual assessment of publication bias for studies reporting relative risk of CVD mortality for schizophrenia compared with controls, all circulatory disease

There is asymmetry on the right-hand side of the funnel plot (A), affecting SMRs and rate ratios outside the pseudo 95% confidence limits, suggesting missing studies. In the contour-enhanced funnel plot (B) the "missing" studies would be in the shaded area of the graph, so would be expected to report significant effects, therefore publication bias is unlikely. However, Egger's test for publication bias is significant for studies reporting SMRs and for studies reporting all effect types *i.e.* the confidence interval for the intercept does not include zero (bias for SMR studies: -7.76, 95% CI: -13.43 to -2.09, p=0.01). Two additional studies were found when a trim and fill procedure [3] was conducted (Fig H).

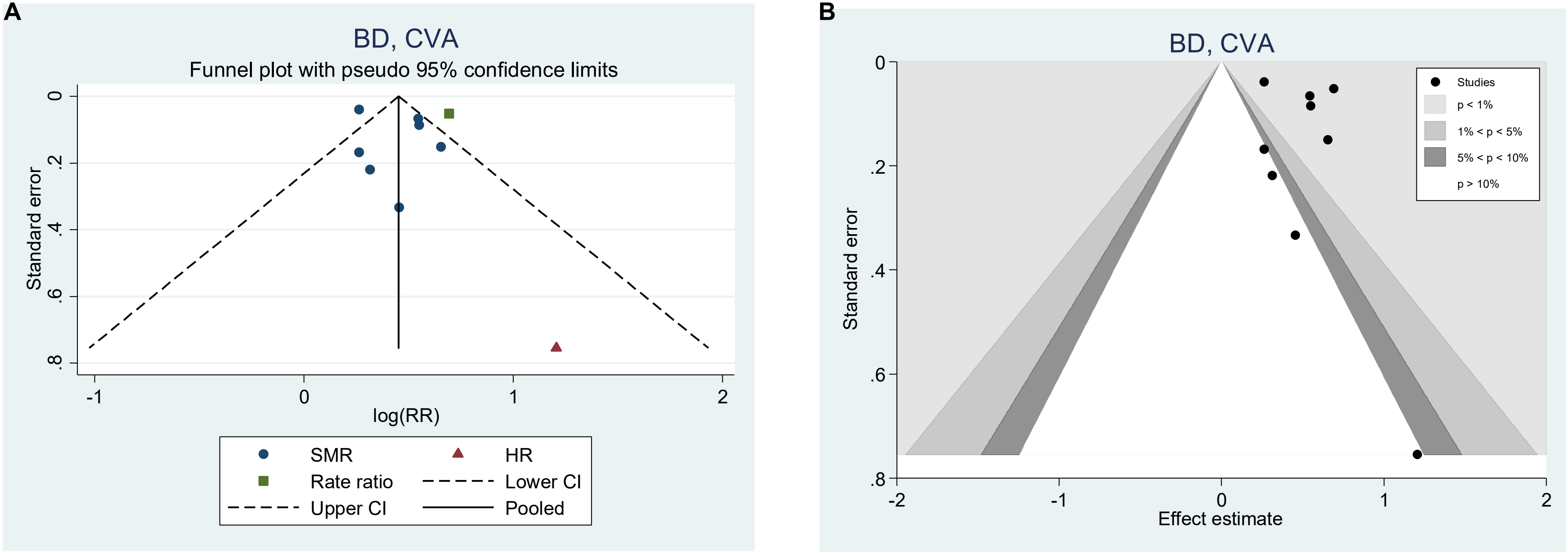

BD – bipolar disorder, CVA – cerebrovascular accident, SMR – standardised mortality ratio, HR – hazard ratio

Fig D: Funnel plots for visual assessment of publication bias for studies reporting relative risk of CVD mortality for bipolar disorder compared with controls, cerebrovascular accident

There are too few studies to assess publication bias.

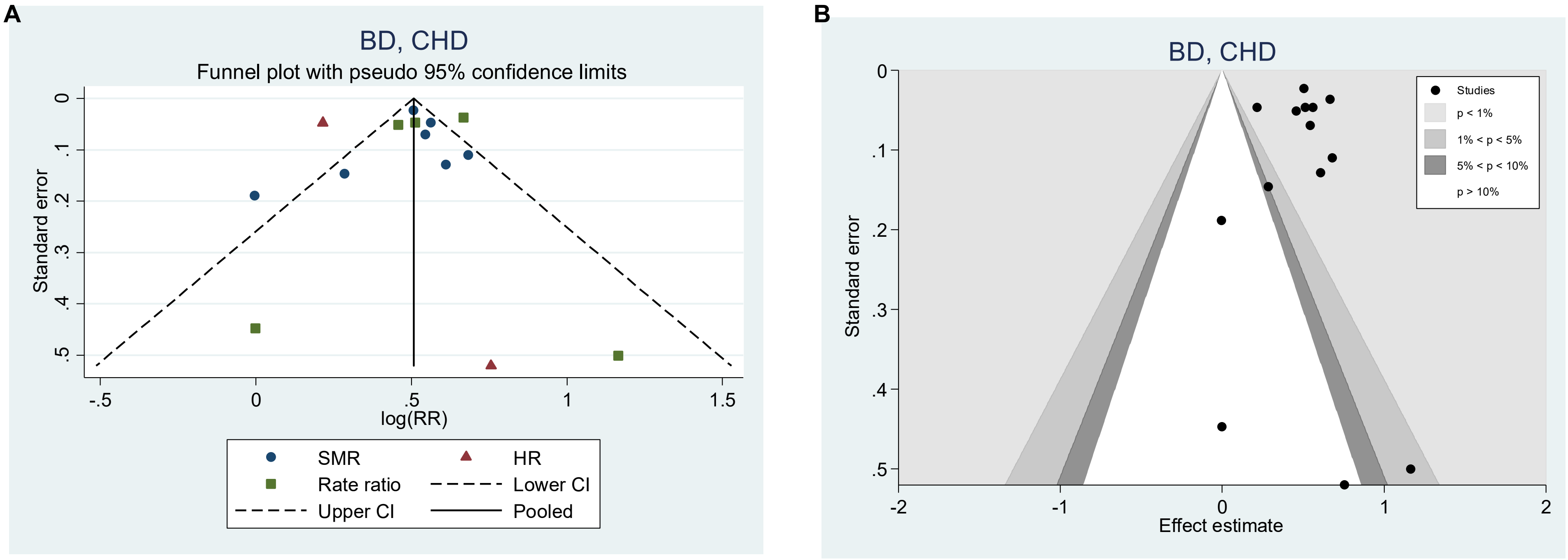

BD – bipolar disorder, CHD – coronary heart disease, SMR – standardised mortality ratio, HR – hazard ratio

Fig E: Funnel plots for visual assessment of publication bias for studies reporting relative risk of CVD mortality for bipolar disorder compared with controls, coronary heart disease

Plot A shows little asymmetry, so publication bias is not suspected.

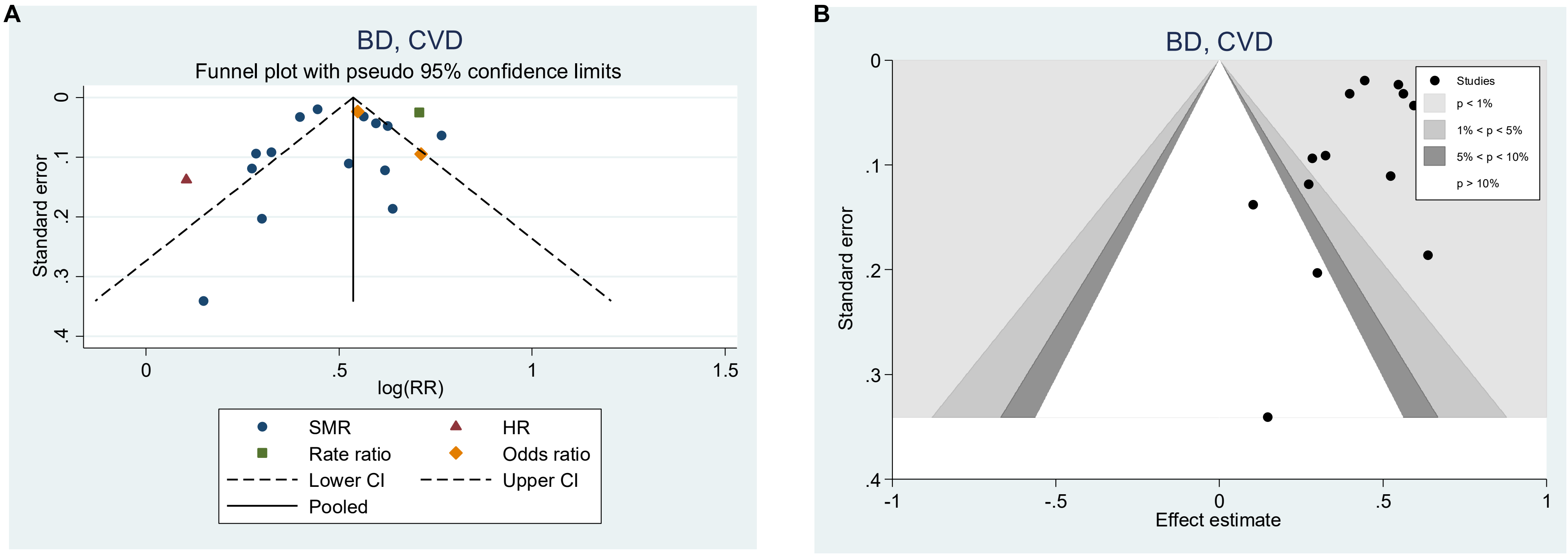

BD – bipolar disorder, CVD all circulatory disease, SMR – standardised mortality ratio, HR – hazard ratio

Fig F: Funnel plots for visual assessment of publication bias for studies reporting relative risk of CVD mortality for bipolar disorder compared with controls, all circulatory disease

Plot A shows little asymmetry, so publication bias is not suspected.

Table A: Results of Egger's tests for publication bias, mortality outcomes

SMI	Mortality outcome	Effect type	Bias (95% CI)	p-value
Schizophrenia	Cerebrovascular accident	SMR	-1.04 (-3.68, 1.60)	0.412
Schizophrenia	Cerebrovascular accident	HR/Rate ratio	Too few studies	
Schizophrenia	Cerebrovascular accident	All effect types	-1.01 (-3.17, 1.16)	0.340
Schizophrenia	Coronary heart disease	SMR	**-7.01** (-10.51, -3.50)	0.001
Schizophrenia	Coronary heart disease	HR/Rate ratio	-2.63 (-15.18, 9.92)	0.642
Schizophrenia	Coronary heart disease	All effect types	-5.19 (-10.87, 0.50)	0.072
Schizophrenia	All circulatory disease	SMR	**-7.76** (-13.43, -2.09)	0.010
Schizophrenia	All circulatory disease	HR/Rate ratio	-4.32 (-11.99, 3.36)	0.244
Schizophrenia	All circulatory disease	All effect types	**-7.67** (-12.22, -3.11)	0.002
Bipolar disorder	Cerebrovascular accident	SMR	Too few studies	-
Bipolar disorder	Cerebrovascular accident	HR/Rate ratio	Too few studies	-
Bipolar disorder	Cerebrovascular accident	All effect types	Too few studies	-
Bipolar disorder	Coronary heart disease	SMR	Too few studies	-
Bipolar disorder	Coronary heart disease	HR/Rate ratio	Too few studies	-
Bipolar disorder	Coronary heart disease	All effect types	-0.41 (-2.76, 1.94)	0.712
Bipolar disorder	All circulatory disease	SMR	0.22 (-2.00, 2.44)	0.833
Bipolar disorder	All circulatory disease	HR/Rate ratio	Too few studies	-
Bipolar disorder	All circulatory disease	All effect types	-0.54 (-2.95, 1.88)	0.644

SMI – severe mental illness, SMR – standardised mortality ratio, HR – hazard ratio, CI – confidence intervals
Results where 95% confidence intervals exclude the null highlighted in ***bold***

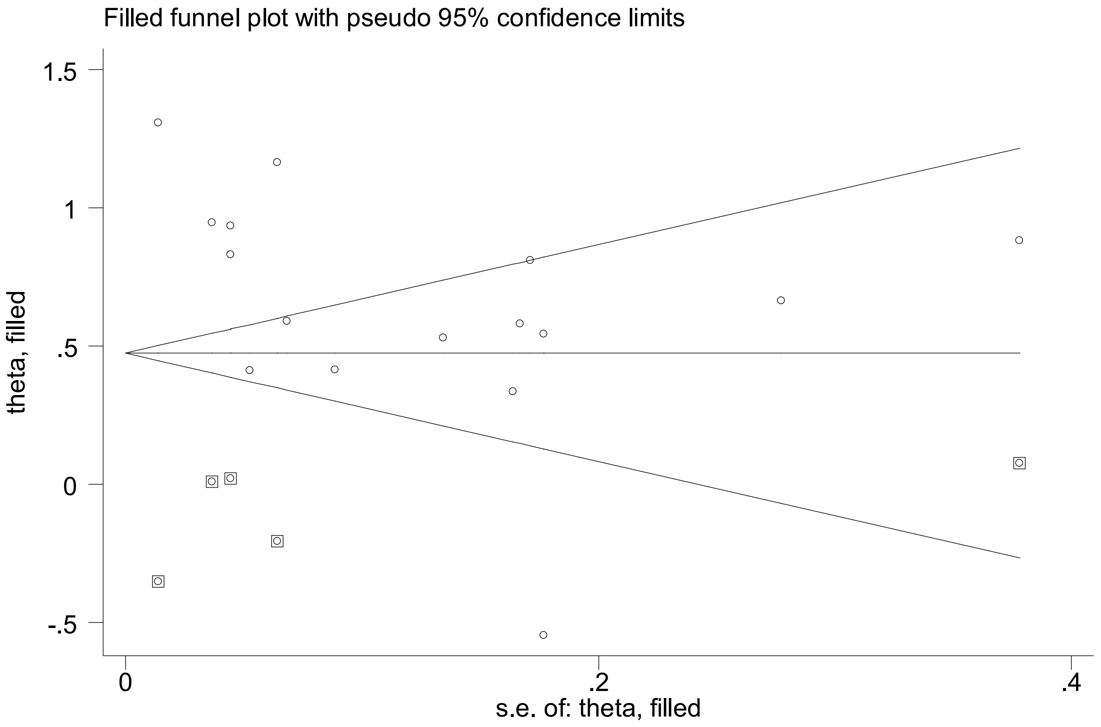

Fig G: Funnel plot of schizophrenia and coronary heart disease mortality including five unpublished studies estimated from trim and fill

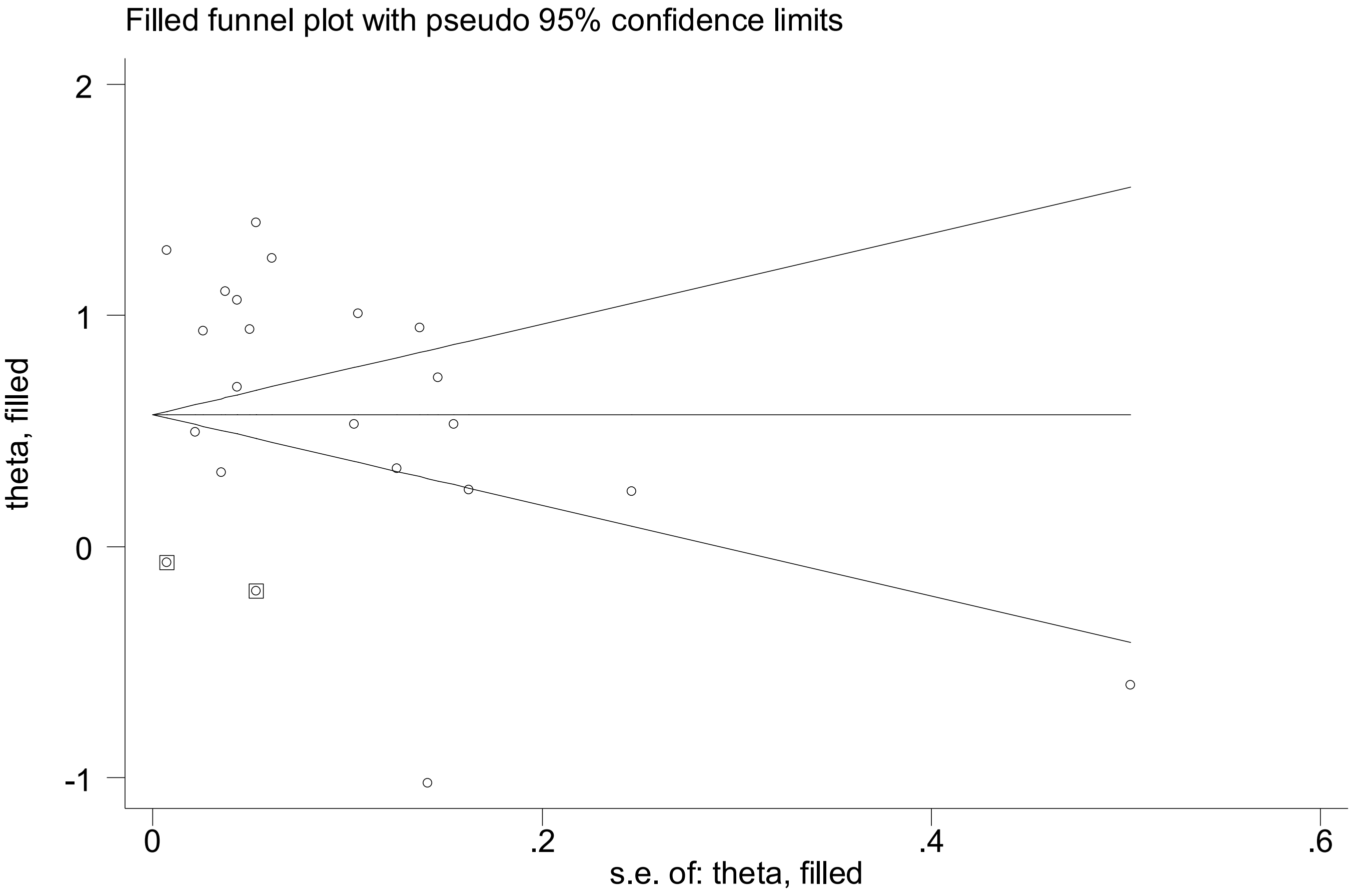

Fig H: Funnel plot of schizophrenia and all circulatory disease mortality including two unpublished studies estimated from trim and fill

References

1. Peters JL, Sutton AJ, Jones DR, Abrams KR, Rushton L. Contour-enhanced meta-analysis funnel plots help distinguish publication bias from other causes of asymmetry. Journal of Clinical Epidemiology. 2008;61(10):991-6. doi: https://doi.org/10.1016/j.jclinepi.2007.11.010.
2. Page MJ, Higgins JPT, Sterne JAC. Chapter 13: Assessing risk of bias due to missing results in a synthesis. 2019. In: Cochrane Handbook for Systematic Reviews of Interventions, Version 6.0. Cochrane, [cited June 12, 2020]. Available from: www.training.cochrane.org/handbook.
3. Sterne JAC, Egger M, Moher D. Chapter 10: Addressing reporting biases. In: Higgins J, Green S, editors. Cochrane Handbook for Systematic Reviews of Interventions. Version 5.1.0 [updated March 2011] ed: The Cochrane Collaboration; 2011.
