# Supplementary material for: Temporal trends in associations between severe mental illness and risk of cardiovascular disease: A systematic review and meta-analysis
Source: PLoS Med. 2022 Apr 19;19(4):e1003960. doi: 10.1371/journal.pmed.1003960 (PMC9017899; doi:10.1371/journal.pmed.1003960)
Supplement: S23 File — Fig A: Funnel plots for visual assessment of publication bias for studies reporting relative risk of CVD incidence for schizophrenia compared with controls, CVA. Fig B: Funnel plots for visual assessment of publication bias for studies reporting relative risk of CVD incidence for schizophrenia compared with controls, CHD. Fig C: Funnel plots for visual assessment of publication bias for studies reporting relative risk of CVD incidence for schizophrenia compared with controls, major cardiovascular events. Fig D: Funnel plots for visual assessment of publication bias for studies reporting relative risk of CVD incidence for schizophrenia compared with controls, heart failure. Fig E: Funnel plots for visual assessment of publication bias for studies reporting relative risk of CVD incidence for BD compared with controls, CVA. Fig F: Funnel plots for visual assessment of publication bias for studies reporting relative risk of CVD incidence for BD compared with controls, CHD. Fig G: Funnel plots for visual assessment of publication bias for studies reporting relative risk of CVD incidence for BD compared with controls, major cardiovascular events. Fig H: Funnel plots for visual assessment of publication bias for studies reporting relative risk of CVD incidence for BD compared with controls, heart failure. Fig I: Funnel plots for visual assessment of publication bias for studies reporting relative risk of CVD incidence for schizophrenia compared with controls, all CVD. Fig J: Funnel plots for visual assessment of publication bias for studies reporting relative risk of CVD incidence for BD compared with controls, all CVD. Fig K: Funnel plots for visual assessment of publication bias for studies reporting relative risk of CVD incidence for schizophrenia and BD compared with controls, CVA. Fig L: Funnel plots for visual assessment of publication bias for studies reporting relative risk of CVD incidence for schizophrenia and BD compared with controls, CHD. Fig M: Funnel plots f [file pmed.1003960.s023.pdf]

S23 File. Assessment of publication bias: incidence studies

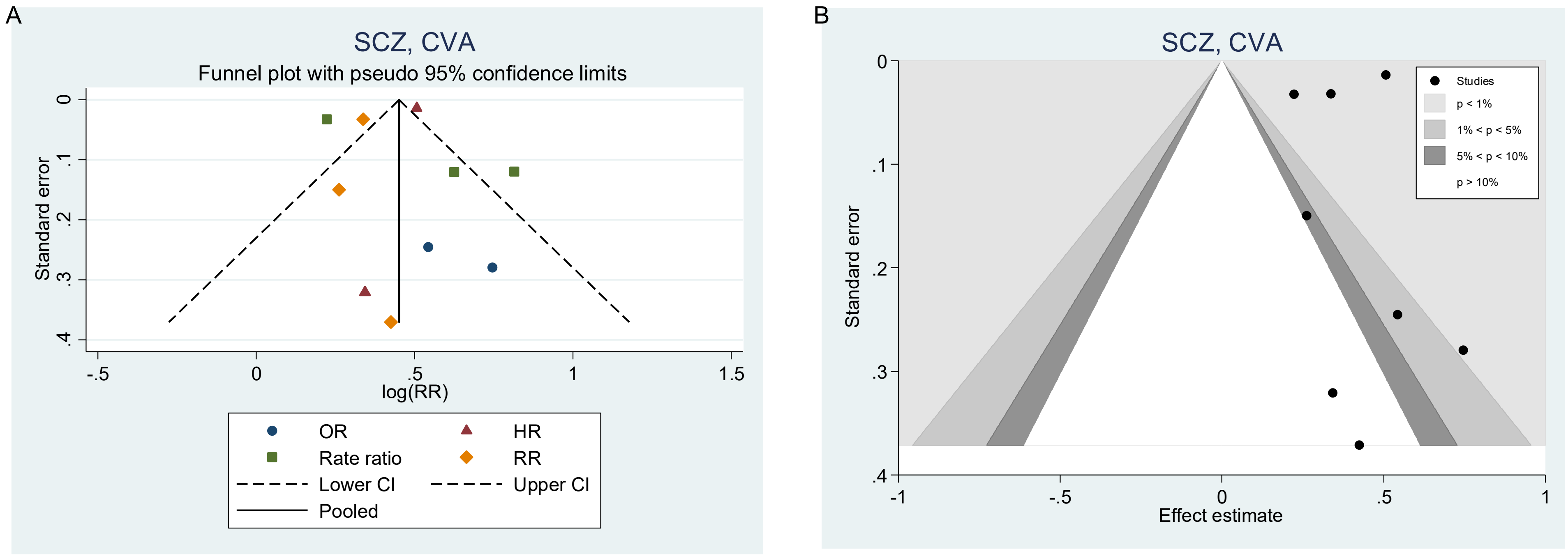

SCZ – schizophrenia, CVA – cerebrovascular accident, OR – odds ratio, HR – hazard ratio, RR – risk ratio

Fig A: Funnel plots for visual assessment of publication bias for studies reporting relative risk of CVD incidence for schizophrenia compared with controls, cerebrovascular accident

Plot A shows little asymmetry, so publication bias is not suspected.

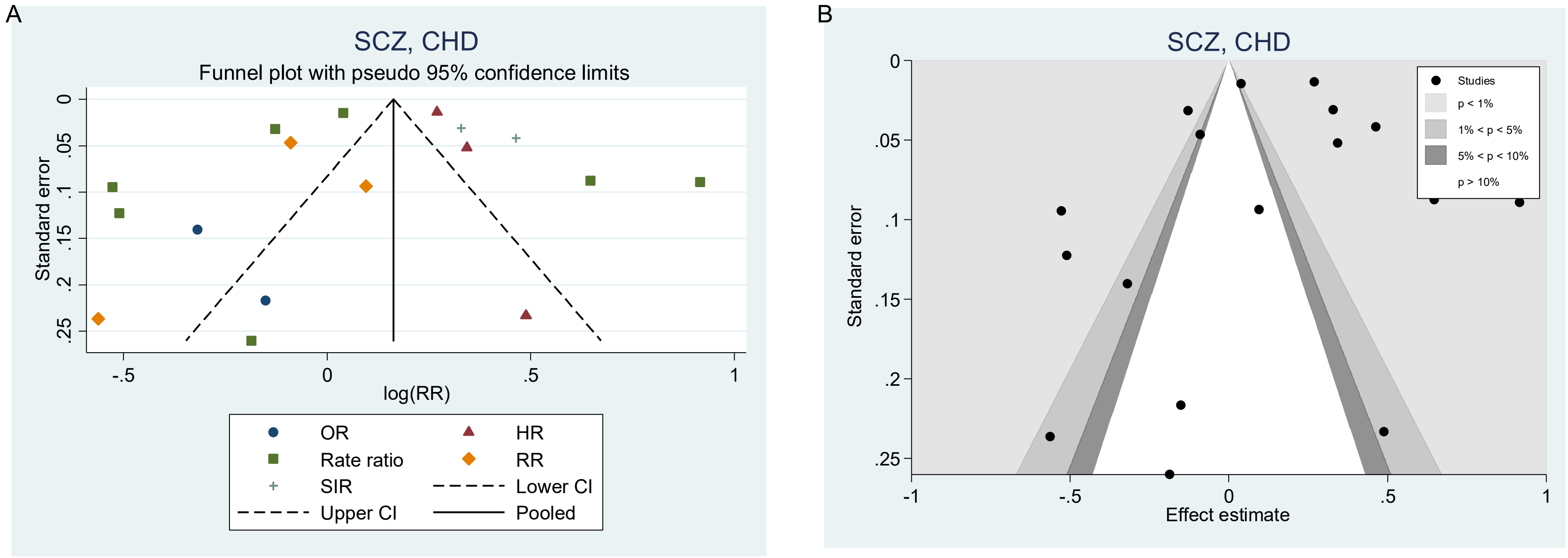

SCZ – schizophrenia, CVA – cerebrovascular accident, OR – odds ratio, HR – hazard ratio, RR – risk ratio

Fig B: Funnel plots for visual assessment of publication bias for studies reporting relative risk of CVD incidence for schizophrenia compared with controls, coronary heart disease

There is some asymmetry on the lower right-hand side of the funnel plot (A), suggesting missing studies falling outside the pseudo 95% confidence limits. In the contour-enhanced funnel plot (B) the "missing" studies would be in the shaded area of the graph, so would be expected to report significant effects. Further, Egger's test is non-significant. Publication bias is therefore not suspected.

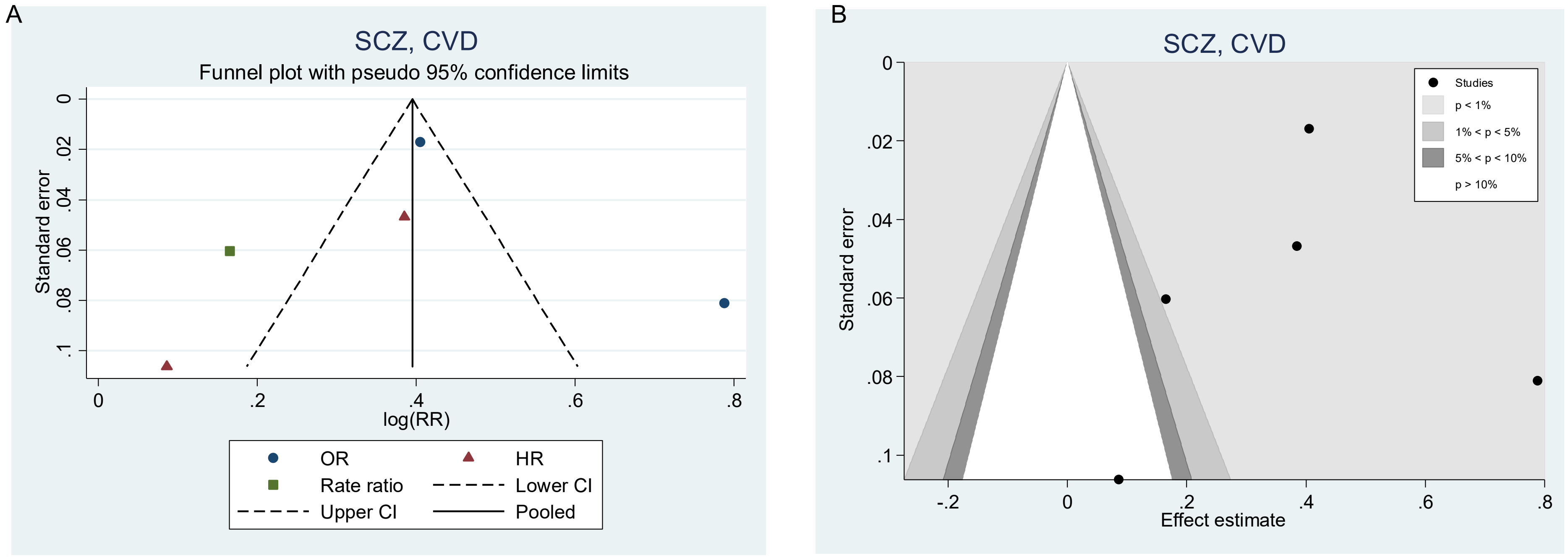

SCZ – schizophrenia, CVD – major cardiovascular events, OR – odds ratio, HR – hazard ratio, RR- risk ratio

Fig C: Funnel plots for visual assessment of publication bias for studies reporting relative risk of CVD incidence for schizophrenia compared with controls, major cardiovascular events

There are too few studies to assess publication bias.

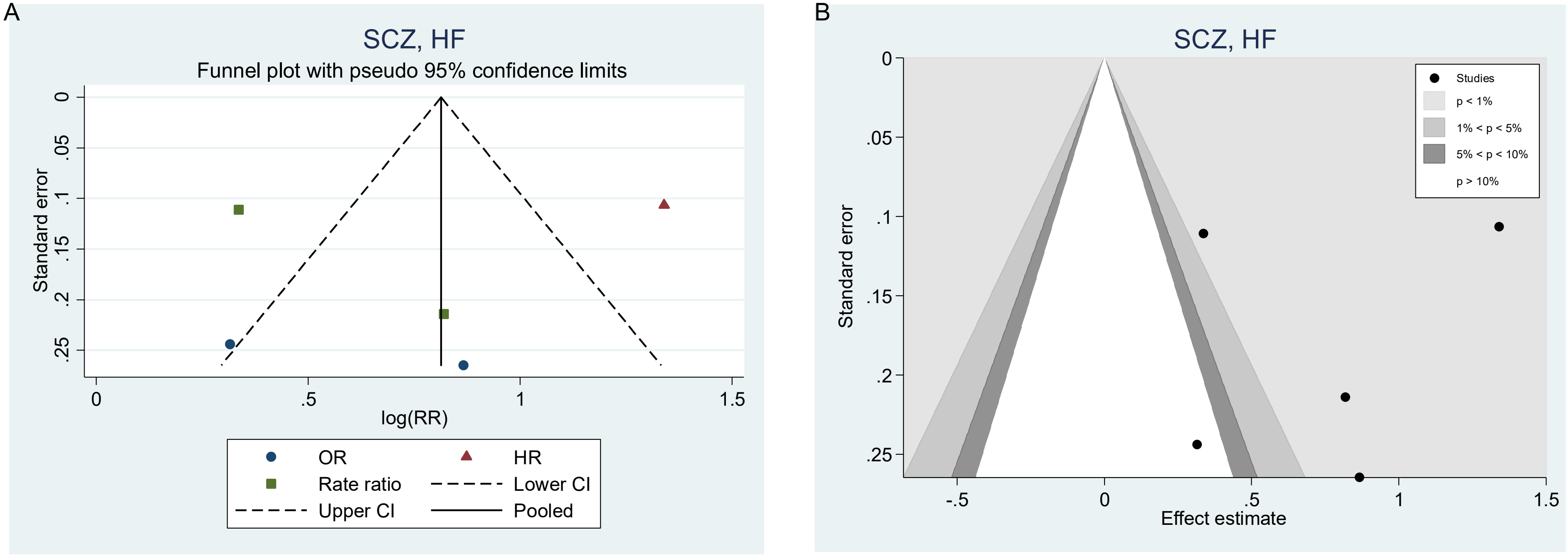

SCZ – schizophrenia, HF – heart failure, OR – odds ratio, HR – hazard ratio, RR- risk ratio

Fig D: Funnel plots for visual assessment of publication bias for studies reporting relative risk of CVD incidence for schizophrenia compared with controls, heart failure

There are too few studies to assess publication bias.

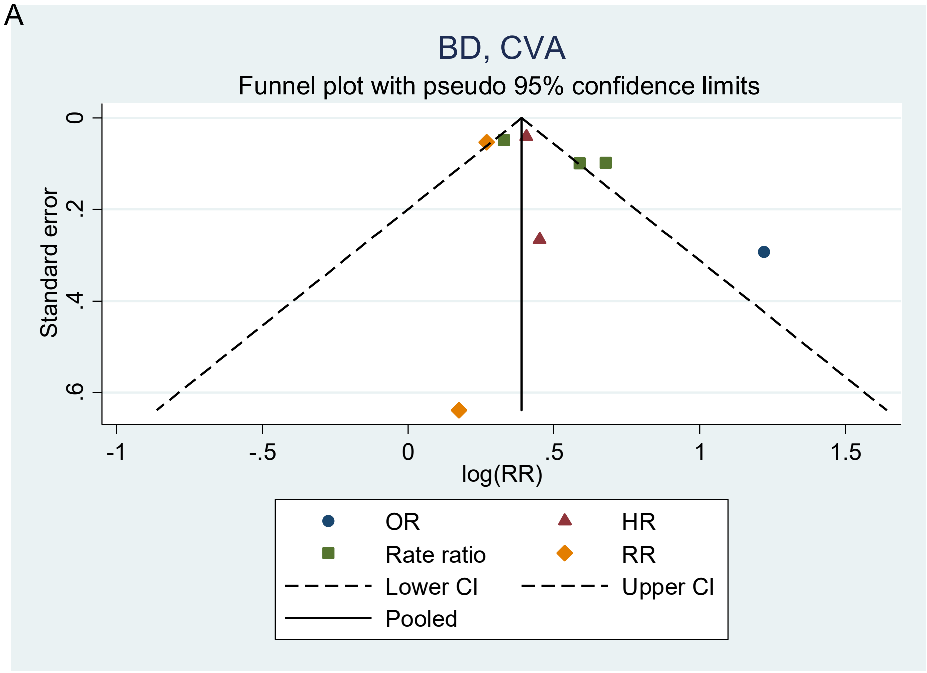

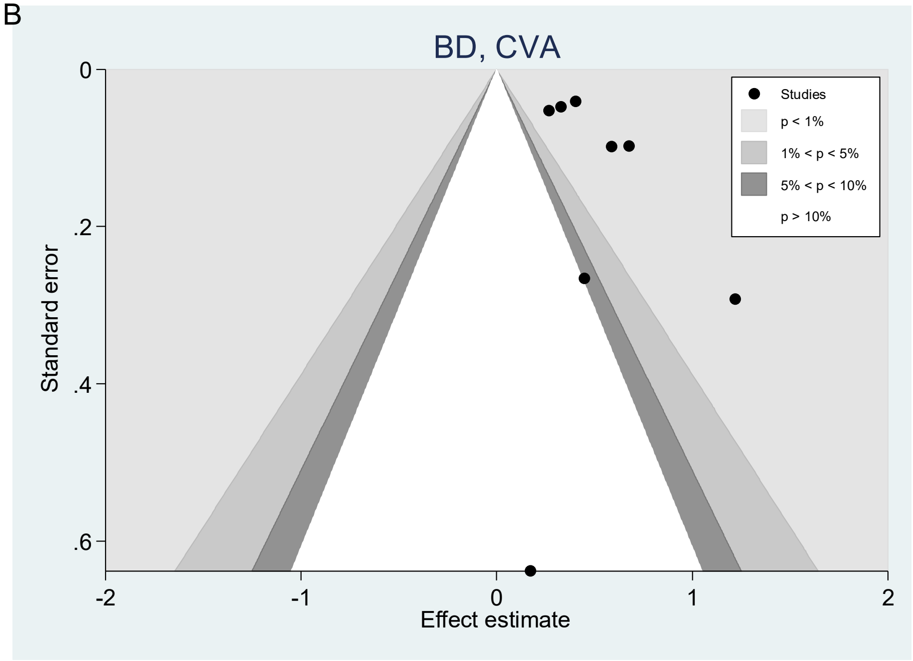

BD – bipolar disorder, CVA – cerebrovascular accident, OR – odds ratio, HR – hazard ratio, RR- risk ratio

Fig E: Funnel plots for visual assessment of publication bias for studies reporting relative risk of CVD incidence for bipolar disorder compared with controls, cerebrovascular accident

There are too few studies to assess publication bias.

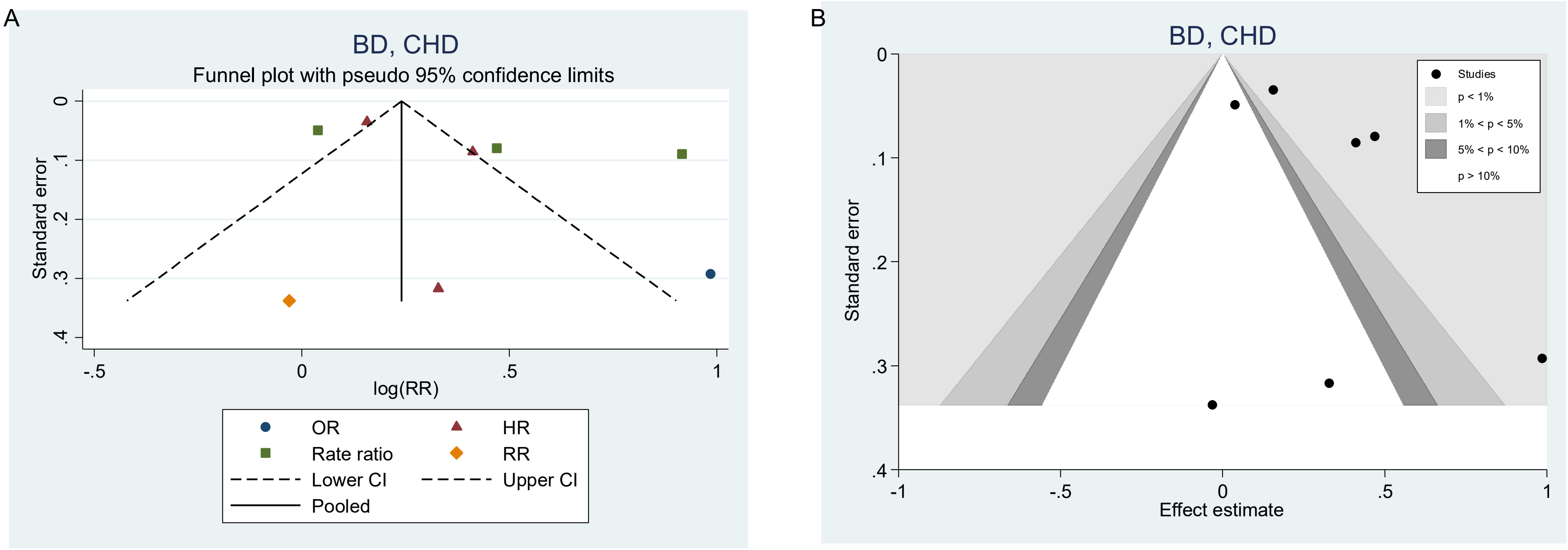

BD – bipolar disorder, CHD – coronary heart disease, OR – odds ratio, HR – hazard ratio, RR- risk ratio

Fig F: Funnel plots for visual assessment of publication bias for studies reporting relative risk of CVD incidence for bipolar disorder compared with controls, coronary heart disease

There are too few studies to assess publication bias.

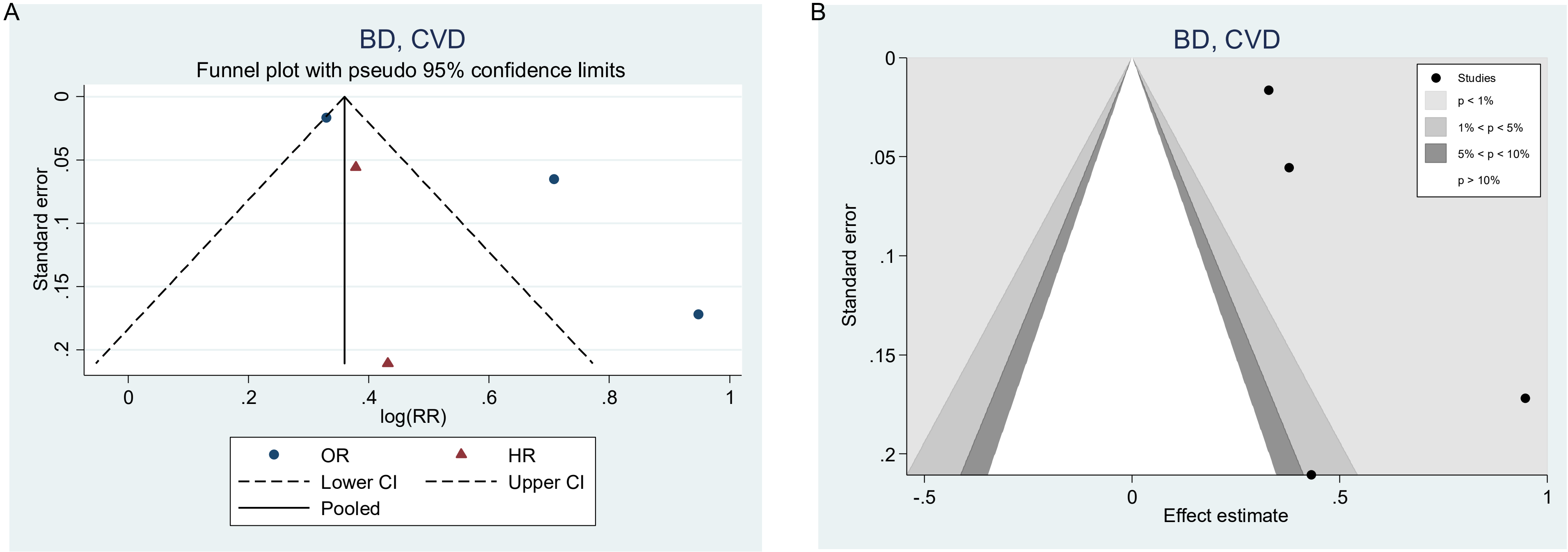

BD – bipolar disorder, CVD – major cardiovascular events, OR – odds ratio, HR – hazard ratio, RR- risk ratio

Fig G: Funnel plots for visual assessment of publication bias for studies reporting relative risk of CVD incidence for bipolar disorder compared with controls, major cardiovascular events

There are too few studies to assess publication bias.

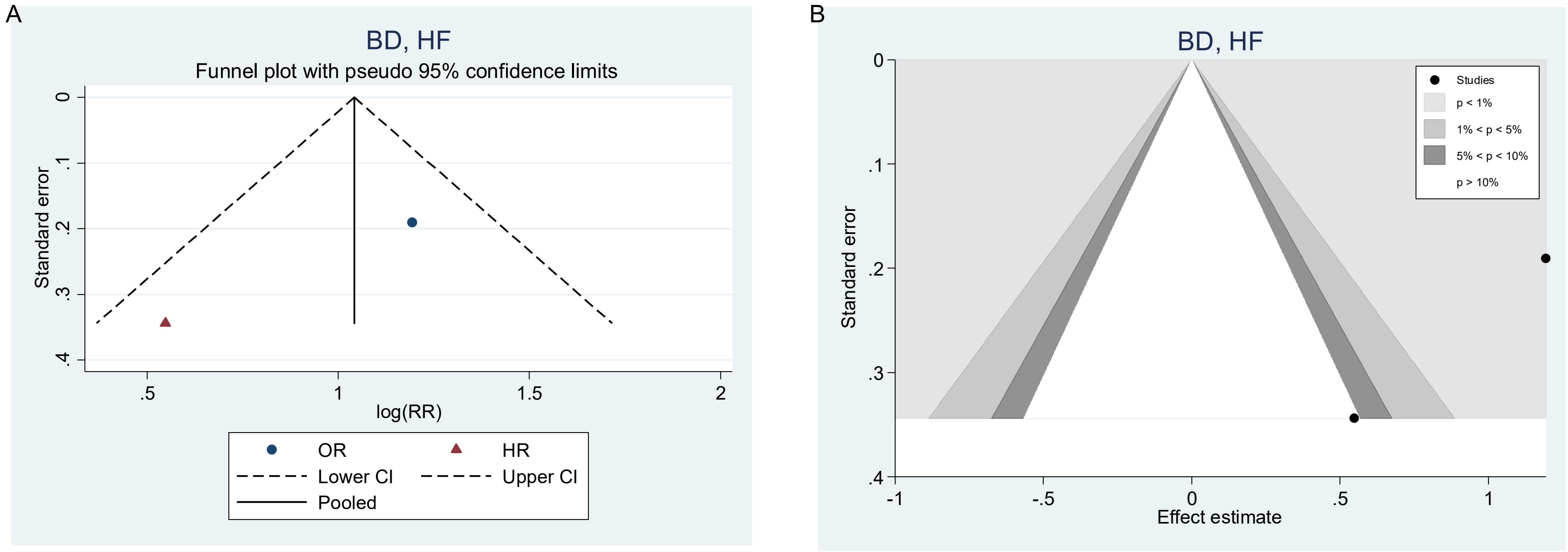

BD – bipolar disorder, HF – heart failure, OR – odds ratio, HR – hazard ratio, RR- risk ratio

Fig H: Funnel plots for visual assessment of publication bias for studies reporting relative risk of CVD incidence for bipolar disorder compared with controls, heart failure

There are too few studies to assess publication bias.

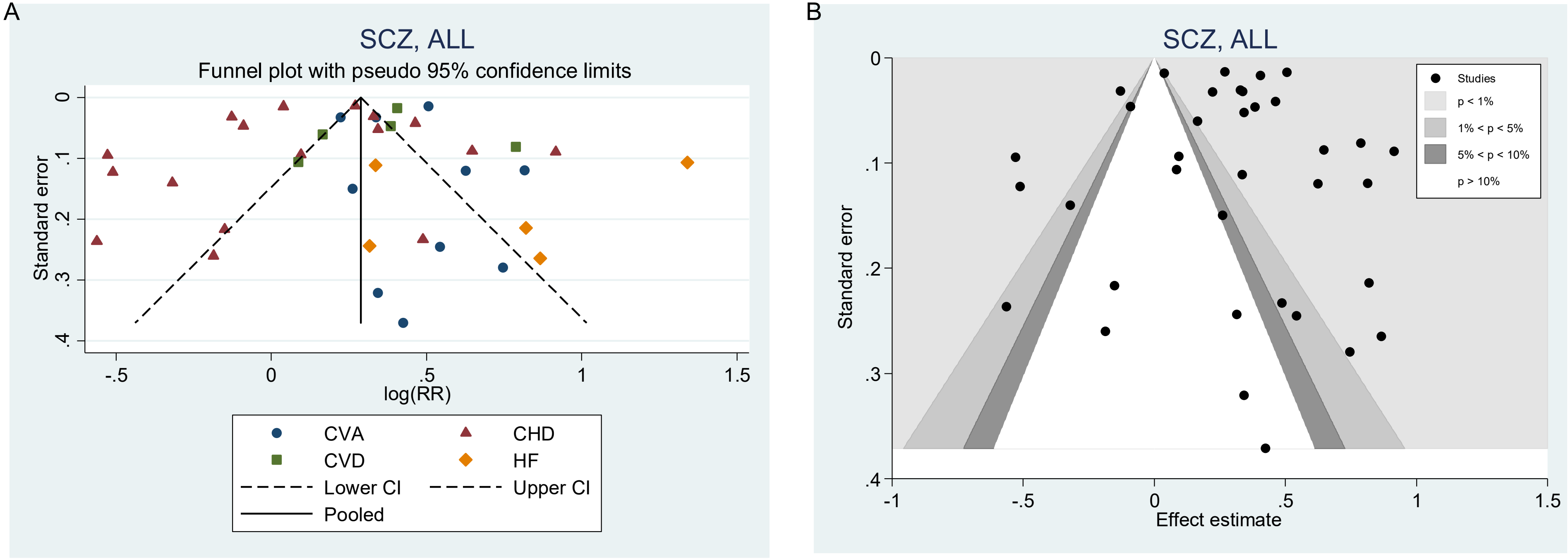

SCZ - schizophrenia, CVA – cerebrovascular accident, CHD – coronary heart disease, CVD – major cardiovascular events, HF – heart failure

Fig I: Funnel plots for visual assessment of publication bias for studies reporting relative risk of CVD incidence for schizophrenia compared with controls, all cardiovascular disease

The funnel plot (A) suggests some asymmetry in the bottom left-hand corner, which could be missing small studies. These would fall inside the white (non-significant) area of the contour-enhanced funnel plot (B), so could indicate possible publication bias. However, the results of Egger's test (0.08, 95% CI: -2.64 to 2.81, p=0.951) were non-significant for small study effects.

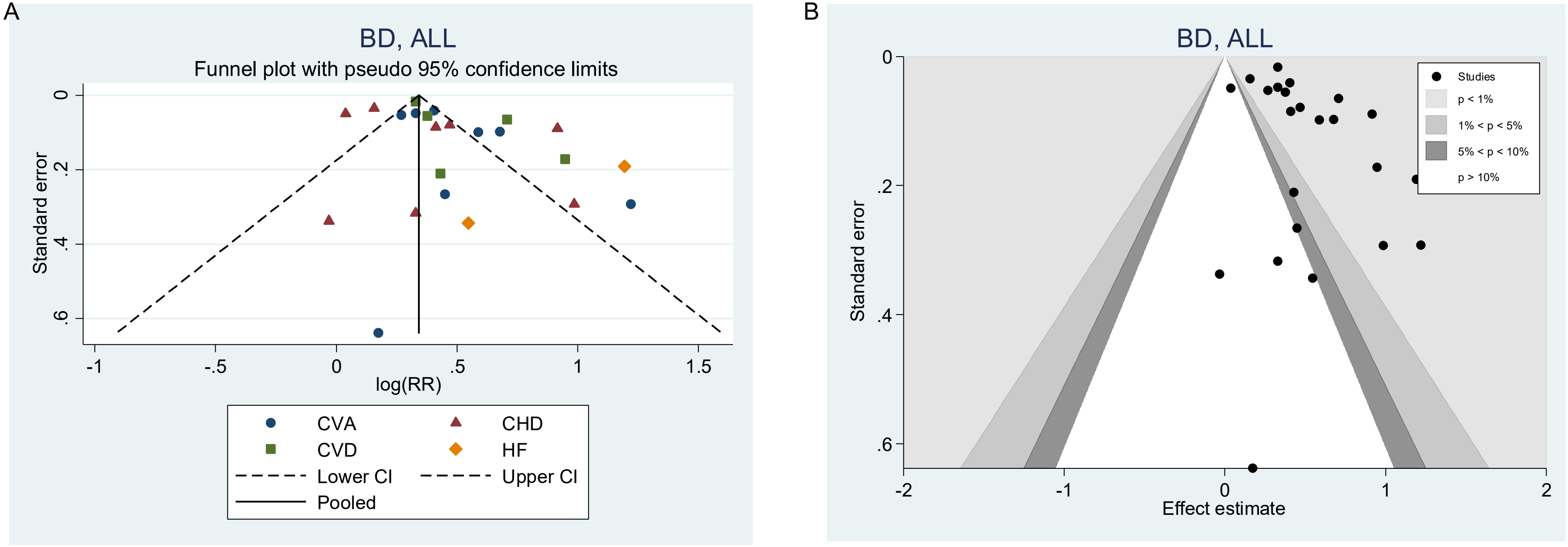

BD – bipolar disorder, CVA – cerebrovascular accident, CHD – coronary heart disease, CVD – major cardiovascular events, HF – heart failure

Fig J: Funnel plots for visual assessment of publication bias for studies reporting relative risk of CVD incidence for bipolar disorder compared with controls, all cardiovascular disease

There is some asymmetry in the upper left-hand side of the funnel plot (A), outside the pseudo 95% confidence limits, which could suggest missing studies. In the contour-enhanced funnel plot (B) the “missing” studies would fall mainly in the white (non-significant) area of the graph, so publication bias is a possibility. Further, Egger’s test indicates a significant small study effect bias (1.94, 95% CI: 0.16 to 3.72, p=0.034), therefore publication bias may exist. Also, some of the smaller studies fall into the significant (shaded) areas of plot B, suggesting that publication bias may not be the only cause of asymmetry in the plots [1]. However, applying trim and fill methodology indicates does not add any additional unpublished studies.

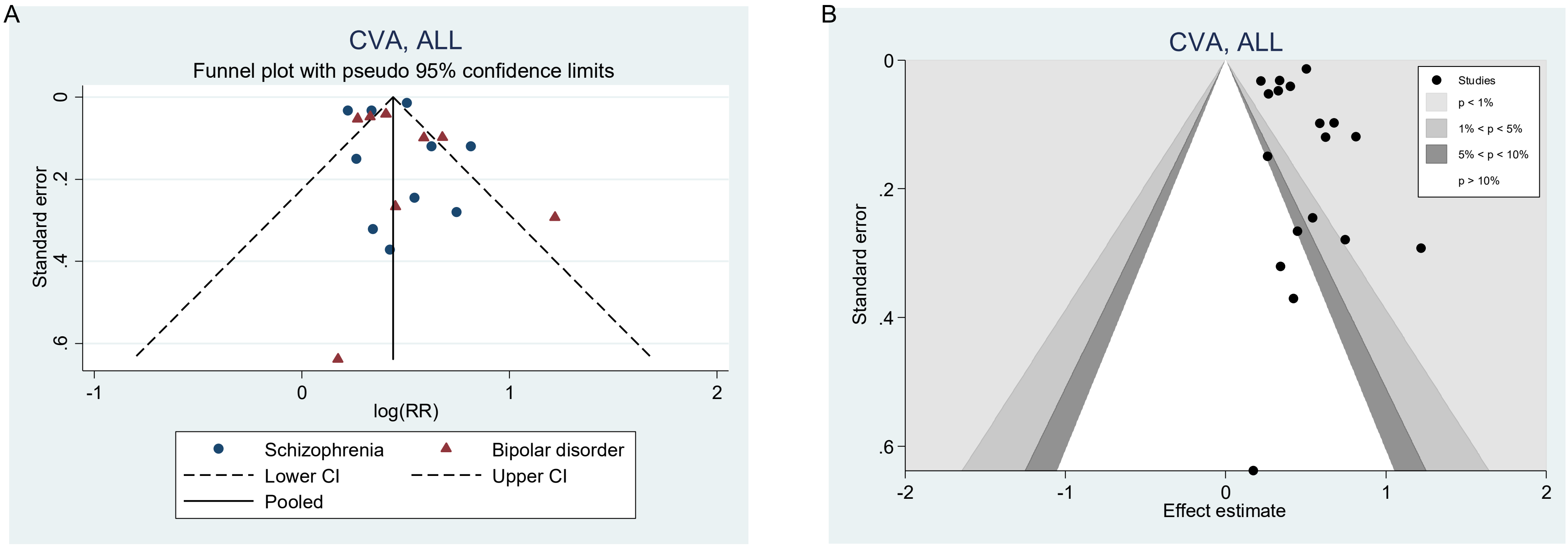

CVA – cerebrovascular accident

Fig K: Funnel plots for visual assessment of publication bias for studies reporting relative risk of CVD incidence for schizophrenia and bipolar disorder compared with controls, cerebrovascular accident

The funnel plot (A) suggests some asymmetry on the left-hand side, within the pseudo 95% confidence limits, which could be missing smaller studies. These would fall inside the white (non-significant) area of the contour-enhanced funnel plot (B), so could indicate possible publication bias. However, the results of Egger's test (-0.05, 95% CI: -1.89 to 1.80, p=0.0.957) were non-significant for small study effects.

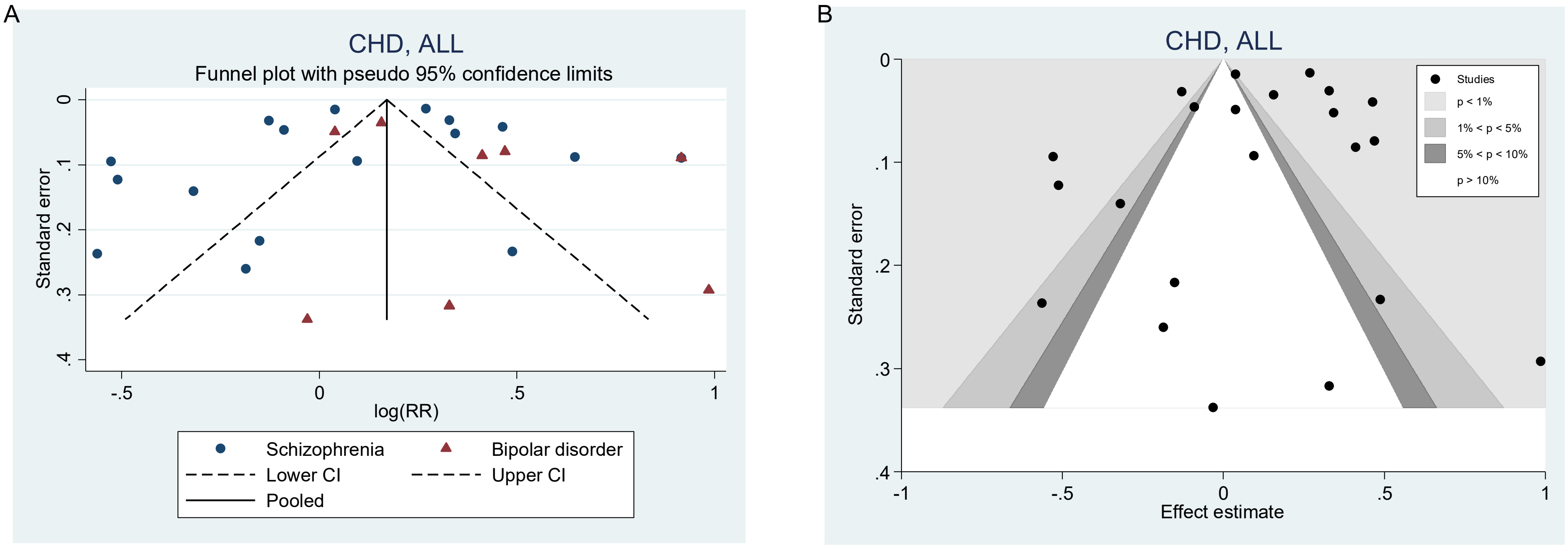

CHD – coronary heart disease

Fig L: Funnel plots for visual assessment of publication bias for studies reporting relative risk of CVD incidence for schizophrenia and bipolar disorder compared with controls, coronary heart disease

There is some asymmetry in the upper right-hand side of the funnel plot (A), outside the pseudo 95% confidence limits, which could suggest missing studies. In the contour-enhanced funnel plot (B) the “missing” studies would fall into the shaded area, indicating that publication bias is unlikely.

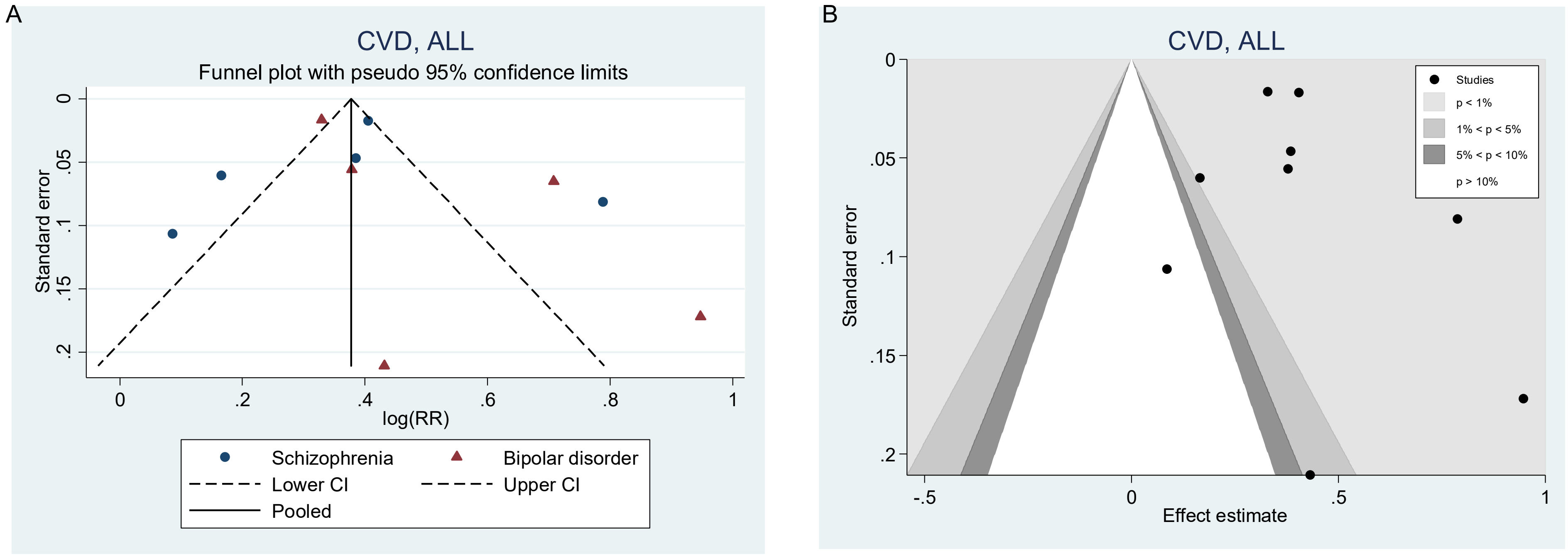

CVD – major cardiovascular events

Fig M: Funnel plots for visual assessment of publication bias for studies reporting relative risk of CVD incidence for schizophrenia and bipolar disorder compared with controls, major cardiovascular events

Plot A shows little asymmetry, so publication bias is not suspected.

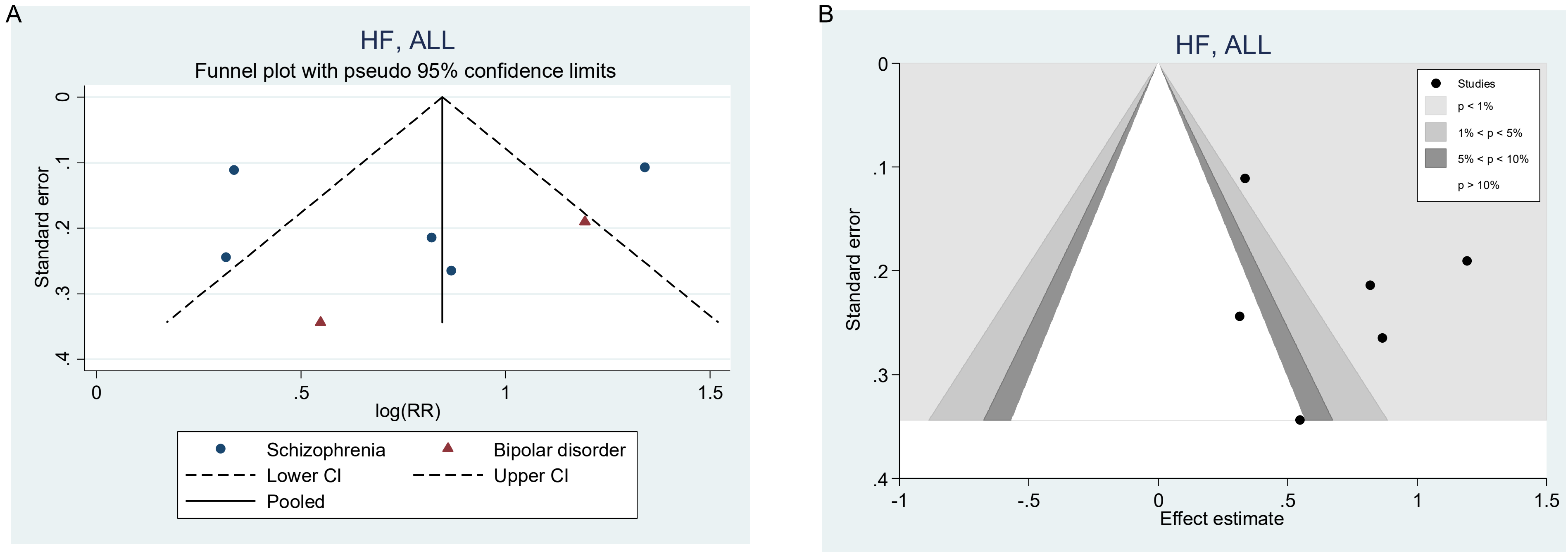

HF – heart failure

Fig N: Funnel plots for visual assessment of publication bias for studies reporting relative risk of CVD incidence for schizophrenia and bipolar disorder compared with controls, heart failure

There are too few studies to assess publication bias.

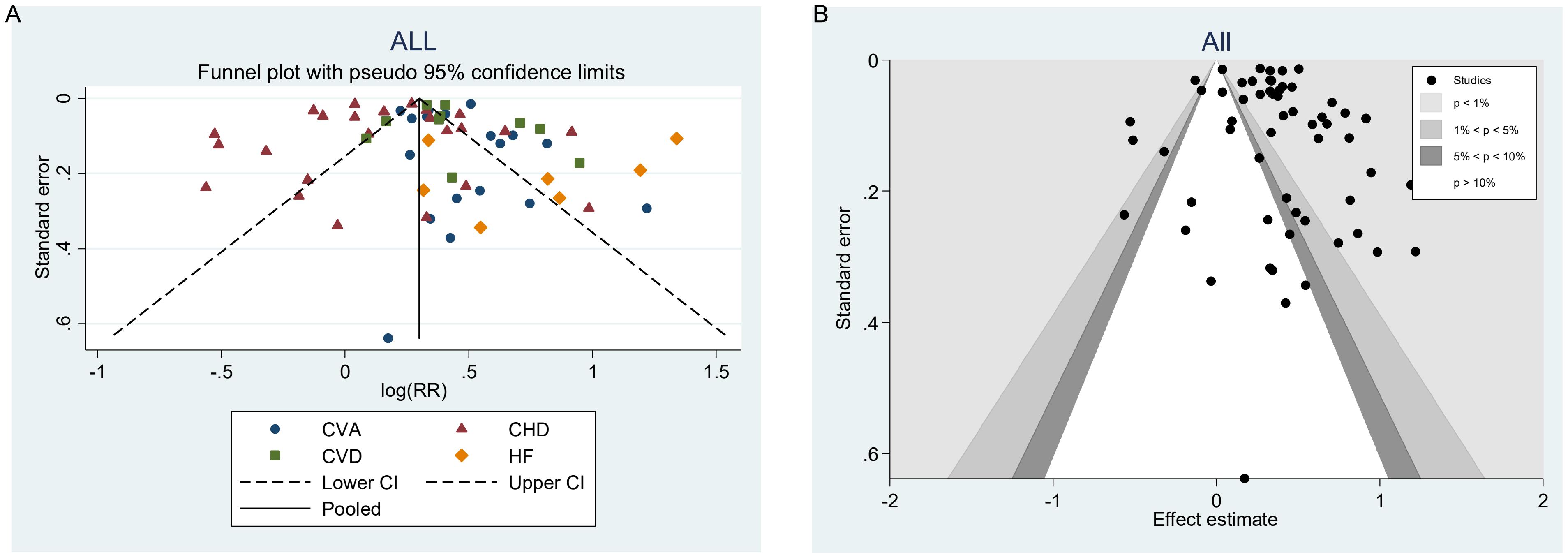

CVA – cerebrovascular accident, CHD – coronary heart disease, CVD – major cardiovascular events, HF – heart failure

Fig O: Funnel plots for visual assessment of publication bias for studies reporting relative risk of CVD incidence for schizophrenia and bipolar disorder compared with controls, all cardiovascular disease

When included results from all studies are considered together, the funnel plot (A) suggests some asymmetry on the left-hand side, within the pseudo 95% confidence limits, which could be missing smaller studies. These would fall inside the white (non-significant) area of the contour-enhanced funnel plot (B), so could suggest publication bias. However, the results of Egger's test (0.86, 95% CI: -0.91 to 2.63, p=0.336) were non-significant for small study effects.

Table A: Results of Egger's tests for publication bias, incidence outcomes

SMI	Incidence outcome	Bias (95% CI)	p-value
Schizophrenia	Cerebrovascular accident	-0.37 (-3.53, 2.79)	0.796
Schizophrenia	Coronary heart disease	-0.77 (-5.17, 3.64)	0.716
Schizophrenia	All circulatory disease events	Too few studies	-
Schizophrenia	Heart failure	Too few studies	-
Bipolar disorder	Cerebrovascular accident	Too few studies	-
Bipolar disorder	Coronary heart disease	Too few studies	-
Bipolar disorder	All circulatory disease events	Too few studies	-
Bipolar disorder	Heart failure	Too few studies	-
All SMI	Cerebrovascular accident	-0.05 (-1.90, 1.80)	0.957
All SMI	Coronary heart disease	0.40 (-2.69, 3.50)	0.791
All SMI	All circulatory disease events	1.48 (-2.13, 5.09)	0.374
All SMI	Heart failure	Too few studies	-
Schizophrenia	All CVD incidence outcomes	0.08 (-2.64, 2.81)	0.951
Bipolar disorder	All CVD incidence outcomes	**1.94** (0.16, 3.72)	0.034
All SMI	All CVD incidence outcomes	0.86 (-0.91, 2.63)	0.336

SMI – severe mental illness
Results where 95% confidence intervals exclude the null highlighted in ***bold***

References

1. Chaimani A, Mavridis D, Salanti G. A hands-on practical tutorial on performing meta-analysis with Stata. Evidence Based Mental Health. 2014;17(4):111. doi: 10.1136/eb-2014-101967.
