# Supplementary material for: Temporal trends in associations between severe mental illness and risk of cardiovascular disease: A systematic review and meta-analysis
Source: PLoS Med. 2022 Apr 19;19(4):e1003960. doi: 10.1371/journal.pmed.1003960 (PMC9017899; doi:10.1371/journal.pmed.1003960)
Supplement: S24 File — (DOCX) [file pmed.1003960.s024.docx]

# S24 File. Example calculations for estimating unreported cases

The number of people with SMI and number of controls for age groups 18-49 and 50-75 were unreported in a 2007 UK study of primary care records [1]. To estimate these numbers, the following methodology was used.

- The total number of SMI exposed and controls for ages 18+ were reported as 46136 and 300246 respectively
- The median and interquartile age ranges for exposed and controls were extracted from the study, which were median: 46.4, IQR: 32.2-63.7 and median: 38.0, IQR: 26.3-55.8, respectively.
- The distribution of the 2001 UK population aged 18 and over by single year of age was calculated by summing the results from all local authorities [2]. The percentages of the UK population for each year of age were calculated
- For UK as a whole, the median age was 45 and the IQR 32-60.5
- The UK population was thus slightly younger than the exposed population in the study but older than the control population
- Two sets of weighting factors were applied to the UK population percentages to adjust the age distribution so that the median and IQR roughly matched a) the exposed and b) the control study populations
- The weighted percentages were applied to the number of exposed and controls from the study (*i.e* 46136 and 300246) to give an estimated number exposed and controls for each year of age
- The estimated numbers of exposed and controls were summed for ages 18-49 and 50-75 to calculate the estimated numbers for these age groups
- The estimated exposed population numbers were further broken down into schizophrenia and bipolar disorder using the percentages reported in the paper (40.2% schizophrenia and 23.3% bipolar disorder)

## References

1. Osborn D, Levy G, Nazareth I, Petersen I, Islam A, King M. Relative risk of cardiovascular and cancer mortality in people with severe mental illness from the United Kingdom's general practice research database. Arch Gen Psychiatr. 2007;64(2):242 - 9. PubMed PMID: doi:10.1001/archpsyc.64.2.242.

2. Office for National Statistics. Estimates of the population for the UK, England and Wales, Scotland and Northern Ireland. 2020.
